# Bifunctional sulfilimines enable synthesis of multiple N-heterocycles from alkenes

**DOI:** 10.1038/s41557-022-00997-y

**Published:** 2022-07-25

**Authors:** Qiang Cheng, Zibo Bai, Srija Tewari, Tobias Ritter

**Affiliations:** 1 Max-Planck-Institut für Kohlenforschung, Mülheim an der Ruhr, Germany; 2 Institute of Organic Chemistry, RWTH Aachen University, Aachen, Germany

**Keywords:** Synthetic chemistry methodology, Synthetic chemistry methodology, Photocatalysis, Photocatalysis

## Abstract

Intramolecular cyclization of nitrogen-containing molecules onto pendant alkenes is an efficient strategy for the construction of N-heterocycles, which are of paramount importance in, for example, pharmaceuticals and materials. Similar intermolecular cyclization reactions, however, are scarcer for nitrogen building blocks, including N-centred radicals, and divergent and modular versions are not established. Here we report the use of sulfilimines as bifunctional N-radical precursors for cyclization reactions with alkenes to produce N-unprotected heterocycles in a single step through photoredox catalysis. Structurally diverse sulfilimines can be synthesized in a single step, and subsequently engage with alkenes to afford synthetically valuable five-, six- and seven-membered heterocycles. The broad and diverse scope is achievable by a radical-polar crossover annulation enabled by the bifunctional character of the reagents, which distinguishes itself from all other N-centred-radical-based reactions. The modular synthesis of the sulfilimines allows for larger structural diversity of N-heterocycle products than is currently achievable with other single cyclization methods.

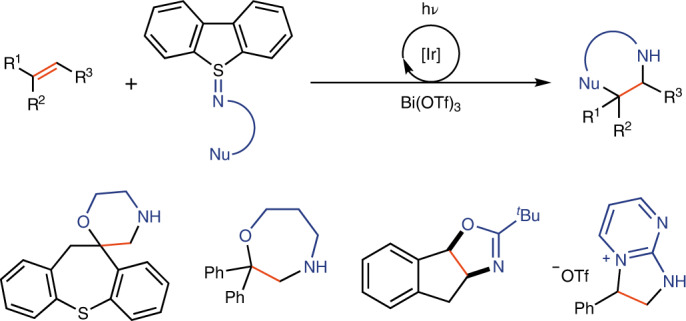

## Abstract (web summary)

Intermolecular cyclization reactions using nitrogen-containing building blocks are scarce. Now, bifunctional sulfilimines have been shown to enable the modular construction of a diverse range of N-heterocycles by reacting with alkenes in a single photocatalysed step. Both sulfilimines and alkenes are easily accessible, providing access to a wide range of N-heterocycles with different ring types, ring sizes and substituents on the skeleton.

## Main

**Fig. 1:**
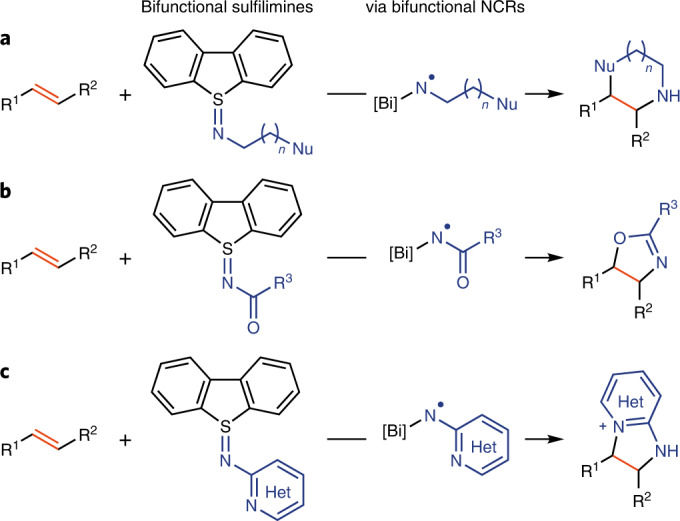
Bifunctional sulfilimines for synthesis of various N-heterocycles. **a**–**c**, In this article, bifunctional sulfilimines that feature both nitrogen-radical and polar reactivity have been developed to react with alkenes, giving a divergent and modular approach to versatile N-heterocycles including morpholines, piperazines and oxazepanes (**a**), dihydrooxazoles (**b**) and dihydroimidazoles (**c**). Nu, nucleophilic group; Het, heteroaryl.

Partially and fully saturated N-heterocycles are of high synthetic value, and can for example be accessed by cyclization onto vinyl sulfonium reagents^1–4^, yet their direct synthesis by cyclization reactions to olefins is not generally established. The development of SnAP reagents is an excellent example of how bifunctional reagents^5^ can quickly generate useful N-heterocycle diversity through cyclization onto aldehydes^6^, but similar reactivity via a single-step-reaction with alkenes has not been developed. In 2001, Oshima and co-workers reported a radical chain process using *N*-allyl-*N*-chlorotosylamide as a nitrogen-radical precursor for reaction with alkenes to generate *N*-tosylpyrrolidines^7^. Shi and co-workers developed diaziridinones as nitrogen-centred radical (NCR) precursors for ring expansion with alkenes to generate *N*-*tert*-butyl-protected imidazolidinones under copper catalysis^8^. Xu and co-workers reported the use of functionalized hydroxylamines to generate carbamate-based NCRs for the construction of oxazolidinones under iron catalysis^9^. Another example uses *N*-fluorobenzenesulfonimide specifically for the construction of sultams under copper catalysis^10^. Despite the large synthetic utility, these methods can only generate a single, specific N-heterocycle, typically with an electron-withdrawing nitrogen-protecting group that may be challenging to remove. No single method appears to be available that can generate several different types of N-heterocycles from olefins^11^. Here we fill this conceptual void and demonstrate a modular approach to access a large variety of different, synthetically valuable heterocycles that are not currently accessible via other NCRs or polar reactions from simple alkenes in a single step (Fig. 1)^12^. For example, while morpholine syntheses are well known, their one-step synthesis from olefins has not been reported^13^.

NCRs are important intermediates in C–N bond formation reactions^14–19^. Due to the high bond dissociation energy of the N–H bond (107 kcal mol^−1^ for ammonia)^20^, NCRs can function as reactive species for intramolecular hydrogen-atom transfer, for example to generate pyrrolidine derivatives^21^, as in the Hofmann–Löffler–Freytag reaction^22,23^. NCRs can also function as electrophilic radicals^24^, adding to electron-rich *π* systems in alkenes or arenes to form alkyl or aryl amines. Synthetic applications of such reactivity have resulted in successful hydroamination^25,26^, aminooxygenation^27–30^, aminofluorination^31–33^, carboamination^34,35^ and aminoazidation^36,37^, all of which introduce two different functional groups to an alkene in a single step. Despite the advance and scope of difunctionalization reactions with NCRs, intermolecular cyclization reactions to furnish N-heterocycles are still challenging because most nitrogen-radical precursors contain sulfonyl or similar groups on the nitrogen to enhance the electrophilicity of the respective NCRs^15,24^.

## Results and discussion

**Fig. 2:**
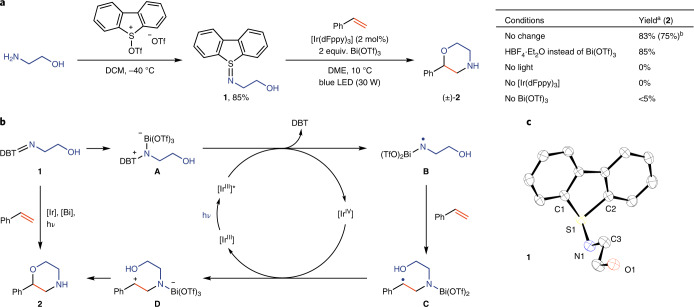
Synthesis of sulfilimine 1, reaction optimization and proposed mechanism of the cyclization reaction. **a**, The sulfilimine **1** was obtained from the reaction of aminoethanol and triflic-anhydride-activated dibenzothiophene-*S*-oxide in a single step. The reaction optimization shows that both photocatalyst and acid additive are essential for the reactivity; ^a^Yield determined from^1^H NMR with CH_2_Br_2_ as an internal standard. ^b^Isolated yield in parenthesis. DCM, dichloromethane; DME, 1,2-dimethoxyethane. **b**. A radical-polar-crossover annulation process was proposed. The acid additive plays a crucial role in activation of sulfilimine **1** to generate bifunctional NCR **B** as a key intermediate. DBT, dibenzothiophene. **c**. X-ray crystal structure of **1** (Supplementary Tables 7 and 8; hydrogen atoms are omitted for clarity). Selected bond distances and angles: S(1)–N(1), 1.591(2) Å; C(1)–S(1)–C(2), 88.85(9)°, C(3)–N(1)–S(1), 116.97(14)°.

Our goal was to generate a stable reagent that could be transformed into an electrophilic NCR under conditions that would tolerate the presence and reactivity of a pendant nucleophile for subsequent ring closure. While, a priori, several NCR precursors could meet such a goal, one reason why such a compound class has not yet been disclosed may be the synthetic challenge to make these reagents due to undesired cross-reactivity of the pendant nucleophile or undesired reactivity of the nitrogen-activating group. For example, the chloride released upon NCR formation from *N*-chloroamonium salts may outcompete a pendant hydroxyl group for addition^38^. Here we report the use of sulfilimines, a substrate class that has previously been used in other transformations^39^, to address this synthetic challenge. The bifunctional sulfilimine **1** was obtained directly from commercially available reagents in a reaction of aminoethanol and dibenzothiophene-*S*-oxide activated by triflic anhydride (Fig. 2a). Irradiation of a photoredox catalyst in the presence of sulfilimine **1**, acid and styrene results in phenylmorpholine formation (Fig. 2a). Light and photoredox catalyst are essential for the reactivity (Supplementary Tables 1–6). Both Brønsted acids and Lewis acids accelerate cyclization, possibly due to more efficient single electron transfer (SET) from the excited photoredox catalyst to the sulfilimine coordinated to acid (Fig. 2b and Supplementary Table 1). According to the recorded cyclic voltammogram of sulfilimine **1** (Supplementary Fig. 7), no reduction peak was observed within the evaluated potential, which indicates that mesolytic cleavage of the S=N bond in sulfilimine **1** by initial SET to **1** to the corresponding NCR is slow with standard photocatalysts. In contrast, the Bi(OTf)_3_-coordinated sulfilimine **1** (**A**) exhibits a high reduction potential (*E*_p_ = −0.4 V versus Ag/AgCl, Supplementary Fig. 7), so that fast SET with the excited iridium photocatalyst (*E*_1/2_(Ir^III^*/Ir^IV^) = −1.28 V vs saturated calomel electrode)^40^ can be observed, to form **B** (Fig. 2b). Bi(OTf)_3_ was identified as optimal because Brønsted acids could result in cationic polymerization of activated olefins such as electron-rich styrenes (Supplementary Table 2)^41^. In addition, both Lewis and Brønsted acids could be responsible for rendering the amine radical electrophilic for polarity-matched addition to the electron-rich *π* system of the olefin^42–44^. A stoichiometric amount of Bi(OTf)_3_ is required due to the basicity of the products. A conceptual advantage of the sulfilimines over other NCR precursors is their ability to enable easy introduction of pendant nucleophilic functional groups on NCRs, and directly afford unprotected N–H nitrogen heterocycles in a single step without the need for covalent activating groups or a deprotection step. Upon addition to the *π* system, the oxidized photoredox catalyst can oxidize the resulting carbon radical **C** for subsequent intramolecular nucleophilic attack (**D**) of the pendant nucleophile and regeneration of the photoredox catalyst resting state (Fig. 2b).

Various bifunctional sulfilimine reagents can be synthesized in a single step (Tables 1 and 2). Primary amines including aminoalcohols, diamines and aminopyridines react to produce iminodibenzothiophenes with pendant hydroxyl (**3**–**6**), amide (**7**, **8**) or pyridinyl groups (**9**, **10**). The diversity of the suitable sulfilimines for heterocycle synthesis was extended by reaction of the parent iminodibenzothiophene (**11**) with acylating reagents, which gives access to other classes of sulfilimines, such as those derived from amides, pyrimidines and triazines from acid chlorides (**12**–**15**), chloropyrimidines (**16**) and chlorotriazines (**17**), respectively. All sulfilimines shown, with the exception of **6**, are easily handled solids and are stable in ambient atmosphere without detectable decomposition for at least three months; while also stable, **6** was isolated as an oil.

**Table 1:**
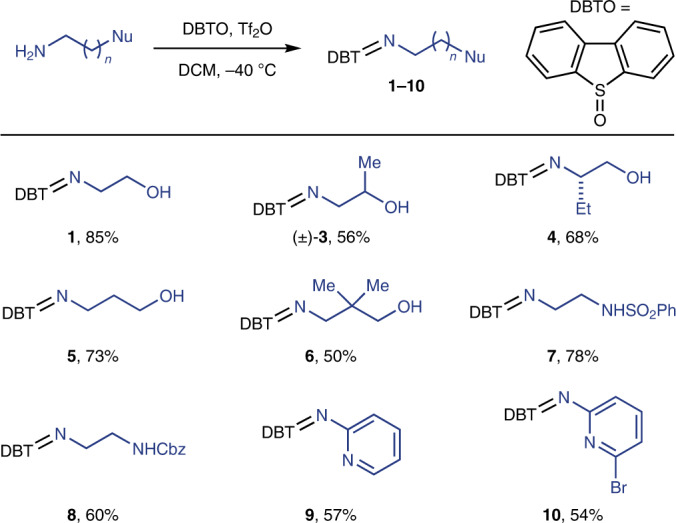
Synthesis of bifunctional sulfilimines from amines

Reaction conditions: 1.0 equiv. of dibenzothiophene-*S*-oxide, 1.1 equiv. of triflic anhydride and 2.5 equiv. of amine in DCM (0.1 M) at −40 °C.

**Table 2:**
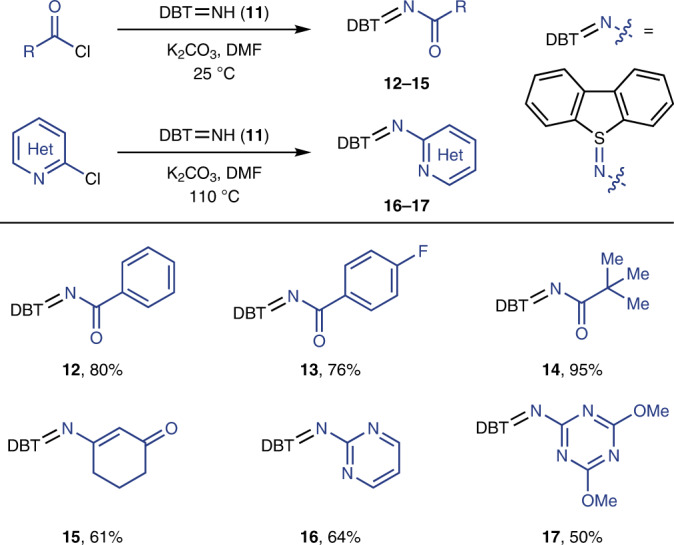
Synthesis of bifunctional sulfilimines from dibenzothiophene-*S*-imine

Reaction conditions: 1.0 equiv. of dibenzothiophene-*S*-imine (**11**), 2.0 equiv. of K_2_CO_3_ and 1.5 equiv. of acid chloride in DMF (0.1 M) at 25 °C for 3 h, or 2.0 equiv. of chloroazine in DMF (0.1 M) at 110 °C for 6 h.

**Fig. 3:**
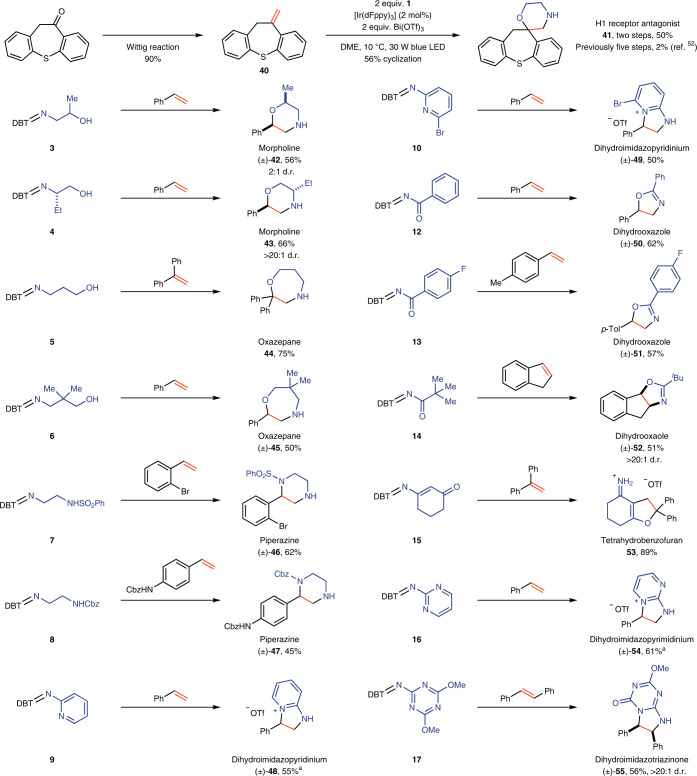
Scope of sulfilimines and synthetic application. Reaction conditions: alkene (0.2 mmol), sulfilimine (0.4 mmol), Bi(OTf)_3_ (0.4 mmol), [Ir(dFppy)_3_] (2 mol%) in DME (1 ml) at 10 °C under a 30 W blue LED. ^a^Methyl methoxyacetate (1 ml) as solvent instead of DME, and base workup is not applied due to easier purification of these products in ionic form. Other types of N-heterocycles, including oxazepanes, piperazines, dihydrooxazoles, dihydroimidazopyridiniums and dihydroimidazotriazinones, are accessible with the modular cyclization approach. A concise route to H1 receptor antagonist **41** has been developed based on the cyclization method.

Cyclization of a variety of sulfilimines with a variety of electron-rich olefins gives access to a large family of diverse, synthetically valuable heterocycles in a single step (Table 3 and Fig. 3). The products can be isolated without protecting groups on nitrogen, but in situ protection of nitrogen with a *tert*-butyloxycarbonyl (Boc) group is facile, if desired, as shown for **18**. Styrene derivatives that are prone to cationic polymerization under acidic conditions^45^ such as **19** and **22** can selectively react with the bifunctional sulfilimine **1** to produce morpholines in 78% and 55% yield, respectively. Olefins with heteroaryl substituents, such as indoles (**24**), pyridines (**25**) and benzothiophenes (**28**), are well tolerated. The putative bismuth(III)-coordinated amine radicals undergo addition reactions chemoselectively to alkenes in the presence of electron-rich arenes (**19**), allylic hydrogens (**23**, **27**, **30**–**33**, **35**) and even ether (DME) as solvent. Such functional groups are often reactive with other NCRs either via radical addition^46,47^ or via hydrogen-atom transfer processes^48–50^. In addition to α-styrenes, 1,1-disubstituted alkenes (**37**) can also undergo cyclization with sulfilimine **1** to produce morpholine heterocycles with a quaternary centre, although for most other investigated alkyl-substituted alkenes that feature allylic hydrogen atoms, allylic amination was observed^44^. For 1,2-disubstituted alkenes that bear a group that can stabilize a positive charge, high diastereoselectivity is observed for cyclization (**26**, **27**), providing 2,3-disubsituted morpholines. Both *trans*- and *cis*-propenylbenzenes produce the same product with the same diastereoselectivity, which supports the proposed intermediacy of NCRs. High regioselectivity is also obtained in reactions with dienes, such as **34** and **35**, as cyclization reaction only occurs at the terminal alkene of the diene, producing alkenyl-substituted morpholines. When alkyl-substituted dienes are used, the thermodynamically more stable *trans*-olefin is obtained as major product (**35**). In the case of styrene-derived dienes (**34**), a known photocatalysed isomerization^51^ occurs to produce *cis*- and *trans*-styrenylmorpholines. Cyclic alkenes afford bicyclic and tricyclic morpholines. Highly diastereoselective formation of morpholine derivatives with fused rings is achieved when endocyclic olefins such as norbornene (**29**), 1-phenyl-1-cyclohexene (**30**) and indenes (**31**, **32**) are used. Exocyclic olefins such as 7-methyl-4-methylenechromane (**33**) and camphene (**37**) are also suitable reaction partners, producing spirocyclic morpholines. These polycyclic heterocycles can be constructed selectively in a single step from the corresponding alkenes, and are not readily accessible via other synthetic methods. Olefins that would afford cations upon radical addition and oxidation that are not sufficiently stabilized, such as in α olefins and 1,2-disubstituted alkenes without stabilizing groups, such as an aryl or vinyl substituent, cannot participate in the reaction (Supplementary Fig. 1), whereas styrene-like, 1,1-disubstituted and diene-based olefins participate successfully. The method can be used for late-stage diversification (**38**, **39**). To further demonstrate the synthetic value of the method, we accomplished a concise synthesis of H1 receptor antagonist **41** (Fig. 3). The modular approach allows us to construct the key morpholine structure directly from alkene **40**, which substantially increased the total yield and reduced the step count compared to the previously published procedure^52^. The reaction requires the use of a stoichiometric amount of dibenzothiophene heterocycle, which, however, can be recycled after successful cyclization; for example, 92% of dibenzothiophene was reisolated after formation of **2**.

**Table 3:**
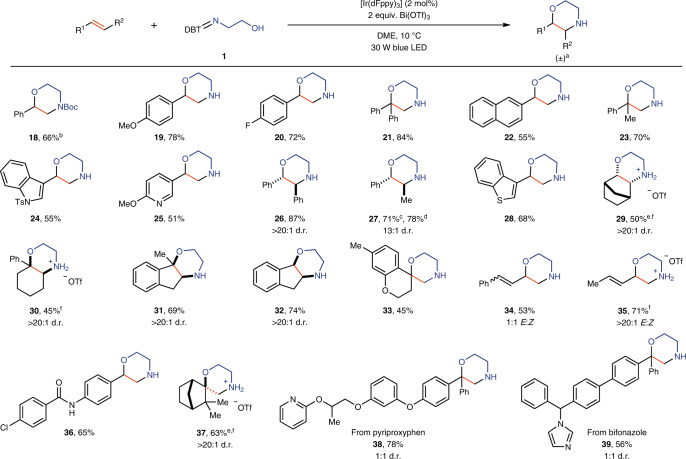
Scope of alkenes for synthesis of morpholine derivatives

Reaction conditions: alkene (0.2 mmol), **1** (0.4 mmol), Bi(OTf)_3_ (0.4 mmol), [Ir(dFppy)_3_] (2 mol%) in DME (1 ml) at 10 °C under a 30 W blue light-emitting diode (LED) for 6 h. ^a^All chiral products are obtained as racemic mixtures. ^b^In situ Boc protection of the morpholine product after irradiation of the reaction mixture for 6 h by addition of 6 equiv. of Et_3_N and 3 equiv. of Boc_2_O. ^c^(*E*)-Propenylbenzene is used. ^d^(*Z*)-Propenylbenzene is used. ^e^Alkene (0.8 mmol), **1** (0.2 mmol), Bi(OTf)_3_ (0.2 mmol). ^f^Base workup is not applied due to easier purification of these products in protonated form.

The modular approach and the accessibility of various bifunctional sulfilimines enables the synthesis of other types of N-heterocycles under the same reaction conditions by simply changing the substituents on the sulfilimines (Fig. 3). Disubstituted morpholines are obtained when sulfilimines such as **3** or **4** react with alkenes. Although low diastereoselectivity (2:1) was observed when the stereocentre is α to the hydroxy substituent, high diastereoselectivity (>20:1) is obtained when the stereocentre is α to the nitrogen substituent. Other sulfilimine reagents (**5**–**8**) enable the construction of oxazepanes (**44**, **45**) and piperazines (**46**, **47**) in synthetically useful yields. We subsequently explored the generality of our modular approach to N-heterocycles with sulfilimines derived from amines other than alkyl amines. Acyl-amine-derived sulfilimines (**12**–**14**) can also function as bifunctional reagents, which undergo cyclization with alkenes under the same reaction conditions to produce dihydrooxazoles (**50**–**52**)^53,54^. An intriguing reactivity was discovered when using sulfilimine **15** as substrate, providing tetrahydrobenzofuran **53** exclusively in 89% yield instead of an N-heterocycle. A plausible rationale is that the generated enamine NCR reacts to the more stable carbon-centred radical, which is then involved in annulation with 1,1-diphenylethylene (Supplementary Fig. 13). The photocatalytic annulation strategy can also be applied to heteroaryl-amine-derived sulfilimines (**9**, **10**, **16**, **17**), generating electrophilic arylamine radicals that are reactive for cyclization with alkenes to form dihydroimidazole derivatives (**48**, **49**, **54**, **55**). In the case of sulfilimine **17**, the initially formed triazinium underwent hydrolysis under basic conditions to form triazinone **55**. NCRs with alkyl, aryl and acyl substituents on nitrogen can be accessed via the same photocatalytic method, which is difficult to achieve with other NCR precursors^55,56^. The scope of heterocycles presented herein exceeds that of other reported single methods^12^.

**Fig. 4:**
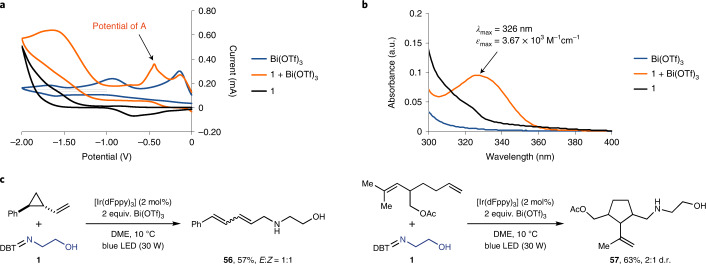
Mechanistic investigations. **a**, Cyclic voltammetry for Bi(OTf)_3_, sulfilimine **1** and a 1:1 mixture of Bi(OTf)_3_ and **1** in acetonitrile under a scan rate of 100 mV s^–1^. A new reduction peak at *E*_p_ = −0.4 V versus Ag/AgCl was observed when using a 1:1 mixture of Bi(OTf)_3_ and **1**. **b**, Ultraviolet–visible spectra of Bi(OTf)_3_, sulfilimine **1** and a 1:1 mixture of Bi(OTf)_3_ and **1** in DME (2.5 × 10^–5^ M). A new absorption peak at 326 nm was observed when using a 1:1 mixture of Bi(OTf)_3_ and **1**. Both cyclic voltammetry and ultraviolet–visible spectra indicate a direct interaction of Bi(OTf)_3_ with **1**, which plays a key role in activation of sulfilimine **1** for the generation of the corresponding NCR. **c**, A radical clock experiment using both 2-vinylcyclopropylbenzene and 1,6-diene shows that the reactions proceed via generation of NCRs.

Preliminary mechanistic experiments are in agreement with the proposed strategy shown in Fig. 2b (Supplementary Figs. 2–12). A 1:1 mixture of sulfilimine **1** and Bi(OTf)_3_ results in a new peak potential that is absent in both **1** and Bi(OTf)_3_ alone, which we assign to the **1**–Bi(OTf)_3_ adduct **A** as observed in the cyclic voltammogram (Fig. 4a). The high reduction potential (*E*_p_ = −0.4 V versus Ag/AgCl) may be responsible for a fast SET from the excited iridium photocatalyst, while reduction of **1** by itself was not observed. The existence of adduct **A** is further substantiated by ultraviolet–visible spectroscopy through a new absorption maximum at 326 nm (Fig. 4b). Radical clock experiments with 2-vinylcyclopropylbenzene and a 1,6-diene under optimized reaction conditions with sulfilimine **1** produce ring-opened product **56** and cyclization product **57**, respectively, in agreement with NCRs (Fig. 4c).

## Conclusion

Photocatalysed modular synthesis has enabled the construction of various N-heterocycles with different ring types, ring sizes and substituents on the skeleton in a single step by reaction of easily available bifunctional sulfilimines and alkenes. The scope of heterocycles provided here is broader than that of other reported single methods for N-heterocycle synthesis from olefins.

## Methods

### General procedure for cyclization

Under a nitrogen atmosphere, to a 4 ml borosilicate vial equipped with a magnetic stir bar were added alkene (if solid) (0.200 mmol, 1.00 equiv.), sulfilimine (0.400 mmol, 2.00 equiv.), [Ir(dFppy)_3_] (3.0 mg, 4.0 µmol, 2.0 mol%), Bi(OTf)_3_ (262 mg, 0.400 mmol, 2.00 equiv.), DME (1 ml, c = 0.2 M), and alkene (if liquid) (0.200 mmol, 1.00 equiv.). The vial was sealed with a septum cap and irradiated for 6 h at 10 °C using a photoreactor equipped with a blue LED module (KT-Elektronik, ‘100 W Power LED blau 450 nm Aquarium’, 450 nm, 30 W), cooled with two Peltier elements (TEC1-12706). Then, the reaction mixture was concentrated to dryness. The residue was dissolved in DCM (5 ml) and washed with saturated aqueous sodium carbonate solution (5 ml). The aqueous phase was extracted with DCM (2 × 5 ml). The organic phase was dried over Na_2_SO_4_ and filtered, and the solvent was removed under reduced pressure. The residue was purified by chromatography on silica gel eluting with CH_2_Cl_2_/MeOH (50/1–10/1 v/v) to afford the cyclization product.

Note: The reaction is air sensitive. The Schlenk technique was used to avoid air. For simplicity, in our research, we have opted to execute the transformation for most compounds in a glovebox. Control experiments showed that yields were within the error of measurement if the reaction was carried out using a glovebox or the Schlenk technique.

## Online content

Any methods, additional references, Nature Research reporting summaries, source data, extended data, supplementary information, acknowledgements, peer review information; details of author contributions and competing interests; and statements of data and code availability are available at 10.1038/s41557-022-00997-y.

## Supplementary information

Supplementary Information Experimental procedures, product characterization, mechanistic studies, Supplementary Figs. 1–13 and Tables 1–8.

Supplementary Data 1 Crystallographic data for compound **1**; CCDC reference 2101016

## Data Availability

All the data generated or analysed during this study are included in this article and its Supplementary Information. Crystallographic data for the structure reported in this article have been deposited at the Cambridge Crystallographic Data Centre, under deposition numbers 2101016 (**1**). Copies of the data can be obtained free of charge via https://www.ccdc.cam.ac.uk/structures/.
