# Supplementary material for: Bifunctional sulfilimines enable synthesis of multiple N-heterocycles from alkenes
Source: Nat Chem. 2022 Jul 25;14(8):898–904. doi: 10.1038/s41557-022-00997-y (PMC9359915; doi:10.1038/s41557-022-00997-y)
Supplement: Supplementary file 1 — Experimental procedures, product characterization, mechanistic studies, Supplementary Figs. 1–13 and Tables 1–8. [file 41557_2022_997_MOESM1_ESM.pdf]

nature portfolio

https://doi.org/10.1038/s41557-022-00997-y

Supplementary information

Bifunctional sulfilimines enable synthesis of multiple N-heterocycles from alkenes

In the format provided by the authors and unedited

TABLE OF CONTENTS

TABLE OF CONTENTS 1

MATERIALS AND METHODS 9

EXPERIMENTAL DATA 11

General procedure and reaction condition optimization for cyclization 11

General procedure for cyclization 11

Table 1. Screening of acid additive [a] 11

Table 2. Investigation of side reactions of different acid additives [a] 12

Table 3. Screening of solvent [a] 12

Table 4. Screening of photocatalyst [a] 13

Table 5. Screening of temperature [a] 14

Table 6. Screening of sulfilimine skeleton [a] 14

Substrates synthesis and cyclization reactions 15

Sulfilimine **1** 15

2-Phenylmorpholine **2** 16

Sulfilimine **3** 16

Sulfilimine **4** 17

Sulfilimine **5** 18

Sulfilimine **6** 19

Sulfilimine **7** 19

Sulfilimine **8** 20

Sulfilimine **9** 21

Sulfilimine **10** 22

Dibenzothiophen-5-imine **11** 22

Sulfilimine **12** 23

Sulfilimine **13** 24

Sulfilimine **14** 25

Sulfilimine **15** 25

Sulfilimine **16** 26

Sulfilimine **17** 27

N-Boc-2-phenylmorpholine **18** 27

2-(4-Methoxyphenyl)morpholine **19** 28

2-(4-Fluorophenyl)morpholine **20** 29

2,2-Diphenylmorpholine **21** 30

2-(Naphthalen-2-yl)morpholine **22** 30

2-Methyl-2-phenylmorpholine **23** 31
Indole-3-morpholine **24** 32
2-(6-Methoxypyridin-3-yl)morpholine **25** 32
2,3-Diphenylmorpholine **26** 33
3-Methyl-2-phenylmorpholine **27** 34
2-(Benzothiophen-3-yl)morpholine **28** 36
Bicyclo[2.2.1]heptane derived morpholine **29** 36
Octahydrobenzo[1,4]oxazine **30** 37
9b-Methyl hexahydroindeno[1,4]oxazine **31** 38
Hexahydroindeno[1,4]oxazine **32** 39
Spiro[chromane-4,2'-morpholine] **33** 39
2-Styrylmorpholine **34** 40
2-(4-Benzamide)phenylmorpholine **35** 42
(E)-2-(Propen-1-yl)morpholine **36** 41
Spirobicyclo[2.2.1]heptane-2,2'-morpholine **37** 42
Pyriproxyphen derived alkene **S1** 43
Pyriproxyphen derived morpholine **38** 44
Bifonazole derived alkene **S2** 45
Bifonazole derived morpholine **39** 45
Dihydrodibenzothiepine **40** 46
Spiro[dibenzothiepine-10,2'-morpholine] **41** 47
2-Methyl-6-phenylmorpholine **42** 47
5-Ethyl-2-phenylmorpholine **43** 48
2,2-Diphenyl-1,4-oxazepane **44** 49
6,6-Dimethyl-2-phenyl-1,4-oxazepane **45** 50
N-Phenylsulfonylpiperazine **46** 50
N-Cbz-piperazine **47** 51
Dihydroimidazopyridinium **48** 52
Dihydroimidazopyridinium **49** 53
Dihydrooxazole **50** 53
Dihydrooxazole **51** 54
Dihydroindeno[2,1-d]oxazole **52** 55
2,2-Diphenyltetrahydrobenzofuran **53** 56
Dihydroimidazopyrimidinium **54** 56
Dihydroimidazo[1,3,5]triazinone **55** 57
Recycling of DBT 58
Reaction of DBT=NH 58

Mechanistic investigations for cyclization 60

Stern-Volmer Luminescence Quenching Studies 60
Cyclic voltammograms of **1**, $Bi(OTf)_3$, and a combination of **1** and $Bi(OTf)_3$ 62
UV-vis spectra 64
Radical trap and radical clock experiments 66
Cyclopropane ring opened product **56** 68
1,6-Diene radical trap product **57** 69
Rationale for the formation of product **53** 70

X-RAY CRYSTALLOGRAPHIC ANALYSIS 71
X-Ray Chrystallographic Data **1** (CCDC 2101016) 71
Experimental 71
Table 7. Crystal data and structure refinement. 71
Table 8. Bond lengths [Å] and angles [°]. 73

SPECTROSCOPIC DATA 75
^{1}H NMR of sulfilimine **1** 75
^{13}C NMR of sulfilimine **1** 76
^{1}H NMR of 2-phenylmorpholine **2** 77
^{13}C NMR of 2-phenylmorpholine **2** 78
^{1}H NMR of sulfilimine **3** 79
^{13}C NMR of sulfilimine **3** 80
^{1}H NMR of sulfilimine **4** 81
^{13}C NMR of sulfilimine **4** 82
^{1}H NMR of sulfilimine **5** 83
^{13}C NMR of sulfilimine **5** 84
^{1}H NMR of sulfilimine **6** 85
^{13}C NMR of sulfilimine **6** 86
^{1}H NMR of sulfilimine **7** 87
^{13}C NMR of sulfilimine **7** 88
^{1}H NMR of sulfilimine **8** 89
^{13}C NMR of sulfilimine **8** 90
^{1}H NMR of sulfilimine **9** 91

^{13}C NMR of sulfilimine **9** 92

^{1}H NMR of sulfilimine **10** 93

^{13}C NMR of sulfilimine **10** 94

^{1}H NMR of dibenzothiophen-5-imine **11** 95

^{13}C NMR of dibenzothiophen-5-imine **11** 96

^{1}H NMR of sulfilimine **12** 97

^{13}C NMR of sulfilimine **12** 98

^{1}H NMR of sulfilimine **13** 99

^{13}C NMR of sulfilimine **13** 100

^{19}F NMR of sulfilimine **13** 101

^{1}H NMR of sulfilimine **14** 102

^{13}C NMR of sulfilimine **14** 103

^{1}H NMR of sulfilimine **15** 104

^{13}C NMR of sulfilimine **15** 105

^{1}H NMR of sulfilimine **16** 106

^{13}C NMR of sulfilimine **16** 107

^{1}H NMR of sulfilimine **17** 108

^{13}C NMR of sulfilimine **17** 109

^{1}H NMR of N-Boc-2-phenylmorpholine **18** 110

^{13}C NMR of N-Boc-2-phenylmorpholine **18** 111

^{1}H NMR of 2-(4-methoxyphenyl)morpholine **19** 112

^{13}C NMR of 2-(4-methoxyphenyl)morpholine **19** 113

^{1}H NMR of 2-(4-fluorophenyl)morpholine **20** 114

^{13}C NMR of 2-(4-fluorophenyl)morpholine **20** 115

^{19}F NMR of 2-(4-fluorophenyl)morpholine **20** 116

^{1}H NMR of 2,2-diphenylmorpholine **21** 117

^{13}C NMR of 2,2-diphenylmorpholine **21** 118

^{1}H NMR of 2-(naphthalen-2-yl)morpholine **22** 119

^{13}C NMR of 2-(naphthalen-2-yl)morpholine **22** 120

^{1}H NMR of 2-methyl-2-phenylmorpholine **23** 121

^{13}C NMR of 2-methyl-2-phenylmorpholine **23** 122

^{1}H NMR of indole-3-morpholine **24** 123

^{13}C NMR of indole-3-morpholine **24** 124

^{1}H NMR of 2-(6-methoxypyridin-3-yl)morpholine **25** 125

^{13}C NMR of 2-(6-methoxypyridin-3-yl)morpholine **25** 126

^{1}H NMR of 2,3-diphenylmorpholine **26** 127

^{13}C NMR of 2,3-diphenylmorpholine **26** 128

^{1}H NMR of 3-methyl-2-phenylmorpholine **27** 129

^{13}C NMR of 3-methyl-2-phenylmorpholine **27** 130

^{1}H NMR of 2-(benzothiophen-3-yl)morpholine **28** 131

^{13}C NMR of 2-(benzothiophen-3-yl)morpholine **28** 132

^{1}H NMR of bicyclo[2.2.1]heptane derived morpholine **29** 133

^{13}C NMR of bicyclo[2.2.1]heptane derived morpholine **29** 134

^{19}F NMR of bicyclo[2.2.1]heptane derived morpholine **29** 135

COSY of bicyclo[2.2.1]heptane derived morpholine **29** 136

HSQC of bicyclo[2.2.1]heptane derived morpholine **29** 137

HMBC of bicyclo[2.2.1]heptane derived morpholine **29** 138

NOESY of bicyclo[2.2.1]heptane derived morpholine **29** 139

^{1}H NMR of octahydrobenzo[1,4]oxazine **30** 140

^{13}C NMR of octahydrobenzo[1,4]oxazine **30** 141

^{19}F NMR of octahydrobenzo[1,4]oxazine **30** 142

COSY of octahydrobenzo[1,4]oxazine **30** 143

HSQC of octahydrobenzo[1,4]oxazine **30** 144

HMBC of octahydrobenzo[1,4]oxazine **30** 145

NOESY of octahydrobenzo[1,4]oxazine **30** 146

^{1}H NMR of 9b-methyl hexahydroindeno[1,4]oxazine **31** 147

^{13}C NMR of 9b-methyl hexahydroindeno[1,4]oxazine **31** 148

HSQC of 9b-methyl hexahydroindeno[1,4]oxazine **31** 149

NOESY of 9b-methyl hexahydroindeno[1,4]oxazine **31** 150

^{1}H NMR of hexahydroindeno[1,4]oxazine **32** 151

^{13}C NMR of hexahydroindeno[1,4]oxazine **32** 152

^{1}H NMR of spiro[chromane-4,2'-morpholine] **33** 153

^{13}C NMR of spiro[chromane-4,2'-morpholine] **33** 154

^{1}H NMR of 2-styrylmorpholine **34** 155

^{13}C NMR of 2-styrylmorpholine **34** 156

^{1}H NMR of 2-(4-benzamide)phenylmorpholine **35** 160

^{13}C NMR of 2-(4-benzamide)phenylmorpholine **35** 161

^{1}H NMR of (E)-2-(Propen-1-yl)morpholine **36** 157

^{13}C NMR of (E)-2-(Propen-1-yl)morpholine **36** 158

^{19}F NMR of (E)-2-(Propen-1-yl)morpholine **36** 159

^{1}H NMR of spirobicyclo[2.2.1]heptane-2,2'-morpholine **37** 162

^{13}C NMR of spirobicyclo[2.2.1]heptane-2,2'-morpholine **37** 163

^{19}F NMR of spirobicyclo[2.2.1]heptane-2,2'-morpholine **37** 164

COSY of spirobicyclo[2.2.1]heptane-2,2'-morpholine **37** 165

HSQC of spirobicyclo[2.2.1]heptane-2,2'-morpholine **37** 166

HMBC of spirobicyclo[2.2.1]heptane-2,2'-morpholine **37** 167

NOESY of spirobicyclo[2.2.1]heptane-2,2'-morpholine **37** 168

^{1}H NMR of pyriproxyphen derived alkene **S1** 169

^{13}C NMR of pyriproxyphen derived alkene **S1** 170

^{1}H NMR of pyriproxyphen derived morpholine **38** 171

^{13}C NMR of pyriproxyphen derived morpholine **38** 172

^{1}H NMR of bifonazole derived alkene **S2** 173

^{13}C NMR of bifonazole derived alkene **S2** 174

^{1}H NMR of bifonazole derived morpholine **39** 175

^{13}C NMR of bifonazole derived morpholine **39** 176

^{1}H NMR of dihydrodibenzothiepine **40** 177

^{13}C NMR of dihydrodibenzothiepine **40** 178

^{1}H NMR of spiro[dibenzothiepine-10,2'-morpholine] **41** 179

^{13}C NMR of spiro[dibenzothiepine-10,2'-morpholine] **41** 180

^{1}H NMR of 2-methyl-6-phenylmorpholine **42** 181

^{13}C NMR of 2-methyl-6-phenylmorpholine **42** 182

COSY of 2-methyl-6-phenylmorpholine **42** 183

HSQC of 2-methyl-6-phenylmorpholine **42** 184

HMBC of 2-methyl-6-phenylmorpholine **42** 185

NOESY of 2-methyl-6-phenylmorpholine **42** 186

^{1}H NMR of 5-ethyl-2-phenylmorpholine **43** 187

^{13}C NMR of 5-ethyl-2-phenylmorpholine **43** 188

COSY of 5-ethyl-2-phenylmorpholine **43** 189

HSQC of 5-ethyl-2-phenylmorpholine **43** 190

NOESY of 5-ethyl-2-phenylmorpholine **43** 191

^{1}H NMR of 2,2-diphenyl-1,4-oxazepane **44** 192

^{13}C NMR of 2,2-diphenyl-1,4-oxazepane **44** 193

^{1}H NMR of 6,6-dimethyl-2-phenyl-1,4-oxazepane **45** 194

^{13}C NMR of 6,6-dimethyl-2-phenyl-1,4-oxazepane **45** 195

^{1}H NMR of N-phenylsulfonylpiperazine **46** 196

^{13}C NMR of N-phenylsulfonylpiperazine **46** 197

^{1}H NMR of N-Cbz-piperazine **47** 198

^{13}C NMR of N-Cbz-piperazine **47** 199

^{1}H NMR of dihydroimidazopyridinium **48** 200

^{13}C NMR of dihydroimidazopyridinium **48** 201

^{19}F NMR of dihydroimidazopyridinium **48** 202

^{1}H NMR of dihydroimidazopyridinium **49** 203

^{13}C NMR of dihydroimidazopyridinium **49** 204

^{19}F NMR of dihydroimidazopyridinium **49** 205

^{1}H NMR of dihydrooxazole **50** 206

^{13}C NMR of dihydrooxazole **50** 207

^{1}H NMR of dihydrooxazole **51** 208

^{13}C NMR of dihydrooxazole **51** 209

^{19}F NMR of dihydrooxazole **51** 210

^{1}H NMR of dihydroindeno[2,1-d]oxazole **52** 211

^{13}C NMR of dihydroindeno[2,1-d]oxazole **52** 212

^{1}H NMR of 2,2-diphenyltetrahydrobenzofuran **53** 213

^{13}C NMR of 2,2-diphenyltetrahydrobenzofuran **53** 214

^{19}F NMR of 2,2-diphenyltetrahydrobenzofuran **53** 215

^{1}H NMR of dihydroimidazopyrimidinium **54** 216

^{13}C NMR of dihydroimidazopyrimidinium **54** 217

^{19}F NMR of dihydroimidazopyrimidinium **54** 218

^{1}H NMR of dihydroimidazo[1,3,5]triazinone **55** 219

^{13}C NMR of dihydroimidazo[1,3,5]triazinone **55** 220

NOESY of dihydroimidazo[1,3,5]triazinone **55** 221

^{1}H NMR of cyclopropane ring opened product **56** 222

^{13}C NMR of cyclopropane ring opened product **56** 223

^{1}H NMR of 1,6-diene radical trap product **57** 224

^{13}C NMR of 1,6-diene radical trap product **57** 225

REFERENCES 226

MATERIALS AND METHODS

All air- and moisture-insensitive reactions were carried out under ambient atmosphere and monitored by thin-layer chromatography (TLC). High-resolution mass spectra were obtained using *Q Exactive Plus* from *Thermo*. Concentration under reduced pressure was performed by rotary evaporation at 25–40 °C at an appropriate pressure. Purified compounds were further dried under high vacuum (0.010–0.005 mBar). Yields refer to purified and spectroscopically pure compounds, unless otherwise stated.

Solvents

Dry DME, MMAc, DMF were purchased from Acros Organics. Anhydrous DCM was obtained from Phoenix Solvent Drying Systems. All deuterated solvents were purchased from Euriso-Top.

Chromatography

Thin layer chromatography (TLC) was performed using EMD TLC plates pre-coated with 250 μm thickness silica gel 60 F_{254} plates and visualized by fluorescence quenching under UV light, ninhydrin stain, and $KMnO_4$ stain. Flash column chromatography was performed using silica gel (40–63 μm particle size) purchased from Geduran®.

Photochemistry

All reactions with blue light were carried out using a photoreactor equipped with a blue LED module (*KT-Elektronik*, "*100W Power LED blau 450 nm Aquarium",* 450 nm, 100 W), consisting out of 100 LED-chips. The power of the LED was adjusted using a linear regulator. The vials were cooled with two Peltier-elements (*TEC1-12706*) while being irradiated with blue light.

Spectroscopy and Instruments

NMR spectra were recorded on a Bruker Ascend^{TM} 500 spectrometer operating at 500 MHz, 471 MHz and 126 MHz, for ^{1}H, ^{19}F and ^{13}C acquisitions, respectively; or on a Varian Unity/Inova 600 spectrometer operating at 600 MHz and 151 MHz for ^{1}H and ^{13}C acquisitions, respectively; or on a Bruker Ultrashield^{TM} 300 spectrometer operating at 300 MHz, 282 MHz and 75 MHz for ^{1}H, ^{19}F and ^{13}C acquisitions, respectively. Chemical shifts are reported in ppm with the solvent residual peak as the internal standard. For ^{1}H NMR: $CDCl_3$, δ 7.26; CD_3CN, δ 1.96; CD_2Cl_2, δ 5.32; $(CD_3)_2SO$, δ 2.50; For ^{13}C NMR: $CDCl_3$, δ 77.16; CD_3CN, δ 1.32; CD_2Cl_2, δ 53.84; $(CD_3)_2SO$, δ 39.52.[1] ^{19}F NMR spectra were referenced using a unified chemical shift scale based on the ^{1}H resonance of tetramethylsilane (1% v/v solution in the respective solvent). Data is reported as follows: s = singlet, d = doublet, t = triplet, q = quartet, m = multiplet, br = broad; coupling constants in Hz; integration.

Starting materials

All substrates were used as received from commercial suppliers, or prepared according to published procedures, respectively, unless otherwise stated. $Bi(OTf)_3$ was purchased from Alfa Aesar, and stored in an

argon-filled glovebox. Alkenes were purchased from Sigma-Aldrich, Chempur, TCI, or Alfa Aesar.

EXPERIMENTAL DATA

General procedure and reaction condition optimization for cyclization

General procedure for cyclization

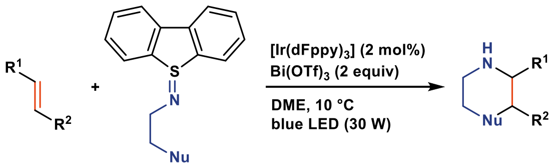

Under a nitrogen atmosphere, to a 4-mL borosilicate vial equipped with a magnetic stir bar were added alkene (if solid) (0.200 mmol, 1.00 equiv.), sulfilimine (0.400 mmol, 2.00 equiv.), [Ir(dFppy)$_3$] (3.0 mg, 4.0 μmol, 2.0 mol%), Bi(OTf)$_3$ (262 mg, 0.400 mmol, 2.00 equiv.), DME (1 mL, c = 0.2 M), and alkene (if liquid) (0.200 mmol, 1.00 equiv.). The vial was sealed with a septum-cap and irradiated for 6 h at 10 °C using a photoreactor equipped with a blue LED module (KT-Elektronik, “100W Power LED blau 450 nm Aquarium”, 450 nm, 30 W), cooled with two Peltier-elements (TEC1-12706). Then, the reaction mixture was concentrated to dryness. The residue was dissolved in DCM (5 mL) and washed with saturated aqueous sodium carbonate solution (5 mL). The aqueous phase was extracted with DCM (2 × 5 mL). The organic phase was dried over Na_2SO_4, filtered, and the solvent was removed under reduced pressure. The residue was purified by chromatography on silica gel eluting with CH_2Cl_2/MeOH (50/1–10/1 (v/v)) to afford the cyclization product.

Note: The reaction is air sensitive. Schlenk technique was used to avoid air. For simplicity, in our research, we have opted to execute the transformation for most compounds by using a glovebox. Control experiments showed that yields were within error of measurement if the reaction was carried out using a glovebox or Schlenk technique.

Table 1. Screening of acid additive [a]

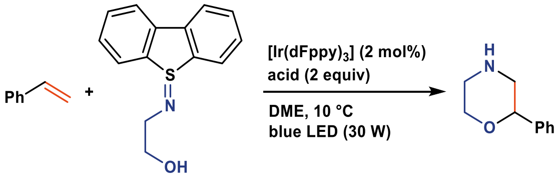

1 2

acid	Yield [%] [b]
$HBF_4·Et_2O$	85
TfOH	69
Tf_2NH	49
AcOH	0
TMSOTf	43

$Bi(OTf)_3$	83
$Cu(OTf)_2$	33
$In(OTf)_3$	29
$Fe(OTf)_3$	0
none	<5

[a] styrene (0.05 mmol), **1** (0.1 mmol, 2 equiv.), $[Ir(dFppy)_3]$ (2 mol%), acid (0.1 mmol, 2 equiv.), DME (0.1 M), blue LED (30 W), 10 °C. [b] ^1H NMR yield with CH_2Br_2 as internal standard.

Table 2. Investigation of side reactions of different acid additives [a]

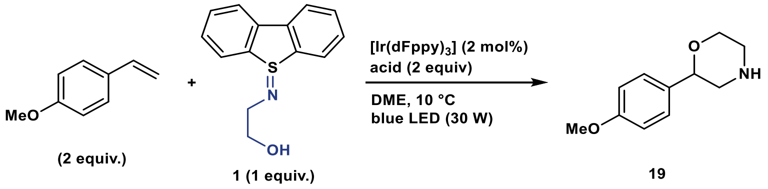

acid	yield [%] [b]	styrene remained [equiv.] [c]
$HBF_4{\cdot}Et_2O$	0	0
TfOH	0	0
$Bi(OTf)_3$	87	1
$Cu(OTf)_2$	54	1

[a] 4-methoxystyrene (0.1 mmol, 2 equiv.), **1** (0.05 mmol), $[Ir(dFppy)_3]$ (2 mol%), acid (0.1 mmol, 2 equiv.), DME (0.1 M), blue LED (30 W), 10 °C. [b] ^1H NMR yield with CH_2Br_2 as internal standard. [c] determined by ^1H NMR with CH_2Br_2 as internal standard.

Table 3. Screening of solvent [a]

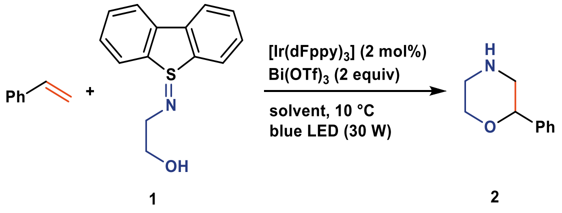

solvent	yield [%] [b]
DME	83
MMAc	70
MeCN	36

dioxane	49
DMF	0
DCM	0
HFIP	0

[a] styrene (0.05 mmol), **1** (0.1 mmol, 2 equiv.), [Ir(dFppy)₃] (2 mol%), $Bi(OTf)_3$ (0.1 mmol, 2 equiv.), solvent (0.1 M), blue LED (30 W), 10 °C. [b] ^1H NMR yield with CH_2Br_2 as internal standard. MMAc = methyl methoxyacetate.

Table 4. Screening of photocatalyst [a]

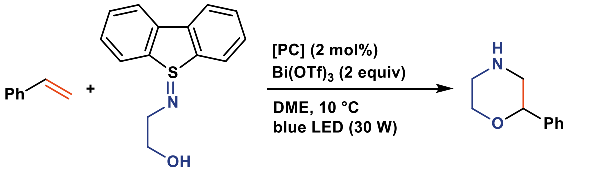

1 2

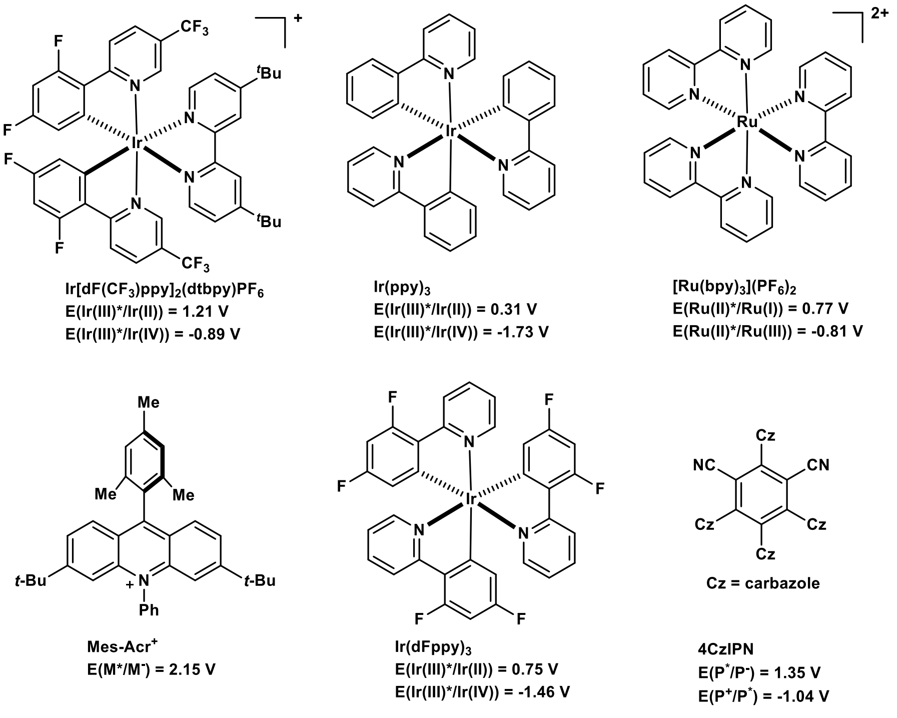

photocatalyst	yield [%] [b]
Ir[dF(CF₃)ppy]₂(dtbpy)PF₆	66
Ir(ppy)₃	63
[Ru(bpy)₃](PF₆)₂	20
Mes-Acr⁺	0

Ir(dFppy)3	83
Ir(dFppy)3	0 [c]
4CzIPN	59
none	0

[a] styrene (0.05 mmol), **1** (0.1 mmol, 2 equiv.), [PC] (2 mol%), $Bi(OTf)_3$ (0.1 mmol, 2 equiv.), DME (0.1 M), blue LED (30 W), 10 °C. [b] ^{1}H NMR yield with CH_2Br_2 as internal standard. [c] Reaction in dark.

Table 5. Screening of temperature [a]

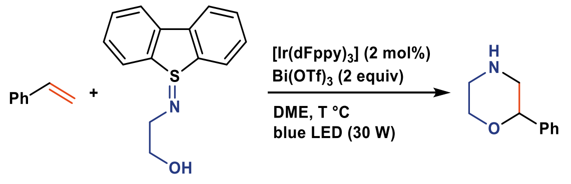

1 2

T [°C]	yield [%] [b]
0	82
10	83
10	80 [c] (75) [d]
30	69

[a] styrene (0.05 mmol), **1** (0.1 mmol, 2 equiv.), [Ir(dFppy)3] (2 mol%), $Bi(OTf)_3$ (0.1 mmol, 2 equiv.), DME (0.1 M), blue LED (30 W), T °C. [b] ^{1}H NMR yield with CH_2Br_2 as internal standard. [c] with concentration DME (0.2 M). [d] isolated yield in parenthesis.

Table 6. Screening of sulfilimine skeleton [a]

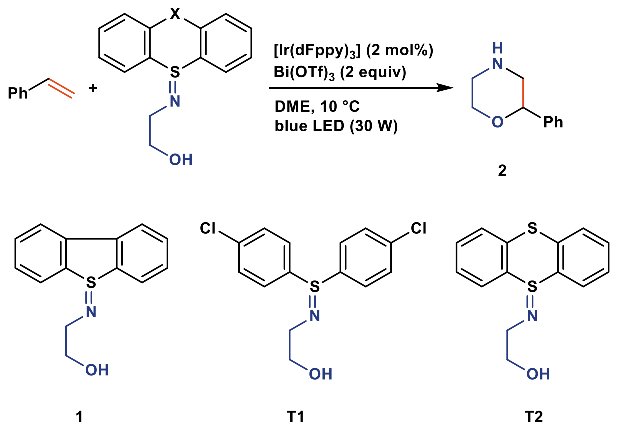

sulfilimine	yield [%] [b]

**1**	83
**T1**	58
**T2**	39

[a] styrene (0.05 mmol), sulfilimine (0.1 mmol, 2 equiv.), $[Ir(dFppy)_3]$ (2 mol%), $Bi(OTf)_3$ (0.1 mmol, 2 equiv.), DME (0.1 M), blue LED (30 W), 10 °C. [b] ^{1}H NMR yield with CH_2Br_2 as internal standard.

Substrates synthesis and cyclization reactions

Sulfilimine 1

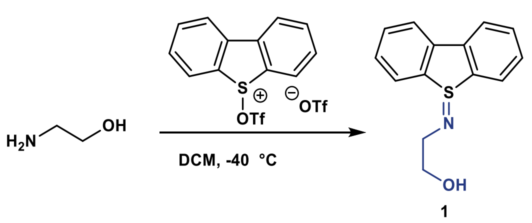

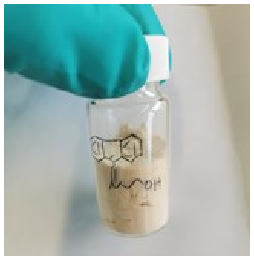

Under an argon atmosphere, to a 500 mL two-necked round-bottomed flask were added dibenzothiophene-*S*-oxide[2] (4.0 g, 20 mmol, 1.0 equiv.) and 150 mL dry DCM (c = 0.13 M). The mixture was stirred at –40 °C for 5 min followed by addition of triflic anhydride (3.4 mL, 5.9 g, 21 mmol, 1.1 equiv.). After stirring at –40 °C for 1 h, a solution of 2-aminoethan-1-ol (3.0 mL, 3.1 g, 50 mmol, 2.5 equiv.) in DCM (50 mL) was added dropwise to the reaction mixture over 10 min. Then, the reaction mixture was warmed to 25 °C. Saturated aqueous sodium carbonate solution (100 mL) was added to the reaction mixture, and the resulting mixture was stirred for 5 min. The mixture was transferred to a separatory funnel, and the organic layer was separated. The aqueous layer was extracted with DCM (2 × 200 mL). The combined organic layers were dried over Na_2SO_4, filtered, and the solvent was removed under reduced pressure. The obtained residue was purified by recrystallization from DCM (20 mL). The solid was obtained by filtration and washed with cold DCM (50 mL). The product **1** was obtained as a colorless solid (4.3 g, 85% yield).

R_f = 0.15 (DCM/MeOH = 10/1 (v/v)).

M.P. = 188 – 189 °C (recryst. solvent: DCM)

NMR Spectroscopy:

^{1}**H NMR** (500 MHz, $CDCl_3$, 23 °C, δ): 7.90 – 7.85 (m, 4H), 7.60 (td, *J* = 7.5, 1.1 Hz, 2H), 7.50 (td, *J* = 7.5, 1.1 Hz, 2H), 3.50 (t, *J* = 5.0 Hz, 2H), 2.44 (t, *J* = 5.0 Hz, 2H).

^{13}**C NMR** (126 MHz, $CDCl_3$, 23 °C, δ): 140.2, 138.1, 131.9, 129.4, 127.0, 121.9, 63.2, 50.4.

HRMS-ESI(m/z) calc'd for $C_{14}H_{14}NOS$ $[M+H]^+$, 244.0790; found, 244.0791; deviation: +0.5 ppm.

2-Phenylmorpholine 2

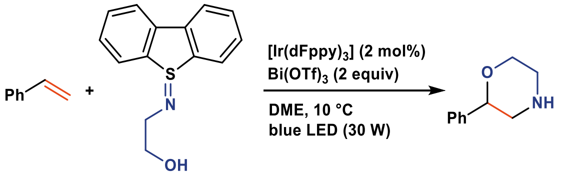

Under a nitrogen atmosphere, to a 4-mL borosilicate vial equipped with a magnetic stir bar were added sulfilimine **1** (97.2 mg, 0.400 mmol, 2.00 equiv.), [Ir(dFppy)$_3$] (3.0 mg, 4.0 µmol, 2.0 mol%), Bi(OTf)$_3$ (262 mg, 0.400 mmol, 2.00 equiv.), DME (1 mL, c = 0.2 M), and styrene (23.0 µL, 20.8 mg, 0.200 mmol, 1.00 equiv.). The vial was sealed with a septum-cap and irradiated for 6 h at 10 °C using a photoreactor equipped with a blue LED module (KT-Elektronik, "100W Power LED blau 450 nm Aquarium", 450 nm, 30 W), cooled with two Peltier-elements (TEC1-12706). Then, the reaction mixture was concentrated to dryness. The residue was dissolved in DCM (5 mL) and washed with saturated aqueous sodium carbonate solution (5 mL). The aqueous phase was extracted with DCM (2 × 5 mL). The organic phase was dried over Na_2SO_4, filtered, and the solvent was removed under reduced pressure. The residue was purified by chromatography on silica gel eluting with CH_2Cl_2/MeOH (50/1–10/1 (v/v)) to afford 24.5 mg of cyclization product **2** as a colorless oil (75% yield).

R*f* = 0.20 (DCM/MeOH = 10/1 (v/v)).

NMR Spectroscopy:

^{1}H NMR (500 MHz, $CDCl_3$, 23 ºC, δ): 7.40 – 7.27 (m, 5H), 5.25 (br, 1H), 4.70 (dd, *J* = 10.8, 2.4 Hz, 1H), 4.11 (dd, *J* = 12.5, 3.7 Hz, 1H), 3.96 (dd, *J* = 12.2, 2.5 Hz, 1H), 3.36 (dd, *J* = 12.5, 2.8 Hz, 1H), 3.25 (d, *J* = 12.6 Hz, 1H), 3.13 (td, *J* = 12.4, 3.7 Hz, 1H), 2.93 (dd, *J* = 12.7, 10.9 Hz, 1H).

^{13}C NMR (126 MHz, $CDCl_3$, 23 ºC, δ): 138.3, 128.8, 128.6, 126.2, 77.0, 65.8, 50.9, 44.5.

HRMS-EI(m/z) calc'd for $C_{10}H_{13}NO$ $[M]^+$, 163.0992; found, 163.0992; deviation: +0.0 ppm.

Sulfilimine 3

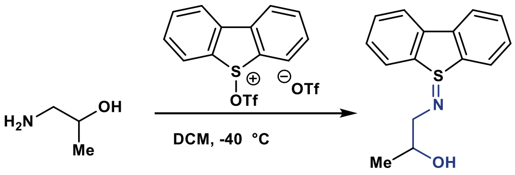

Under an argon atmosphere, to a 500 mL two-necked round-bottomed flask were added dibenzothiophene-*S*-oxide (1.0 g, 5.0 mmol, 1.0 equiv.) and 40 mL dry DCM (c = 0.13). The mixture was stirred at –40 °C for 5 min followed by addition of triflic anhydride (0.8 mL, 1.4 g, 5.0 mmol, 1.0 equiv.). After stirring at –40 °C for 1 h, a solution of 1-aminopropan-2-ol (1.0 mL, 0.97 g, 13 mmol, 2.5 equiv.) in DCM (20 mL) was added dropwise to the reaction mixture over 10 min. Then, the reaction mixture was warmed to 25 °C. Saturated

aqueous sodium carbonate solution (50 mL) was added to the reaction mixture, and the resulting mixture was stirred for 5 min. The mixture was transferred to a separatory funnel, and the organic layer was separated. The aqueous layer was extracted with DCM (2 × 50 mL). The combined organic layers were dried over Na_2SO_4, filtered, and the solvent was removed under reduced pressure. The obtained residue was purified by flash column chromatography on silica gel eluting with CH_2Cl_2/i-PrOH (50/1–10/1 (v/v)) to afford 723 mg of product **3** as a colorless solid (56% yield).

R*f* = 0.18 (DCM/MeOH = 10/1 (v/v)).

M.P. = 167 – 168 °C (recryst. solvent: DCM)

NMR Spectroscopy:

^{1}H NMR (500 MHz, $CDCl_3$, 23 ºC, δ): 7.94 – 7.84 (m, 4H), 7.62 (tt, *J* = 7.6, 1.4 Hz, 2H), 7.52 (tdd, *J* = 7.6, 3.5, 1.2 Hz, 2H), 3.77 – 3.67 (m, 1H), 3.20 (br, 1H), 2.39 (dd, *J* = 11.9, 3.3 Hz, 1H), 2.08 (dd, *J* = 11.9, 8.8 Hz, 1H), 0.94 (d, *J* = 6.2 Hz, 3H).

^{13}C NMR (126 MHz, $CDCl_3$, 23 ºC, δ): 139.9, 139.3, 138.2, 138.0, 132.1, 132.0, 129.6, 129.4, 127.2, 127.0, 122.1, 122.0, 67.6, 55.8, 20.1

HRMS-ESI(m/z) calc'd for $C_{15}H_{15}NOS$ $[M+H]^+$, 258.0948 ; found, 258.0947; deviation: +0.3 ppm.

Sulfilimine 4

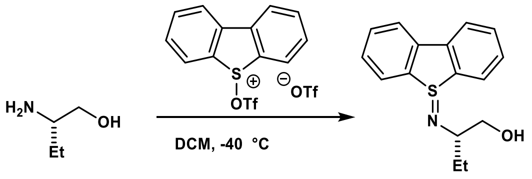

4

Under an argon atmosphere, to a 100 mL two-necked round-bottomed flask were added dibenzothiophene-*S*-oxide (1.0 g, 5.0 mmol, 1.0 equiv.) and 40 mL dry DCM (c = 0.13). The mixture was stirred at –40 °C for 5 min followed by addition of triflic anhydride (0.85 mL, 1.4 g, 5.1 mmol, 1.0 equiv.). After stirring at –40 °C for 1 h, a solution of 2-amino-1-butanol (1.2 mL, 1.2 g, 13 mmol, 2.5 equiv.) in DCM (10 mL) was added dropwise to the reaction mixture over 10 min. Then, the reaction mixture was warmed to 25 °C. Saturated aqueous sodium carbonate solution (20 mL) was added to the reaction mixture, and the resulting mixture was stirred for 5 min. The mixture was transferred to a separatory funnel, and the organic layer was separated. The aqueous layer was extracted with DCM (2 × 40 mL). The combined organic layers were dried over Na_2SO_4, filtered, and the solvent was removed under reduced pressure. The obtained residue was purified by chromatography on silica gel eluting with CH_2Cl_2/MeOH (50/1–10/1 (v/v)) to afford 0.92 g of **4** as a colorless solid (73% yield).

R*f* = 0.20 (DCM/MeOH = 10/1 (v/v)).

M.P. = 192 – 193 °C (recryst. solvent: DCM)

NMR Spectroscopy:

^{1}H NMR (500 MHz, MeOD, 23 ºC, δ): 8.05 (d, *J* = 7.8 Hz, 2H), 7.95 (t, *J* = 7.2 Hz, 2H), 7.68 (d, *J* = 7.0 Hz, 1H), 7.58 (t, *J* = 7.7 Hz, 2H), 3.15 (q, *J* = 6.9 Hz, 2H), 2.24 – 2.19 (m, 1H), 1.45 – 1.29 (m, 1H), 1.17 (dt, *J* = 13.9, 7.1 Hz, 1H), 0.74 (t, *J* = 7.7 Hz, 3H).

^{13}C NMR (126 MHz, MeOD, 23 ºC, δ): 142.2, 141.5, 139.2, 139.0, 133.5, 133.4, 130.6, 130.5, 128.4, 128.3, 123.3, 123.2, 66.9, 63.6, 27.7, 10.8.

HRMS-ESI(m/z) calc'd for $C_{16}H_{18}NOS$ $[M+H]^+$, 272.1104; found, 272.1104; deviation: –0.1 ppm.

Sulfilimine 5

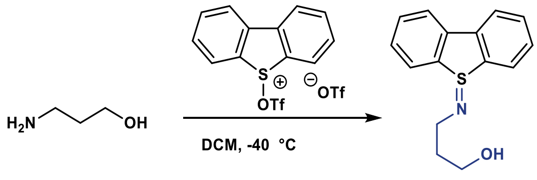

5

Under an argon atmosphere, to a 100 mL two-necked round-bottomed flask were added dibenzothiophene-*S*-oxide (1.0 g, 5.0 mmol, 1.0 equiv.) and 40 mL dry DCM (c = 0.13). The mixture was stirred at –40 °C for 5 min followed by addition of triflic anhydride (0.85 mL, 1.4 g, 5.1 mmol, 1.0 equiv.). After stirring at –40 °C for 1 h, a solution of 3-aminopropan-1-ol (0.95 mL, 0.94 g, 13 mmol, 2.5 equiv.) in DCM (10 mL) was added dropwise to the reaction mixture over 10 min. Then, the reaction mixture was warmed to 25 °C. Saturated aqueous sodium carbonate solution (20 mL) was added to the reaction mixture, and the resulting mixture was stirred for 5 min. The mixture was transferred to a separatory funnel, and the organic layer was separated. The aqueous layer was extracted with DCM (2 × 40 mL). The combined organic layers were dried over Na_2SO_4, filtered, and the solvent was removed under reduced pressure. The obtained residue was purified by chromatography on silica gel eluting with CH_2Cl_2/MeOH (50/1–10/1 (v/v)) to afford 0.94 g of **5** as a colorless solid (73% yield).

R_f = 0.20 (DCM/MeOH = 10/1 (v/v)).

M.P. = 123 – 124 °C (recryst. solvent: DCM)

NMR Spectroscopy:

^{1}H NMR (500 MHz, CD_2Cl_2, 23 ºC, δ): 7.91 (d, *J* = 7.6 Hz, 2H), 7.88 (d, *J* = 7.6 Hz, 2H), 7.62 (td, *J* = 7.5, 1.2 Hz, 2H), 7.53 (td, *J* = 7.6, 1.2 Hz, 2H), 3.87 (br, 1H), 3.65 – 3.58 (m, 2H), 2.46 (t, *J* = 5.7 Hz, 2H), 1.56 – 1.48 (m, 2H).

^{13}C NMR (126 MHz, CD_2Cl_2, 23 ºC, δ): 140.2, 138.5, 132.0, 129.5, 127.1, 122.2, 64.6, 49.1, 33.3.

HRMS-ESI(m/z) calc'd for $C_{15}H_{16}NOS$ $[M+H]^+$, 258.0948; found, 258.0947; deviation: –0.5 ppm.

Sulfilimine 6

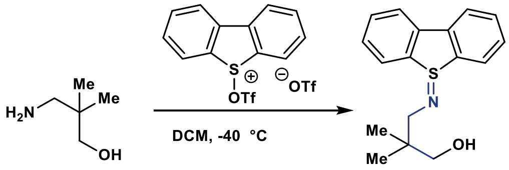

6

Under an argon atmosphere, to a 500 mL two-necked round-bottomed flask were added dibenzothiophene-*S*-oxide (0.50 g, 2.5 mmol, 1.0 equiv.) and 20 mL dry DCM (c = 0.13). The mixture was stirred at –40 °C for 5 min followed by addition of triflic anhydride (0.40 mL, 0.70 g, 2.5 mmol, 1.0 equiv.). After stirring at –40 °C for 1 h, a solution of 3-amino-2,2-dimethylpropan-1-ol (0.60 g, 6.2 mmol, 2.5 equiv.) in DCM (10 mL) was added dropwise to the reaction mixture over 10 min. Then, the reaction mixture was warmed to 25 °C. Saturated aqueous sodium carbonate solution (50 mL) was added to the reaction mixture, and the resulting mixture was stirred for 5 min. The mixture was transferred to a separatory funnel, and the organic layer was separated. The aqueous layer was extracted with DCM (2 × 50 mL). The combined organic layers were dried over Na_2SO_4, filtered, and the solvent was removed under reduced pressure. The obtained residue was purified by flash column chromatography on silica gel eluting with CH_2Cl_2/i-PrOH (50/1–10/1 (v/v)) to afford 346 mg product **6** as a colorless oil (50% yield).

R*f* = 0.30 (DCM/MeOH = 10/1 (v/v)).

NMR Spectroscopy:

^{1}H NMR (500 MHz, $CDCl_3$, 23 ºC, δ): 8.28 (d, *J* = 7.7 Hz, 2H), 8.02 (dd, *J* = 7.7, 1.1 Hz, 2H), 7.86 (td, *J* = 7.6, 1.1 Hz, 2H), 7.72 (td, *J* = 7.7, 1.1 Hz, 2H), 7.15 (br, 1H), 3.36 (s, 2H), 2.24 (d, *J* = 6.0 Hz, 2H), 0.72 (s, 6H).

^{13}C NMR (126 MHz, $CDCl_3$, 23 ºC, δ): 139.0, 135.4, 131.6, 129.6, 129.2, 123.4, 68.6, 50.6, 36.0, 22.6

HRMS-ESI(m/z) calc'd for $C_{17}H_{19}NOS$ $[M+H]^+$, 286.1261; found, 286.1260; deviation: +0.3 ppm.

Sulfilimine 7

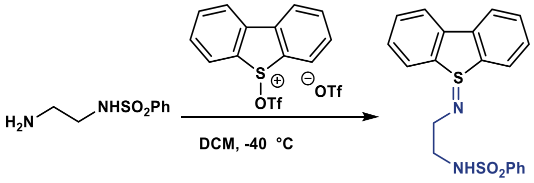

7

Under an argon atmosphere, to a 100 mL two-necked round-bottomed flask were added dibenzothiophene-*S*-oxide (1.0 g, 5.0 mmol, 1.0 equiv.) and 40 mL dry DCM (c = 0.13). The mixture was stirred at –40 °C for 5 min followed by addition of triflic anhydride (0.85 mL, 1.4 g, 5.1 mmol, 1.0 equiv.). After stirring at –40 °C for 1 h, a solution of N-(2-aminoethyl)benzenesulfonamide (2.5 g, 13 mmol, 2.5 equiv.) in DCM (10 mL) was added dropwise to the reaction mixture over 10 min. Then, the reaction mixture was warmed to 25 °C. Saturated

aqueous sodium carbonate solution (20 mL) was added to the reaction mixture, and the resulting mixture was stirred for 5 min. The mixture was transferred to a separatory funnel, and the organic layer was separated. The aqueous layer was extracted with DCM (2 × 40 mL). The combined organic layers were dried over Na_2SO_4, filtered, and the solvent was removed under reduced pressure. The obtained residue was purified by chromatography on silica gel eluting with CH_2Cl_2/MeOH (50/1–20/1 (v/v)) to afford 1.5 g of **7** as a colorless solid (78% yield).

R*f* = 0.20 (DCM/MeOH = 10/1 (v/v)).

M.P. = 140 – 141 °C (recryst. solvent: DCM)

NMR Spectroscopy:

^{1}H NMR (500 MHz, $CDCl_3$, 23 ºC, δ): 7.88 – 7.78 (m, 6H), 7.60 (t, *J* = 7.5 Hz, 2H), 7.55 – 7.41 (m, 5H), 2.86 (t, *J* = 5.5 Hz, 2H), 2.38 (t, *J* = 5.5 Hz, 2H).

^{13}C NMR (126 MHz, $CDCl_3$, 23 ºC, δ): 140.2, 139.0, 138.1, 132.4, 132.2, 129.5, 129.0, 127.2, 127.0, 122.0, 47.1, 44.4.

HRMS-ESI(m/z) calc'd for $C_{20}H_{19}N_2O_2S_2$ $[M+H]^+$, 383.0883; found, 383.0882; deviation: –0.3 ppm.

Sulfilimine 8

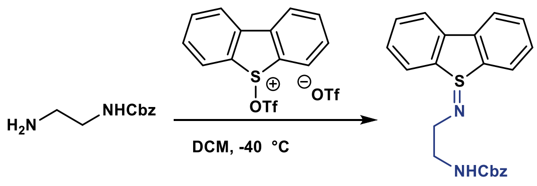

8

Under an argon atmosphere, to a 100 mL two-necked round-bottomed flask were added dibenzothiophene-*S*-oxide (1.0 g, 5.0 mmol, 1.0 equiv.) and 40 mL dry DCM (c = 0.13). The mixture was stirred at –40 °C for 5 min followed by addition of triflic anhydride (0.85 mL, 1.4 g, 5.1 mmol, 1.0 equiv.). After stirring at –40 °C for 1 h, a solution of benzyl (2-aminoethyl)carbamate (2.5 g, 13 mmol, 2.5 equiv.) in DCM (10 mL) was added dropwise to the reaction mixture over 10 min. Then, the reaction mixture was warmed to 25 °C. Saturated aqueous sodium carbonate solution (20 mL) was added to the reaction mixture, and the resulting mixture was stirred for 5 min. The mixture was transferred to a separatory funnel, and the organic layer was separated. The aqueous layer was extracted with DCM (2 × 40 mL). The combined organic layers were dried over Na_2SO_4, filtered, and the solvent was removed under reduced pressure. The obtained residue was purified by chromatography on silica gel eluting with CH_2Cl_2/MeOH (50/1–20/1 (v/v)) to afford 1.1 g of **8** as a colorless solid (60% yield).

R*f* = 0.20 (DCM/MeOH = 10/1 (v/v)).

M.P. = 150 – 151 °C (recryst. solvent: DCM)

NMR Spectroscopy:

^{1}H NMR (500 MHz, $CDCl_3$, 23 ºC, δ): 7.92 – 7.81 (m, 4H), 7.59 (t, *J* = 7.7 Hz, 2H), 7.50 – 7.42 (m, 2H), 7.38 – 7.25 (m, 4H), 5.51 (br, 1H), 5.04 (s, 2H), 3.14 (t, *J* = 5.7 Hz, 2H), 2.40 (t, *J* = 5.7 Hz, 2H).

^{13}C NMR (126 MHz, $CDCl_3$, 23 ºC, δ): 156.5, 138.1, 137.0, 132.1, 129.5, 128.6, 128.0, 127.0, 126.8, 122.0, 121.7, 66.4, 47.6, 42.5.

HRMS-ESI(m/z) calc'd for $C_{22}H_{21}N_2O_2S$ $[M+H]^+$, 377.1318; found, 377.1318; deviation: +0.0 ppm.

Sulfilimine 9

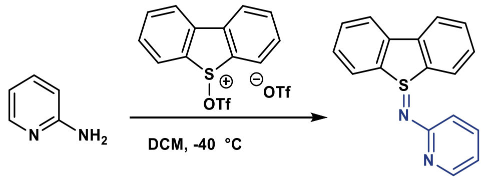

9

Under an argon atmosphere, to a 100 mL two-necked round-bottomed flask were added dibenzothiophene-*S*-oxide (1.0 g, 5.0 mmol, 1.0 equiv.) and 40 mL dry DCM (c = 0.13). The mixture was stirred at –40 °C for 5 min followed by addition of triflic anhydride (0.85 mL, 1.4 g, 5.1 mmol, 1.0 equiv.). After stirring at –40 °C for 1 h, a solution of 2-aminopyridine (1.2 g, 13 mmol, 2.5 equiv.) in DCM (10 mL) was added dropwise to the reaction mixture over 10 min. Then, the reaction mixture was warmed to 25 °C. Saturated aqueous sodium carbonate solution (20 mL) was added to the reaction mixture, and the resulting mixture was stirred for 5 min. The mixture was transferred to a separatory funnel, and the organic layer was separated. The aqueous layer was extracted with DCM (2 × 40 mL). The combined organic layers were dried over Na_2SO_4, filtered, and the solvent was removed under reduced pressure. The obtained residue was purified by trituration with Et_2O (3 × 10 mL) to afford 786 mg of **9** as a yellow solid (57% yield).

R_f = 0.40 (DCM/MeOH = 10/1 (v/v)).

M.P. = 114 – 115 °C (recryst. solvents: DCM/Et_2O)

NMR Spectroscopy:

^{1}H NMR (500 MHz, $CDCl_3$, 23 ºC, δ): 8.11 (d, *J* = 7.2 Hz, 2H), 8.06 – 8.01 (m, 1H), 7.86 (d, *J* = 7.8 Hz, 2H), 7.56 (td, *J* = 7.6, 1.2 Hz, 2H), 7.44 (td, *J* = 7.6, 1.2 Hz, 2H), 7.35 (ddd, *J* = 8.5, 6.9, 1.9 Hz, 1H), 6.84 (dt, *J* = 8.4, 1.1 Hz, 1H), 6.57 (ddd, *J* = 7.0, 5.1, 1.1 Hz, 1H).

^{13}C NMR (126 MHz, $CDCl_3$, 23 ºC, δ): 164.9, 146.9, 140.9, 137.8, 136.8, 131.7, 129.5, 127.9, 122.0, 115.2, 112.8.

HRMS-EI(m/z) calc'd for $C_{17}H_{12}N_2S$ $[M]^+$, 276.0718; found, 276.0716; deviation: –0.7 ppm.

Sulfilimine 10

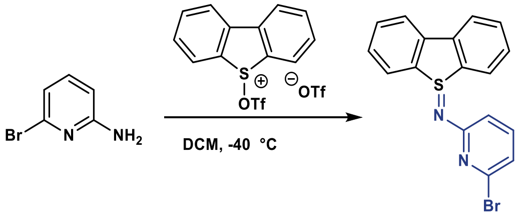

10

Under an argon atmosphere, to a 100 mL two-necked round-bottomed flask were added dibenzothiophene-*S*-oxide (1.0 g, 5.0 mmol, 1.0 equiv.) and 40 mL dry DCM (c = 0.13). The mixture was stirred at –40 °C for 5 min followed by addition of triflic anhydride (0.85 mL, 1.4 g, 5.1 mmol, 1.0 equiv.). After stirring at –40 °C for 1 h, a solution of 6-bromo-2-aminopyridine (2.2 g, 13 mmol, 2.5 equiv.) in DCM (10 mL) was added dropwise to the reaction mixture over 10 min. Then, the reaction mixture was warmed to 25 °C. Saturated aqueous sodium carbonate solution (20 mL) was added to the reaction mixture, and the resulting mixture was stirred for 5 min. The mixture was transferred to a separatory funnel, and the organic layer was separated. The aqueous layer was extracted with DCM (2 × 40 mL). The combined organic layers were dried over Na_2SO_4, filtered, and the solvent was removed under reduced pressure. The obtained residue was purified by trituration with MeCN (3 × 5 mL) to afford 956 mg of **10** as a yellow solid (54% yield).

R*f* = 0.40 (DCM/MeOH = 10/1 (v/v)).

M.P. = 154 – 155 °C (recryst. solvents: MeCN)

NMR Spectroscopy:

^{1}H NMR (500 MHz, $CDCl_3$, 23 ºC, δ): 8.09 (d, *J* = 7.6 Hz, 2H), 7.91 (dd, *J* = 7.8, 1.1 Hz, 2H), 7.61 (td, *J* = 7.6, 1.2 Hz, 2H), 7.48 (td, *J* = 7.6, 1.2 Hz, 2H), 7.15 (dd, *J* = 8.5, 7.0 Hz, 1H), 6.73 (d, *J* = 8.2 Hz, 1H), 6.67 (d, *J* = 7.3 Hz, 1H).

^{13}C NMR (126 MHz, $CDCl_3$, 23 ºC, δ): 164.9, 139.7, 138.8, 138.6, 138.5, 132.0, 129.6, 127.8, 122.1, 115.6, 113.6.

HRMS-ESI(m/z) calc'd for $C_{17}H_{12}N_2SBr$ $[M+1]^+$, 354.9898; found, 354.9899; deviation: +0.3 ppm.

Dibenzothiophen-5-imine 11

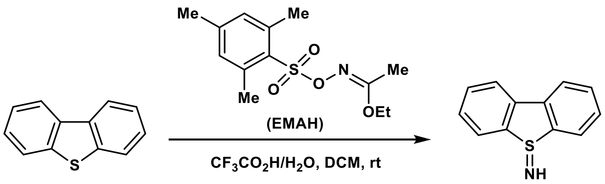

11

Dibenzothiophene (5.5 g, 30 mmol) was dissolved in 70 mL of CH_2Cl_2, and into this solution, 5.7 mL (75 mmol, 2.5 equiv.) of trifluoroacetic acid and 1.1 mL (60 mmol, 2.0 equiv.) of H_2O were added. To this reaction mixture was added EMAH (11 g, 39 mmol, 1.3 equiv) in CH_2Cl_2 (50 mL). After stirring at 25 °C for 12 h, the

reaction mixture was basified with saturated aqueous sodium carbonate solution (200 mL). The resulting mixture was transferred to a separatory funnel, and the organic layer was separated. The aqueous layer was extracted with chloroform (3 × 70 mL). The organic layers were washed with brine (100 mL), dried over Na_2SO_4, filtered, and the solvent was removed under reduced pressure. The residue was purified by chromatography on silica gel eluting with CH_2Cl_2/MeOH (20/1–5/1 (v/v)) to afford 3.7 g of **11** as a colorless solid (63 % yield).

R*f* = 0.10 (DCM/MeOH = 8/1 (v/v)).

M.P. = 180 – 181 °C (recryst. solvent: DCM)

NMR Spectroscopy:

^{1}**H NMR** (500 MHz, CD_2Cl_2, 23 ºC, δ): 7.90 (dt, *J* = 7.6, 0.8 Hz, 2H), 7.86 (dt, *J* = 7.8, 1.0 Hz, 2H), 7.59 (td, *J* = 7.6, 1.3 Hz, 2H), 7.51 (td, *J* = 7.6, 1.2 Hz, 2H), 2.19 (s, 1H).

^{13}**C NMR** (126 MHz, CD_2Cl_2, 23 ºC, δ): 146.7, 136.2, 131.9, 129.8, 126.6, 122.2.

HRMS-ESI(m/z) calc'd for $C_{12}H_{10}NS$ $[M+H]^+$, 200.0530; found, 200.0528; deviation: –0.8 ppm.

Sulfilimine 12

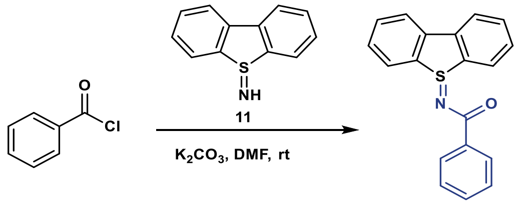

12

Under an argon atmosphere, to a 100 mL two-necked round-bottomed flask were added dibenzothiophene-*S*-imine[3] **11** (199 mg, 1.00 mmol, 1.00 equiv.), K_2CO_3 (276 mg, 2.00 mmol, 2.00 equiv.), and 10 mL dry DMF (c = 0.10 M). The mixture was stirred at 0 °C for 5 min followed by addition of benzoyl chloride (174 µL, 211 mg, 1.50 mmol, 1.50 equiv.). Then, the reaction mixture was warmed to 25 °C and stirred for 3 h. After complete consumption of dibenzothiophene-S-imine as monitored by TLC, water (30 mL) and EtOAc (30 mL) was added to the reaction mixture. The resulting mixture was transferred to a separatory funnel, and the organic layer was separated. The aqueous layer was extracted with EtOAc (2 × 30 mL). The combined organic layers were washed with brine (3 × 20 mL), dried over Na_2SO_4, filtered, and the solvent was removed under reduced pressure. The obtained residue was purified by chromatography on silica gel eluting with CH_2Cl_2/MeOH (100/1–50/1 (v/v)) to afford 242 mg of **12** as a colorless solid (80% yield).

R*f* = 0.30 (DCM/MeOH = 20/1 (v/v)).

M.P. = 170 – 171 °C (recryst. solvent: Et_2O)

NMR Spectroscopy:

^{1}**H NMR** (500 MHz, $CDCl_3$, 23 ºC, δ): 8.25 (d, *J* = 7.8 Hz, 2H), 8.12 (dd, *J* = 7.8, 1.8 Hz, 2H), 7.94 (d, *J* =

7.8 Hz, 2H), 7.68 (td, *J* = 7.6, 1.1 Hz, 2H), 7.56 (td, *J* = 7.6, 1.2 Hz, 2H), 7.43 (t, *J* = 7.3 Hz, 1H), 7.36 (t, *J* = 7.3 Hz, 2H).

^{13}C NMR (126 MHz, $CDCl_3$, 23 ºC, δ): 178.6, 138.5, 138.2, 135.9, 132.8, 131.2, 130.0, 129.1, 129.0, 128.0, 122.4.

HRMS-ESI(m/z) calc'd for $C_{19}H_{14}NOS$ $[M+H]^+$, 304.0789; found, 304.0790; deviation: +0.6 ppm.

Sulfilimine 13

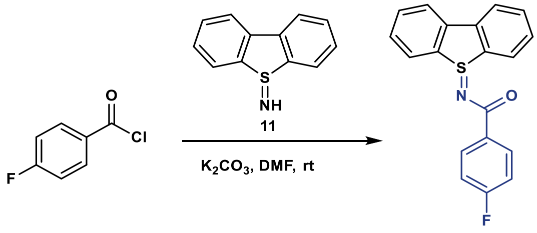

13

Under an argon atmosphere, to a 100 mL two-necked round-bottomed flask were added dibenzothiophene-*S*-imine **11** (199 mg, 1.00 mmol, 1.00 equiv.), K_2CO_3 (276 mg, 2.00 mmol, 2.00 equiv.), and 10 mL dry DMF (c = 0.10 M). The mixture was stirred at 0 °C for 5 min followed by addition of 4-fluorobenzoyl chloride (177 μL, 238 mg, 1.50 mmol, 1.50 equiv.). Then, the reaction mixture was warmed to 25 °C and stirred for 3 h. After complete consumption of dibenzothiophene-S-imine as monitored by TLC, water (30 mL) and EtOAc (30 mL) was added to the reaction mixture. The resulting mixture was transferred to a separatory funnel, and the organic layer was separated. The aqueous layer was extracted with EtOAc (2 × 30 mL). The combined organic layers were washed with brine (3 × 20 mL), dried over Na_2SO_4, filtered, and the solvent was removed under reduced pressure. The obtained residue was purified by chromatography on silica gel eluting with CH_2Cl_2/MeOH (100/1–50/1 (v/v)) to afford 244 mg of **13** as a colorless solid (76% yield).

R*f* = 0.35 (DCM/MeOH = 20/1 (v/v)).

M.P. = 169 – 170 °C (recryst. solvent: Et_2O)

NMR Spectroscopy:

^{1}H NMR (500 MHz, $CDCl_3$, 23 ºC, δ): 8.22 (d, *J* = 7.8 Hz, 2H), 8.12 (dd, *J* = 8.7, 5.7 Hz, 2H), 7.93 (dd, *J* = 7.8, 1.0 Hz, 2H), 7.67 (td, *J* = 7.6, 1.1 Hz, 2H), 7.55 (td, *J* = 7.7, 1.2 Hz, 2H), 7.01 (t, *J* = 8.7 Hz, 2H).

^{13}C NMR (126 MHz, $CDCl_3$, 23 ºC, δ): 177.6, 164.9 (d, *J* = 250.3 Hz), 138.5, 138.2, 132.6, 132.2 (d, *J* = 3.0 Hz), 131.4 (d, *J* = 8.9 Hz), 130.0, 128.9, 122.5, 114.8 (d, *J* = 21.9 Hz).

^{19}F NMR (471 MHz, $CDCl_3$, 23 ºC, δ): –109.62.

HRMS-ESI(m/z) calc'd for $C_{19}H_{13}NOSF$ $[M+H]^+$, 322.0695; found, 322.0696; deviation: +0.4 ppm.

Sulfilimine 14

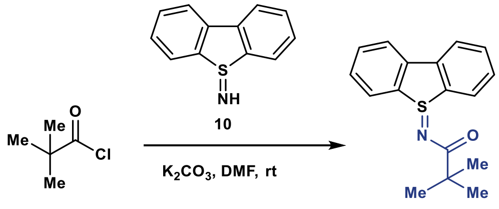

14

Under an argon atmosphere, to a 100 mL two-necked round-bottomed flask were added dibenzothiophene-*S*-imine **11** (199 mg, 1.00 mmol, 1.00 equiv.), K_2CO_3 (276 mg, 2.00 mmol, 2.00 equiv.), and 10 mL dry DMF (c = 0.10 M). The mixture was stirred at 0 °C for 5 min followed by addition of pivaloyl chloride (181 µL, 185 mg, 1.50 mmol, 1.50 equiv.). Then, the reaction mixture was warmed to 25 °C and stirred for 3 h. After complete consumption of dibenzothiophene-S-imine as monitored by TLC, water (30 mL) and EtOAc (30 mL) was added to the reaction mixture. The resulting mixture was transferred to a separatory funnel, and the organic layer was separated. The aqueous layer was extracted with EtOAc (2 × 30 mL). The combined organic layers were washed with brine (3 × 20 mL), dried over Na_2SO_4, filtered, and the solvent was removed under reduced pressure. The obtained residue was purified by chromatography on silica gel eluting with CH_2Cl_2/MeOH (100/1–50/1 (v/v)) to afford 269 mg of **14** as a colorless solid (95% yield).

R*f* = 0.40 (DCM/MeOH = 20/1 (v/v)).

M.P. = 143 – 144 °C (recryst. solvent: Et_2O)

NMR Spectroscopy:

^{1}H NMR (500 MHz, $CDCl_3$, 23 ºC, δ): 8.12 (d, *J* = 7.8 Hz, 1H), 7.89 (d, *J* = 7.6 Hz, 1H), 7.62 (td, *J* = 7.6, 1.2 Hz, 2H), 7.51 (td, *J* = 7.6, 1.2 Hz, 2H), 1.24 (s, 9H).

^{13}C NMR (126 MHz, $CDCl_3$, 23 ºC, δ): 192.2, 138.6, 138.3, 132.2, 129.8, 128.6, 122.3, 40.2, 28.8.

HRMS-ESI(m/z) calc'd for $C_{17}H_{18}NOS$ $[M+H]^+$, 284.1105; found, 284.1104; deviation: –0.6 ppm.

Sulfilimine 15

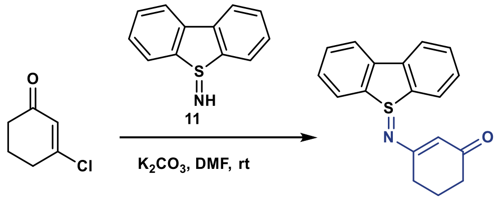

15

Under an argon atmosphere, to a 100 mL two-necked round-bottomed flask were added dibenzothiophene-*S*-imine **11** (199 mg, 1.00 mmol, 1.00 equiv.), K_2CO_3 (276 mg, 2.00 mmol, 2.00 equiv.), and 10 mL dry DMF (c = 0.10 M). The mixture was stirred at 0 °C for 5 min followed by addition of 3-chlorocyclohex-2-en-1-one (195 mg, 1.50 mmol, 1.50 equiv.). Then, the reaction mixture was warmed to 25 °C and stirred for 3 h. After complete consumption of dibenzothiophene-S-imine as monitored by TLC, water (30 mL) and EtOAc (30 mL)

was added to the reaction mixture. The resulting mixture was transferred to a separatory funnel, and the organic layer was separated. The aqueous layer was extracted with EtOAc (2 × 30 mL). The combined organic layers were washed with brine (3 × 20 mL), dried over Na_2SO_4, filtered, and the solvent was removed under reduced pressure. The obtained residue was purified by trituration with Et_2O (3 × 10 mL) to afford 179 mg of **15** as a colorless solid (61% yield).

R*f* = 0.50 (DCM/MeOH = 20/1 (v/v)).

M.P. = 119 – 120 °C (recryst. solvents: DCM/Et_2O)

NMR Spectroscopy:

^{1}**H NMR** (500 MHz, $CDCl_3$, 23 ºC, δ): 7.96 – 7.90 (m, 4H), 7.67 (t, *J* = 7.6 Hz, 2H), 7.54 (t, *J* = 7.6 Hz, 2H), 5.62 (s, 1H), 2.47 (t, *J* = 6.2 Hz, 2H), 2.31 (t, *J* = 6.5 Hz, 2H), 1.96 (p, *J* = 6.4 Hz, 2H).

^{13}**C NMR** (126 MHz, $CDCl_3$, 23 ºC, δ): 198.0, 177.6, 138.0, 137.8, 132.9, 130.3, 127.6, 122.9, 102.6, 36.8, 33.9, 22.7.

HRMS-ESI(m/z) calc'd for $C_{18}H_{16}NOS$ $[M+H]^+$, 294.0948; found, 294.0947; deviation: –0.3 ppm.

Sulfilimine 16

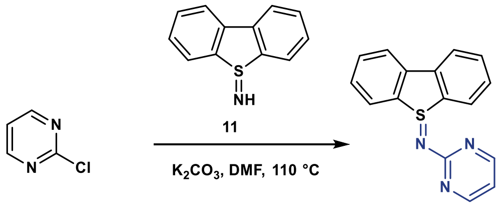

16

Under an argon atmosphere, to a 100 mL two-necked round-bottomed flask were added dibenzothiophene-*S*-imine **11** (199 mg, 1.00 mmol, 1.00 equiv.), K_2CO_3 (276 mg, 2.00 mmol, 2.00 equiv.), 2-chloropyrimidine (229 mg, 2.00 mmol, 2.00 equiv.), and 10 mL dry DMF (c = 0.10 M). Then, the reaction mixture was heated at 110 °C for 6 h. After complete consumption of dibenzothiophene-S-imine as monitored by TLC, water (30 mL) and EtOAc (30 mL) was added to the reaction mixture. The resulting mixture was transferred to a separatory funnel, and the organic layer was separated. The aqueous layer was extracted with EtOAc (2 × 30 mL). The combined organic layers were washed with brine (3 × 20 mL), dried over Na_2SO_4, filtered, and the solvent was removed under reduced pressure. The obtained residue was purified by trituration with Et_2O (3 × 10 mL) to afford 177 mg of **16** as a colorless solid (64% yield).

R*f* = 0.20 (DCM/MeOH = 20/1 (v/v)).

M.P. = 189 – 190 °C (recryst. solvents: DCM/Et_2O)

NMR Spectroscopy:

^{1}**H NMR** (500 MHz, $CDCl_3$, 23 ºC, δ): 8.29 (d, *J* = 4.9 Hz, 2H), 8.11 (d, *J* = 7.8 Hz, 2H), 7.85 (d, *J* = 7.5 Hz, 2H), 7.57 (td, *J* = 7.6, 1.1 Hz, 2H), 7.44 (td, *J* = 7.6, 1.1 Hz, 2H), 6.54 (t, *J* = 4.8 Hz, 1H).

^{13}C NMR (126 MHz, $CDCl_3$, 23 ºC, δ): 169.3, 157.9, 139.8, 138.0, 132.1, 129.5, 128.2, 122.1, 110.6.

HRMS-ESI(m/z) calc'd for $C_{16}H_{12}N_3S$ $[M+H]^+$, 278.0744; found, 278.0746; deviation: +0.7 ppm.

Sulfilimine 17

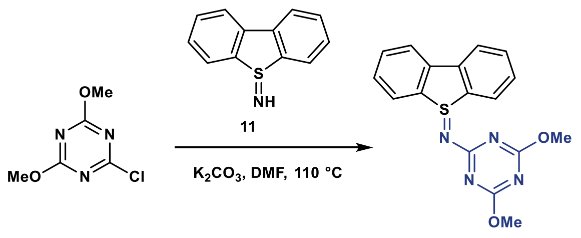

Under an argon atmosphere, to a 100 mL two-necked round-bottomed flask were added dibenzothiophene-*S*-imine **11** (199 mg, 1.00 mmol, 1.00 equiv.), K_2CO_3 (276 mg, 2.00 mmol, 2.00 equiv.), 2-chloro-4,6-dimethoxy-1,3,5-triazine (351 mg, 2.00 mmol, 2.00 equiv.), and 10 mL dry DMF (c = 0.10 M). Then, the reaction mixture was heated at 110 °C for 6 h. After complete consumption of dibenzothiophene-S-imine as monitored by TLC, water (30 mL) and EtOAc (30 mL) was added to the reaction mixture. The resulting mixture was transferred to a separatory funnel, and the organic layer was separated. The aqueous layer was extracted with EtOAc (2 × 30 mL). The combined organic layers were washed with brine (3 × 20 mL), dried over Na_2SO_4, filtered, and the solvent was removed under reduced pressure. The obtained residue was purified by trituration with Et_2O (3 ×10 mL) to afford 169 mg of **17** as a colorless solid (50% yield).

R*f* = 0.25 (DCM/MeOH = 20/1 (v/v)).

M.P. = 195 – 196 °C (recryst. solvents: DCM/Et_2O)

NMR Spectroscopy:

^{1}H NMR (500 MHz, $CDCl_3$, 23 ºC, δ): 8.16 (d, *J* = 7.6 Hz, 2H), 7.89 (d, *J* = 7.2 Hz, 2H), 7.64 (t, *J* = 7.6 Hz, 2H), 7.50 (td, *J* = 7.6, 1.1 Hz, 2H), 3.94 (s, 6H).

^{13}C NMR (126 MHz, $CDCl_3$, 23 ºC, δ): 175.9, 172.2, 138.5, 138.3, 132.8, 130.0, 128.8, 122.4, 54.7.

HRMS-EI(m/z) calc'd for $C_{17}H_{14}N_2O_2S$ $[M]^+$, 338.0837; found, 338.0832; deviation: –1.4 ppm.

N-Boc-2-phenylmorpholine 18

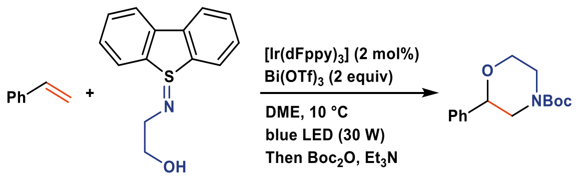

Under a nitrogen atmosphere, to a 4-mL borosilicate vial equipped with a magnetic stir bar were added sulfilimine **1** (97.2 mg, 0.400 mmol, 2.00 equiv.), $[Ir(dFppy)_3]$ (3.0 mg, 4.0 µmol, 2.0 mol%), $Bi(OTf)_3$ (262 mg, 0.400 mmol, 2.00 equiv.), DME (1 mL, c = 0.2 M), and styrene (23 µL, 21 mg, 0.20 mmol, 1.0 equiv.). The vial

was sealed with a septum-cap and irradiated for 6 h at 10 °C using a photoreactor equipped with a blue LED module (KT-Elektronik, "100W Power LED blau 450 nm Aquarium", 450 nm, 30 W), cooled with two Peltier-elements (TEC1-12706). Then, Et_3N (166 µL, 121 mg, 1.20 mmol, 6.00 equiv.) was added, followed by the addition of Boc_2O (131 mg, 0.600 mmol, 3.00 equiv.). The reaction mixture was stirred for 12 h and concentrated. The residue was dissolved in DCM (5 mL) and washed with water (5 mL). The aqueous phase was extracted with DCM (2 × 5 mL). The organic phase was dried over Na_2SO_4, filtered, and the solvent was removed under reduced pressure. The residue was purified by chromatography on silica gel eluting with pentane/EtOAc (20/1–10/1 (v/v)) to afford 34.7 mg of cyclization product **18** as a colorless oil (66% yield).

R_f = 0.20 (pentane/EtOAc = 10/1 (v/v)).

NMR Spectroscopy:

^{1}H NMR (300 MHz, $CDCl_3$, 23 ºC, δ): 7.41 – 7.33 (m, 4H), 7.33 – 7.28 (m, 1H), 4.41 (d, *J* = 9.7 Hz, 1H), 4.24 – 3.86 (m, 3H), 3.68 (td, *J* = 11.9, 3.0 Hz, 1H), 3.13 – 2.97 (m, 1H), 2.93 – 2.74 (m, 1H), 1.48 (s, 9H).

^{13}C NMR (151 MHz, $CDCl_3$, 23 ºC, δ): 154.8, 139.5, 128.6, 128.2, 126.3, 80.3, 78.0, 66.9, 49.6, 43.1, 28.6.

HRMS-ESI(m/z) calc'd for $C_{15}H_{21}NO_3Na$ $[M+Na]^+$, 286.1415; found, 286.1414; deviation: –0.4 ppm.

2-(4-Methoxyphenyl)morpholine 19

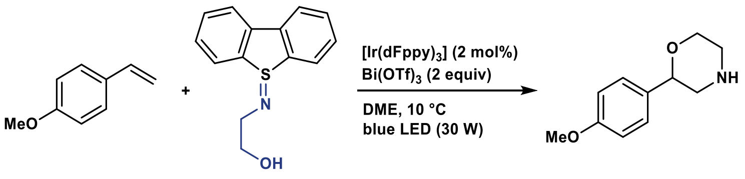

1 19

Under a nitrogen atmosphere, to a 4-mL borosilicate vial equipped with a magnetic stir bar were added sulfilimine **1** (97.2 mg, 0.400 mmol, 2.00 equiv.), $[Ir(dFppy)_3]$ (3.0 mg, 4.0 µmol, 2.0 mol%), $Bi(OTf)_3$ (262 mg, 0.400 mmol, 2.00 equiv.), DME (1 mL, c = 0.2 M), and 4-methoxystyrene (27 µL, 27 mg, 0.20 mmol, 1.0 equiv.). The vial was sealed with a septum-cap and irradiated for 6 h at 10 °C using a photoreactor equipped with a blue LED module (KT-Elektronik, "100W Power LED blau 450 nm Aquarium", 450 nm, 30 W), cooled with two Peltier-elements (TEC1-12706). Then, the reaction mixture was concentrated to dryness. The residue was dissolved in DCM (5 mL) and washed with saturated aqueous sodium carbonate solution (5 mL). The aqueous phase was extracted with DCM (2 × 5 mL). The organic phase was dried over Na_2SO_4, filtered, and the solvent was removed under reduced pressure. The residue was purified by chromatography on silica gel eluting with CH_2Cl_2/MeOH (50/1–10/1 (v/v)) to afford 30.1 mg of cyclization product **19** as a colorless oil (78% yield).

R_f = 0.20 (DCM/MeOH = 10/1 (v/v)).

NMR Spectroscopy:

^{1}H NMR (500 MHz, $CDCl_3$, 23 ºC, δ): 7.22 (d, *J* = 8.7 Hz, 2H), 6.82 (d, *J* = 8.8 Hz, 2H), 4.37 (dd, *J* = 10.3, 2.5 Hz, 1H), 3.96 (dd, *J* = 11.2, 3.3 Hz, 1H), 3.74 (s, 3H), 3.70 (dd, *J* = 11.5, 2.7 Hz, 1H), 3.00 – 2.89 (m, 2H), 2.86 – 2.79 (m, 1H), 2.74 (dd, *J* = 12.4, 10.3 Hz, 1H), 2.27 (br, 1H).

^{13}C NMR (126 MHz, $CDCl_3$, 23 ºC, δ): 159.3, 132.8, 127.5, 113.9, 79.0, 68.5, 55.4, 53.2, 45.7.

HRMS-ESI(m/z) calc'd for $C_{11}H_{16}NO_2$ $[M+H]^+$, 194.1174; found, 194.1176; deviation: +0.6 ppm.

2-(4-Fluorophenyl)morpholine 20

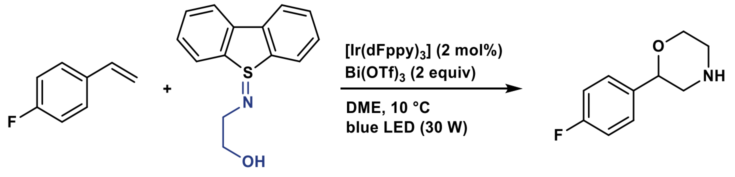

1 20

Under a nitrogen atmosphere, to a 4-mL borosilicate vial equipped with a magnetic stir bar were added sulfilimine **1** (97.2 mg, 0.400 mmol, 2.00 equiv.), [Ir(dFppy)$_3$] (3.0 mg, 4.0 µmol, 2.0 mol%), $Bi(OTf)_3$ (262 mg, 0.400 mmol, 2.00 equiv.), DME (1 mL, c = 0.2 M), and 4-fluorostyrene (24 µL, 24 mg, 0.20 mmol, 1.0 equiv.). The vial was sealed with a septum-cap and irradiated for 6 h at 10 °C using a photoreactor equipped with a blue LED module (KT-Elektronik, "100W Power LED blau 450 nm Aquarium", 450 nm, 30 W), cooled with two Peltier-elements (TEC1-12706). Then, the reaction mixture was concentrated to dryness. The residue was dissolved in DCM (5 mL) and washed with saturated aqueous sodium carbonate solution (5 mL). The aqueous phase was extracted with DCM (2 × 5 mL). The organic phase was dried over Na_2SO_4, filtered, and the solvent was removed under reduced pressure. The residue was purified by chromatography on silica gel eluting with CH_2Cl_2/MeOH (50/1–10/1 (v/v)) to afford 26.0 mg of cyclization product **20** as a colorless oil (72% yield).

R*f* = 0.22 (DCM/MeOH = 10/1 (v/v)).

NMR Spectroscopy:

^{1}H NMR (500 MHz, $CDCl_3$, 23 ºC, δ): 7.32 (dd, *J* = 8.3, 5.4 Hz, 2H), 7.02 (dd, *J* = 8.3 Hz, 2H), 4.49 (dd, *J* = 10.5, 2.5 Hz, 1H), 4.03 (dd, *J* = 11.6, 3.3 Hz, 1H), 3.84 – 3.74 (m, 1H), 3.05 (dd, *J* = 12.5, 2.5 Hz, 1H), 2.99 (td, *J* = 11.9, 3.3 Hz, 1H), 2.92 (dd, *J* = 12.4, 1.7 Hz, 1H), 2.89 (br, 1H), 2.76 (dd, *J* = 12.3, 10.5 Hz, 1H).

^{13}C NMR (126 MHz, $CDCl_3$, 23 ºC, δ): 162.4 (d, *J* = 245.6 Hz), 136.2 (d, *J* = 3.0 Hz), 127.9 (d, *J* = 8.2 Hz), 115.4 (d, *J* = 21.1 Hz), 78.5, 68.2, 53.1, 45.5.

^{19}F NMR (471 MHz, $CDCl_3$, 23 ºC, δ): –114.62.

HRMS-EI(m/z) calc'd for $C_{10}H_{12}NOF$ $[M]^+$, 181.0900; found, 181.0897; deviation: –1.5 ppm.

2,2-Diphenylmorpholine 21

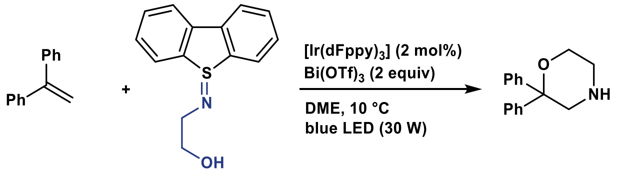

Under a nitrogen atmosphere, to a 4-mL borosilicate vial equipped with a magnetic stir bar were added sulfilimine **1** (97.2 mg, 0.400 mmol, 2.00 equiv.), [Ir(dFppy)$_3$] (3.0 mg, 4.0 µmol, 2.0 mol%), Bi(OTf)$_3$ (262 mg, 0.400 mmol, 2.00 equiv.), DME (1 mL, c = 0.2 M), and 1,1-diphenylethylene (35 µL, 36 mg, 0.20 mmol, 1.0 equiv.). The vial was sealed with a septum-cap and irradiated for 6 h at 10 °C using a photoreactor equipped with a blue LED module (KT-Elektronik, "100W Power LED blau 450 nm Aquarium", 450 nm, 30 W), cooled with two Peltier-elements (TEC1-12706). Then, the reaction mixture was concentrated to dryness. The residue was dissolved in DCM (5 mL) and washed with saturated aqueous sodium carbonate solution (5 mL). The aqueous phase was extracted with DCM (2 × 5 mL). The organic phase was dried over Na_2SO_4, filtered, and the solvent was removed under reduced pressure. The residue was purified by chromatography on silica gel eluting with CH_2Cl_2/MeOH (50/1–10/1 (v/v)) to afford 40.0 mg of cyclization product **21** as a colorless oil (84% yield).

R_f = 0.35 (DCM/MeOH = 10/1 (v/v)).

NMR Spectroscopy:

^{1}H NMR (500 MHz, $CDCl_3$, 23 ºC, δ): 7.44 (dd, *J* = 8.4, 1.4 Hz, 4H), 7.37 (dd, *J* = 8.6, 6.9 Hz, 4H), 7.32 – 7.24 (m, 2H), 3.77 (dd, *J* = 5.9, 4.0 Hz, 2H), 3.53 (s, 2H), 3.42 (br, 1H), 3.02 – 2.97 (m, 2H).

^{13}C NMR (126 MHz, $CDCl_3$, 23 ºC, δ): 143.6, 128.6, 127.3, 126.7, 78.8, 62.1, 53.3, 45.6.

HRMS-ESI(m/z) calc'd for $C_{16}H_{18}NO$ $[M+H]^+$, 240.1384; found, 240.1383; deviation: –0.4 ppm.

2-(Naphthalen-2-yl)morpholine 22

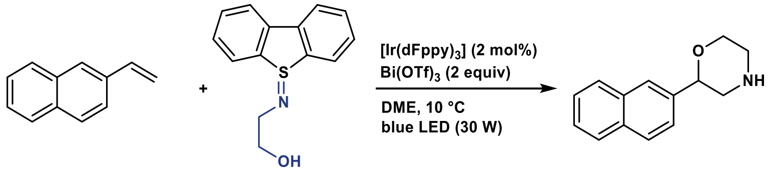

Under a nitrogen atmosphere, to a 4-mL borosilicate vial equipped with a magnetic stir bar were added sulfilimine **1** (97.2 mg, 0.400 mmol, 2.00 equiv.), [Ir(dFppy)$_3$] (3.0 mg, 4.0 µmol, 2.0 mol%), Bi(OTf)$_3$ (262 mg, 0.400 mmol, 2.00 equiv.), and 2-vinylnaphthalene (30.8 mg, 0.200 mmol, 1.00 equiv.), DME (1 mL, c = 0.2 M). The vial was sealed with a septum-cap and irradiated for 6 h at 10 °C using a photoreactor equipped with a blue LED module (KT-Elektronik, "100W Power LED blau 450 nm Aquarium", 450 nm, 30 W), cooled with two Peltier-elements (TEC1-12706). Then, the reaction mixture was concentrated to dryness. The residue

was dissolved in DCM (5 mL) and washed with saturated aqueous sodium carbonate solution (5 mL). The aqueous phase was extracted with DCM (2 × 5 mL). The organic phase was dried over Na_2SO_4, filtered, and the solvent was removed under reduced pressure. The residue was purified by chromatography on silica gel eluting with CH_2Cl_2/MeOH (50/1–10/1 (v/v)) to afford 23.4 mg of cyclization product **22** as a colorless oil (55% yield).

R*f* = 0.25 (DCM/MeOH = 10/1 (v/v)).

NMR Spectroscopy:

^{1}H NMR (500 MHz, $CDCl_3$, 23 ºC, δ): 7.92 – 7.78 (m, 4H), 7.52 – 7.42 (m, 3H), 4.77 (dd, *J* = 10.5, 2.5 Hz, 1H), 4.13 (dd, *J* = 12.4, 3.7 Hz, 1H), 3.99 – 3.91 (m, 1H), 3.26 (dd, *J* = 12.7, 2.6 Hz, 1H), 3.14 – 2.98 (m, 2H), 2.93 (dd, *J* = 12.5, 10.5 Hz, 1H), 2.72 (br, 1H).

^{13}C NMR (126 MHz, $CDCl_3$, 23 ºC, δ): 136.7, 133.4, 133.3, 128.5, 128.2, 127.8, 126.4, 126.3, 125.2, 124.0, 78.1, 67.0, 51.6, 44.7.

HRMS-ESI(m/z) calc'd for $C_{14}H_{16}NO$ $[M+H]^+$, 214.1228; found, 214.1226; deviation: –0.9 ppm.

2-Methyl-2-phenylmorpholine 23

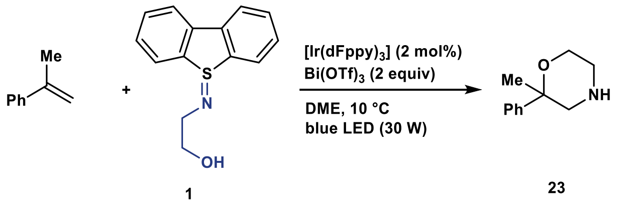

Under a nitrogen atmosphere, to a 4-mL borosilicate vial equipped with a magnetic stir bar were added sulfilimine **1** (97.2 mg, 0.400 mmol, 2.00 equiv.), $[Ir(dFppy)_3]$ (3.0 mg, 4.0 µmol, 2.0 mol%), $Bi(OTf)_3$ (262 mg, 0.400 mmol, 2.00 equiv.), DME (1 mL, c = 0.2 M), and 2-phenyl-1-propene (26 µL, 24 mg, 0.20 mmol, 1.0 equiv.). The vial was sealed with a septum-cap and irradiated for 6 h at 10 °C using a photoreactor equipped with a blue LED module (KT-Elektronik, "100W Power LED blau 450 nm Aquarium", 450 nm, 30 W), cooled with two Peltier-elements (TEC1-12706). Then, the reaction mixture was concentrated to dryness. The residue was dissolved in DCM (5 mL) and washed with saturated aqueous sodium carbonate solution (5 mL). The aqueous phase was extracted with DCM (2 × 5 mL). The organic phase was dried over Na_2SO_4, filtered, and the solvent was removed under reduced pressure. The residue was purified by chromatography on silica gel eluting with CH_2Cl_2/MeOH (50/1–10/1 (v/v)) to afford 24.8 mg of cyclization product **23** as a colorless oil (70% yield).

R*f* = 0.30 (DCM/MeOH = 10/1 (v/v)).

NMR Spectroscopy:

^{1}H NMR (500 MHz, $CDCl_3$, 23 ºC, δ): 7.47 – 7.30 (m, 4H), 7.33 – 7.21 (m, 1H), 3.73 (d, *J* = 11.7 Hz, 1H), 3.70 – 3.59 (m, 1H), 3.49 (d, *J* = 13.3 Hz, 1H), 3.06 – 2.96 (m, 2H), 2.84 – 2.78 (m, 1H), 2.53 (br, 1H), 1.42 (s, 3H).

^{13}C NMR (126 MHz, $CDCl_3$, 23 °C, δ): 143.8, 128.9, 127.2, 125.9, 74.8, 62.0, 53.2, 45.5, 28.8.

HRMS-EI(m/z) calc'd for $C_{11}H_{15}NO$ $[M]^+$, 117.1148; found, 117.1148; deviation: –0.1 ppm.

Indole-3-morpholine 24

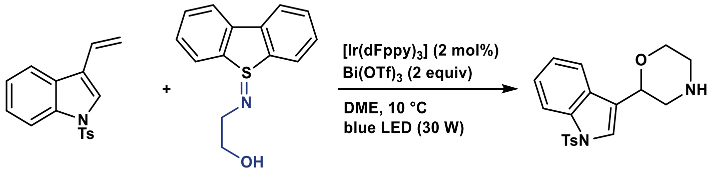

1 24

Under a nitrogen atmosphere, to a 4-mL borosilicate vial equipped with a magnetic stir bar were added sulfilimine **1** (97.2 mg, 0.400 mmol, 2.00 equiv.), $[Ir(dFppy)_3]$ (3.0 mg, 4.0 μmol, 2.0 mol%), $Bi(OTf)_3$ (262 mg, 0.400 mmol, 2.00 equiv.), DME (1 mL, c = 0.2 M), and 3-vinyl indole (59.4 mg, 0.200 mmol, 1.00 equiv.). The vial was sealed with a septum-cap and irradiated for 6 h at 10 °C using a photoreactor equipped with a blue LED module (KT-Elektronik, "100W Power LED blau 450 nm Aquarium", 450 nm, 30 W), cooled with two Peltier-elements (TEC1-12706). Then, the reaction mixture was concentrated to dryness. The residue was dissolved in DCM (5 mL) and washed with saturated aqueous sodium carbonate solution (5 mL). The aqueous phase was extracted with DCM (2 × 5 mL). The organic phase was dried over Na_2SO_4, filtered, and the solvent was removed under reduced pressure. The residue was purified by chromatography on silica gel eluting with CH_2Cl_2/MeOH (50/1–20/1 (v/v)) to afford 42.7 mg of cyclization product **24** as a colorless oil (60% yield).

R_f = 0.20 (DCM/MeOH = 10/1 (v/v)).

NMR Spectroscopy:

^{1}H NMR (500 MHz, $CDCl_3$, 23 °C, δ): 7.96 (dt, *J* = 8.2, 0.9 Hz, 1H), 7.76 (d, *J* = 8.4 Hz, 2H), 7.62 (dt, *J* = 7.8, 1.0 Hz, 1H), 7.54 (d, *J* = 0.9 Hz, 1H), 7.30 (ddd, *J* = 8.4, 7.2, 1.2 Hz, 1H), 7.26 – 7.18 (m, 3H), 4.80 (dd, *J* = 10.2, 2.9 Hz, 1H), 4.09 – 4.00 (m, 1H), 3.85 (td, *J* = 11.4, 2.9 Hz, 1H), 3.22 (dd, *J* = 12.2, 2.5 Hz, 1H), 3.09 – 2.95 (m, 3H), 2.46 (br, 1H), 2.34 (s, 3H).

^{13}C NMR (151 MHz, CD_2Cl_2, 23 °C, δ): 145.8, 135.5, 135.5, 130.3, 129.6, 127.1, 125.1, 123.5, 123.4, 122.9, 120.9, 113.9, 73.3, 68.7, 52.0, 46.3, 21.7.

HRMS-ESI(m/z) calc'd for $C_{19}H_{21}N_2O_3S$ $[M+1]^+$, 357.1265; found, 357.1267; deviation: +0.8 ppm.

2-(6-Methoxypyridin-3-yl)morpholine 25

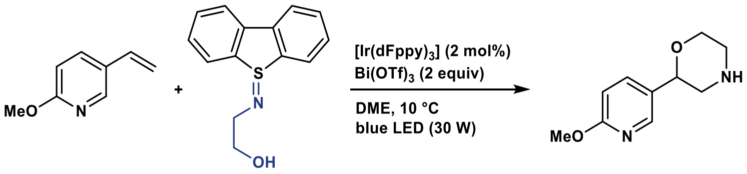

1 25

Under a nitrogen atmosphere, to a 4-mL borosilicate vial equipped with a magnetic stir bar were added sulfilimine **1** (97.2 mg, 0.400 mmol, 2.00 equiv.), [Ir(dFppy)$_3$] (3.0 mg, 4.0 µmol, 2.0 mol%), Bi(OTf)$_3$ (262 mg, 0.400 mmol, 2.00 equiv.), DME (1 mL, c = 0.2 M), and 2-methoxy-5-vinylpyridine[4] (27.0 mg, 0.200 mmol, 1.00 equiv.). The vial was sealed with a septum-cap and irradiated for 6 h at 10 °C using a photoreactor equipped with a blue LED module (KT-Elektronik, "100W Power LED blau 450 nm Aquarium", 450 nm, 30 W), cooled with two Peltier-elements (TEC1-12706). Then, the reaction mixture was concentrated to dryness. The residue was dissolved in DCM (5 mL) and washed with saturated aqueous sodium carbonate solution (5 mL). The aqueous phase was extracted with DCM (2 × 5 mL). The organic phase was dried over Na_2SO_4, filtered, and the solvent was removed under reduced pressure. The residue was purified by chromatography on silica gel eluting with CH_2Cl_2/MeOH (50/1–10/1 (v/v)) to afford 20.0 mg of cyclization product **25** as a colorless oil (51% yield).

R*f* = 0.20 (DCM/MeOH = 10/1 (v/v)).

NMR Spectroscopy:

^{1}H NMR (500 MHz, $CDCl_3$, 23 ºC, δ): 8.12 (d, *J* = 2.5 Hz, 1H), 7.58 (dd, *J* = 8.6, 2.5 Hz, 1H), 6.77 – 6.69 (m, 1H), 4.55 (dd, *J* = 10.6, 2.5 Hz, 1H), 4.04 (d, *J* = 11.5 Hz, 1H), 3.92 (s, 3H), 3.90 – 3.82 (m, 1H), 3.76 (br, 1H), 3.11 (dd, *J* = 12.5, 2.5 Hz, 1H), 3.08 – 2.98 (m, 2H), 2.84 (dd, *J* = 12.5, 10.6 Hz, 1H).

^{13}C NMR (126 MHz, $CDCl_3$, 23 ºC, δ): 164.3, 145.1, 136.9, 127.9, 111.0, 75.8, 67.2, 53.7, 51.6, 44.8.

HRMS-EI(m/z) calc'd for $C_{10}H_{14}N_2O_2$ $[M]^+$, 194.1052; found, 194.1050; deviation: –0.9 ppm.

2,3-Diphenylmorpholine 26

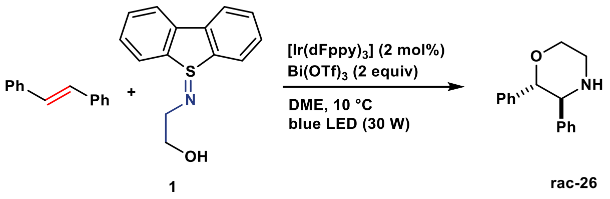

Under a nitrogen atmosphere, to a 4-mL borosilicate vial equipped with a magnetic stir bar were added sulfilimine **1** (97.3 mg, 0.400 mmol, 2.00 equiv.), [Ir(dFppy)$_3$] (3.0 mg, 4.0 µmol, 2.0 mol%), Bi(OTf)$_3$ (262.4 mg, 0.400 mmol, 2.00 equiv.), DME (1 mL, c = 0.2 M), and trans-Stilbene (36.0 mg, 0.200 mmol, 1.00 equiv.). The vial was sealed with a septum-cap and irradiated for 6 h at 10 °C using a photoreactor equipped with a blue LED module (KT-Elektronik, "100W Power LED blau 450 nm Aquarium", 450 nm, 30 W), cooled with two Peltier-elements (TEC1-12706). Then, the reaction mixture was concentrated to dryness. The residue was dissolved in DCM (5 mL) and washed with saturated aqueous sodium carbonate solution (5 mL). The aqueous phase was extracted with DCM (2 × 5 mL). The organic phase was dried over Na_2SO_4, filtered, and the solvent was removed under reduced pressure. The residue was purified by chromatography on silica gel eluting with CH_2Cl_2/MeOH (100/1–10/1 (v/v)) to afford 41.5 mg of cyclization product **26** as white solid (87% yield, >20:1 dr).

R_f = 0.40 (DCM/MeOH = 10/1 (v/v)).

NMR Spectroscopy:

^{1}H NMR (500 MHz, CD_3CN, 23 ºC, δ): 7.19 – 7.08 (m, 8H), 7.08 – 7.01 (m, 2H), 4.35 (d, *J* = 9.0 Hz, 1H), 3.97 (ddd, *J* = 11.1, 3.4, 1.3 Hz, 1H), 3.83 (dd, *J* = 11.5, 2.6 Hz, 1H), 3.78 (d, *J* = 9.0 Hz, 1H), 3.14 (td, *J* = 11.8, 3.4 Hz, 1H), 2.97 (ddd, *J* = 12.0, 2.7, 1.3 Hz, 1H).

^{13}C NMR (126 MHz, CD_3CN, 23 ºC, δ): 142.0, 140.9, 129.2, 128.8, 128.7, 128.6, 128.4, 128.2, 85.8, 68.6, 67.8, 47.2.

HRMS-ESI(m/z) calc'd for $C_{16}H_{17}NO$ $[M+H]^+$, 240.1384; found, 240.1383; deviation: +0.6 ppm.

3-Methyl-2-phenylmorpholine 27

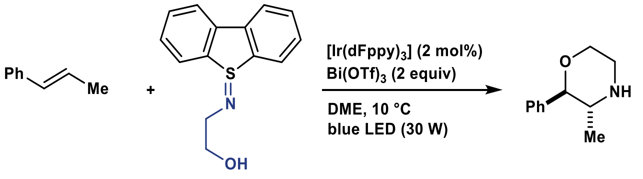

1 rac-27

Under a nitrogen atmosphere, to a 4-mL borosilicate vial equipped with a magnetic stir bar were added sulfilimine **1** (97.2 mg, 0.400 mmol, 2.00 equiv.), [Ir(dFppy)$_3$] (3.0 mg, 4.0 µmol, 2.0 mol%), $Bi(OTf)_3$ (262 mg, 0.400 mmol, 2.00 equiv.), DME (1 mL, c = 0.2 M), and (E)-prop-1-en-1-ylbenzene (23.6 mg, 0.200 mmol, 1.00 equiv.). The vial was sealed with a septum-cap and irradiated for 6 h at 10 °C using a photoreactor equipped with a blue LED module (KT-Elektronik, "100W Power LED blau 450 nm Aquarium", 450 nm, 30 W), cooled with two Peltier-elements (TEC1-12706). Then, the reaction mixture was concentrated to dryness. The residue was dissolved in DCM (5 mL) and washed with saturated aqueous sodium carbonate solution (5 mL). The aqueous phase was extracted with DCM (2 × 5 mL). The organic phase was dried over Na_2SO_4, filtered, and the solvent was removed under reduced pressure. The residue was purified by chromatography on silica gel eluting with CH_2Cl_2/MeOH (50/1–10/1 (v/v)). The product-containing fractions were collected and concentrated under reduced pressure to afford a mixture of diastereoisomers as a colorless oil. The diastereoselectivity was determined by appropriate integration of the peaks of methyl protons at 0.9 and 0.8 ppm, respectively, in the ^{1}H NMR spectrum (13:1 dr). The mixture was further purified by chromatography on silica gel eluting with CH_2Cl_2/MeOH (50/1–10/1 (v/v)) to afford 25.0 mg of product **27** as a colorless oil (71% yield).

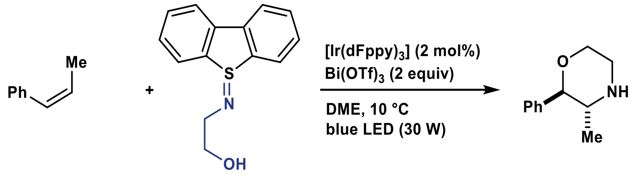

1 rac-27

Under a nitrogen atmosphere, to a 4-mL borosilicate vial equipped with a magnetic stir bar were added

sulfilimine **1** (97.2 mg, 0.400 mmol, 2.00 equiv.), [Ir(dFppy)$_3$] (3.0 mg, 4.0 µmol, 2.0 mol%), Bi(OTf)$_3$ (262 mg, 0.400 mmol, 2.00 equiv.), DME (1 mL, c = 0.2 M), and (Z)-prop-1-en-1-ylbenzene (23.6 mg, 0.200 mmol, 1.00 equiv.). The vial was sealed with a septum-cap and irradiated for 6 h at 10 °C using a photoreactor equipped with a blue LED module (KT-Elektronik, “100W Power LED blau 450 nm Aquarium”, 450 nm, 30 W), cooled with two Peltier-elements (TEC1-12706). Then, the reaction mixture was concentrated to dryness. The residue was dissolved in DCM (5 mL) and washed with saturated aqueous sodium carbonate solution (5 mL). The aqueous phase was extracted with DCM (2 × 5 mL). The organic phase was dried over Na_2SO_4, filtered, and the solvent was removed under reduced pressure. The product-containing fractions were collected and concentrated under reduced pressure to afford a mixture of diastereoisomers as a colorless oil. The diastereoselectivity was determined by appropriate integration of the peaks of methyl protons at 0.9 and 0.8 ppm in the ^{1}H NMR spectra (13:1 dr). The mixture was further purified by chromatography on silica gel eluting with CH_2Cl_2/MeOH (50/1–10/1 (v/v)) to afford 27.6 mg of product **27** as a colorless oil (78% yield, 13:1 dr).

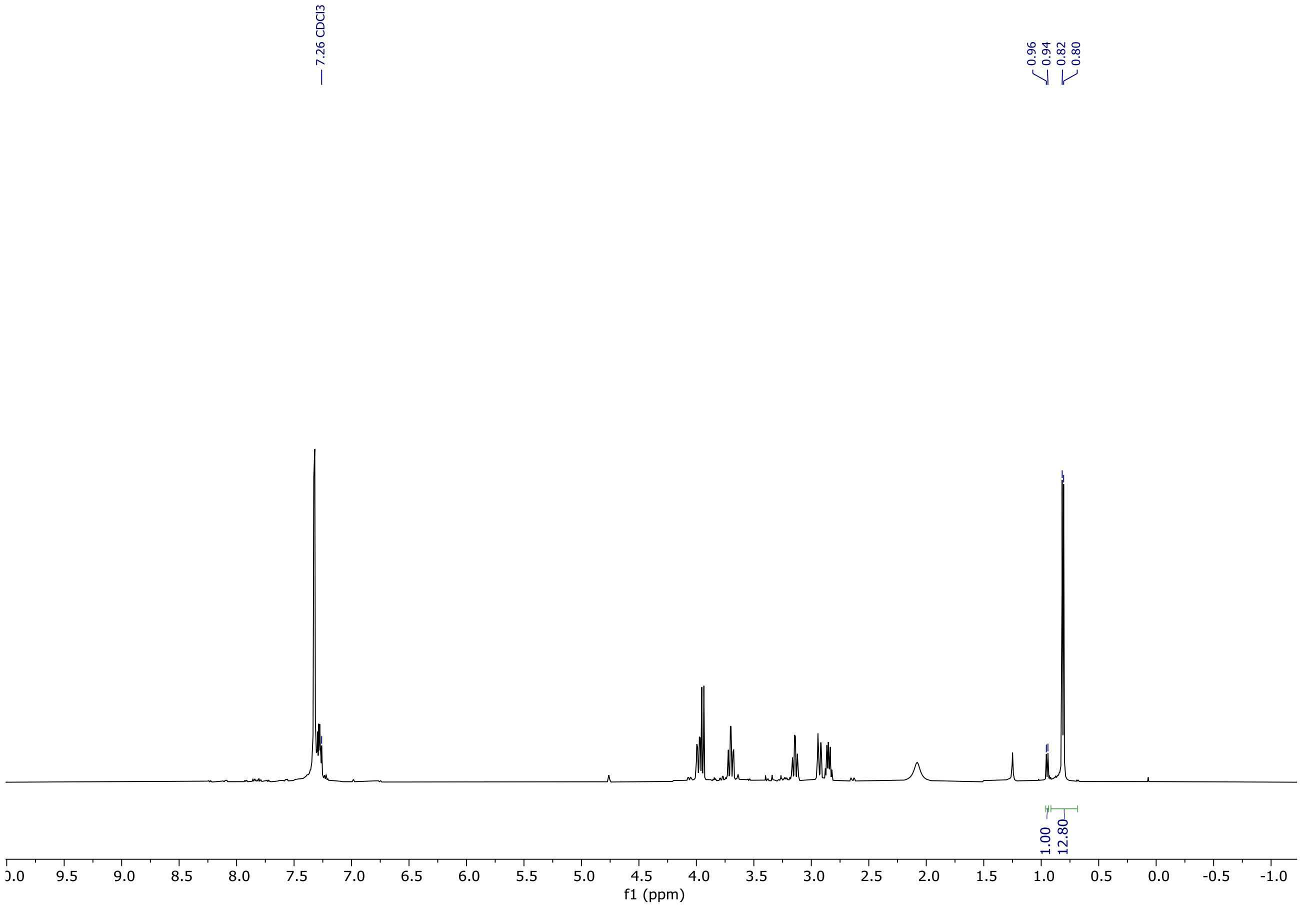

R*f* = 0.25 (DCM/MeOH = 10/1 (v/v)).

NMR Spectroscopy:

^{1}H NMR (500 MHz, $CDCl_3$, 23 ºC, δ): 7.39 – 7.27 (m, 5H), 4.00 (ddd, *J* = 11.4, 3.5, 1.3 Hz, 1H), 3.97 (d, *J* = 9.0 Hz, 1H), 3.73 (td, *J* = 11.6, 2.6 Hz, 1H), 3.17 (td, *J* = 12.0, 3.4 Hz, 1H), 2.96 (dd, *J* = 12.4, 1.4 Hz, 1H), 2.93 – 2.83 (m, 1H), 0.84 (d, *J* = 6.4 Hz, 3H).

^{13}C NMR (126 MHz, $CDCl_3$, 23 ºC, δ): 139.9, 128.5, 128.3, 127.6, 86.4, 68.2, 56.3, 46.6, 18.4.

HRMS-EI(m/z) calc'd for $C_{11}H_{15}NO$ $[M]^+$, 177.1147; found,177.1148; deviation: +0.4 ppm.

2-(Benzothiophen-3-yl)morpholine 28

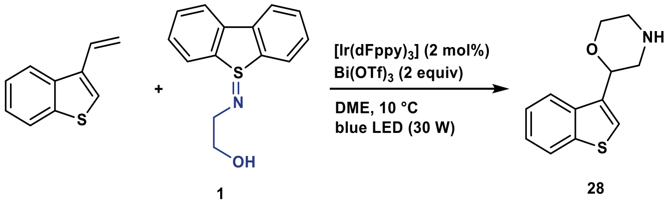

Under a nitrogen atmosphere, to a 4-mL borosilicate vial equipped with a magnetic stir bar were added sulfilimine **1** (97.2 mg, 0.400 mmol, 2.00 equiv.), $[Ir(dFppy)_3]$ (3.0 mg, 4.0 µmol, 2.0 mol%), $Bi(OTf)_3$ (262 mg, 0.400 mmol, 2.00 equiv.), DME (1 mL, c = 0.2 M), and 3-vinylbenzo[b]thiophene (32.0 mg, 0.200 mmol, 1.00 equiv.). The vial was sealed with a septum-cap and irradiated for 6 h at 10 °C using a photoreactor equipped with a blue LED module (KT-Elektronik, "100W Power LED blau 450 nm Aquarium", 450 nm, 30 W), cooled with two Peltier-elements (TEC1-12706). Then, the reaction mixture was concentrated to dryness. The residue was dissolved in DCM (5 mL) and washed with saturated aqueous sodium carbonate solution (5 mL). The aqueous phase was extracted with DCM (2 × 5 mL). The organic phase was dried over Na_2SO_4, filtered, and the solvent was removed under reduced pressure. The residue was purified by chromatography on silica gel eluting with CH_2Cl_2/MeOH (50/1–10/1 (v/v)) to afford 29.8 mg of cyclization product **28** as a colorless oil (68% yield).

R_f = 0.20 (DCM/MeOH = 10/1 (v/v)).

NMR Spectroscopy:

^{1}H NMR (500 MHz, $CDCl_3$, 23 ºC, δ): 7.93 – 7.80 (m, 2H), 7.42 (d, J = 0.9 Hz, 1H), 7.42 – 7.31 (m, 2H), 4.98 (dd, J = 10.3, 2.5 Hz, 1H), 4.09 (d, J = 12.0 Hz, 1H), 3.93 (td, J = 11.4, 2.8 Hz, 1H), 3.32 (d, J = 13.6 Hz, 1H), 3.15 – 3.00 (m, 3H), 2.63 (br, 1H).

^{13}C NMR (126 MHz, $CDCl_3$, 23 ºC, δ): 140.7, 137.4, 135.4, 124.5, 124.2, 123.1, 123.0, 122.2, 68.4, 51.6, 45.8, 29.8.

HRMS-EI(m/z) calc'd for $C_{12}H_{13}NOS$ $[M]^+$, 219.0714; found, 219.0712; deviation: –0.5 ppm.

Bicyclo[2.2.1]heptane derived morpholine 29

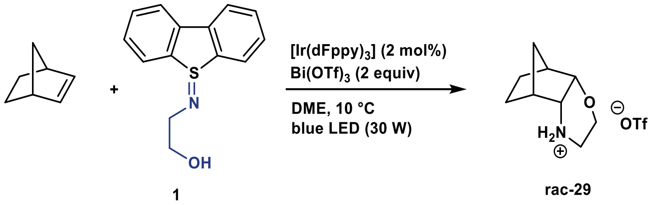

Under a nitrogen atmosphere, to a 4-mL borosilicate vial equipped with a magnetic stir bar were added

sulfilimine **1** (48.6 mg, 0.200 mmol, 1.00 equiv.), [Ir(dFppy)$_3$] (3.0 mg, 4.0 μmol, 2.0 mol%), Bi(OTf)$_3$ (131 mg, 0.200 mmol, 1.00 equiv.), norbornene (75.2 mg, 0.800 mmol, 4.00 equiv.), and DME (1 mL, c = 0.2 M). The vial was sealed with a septum-cap and irradiated for 6 h at 10 °C using a photoreactor equipped with a blue LED module (KT-Elektronik, "100W Power LED blau 450 nm Aquarium", 450 nm, 30 W), cooled with two Peltier-elements (TEC1-12706). Then, the reaction mixture was concentrated to dryness. The residue was purified by chromatography on silica gel eluting with CH_2Cl_2/MeOH (50/1–10/1 (v/v)) to afford 30.3 mg of cyclization product **29** as a colorless oil (50% yield, >20:1 dr).

R*f* = 0.15 (DCM/MeOH = 10/1 (v/v)).

NMR Spectroscopy:

^{1}H NMR (500 MHz, $CDCl_3$, 23 ºC, δ): 6.95 (br, 2H), 3.63 (dt, *J* = 10.4, 5.0 Hz, 1H), 3.55 (dt, *J* = 10.5, 5.0 Hz, 1H), 3.43 – 3.33 (m, 1H), 3.18 (q, *J* = 5.2 Hz, 2H), 2.29 (d, *J* = 5.2 Hz, 1H), 2.22 (s, 1H), 1.65 – 1.31 (m, 4H), 1.10 – 0.91 (m, 3H).

^{13}C NMR (126 MHz, CD_3CN, 23 ºC, δ): 121.7 (q, *J* = 319.7 Hz), 83.8, 63.8, 41.3, 41.1, 39.9, 36.1, 35.3, 29.1, 24.9.

^{19}F NMR (471 MHz, $CDCl_3$, 23 ºC, δ): –78.41.

HRMS-ESI(m/z) calc'd for $C_9H_{16}NO$ $[M]^+$, 154.1228; found, 154.1226; deviation: –0.8 ppm.

Octahydrobenzo[1,4]oxazine 30

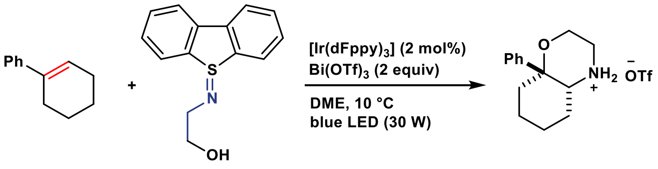

1 rac-30

Under a nitrogen atmosphere, to a 4-mL borosilicate vial equipped with a magnetic stir bar were added sulfilimine **1** (97.3 mg, 0.400 mmol, 2.00 equiv.), [Ir(dFppy)$_3$] (3.1 mg, 4.0 μmol, 2.0 mol%), Bi(OTf)$_3$ (262.5 mg, 0.400 mmol, 2.00 equiv.), DME (1 mL, c = 0.2 M), and 1-Phenyl-1-cyclohexene (32.0 μL, 31.6 mg, 0.200 mmol, 1.00 equiv.). The vial was sealed with a septum-cap and irradiated for 6 h at 10 °C using a photoreactor equipped with a blue LED module (KT-Elektronik, "100W Power LED blau 450 nm Aquarium", 450 nm, 30 W), cooled with two Peltier-elements (TEC1-12706). Then, the reaction mixture was concentrated to dryness. The residue was purified by chromatography on silica gel eluting with CH_2Cl_2/MeOH (100/1–10/1 (v/v)) to afford 33.0 mg of cyclization product **30** as a yellow oil (45% yield, >20:1 dr).

R*f* = 0.46 (DCM/MeOH = 10/1 (v/v)).

NMR Spectroscopy:

^{1}H NMR (500 MHz, $CDCl_3$, 23 ºC, δ): 7.71 (br, 2H), 7.48 – 7.18 (m, 5H), 4.18 (dd, *J* = 11.6, 4.9 Hz, 1H), 3.90 – 3.78 (m, 2H), 3.51 – 3.38 (m, 1H), 3.07 (d, *J* = 13.0 Hz, 1H), 2.30 – 2.19 (m, 1H), 2.14 – 2.06 (m,

1H), 2.00 (dd, *J* = 14.7, 2.6 Hz, 1H), 1.96 – 1.89 (m, 1H), 1.73 – 1.61 (m, 1H), 1.56 – 1.49 (m, 1H), 1.49 – 1.34 (m, 2H).

^{13}C NMR (126 MHz, $CDCl_3$, 23 ºC, δ): 140.0, 129.8, 128.4, 125.3, 119.92 (q, *J* = 317.7 Hz), 76.0, 58.3, 53.2, 42.0, 37.9, 23.7, 23.7, 21.2.

^{19}F NMR (565 MHz, $CDCl_3$, 23 ºC, δ): –78.56.

HRMS-ESI(m/z) calc'd for $C_{14}H_{20}NO$ $[M]^+$, 218.1542; found, 218.1539; deviation: +1.1 ppm.

9b-Methyl hexahydroindeno[1,4]oxazine 31

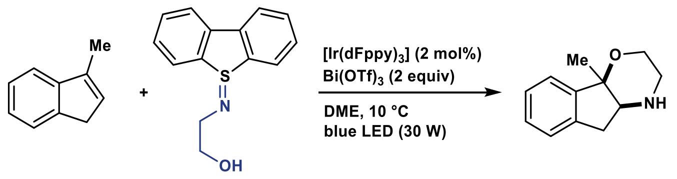

1

rac-31

Under a nitrogen atmosphere, to a 4-mL borosilicate vial equipped with a magnetic stir bar were added sulfilimine **1** (97.2 mg, 0.400 mmol, 2.00 equiv.), [Ir(dFppy)$_3$] (3.0 mg, 4.0 µmol, 2.0 mol%), Bi(OTf)$_3$ (262 mg, 0.400 mmol, 2.00 equiv.), DME (1 mL, c = 0.2 M), and 3-methyl-1H-indene (26.0 mg, 0.200 mmol, 1.00 equiv.). The vial was sealed with a septum-cap and irradiated for 6 h at 10 °C using a photoreactor equipped with a blue LED module (KT-Elektronik, "100W Power LED blau 450 nm Aquarium", 450 nm, 30 W), cooled with two Peltier-elements (TEC1-12706). Then, the reaction mixture was concentrated to dryness. The residue was dissolved in DCM (5 mL) and washed with saturated aqueous sodium carbonate solution (5 mL). The aqueous phase was extracted with DCM (2 × 5 mL). The organic phase was dried over Na_2SO_4, filtered, and the solvent was removed under reduced pressure. The residue was purified by chromatography on silica gel eluting with CH_2Cl_2/MeOH (50/1–10/1 (v/v)) to afford 26.1 mg of cyclization product **31** as a colorless oil (69% yield, >20:1 dr).

R_f = 0.25 (DCM/MeOH = 10/1 (v/v)).

NMR Spectroscopy:

^{1}H NMR (500 MHz, $CDCl_3$, 23 ºC, δ): 7.29 – 7.23 (m, 1H), 7.22 – 7.13 (m, 3H), 3.65 – 3.54 (m, 1H), 3.48 – 3.33 (m, 1H), 3.27 (d, *J* = 5.3 Hz, 1H), 2.98 (dd, *J* = 15.9, 5.3 Hz, 1H), 2.83 (td, *J* = 11.4, 10.1, 3.0 Hz, 1H), 2.67 (d, *J* = 13.1 Hz, 1H), 2.59 (dd, *J* = 15.9, 2.0 Hz, 1H), 2.02 (br, 1H), 1.32 (s, 3H).

^{13}C NMR (126 MHz, $CDCl_3$, 23 ºC, δ): 144.5, 140.2, 128.2, 127.2, 126.1, 123.4, 82.0, 62.6, 61.8, 43.5, 36.8, 25.6.

HRMS-EI(m/z) calc'd for $C_{12}H_{15}NO$ $[M]^+$, 189.1148; found, 189.1148; deviation: +0.3 ppm.

Hexahydroindeno[1,4]oxazine 32

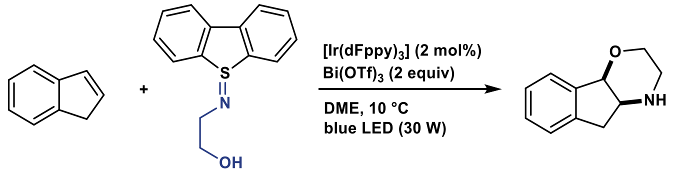

Under a nitrogen atmosphere, to a 4-mL borosilicate vial equipped with a magnetic stir bar were added sulfilimine **1** (97.2 mg, 0.400 mmol, 2.00 equiv.), [Ir(dFppy)$_3$] (3.0 mg, 4.0 µmol, 2.0 mol%), Bi(OTf)$_3$ (262 mg, 0.400 mmol, 2.00 equiv.), DME (1 mL, c = 0.2 M), and 1H-indene (23 µL, 23 mg, 0.20 mmol, 1.0 equiv.). The vial was sealed with a septum-cap and irradiated for 6 h at 10 °C using a photoreactor equipped with a blue LED module (KT-Elektronik, "100W Power LED blau 450 nm Aquarium", 450 nm, 30 W), cooled with two Peltier-elements (TEC1-12706). Then, the reaction mixture was concentrated to dryness. The residue was dissolved in DCM (5 mL) and washed with saturated aqueous sodium carbonate solution (5 mL). The aqueous phase was extracted with DCM (2 × 5 mL). The organic phase was dried over Na_2SO_4, filtered, and the solvent was removed under reduced pressure. The residue was purified by chromatography on silica gel eluting with CH_2Cl_2/MeOH (50/1–10/1 (v/v)) to afford 26.0 mg of cyclization product **32** as a colorless oil (74% yield, >20:1 dr).

R*f* = 0.25 (DCM/MeOH = 10/1 (v/v)).

NMR Spectroscopy:

^{1}H NMR (500 MHz, $CDCl_3$, 23 ºC, δ): 7.40 (dd, *J* = 5.3, 2.7 Hz, 1H), 7.30 – 7.20 (m, 3H), 4.89 (d, *J* = 4.6 Hz, 1H), 3.72 – 3.58 (m, 3H), 3.00 – 2.84 (m, 4H), 2.10 (br, 1H).

^{13}C NMR (126 MHz, $CDCl_3$, 23 ºC, δ): 141.9, 140.7, 128.6, 127.0, 125.9, 125.0, 78.9, 63.5, 56.1, 42.2, 35.2.

HRMS-ESI(m/z) calc'd for $C_{11}H_{13}NO$ $[M+H]^+$, 175.0995; found, 175.0992; deviation: –1.9 ppm.

Spiro[chromane-4,2'-morpholine] 33

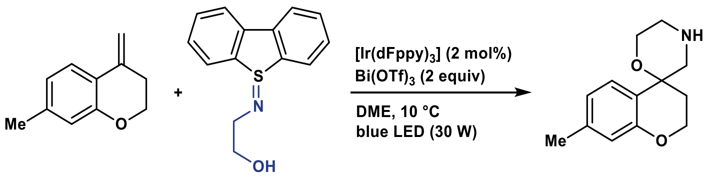

Under a nitrogen atmosphere, to a 4-mL borosilicate vial equipped with a magnetic stir bar were added sulfilimine **1** (97.2 mg, 0.400 mmol, 2.00 equiv.), [Ir(dFppy)$_3$] (3.0 mg, 4.0 µmol, 2.0 mol%), Bi(OTf)$_3$ (262 mg, 0.400 mmol, 2.00 equiv.), DME (1 mL, c = 0.2 M), and 7-methyl-4-methylenechromane[5] (32.0 mg, 0.200 mmol, 1.00 equiv.). The vial was sealed with a septum-cap and irradiated for 6 h at 10 °C using a photoreactor equipped with a blue LED module (KT-Elektronik, "100W Power LED blau 450 nm Aquarium",

450 nm, 30 W), cooled with two Peltier-elements (TEC1-12706). Then, the reaction mixture was concentrated to dryness. The residue was dissolved in DCM (5 mL) and washed with saturated aqueous sodium carbonate solution (5 mL). The aqueous phase was extracted with DCM (2 × 5 mL). The organic phase was dried over Na_2SO_4, filtered, and the solvent was removed under reduced pressure. The residue was purified by chromatography on silica gel eluting with CH_2Cl_2/MeOH (50/1–10/1 (v/v)) to afford 19.5 mg of cyclization product **33** as a colorless oil (45% yield).

R*f* = 0.31 (DCM/MeOH = 10/1 (v/v)).

NMR Spectroscopy:

^{1}H NMR (500 MHz, $CDCl_3$, 23 ºC, δ): 7.39 (s, 1H), 6.97 (dd, *J* = 8.3, 2.2 Hz, 1H), 6.70 (d, *J* = 8.3 Hz, 1H), 4.28 (ddd, *J* = 11.4, 5.8, 3.8 Hz, 1H), 4.08 (td, *J* = 10.9, 2.7 Hz, 1H), 3.95 – 3.88 (m, 1H), 3.75 (dd, *J* = 11.8, 5.4 Hz, 1H), 3.12 (d, *J* = 12.5 Hz, 1H), 3.05 (td, *J* = 11.8, 3.7 Hz, 1H), 2.98 (d, *J* = 12.6 Hz, 1H), 2.90 (d, *J* = 12.9 Hz, 1H), 2.59 (ddd, *J* = 13.7, 5.8, 2.7 Hz, 1H), 2.29 (s, 3H), 2.21 (ddd, *J* = 12.9, 10.3, 3.9 Hz, 1H), 1.85 (br, 1H).

^{13}C NMR (126 MHz, $CDCl_3$, 23 ºC, δ): 152.6, 130.0, 129.9, 127.8, 125.5, 116.5, 77.4, 69.6, 63.5, 62.1, 54.8, 46.2, 28.5, 20.8.

HRMS-EI(m/z) calc'd for $C_{13}H_{17}NO_2$ $[M]^+$, 219.1254; found, 219.1254; deviation: –0.0 ppm.

2-Styrylmorpholine 34

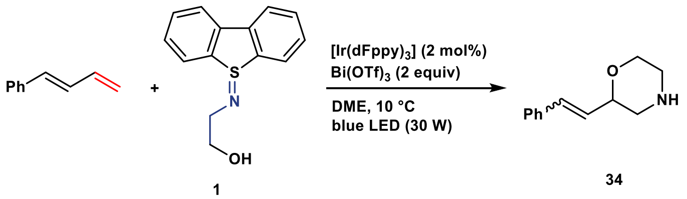

1

34

Under a nitrogen atmosphere, to a 4-mL borosilicate vial equipped with a magnetic stir bar were added sulfilimine **1** (48.7 mg, 0.200 mmol, 2.00 equiv.), [Ir(dFppy)$_3$] (1.5 mg, 2.0 µmol, 2.0 mol%), Bi(OTf)$_3$ (131.2 mg, 0.200 mmol, 2.00 equiv.), DME (1 mL, c = 0.1 M), and (E)-buta-1,3-dien-1-ylbenzene (14 µL, 13 mg, 0.10 mmol, 1.0 equiv.). The vial was sealed with a septum-cap and irradiated for 6 h at 10 °C using a photoreactor equipped with a blue LED module (KT-Elektronik, "100W Power LED blau 450 nm Aquarium", 450 nm, 30 W), cooled with two Peltier-elements (TEC1-12706). Then, the reaction mixture was concentrated to dryness. The residue was dissolved in DCM (5 mL) and washed with saturated aqueous sodium carbonate solution (5 mL). The aqueous phase was extracted with DCM (2 × 5 mL). The organic phase was dried over Na_2SO_4, filtered, and the solvent was removed under reduced pressure. The residue was purified by chromatography on silica gel eluting with CH_2Cl_2/MeOH (100/1–10/1 (v/v)) to afford 10.1 mg of cyclization product **34** as a yellow oil (53% yield, 1:1 Z:E).

R*f* = 0.44 (DCM/MeOH = 10/1 (v/v)).

NMR Spectroscopy:

^{1}**H NMR** (500 MHz, $CDCl_3$, 23 ºC, δ): 7.40 – 7.22 (m, 12H) 6.78 – 6.68 (m, 2H), 6.04 (dd, *J* = 16.1, 6.0 Hz, 1H), 5.51 (dd, *J* = 11.7, 8.6 Hz, 1H), 4.71 – 4.63 (m, 1H), 4.47 – 4.40 (m, 1H), 4.11 (dd, *J* = 13.0, 3.7 Hz, 1H), 4.09 – 4.03 (m, 1H), 4.01 – 3.86 (m, 2H), 3.44 (dd, *J* = 12.5, 2.2 Hz, 1H), 3.37 – 3.30 (m, 3H), 3.19 – 3.06 (m, 2H), 3.00 – 2.91 (m, 2H).

^{13}**C NMR** (151 MHz, $CDCl_3$, 23 ºC, δ): 136.8, 136.6, 136.2, 135.1, 129.8, 129.7, 129.7, 129.7, 129.2, 127.7, 126.6, 124.7, 74.6, 70.8, 64.5, 64.1, 48.1, 47.3, 43.5, 43.4.

HRMS-ESI(m/z) calc'd for $C_{12}H_{15}NO$ $[M+H]^+$, 190.1229; found, 190.1226; deviation: +1.2 ppm.

(E)-2-(Propen-1-yl)morpholine 35

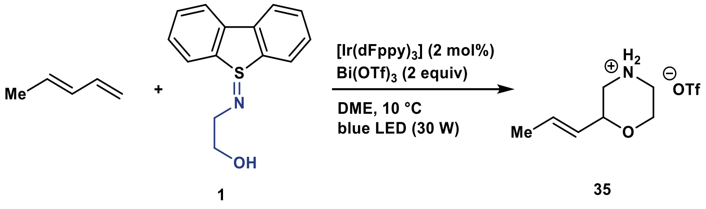

Under a nitrogen atmosphere, to a 4-mL borosilicate vial equipped with a magnetic stir bar were added sulfilimine **1** (97.2 mg, 0.400 mmol, 2.00 equiv.), $[Ir(dFppy)_3]$ (3.0 mg, 4.0 µmol, 2.0 mol%), $Bi(OTf)_3$ (262 mg, 0.400 mmol, 2.00 equiv.), DME (1 mL, c = 0.2 M), and (E)-penta-1,3-diene (13.6 mg, 0.200 mmol, 1.00 equiv.). The vial was sealed with a septum-cap and irradiated for 6 h at 10 °C using a photoreactor equipped with a blue LED module (KT-Elektronik, "100W Power LED blau 450 nm Aquarium", 450 nm, 30 W), cooled with two Peltier-elements (TEC1-12706). Then, the reaction mixture was concentrated to dryness. The residue was purified by chromatography on silica gel eluting with CH_2Cl_2/MeOH (50/1–10/1 (v/v)) to afford 39.3 mg of cyclization product **35** as a colorless oil (71% yield, E:Z >20:1).

$\mathbf{R}_{\boldsymbol{f}}$ = 0.18 (DCM/MeOH = 10/1 (v/v)).

NMR Spectroscopy:

^{1}**H NMR** (500 MHz, $CDCl_3$, 23 ºC, δ): 5.92 – 5.81 (m, 1H), 5.36 (ddd, *J* = 15.4, 6.6, 1.8 Hz, 1H), 4.80 (br, 2H), 4.21 (t, *J* = 7.6 Hz, 1H), 4.05 (dd, *J* = 13.0, 3.4 Hz, 1H), 3.91 (td, *J* = 12.5, 2.3 Hz, 1H), 3.40 – 3.23 (m, 2H), 3.10 (td, *J* = 12.5, 3.9 Hz, 1H), 2.86 (dd, *J* = 13.0, 11.0 Hz, 1H), 1.72 (dd, *J* = 6.6, 2.1 Hz, 3H).

^{13}**C NMR** (126 MHz, $CDCl_3$, 23 ºC, δ): 132.1, 126.0, 120.1 (q, *J* = 318.8 Hz), 73.8, 63.4, 47.8, 43.4, 18.0.

^{19}**F NMR** (471 MHz, $CDCl_3$, 23 ºC, δ): –78.51.

HRMS-ESI(m/z) calc'd for $C_7H_{14}NO$ $[M]^+$, 128.1071; found, 128.1070; deviation: –1.0 ppm.

2-(4-Benzamide)phenylmorpholine 36

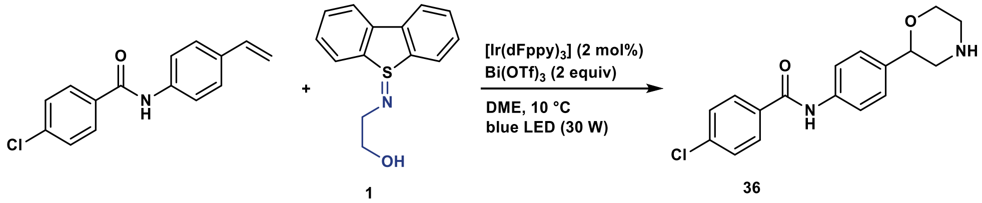

Under a nitrogen atmosphere, to a 4-mL borosilicate vial equipped with a magnetic stir bar were added 4-chloro-N-(4-vinylphenyl)benzamide (51.4 mg, 0.200 mmol, 1.00 equiv.), sulfilimine **1** (97.2 mg, 0.400 mmol, 2.00 equiv.), [Ir(dFppy)$_3$] (3.0 mg, 4.0 µmol, 2.0 mol%), Bi(OTf)$_3$ (262 mg, 0.400 mmol, 2.00 equiv.), and DME (1 mL, c = 0.2 M). The vial was sealed with a septum-cap and irradiated for 6 h at 10 °C using a photoreactor equipped with a blue LED module (KT-Elektronik, "100W Power LED blau 450 nm Aquarium", 450 nm, 30 W), cooled with two Peltier-elements (TEC1-12706). Then, the reaction mixture was concentrated to dryness. The residue was dissolved in DCM (5 mL) and washed with saturated aqueous sodium carbonate solution (5 mL). The aqueous phase was extracted with DCM (2 × 5 mL). The organic phase was dried over Na_2SO_4, filtered, and the solvent was removed under reduced pressure. The residue was purified by chromatography on silica gel eluting with CH_2Cl_2/MeOH (50/1–10/1 (v/v)) to afford 41.0 mg of cyclization product **36** as a colorless solid (65% yield).

R*f* = 0.15 (DCM/MeOH = 10/1 (v/v)).

NMR Spectroscopy:

^{1}H NMR (500 MHz, d$_6$-DMSO, 23 ºC, δ): 10.32 (s, 1H), 7.99 (d, *J* = 8.2 Hz, 2H), 7.73 (d, *J* = 8.2 Hz, 2H), 7.60 (d, *J* = 8.2 Hz, 2H), 7.30 (d, *J* = 8.1 Hz, 2H), 4.35 (d, *J* = 10.2 Hz, 1H), 3.87 (d, *J* = 11.0 Hz, 1H), 3.60 (dt, *J* = 12.4, 7.4 Hz, 1H), 3.27 (br, 1H), 2.91 (d, *J* = 12.3 Hz, 1H), 2.74 (d, *J* = 6.8 Hz, 2H), 2.57 – 2.51 (m, 1H).

^{13}C NMR (126 MHz, d$_6$-DMSO, 23 ºC, δ): 164.3, 138.1, 136.5, 136.4, 133.6, 129.6, 128.4, 126.2, 120.1, 78.0, 67.6, 52.9, 45.1.

HRMS-ESI(m/z) calc'd for $C_{17}H_{18}N_2O_2Cl$ [M+H]$^+$, 317.1052; found, 317.1051; deviation: –0.2 ppm.

Spirobicyclo[2.2.1]heptane-2,2'-morpholine 37

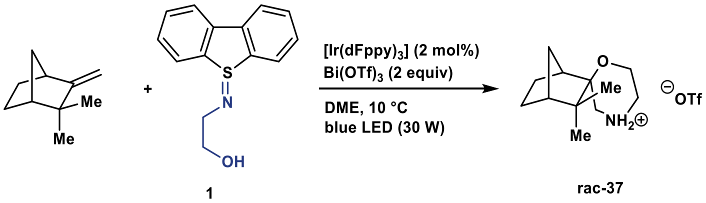

Under a nitrogen atmosphere, to a 4-mL borosilicate vial equipped with a magnetic stir bar were added sulfilimine **1** (48.6 mg, 0.200 mmol, 1.00 equiv.), [Ir(dFppy)$_3$] (3.0 mg, 4.0 µmol, 2.0 mol%), Bi(OTf)$_3$ (131 mg, 0.200 mmol, 1.00 equiv.), camphene (109 mg, 0.800 mmol, 4.00 equiv.), and DME (1 mL, c = 0.2 M). The vial

was sealed with a septum-cap and irradiated for 6 h at 10 °C using a photoreactor equipped with a blue LED module (KT-Elektronik, "100W Power LED blau 450 nm Aquarium", 450 nm, 30 W), cooled with two Peltier-elements (TEC1-12706). Then, the reaction mixture was concentrated to dryness. The residue was purified by chromatography on silica gel eluting with CH_2Cl_2/MeOH (50/1–10/1 (v/v)) to afford 43.5 mg of cyclization product **37** as a colorless oil (63% yield, >20:1 dr).

R*f* = 0.30 (DCM/MeOH = 10/1 (v/v)).

NMR Spectroscopy:

^{1}**H NMR** (500 MHz, $CDCl_3$, 23 ºC, δ): 7.97 (br, 2H), 4.06 (td, *J* = 13.0, 2.7 Hz, 1H), 3.78 (dd, *J* = 13.6, 4.4 Hz, 1H), 3.52 (d, *J* = 13.1 Hz, 1H), 3.30 (d, *J* = 12.4 Hz, 1H), 3.06 – 3.00 (m, 1H), 2.89 – 2.81 (m, 2H), 1.94 (d, *J* = 10.2 Hz, 1H), 1.77 (d, *J* = 2.3 Hz, 1H), 1.63 (t, *J* = 13.2 Hz, 1H), 1.57 – 1.48 (m, 1H), 1.36 – 1.28 (m, 1H), 1.27 – 1.20 (m, 1H), 1.15 (d, *J* = 10.1 Hz, 1H), 0.95 (s, 3H), 0.86 (s, 3H).

^{13}**C NMR** (126 MHz, $CDCl_3$, 23 ºC, δ): 120.1 (q, *J* = 318.3 Hz), 81.2, 58.1, 49.3, 45.0, 44.9, 43.6, 39.7, 34.4, 24.3, 24.0, 22.6, 21.9.

^{19}**F NMR** (471 MHz, $CDCl_3$, 23 ºC, δ): –78.39.

HRMS-ESI(m/z) calc'd for $C_{12}H_{22}NO$ $[M]^+$, 196.1697; found, 196.1696; deviation: –0.5 ppm.

Pyriproxyphen derived alkene S1

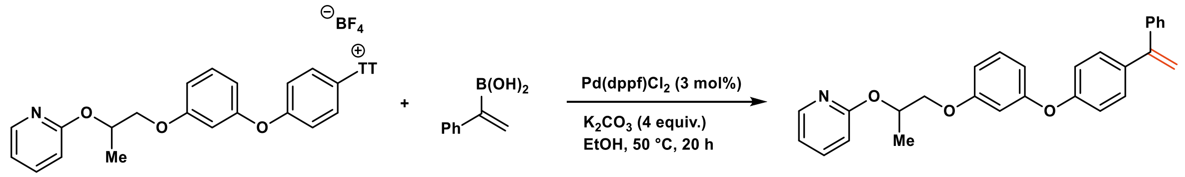

Under a nitrogen atmosphere, a 10 ml roundbottom flask, equipped with a teflon coated magnetic stirbar, and fitted with a septum, was charged with pyriproxyphen derived thianthrenium salt (125 mg, 0.200 mmol, 1.00 equiv.), Pd(dppf)Cl2 (4.4 mg, 0.006 mmol, 3.0 mol%), K_2CO_3 (110 mg, 0.800 mmol, 4.00 equiv.), and 1-Phenylvinylboronic acid (59 mg, 0.40 mmol, 2.0 equiv.). After adding EtOH (0.80 ml, c = 0.25 M), the mixture was degassed by purging with argon for 5 minutes. The mixture was stirred at 50 °C for 20 h. The reaction mixture was concentrated under reduced pressure. The residue was purified by chromatography on silica gel eluting with hexane/EtOAc (20:1 (v/v)). The product was dried in vacuo to afford 78 mg (92 %) of **S1** as a colorless solid.

R*f* = 0.25 (hexane/EtOAc = 4/1 (v/v)).

NMR Spectroscopy:

^{1}**H NMR** (500 MHz, $CDCl_3$, 23 ºC, δ): 8.18 (ddd, *J* = 5.0, 2.0, 0.9 Hz, 1H), 7.60 (d, *J* = 2.0 Hz, 1H), 7.40 – 7.26 (m, 7H), 7.02 (d, *J* = 9.2 Hz, 2H), 6.96 (d, *J* = 9.2 Hz, 2H), 6.92 (d, *J* = 8.9 Hz, 2H), 6.88 (ddd, *J* = 7.0, 5.0, 1.0 Hz, 1H), 6.77 (dt, *J* = 8.4, 0.9 Hz, 1H), 5.63 (qt, *J* = 6.4, 5.0 Hz, 1H), 5.44 (d, *J* = 1.2 Hz,

1H), 5.41 (d, *J* = 1.2 Hz, 1H), 4.22 (dd, *J* = 9.9, 5.3 Hz, 1H), 4.10 (dd, *J* = 9.8, 4.8 Hz, 1H), 1.51 (d, *J* = 6.4 Hz, 3H).

^{13}C NMR (126 MHz, $CDCl_3$, 23 ºC, δ): 163.2, 158.4, 155.5, 150.2, 149.5, 146.8, 141.7, 138.9, 135.8, 129.6, 128.4, 128.3, 127.8, 121.0, 117.2, 116.9, 115.9, 113.6, 111.8, 71.2, 69.5, 17.1.

HRMS-ESI(m/z) calc'd for $C_{28}H_{26}NO_3$ $[M+H]^+$, 424.1910; found, 424.1907; deviation: –0.6 ppm.

Pyriproxyphen derived morpholine 38

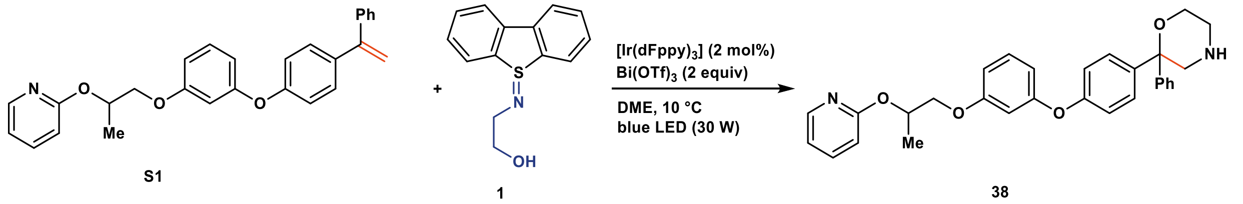

Under a nitrogen atmosphere, to a 4-mL borosilicate vial equipped with a magnetic stir bar were added **S1** (63.5 mg, 0.150 mmol, 1.00 equiv.), sulfilimine **1** (72.9 mg, 0.300 mmol, 2.00 equiv.), $[Ir(dFppy)_3]$ (2.2 mg, 3.0 μmol, 2.0 mol%), $Bi(OTf)_3$ (196 mg, 0.300 mmol, 2.00 equiv.), and DME (1 mL, c = 0.2 M). The vial was sealed with a septum-cap and irradiated for 6 h at 10 °C using a photoreactor equipped with a blue LED module (KT-Elektronik, "100W Power LED blau 450 nm Aquarium", 450 nm, 30 W), cooled with two Peltier-elements (TEC1-12706). Then, the reaction mixture was concentrated to dryness. The residue was dissolved in DCM (5 mL) and washed with saturated aqueous sodium carbonate solution (5 mL). The aqueous phase was extracted with DCM (2 × 5 mL). The organic phase was dried over Na_2SO_4, filtered, and the solvent was removed under reduced pressure. The residue was purified by chromatography on silica gel eluting with CH_2Cl_2/MeOH (50/1–20/1 (v/v)) to afford 56.5 mg of cyclization product **38** as a colorless solid (78% yield).

R*f* = 0.20 (DCM/MeOH = 15/1 (v/v)).

NMR Spectroscopy:

^{1}H NMR (500 MHz, CD_3CN, 23 ºC, δ): 8.12 (ddd, *J* = 5.2, 2.1, 0.8 Hz, 1H), 7.63 (ddd, *J* = 8.4, 7.2, 2.1 Hz, 1H), 7.42 – 7.28 (m, 5H), 7.26 – 7.19 (m, 1H), 6.96 – 6.88 (m, 4H), 6.85 (d, *J* = 9.0 Hz, 2H), 6.71 (d, *J* = 8.2 Hz, 1H), 5.58 – 5.48 (m, 1H), 4.14 (dd, *J* = 10.2, 6.0 Hz, 1H), 4.08 (dd, *J* = 10.4, 4.1 Hz, 1H), 3.67 – 3.62 (m, 2H), 3.43 (d, *J* = 1.5 Hz, 2H), 2.89 – 2.84 (m, 2H), 1.38 (d, *J* = 6.6 Hz, 3H).

^{13}C NMR (126 MHz, CD_3CN, 23 ºC, δ): 164.2, 158.5, 156.3, 151.0, 147.9, 145.1, 140.2, 139.1, 129.4, 129.0, 128.0, 127.3, 125.7, 123.1, 121.9, 120.6, 118.0, 116.8, 112.2, 79.0, 72.0, 70.3, 62.6, 53.6, 46.1, 17.0.

HRMS-ESI(m/z) calc'd for $C_{30}H_{31}N_2O_4$ $[M+H]^+$, 483.2279; found, 483.2278; deviation: –0.2 ppm.

Bifonazole derived alkene S2

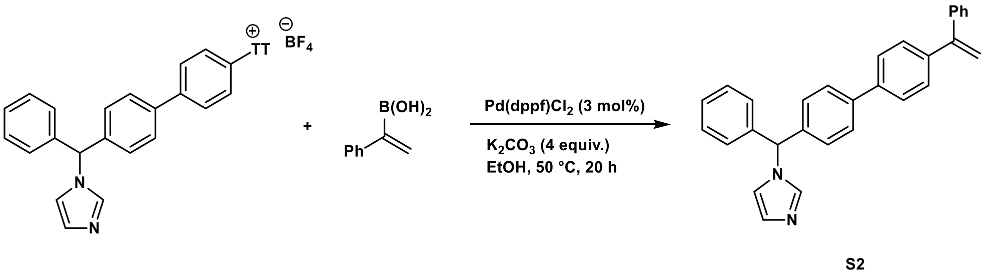

Under a nitrogen atmosphere, a 10 ml roundbottom flask, equipped with a teflon coated magnetic stirbar, and fitted with a septum, was charged with bifonazole derived thianthrenium salt (122 mg, 0.200 mmol, 1.00 equiv.), Pd(dppf)Cl2 (4.4 mg, 0.006 mmol, 3.0 mol%), K_2CO_3 (110 mg, 0.800 mmol, 4.00 equiv.), and 1-Phenylvinylboronic acid (59 mg, 0.40 mmol, 2.0 equiv.). After adding EtOH (0.80 ml, c = 0.25 M), the mixture was degassed by purging with argon for 5 minutes. The mixture was stirred at 50 °C for 20 h. The reaction mixture was concentrated under reduced pressure. The residue was purified by chromatography on silica gel eluting with hexane/EtOAc (4:1 (v/v)). The product was dried in vacuo to afford 68 mg (82 %) of **S2** as a colorless solid.

R*f* = 0.20 (hexane/EtOAc = 1/1 (v/v)).

NMR Spectroscopy:

^{1}H NMR (500 MHz, $CDCl_3$, 23 ºC, δ): 7.61 (d, *J* = 8.4 Hz, 2H), 7.56 (d, *J* = 8.2 Hz, 2H), 7.52 – 7.48 (m, 1H), 7.43 (d, *J* = 8.4 Hz, 2H), 7.41 – 7.33 (m, 8H), 7.22 – 7.11 (m, 5H), 6.95 – 6.91 (m, 1H), 6.57 (s, 1H), 5.54 (d, *J* = 1.4 Hz, 1H), 5.49 (d, *J* = 1.4 Hz, 1H).

^{13}C NMR (126 MHz, $CDCl_3$, 23 ºC, δ): 149.6, 141.4, 140.9, 140.9, 139.6, 139.1, 138.2, 129.5, 129.0, 129.0, 128.8, 128.6, 128.5, 128.4, 128.3, 128.2, 127.9, 127.5, 126.9, 119.6, 114.6, 64.9.

HRMS-ESI(m/z) calc'd for $C_{30}H_{25}N_2$ $[M+H]^+$, 413.2012; found, 413.2012; deviation: –0.1 ppm.

Bifonazole derived morpholine 39

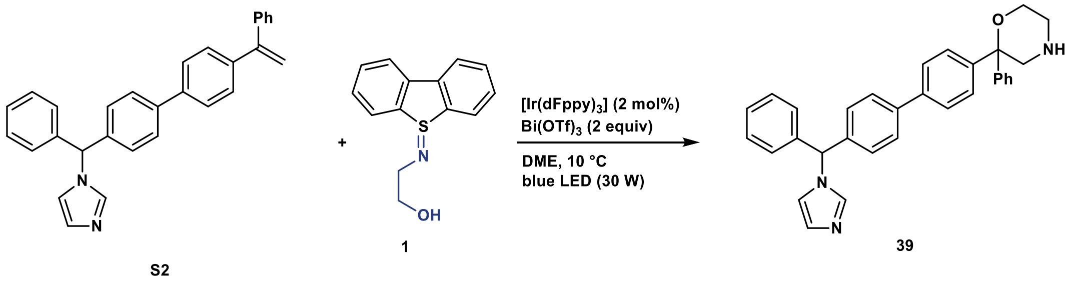

Under a nitrogen atmosphere, to a 4-mL borosilicate vial equipped with a magnetic stir bar were added **S2** (41.2 mg, 0.100 mmol, 1.00 equiv.), sulfilimine **1** (48.6 mg, 0.200 mmol, 2.00 equiv.), $[Ir(dFppy)_3]$ (1.5 mg, 2.0 μmol, 2.0 mol%), $Bi(OTf)_3$ (131 mg, 0.200 mmol, 2.00 equiv.), and DME (1 mL, c = 0.1 M). The vial was sealed with a septum-cap and irradiated for 6 h at 10 °C using a photoreactor equipped with a blue LED

module (KT-Elektronik, “100W Power LED blau 450 nm Aquarium”, 450 nm, 30 W), cooled with two Peltier-elements (TEC1-12706). Then, the reaction mixture was concentrated to dryness. The residue was dissolved in DCM (5 mL) and washed with saturated aqueous sodium carbonate solution (5 mL). The aqueous phase was extracted with DCM (2 × 5 mL). The organic phase was dried over Na_2SO_4, filtered, and the solvent was removed under reduced pressure. The residue was purified by chromatography on silica gel eluting with CH_2Cl_2/MeOH (50/1–10/1 (v/v)) to afford 26.4 mg of cyclization product **39** as a colorless solid (56% yield).

R*f* = 0.20 (DCM/MeOH = 10/1 (v/v)).

NMR Spectroscopy:

^{1}H NMR (500 MHz, $CDCl_3$, 23 ºC, δ): 7.67 – 7.35 (m, 14H), 7.28 (d, *J* = 8.1 Hz, 1H), 7.21 – 7.06 (m, 5H), 6.90 (s, 1H), 6.57 (s, 1H), 3.78 (t, *J* = 4.9 Hz, 2H), 3.56 – 3.53 (m, 2H), 3.00 (t, *J* = 5.0 Hz, 2H).

^{13}C NMR (126 MHz, $CDCl_3$, 23 ºC, δ): 143.8, 143.6, 140.9, 139.1, 139.0, 138.2, 137.5, 129.5, 129.0, 128.6, 128.6, 128.5, 128.1, 127.5, 127.3, 127.3, 127.2, 126.7, 119.5, 64.9, 62.6, 53.5, 45.8.

HRMS-ESI(m/z) calc’d for $C_{32}H_{30}N_3O$ $[M+H]^+$, 472.2382; found, 472.2383; deviation: +0.2 ppm.

Dihydrodibenzothiepine 40

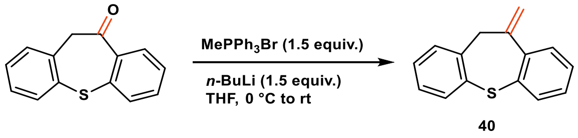

Under an argon atmosphere, to a 50-mL two-necked round bottom flask equipped with a magnetic stir bar were added $MePPh_3Br$ (1.07 g, 3.00 mmol, 1.50 equiv.) and THF (15 mL). After cooling the reaction mixture to 0 °C with an ice bath, n-BuLi (2.0 M in hexane, 1.5 mL, 3.0 mmol, 1.5 equiv.) was added slowly. The reaction mixture was stirred at 25 °C for 1 h, and then cooled to 0 °C with an ice bath. A THF (5 mL) solution of dibenzothiepin-10-one[6] (512 mg, 2.00 mmol, 1.00 equiv.) was added dropwise to the reaction mixture in 10 min. After stirring for 12 h, saturated NH_4Cl aqueous solution (10 mL) was added to the reaction mixture. The resulting mixture was extracted with EtOAc (3 × 20 mL). The organic phase was washed with brine (20 mL) and dried over Na_2SO_4. The solvent was removed under reduced pressure. The residue was purified by chromatography on silica gel eluting with hexane/EtOAc (50/1 (v/v)) to afford 403 mg of product **40** as a colorless solid (90% yield).

R*f* = 0.40 (hexane/EtOAc = 20/1 (v/v)).

NMR Spectroscopy:

^{1}H NMR (500 MHz, $CDCl_3$, 23 ºC, δ): 7.60 – 7.52 (m, 2H), 7.44 (dd, *J* = 7.2, 2.1 Hz, 1H), 7.36 – 7.24 (m, 2H), 7.23 – 7.12 (m, 3H), 5.43 (s, 1H), 5.16 (s, 1H), 4.09 (d, *J* = 2.3 Hz, 2H).

^{13}C NMR (126 MHz, $CDCl_3$, 23 ºC, δ): 145.1, 144.2, 138.5, 133.8, 132.7, 130.8, 129.6, 129.2, 129.2, 128.0, 127.6, 126.6, 126.4, 114.8, 43.0.

HRMS-EI(m/z) calc'd for $C_{15}H_{12}S$ $[M]^+$, 224.0658; found, 224.0654; deviation: –1.6 ppm.

Spiro[dibenzothiepine-10,2'-morpholine] 41

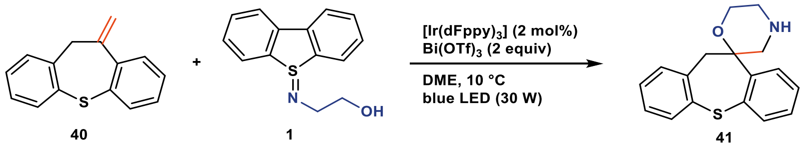

Under a nitrogen atmosphere, to a 4-mL borosilicate vial equipped with a magnetic stir bar were added sulfilimine **1** (97.2 mg, 0.400 mmol, 2.00 equiv.), [Ir(dFppy)3] (3.0 mg, 4.0 μmol, 2.0 mol%), $Bi(OTf)_3$ (262 mg, 0.400 mmol, 2.00 equiv.), DME (1 mL, c = 0.2 M), and 10-methylene-10,11-dihydrodibenzothiepine **40** (44.8 mg, 0.200 mmol, 1.00 equiv.). The vial was sealed with a septum-cap and irradiated for 6 h at 10 °C using a photoreactor equipped with a blue LED module (KT-Elektronik, "100W Power LED blau 450 nm Aquarium", 450 nm, 30 W), cooled with two Peltier-elements (TEC1-12706). Then, the reaction mixture was concentrated to dryness. The residue was dissolved in DCM (5 mL) and washed with saturated aqueous sodium carbonate solution (5 mL). The aqueous phase was extracted with DCM (2 × 5 mL). The organic phase was dried over Na_2SO_4, filtered, and the solvent was removed under reduced pressure. The residue was purified by chromatography on silica gel eluting with CH_2Cl_2/MeOH (50/1–10/1 (v/v)) to afford 33.4 mg of cyclization product **41** as a colorless oil (56% yield).

R_f = 0.30 (DCM/MeOH = 10/1 (v/v)).

NMR Spectroscopy:

^{1}H NMR (500 MHz, $CDCl_3$, 23 ºC, δ): 7.85 (dd, *J* = 8.1, 1.5 Hz, 1H), 7.54 (dd, *J* = 7.7, 1.4 Hz, 1H), 7.49 (dd, *J* = 7.6, 1.6 Hz, 1H), 7.43 (dd, *J* = 7.8, 1.5 Hz, 1H), 7.30 – 7.18 (m, 2H), 7.19 – 7.06 (m, 2H), 4.31 (td, *J* = 11.7, 3.4 Hz, 1H), 3.97 (d, *J* = 2.1 Hz, 2H), 3.86 (dd, *J* = 11.7, 3.4 Hz, 1H), 3.10 (td, *J* = 11.9, 3.7 Hz, 1H), 3.04 (dd, *J* = 12.3, 3.4 Hz, 1H), 2.92 (d, *J* = 12.8 Hz, 1H), 2.80 (d, *J* = 12.7 Hz, 1H), 2.01 (br, 1H).

^{13}C NMR (126 MHz, $CDCl_3$, 23 ºC, δ): 142.7, 140.8, 137.2, 133.5, 131.7, 131.3, 130.9, 130.3, 128.6, 127.7, 127.2, 126.8, 75.5, 61.5, 56.6, 46.2, 36.0.

HRMS-EI(m/z) calc'd for $C_{17}H_{17}NOS$ $[M]^+$, 283.1027; found, 283.1025; deviation: –0.4 ppm.

2-Methyl-6-phenylmorpholine 42

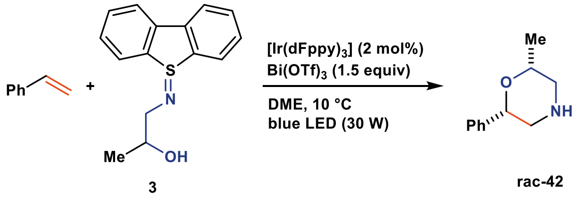

Under a nitrogen atmosphere, to a 4-mL borosilicate vial equipped with a magnetic stir bar were added sulfilimine **3** (38.6 mg, 0.150 mmol, 1.50 equiv.), [Ir(dFppy)3] (1.5 mg, 2.0 μmol, 2.0 mol%), $Bi(OTf)_3$ (98.4 mg,

0.150 mmol, 1.50 equiv.), DME (1 mL, c = 0.1 M), and styrene (12 µL, 10 mg, 0.10 mmol, 1.0 equiv.). The vial was sealed with a septum-cap and irradiated for 6 h at 10 °C using a photoreactor equipped with a blue LED module (KT-Elektronik, “100W Power LED blau 450 nm Aquarium”, 450 nm, 30 W), cooled with two Peltier-elements (TEC1-12706). Then, the reaction mixture was concentrated to dryness. The residue was dissolved in DCM (5 mL) and washed with saturated aqueous sodium carbonate solution (5 mL). The aqueous phase was extracted with DCM (2 × 5 mL). The organic phase was dried over Na_2SO_4, filtered, and the solvent was removed under reduced pressure. The residue was purified by chromatography on silica gel eluting with CH_2Cl_2/MeOH (50/1–10/1 (v/v)) to afford 9.9 mg of cyclization product **42** as a colorless oil (56% yield, 2:1 dr).

R*f* = 0.37 (DCM/MeOH = 10/1 (v/v)).

NMR Spectroscopy:

^{1}H NMR (600 MHz, $CDCl_3$, 23 ºC, δ): 7.43 – 7.28 (m, 7.5H), 6.09 (br, 1.5H), 4.99 (dd, *J* = 8.0, 3.2 Hz, 0.5H), 4.78 (dd, *J* = 11.1, 2.4 Hz, 1H), 4.18 (dt, *J* = 6.7, 4.0 Hz, 0.5H), 4.02 (dqd, *J* = 10.9, 6.3, 2.2 Hz, 1H), 3.40 – 3.38 (m, 0.5H), 3.38 – 3.33 (m, 1H), 3.29 (ddd, *J* = 12.6, 2.3, 1.2 Hz, 1H), 3.19 (dd, *J* = 12.7, 3.9 Hz, 0.5 H), 3.14 (dd, *J* = 12.9, 8.0 Hz, 0.5H), 2.96 (dd, *J* = 12.7, 4.2 Hz, 0.5H), 2.85 (dd, *J* = 12.7, 11.1 Hz, 1H), 2.75 (dd, *J* = 12.6, 10.9 Hz, 1H), 1.40 (d, *J* = 6.8 Hz, 1.5H), 1.29 (d, *J* = 6.3 Hz, 3H).

^{13}C NMR (151 MHz, $CDCl_3$, 23 ºC, δ): 138.3, 137.8, 128.9, 128.8, 128.7, 128.3, 126.4, 126.2, 76.2, 70.8, 69.8, 66.5, 49.3, 49.2, 48.9, 48.5, 18.8, 16.7.

HRMS-ESI(m/z) calc’d for $C_{11}H_{15}NO$ $[M+H]^+$, 178.1227; found, 178.1226; deviation: +0.5 ppm.

5-Ethyl-2-phenylmorpholine 43

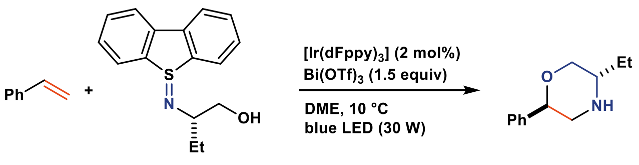

4 rac-43

Under a nitrogen atmosphere, to a 4-mL borosilicate vial equipped with a magnetic stir bar were added sulfilimine **4** (109 mg, 0.400 mmol, 2.00 equiv.), [Ir(dFppy)$_3$] (3.0 mg, 4.0 µmol, 2.0 mol%), $Bi(OTf)_3$ (262 mg, 0.400 mmol, 2.00 equiv.), DME (1 mL, c = 0.2 M), and styrene (23 µL, 21 mg, 0.20 mmol, 1.0 equiv.). The vial was sealed with a septum-cap and irradiated for 6 h at 10 °C using a photoreactor equipped with a blue LED module (KT-Elektronik, “100W Power LED blau 450 nm Aquarium”, 450 nm, 30 W), cooled with two Peltier-elements (TEC1-12706). Then, the reaction mixture was concentrated to dryness. The residue was dissolved in DCM (5 mL) and washed with saturated aqueous sodium carbonate solution (5 mL). The aqueous phase was extracted with DCM (2 × 5 mL). The organic phase was dried over Na_2SO_4, filtered, and the solvent was removed under reduced pressure. The residue was purified by chromatography on silica gel eluting with CH_2Cl_2/MeOH (50/1–10/1 (v/v)) to afford 25.2 mg of cyclization product **43** as a colorless oil (66% yield, >20:1 dr).

R*f* = 0.30 (DCM/MeOH = 10/1 (v/v)).

NMR Spectroscopy:

^{1}H NMR (500 MHz, CD_3CN, 23 ºC, δ): 7.41 – 7.11 (m, 5H), 4.37 (dd, *J* = 10.5, 2.6 Hz, 1H), 3.94 (dd, *J* = 11.0, 3.2 Hz, 1H), 3.26 (dd, *J* = 11.1, 10.3 Hz, 1H), 3.02 (dd, *J* = 12.4, 2.6 Hz, 1H), 2.72 – 2.63 (m, 2H), 1.41 – 1.22 (m, 2H), 0.93 (t, *J* = 7.6 Hz, 3H).

^{13}C NMR (126 MHz, CD_3CN, 23 ºC, δ): 142.1, 129.2, 128.4, 127.0, 79.5, 73.4, 56.7, 54.1, 25.8, 10.5.

HRMS-ESI(m/z) calc'd for $C_{12}H_{18}NO$ $[M+H]^+$, 192.1385; found, 192.1383; deviation: –1.2 ppm.

2,2-Diphenyl-1,4-oxazepane 44

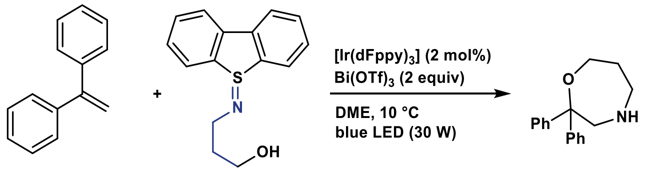

5 44

Under a nitrogen atmosphere, to a 4-mL borosilicate vial equipped with a magnetic stir bar were added sulfilimine **5** (103 mg, 0.400 mmol, 2.00 equiv.), $[Ir(dFppy)_3]$ (3.0 mg, 4.0 μmol, 2.0 mol%), $Bi(OTf)_3$ (262 mg, 0.400 mmol, 2.00 equiv.), DME (1 mL, c = 0.2 M), and 1,1-diphenylethylene (36.0 mg, 0.200 mmol, 1.00 equiv.). The vial was sealed with a septum-cap and irradiated for 6 h at 10 °C using a photoreactor equipped with a blue LED module (KT-Elektronik, "100W Power LED blau 450 nm Aquarium", 450 nm, 30 W), cooled with two Peltier-elements (TEC1-12706). Then, the reaction mixture was concentrated to dryness. The residue was dissolved in DCM (5 mL) and washed with saturated aqueous sodium carbonate solution (5 mL). The aqueous phase was extracted with DCM (2 × 5 mL). The organic phase was dried over Na_2SO_4, filtered, and the solvent was removed under reduced pressure. The residue was purified by chromatography on silica gel eluting with CH_2Cl_2/MeOH (50/1–10/1 (v/v)) to afford 38.0 mg of cyclization product **44** as a colorless solid (75% yield).

R*f* = 0.32 (DCM/MeOH = 10/1 (v/v)).

NMR Spectroscopy:

^{1}H NMR (500 MHz, $CDCl_3$, 23 ºC, δ): 7.46 – 7.36 (m, 4H), 7.32 (dd, *J* = 8.6, 7.0 Hz, 4H), 7.29 – 7.18 (m, 2H), 3.84 – 3.79 (m, 2H), 3.73 (s, 2H), 3.19 (br, 1H), 2.98 (t, *J* = 6.1 Hz, 2H), 1.95 – 1.87 (m, 2H).

^{13}C NMR (126 MHz, $CDCl_3$, 23 ºC, δ): 145.2, 128.5, 127.2, 126.4, 83.3, 63.8, 58.6, 49.6, 32.8.

HRMS-ESI(m/z) calc'd for $C_{17}H_{20}NO$ $[M+H]^+$, 254.1542; found, 254.1539; deviation: –0.8 ppm.

6,6-Dimethyl-2-phenyl-1,4-oxazepane 45

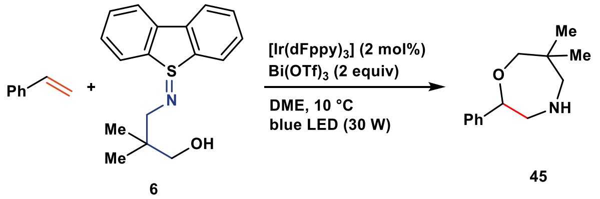

Under a nitrogen atmosphere, to a 4-mL borosilicate vial equipped with a magnetic stir bar were added sulfilimine **6** (57.1 mg, 0.200 mmol, 2.00 equiv.), [Ir(dFppy)$_3$] (1.5 mg, 2.0 µmol, 2.0 mol%), Bi(OTf)$_3$ (131.2 mg, 0.200 mmol, 2.00 equiv.), DME (1 mL, c = 0.1 M), and styrene (12 µL, 10 mg, 0.10 mmol, 1.0 equiv.). The vial was sealed with a septum-cap and irradiated for 6 h at 10 °C using a photoreactor equipped with a blue LED module (KT-Elektronik, “100W Power LED blau 450 nm Aquarium”, 450 nm, 30 W), cooled with two Peltier-elements (TEC1-12706). Then, the reaction mixture was concentrated to dryness. The residue was dissolved in DCM (5 mL) and washed with saturated aqueous sodium carbonate solution (5 mL). The aqueous phase was extracted with DCM (2 × 5 mL). The organic phase was dried over Na_2SO_4, filtered, and the solvent was removed under reduced pressure. The residue was purified by chromatography on silica gel eluting with CH_2Cl_2/MeOH (100/1–10/1 (v/v)) to afford 10.2 mg of cyclization product **45** as yellow oil (50% yield).

R*f* = 0.37 (DCM/MeOH = 10/1 (v/v)).

NMR Spectroscopy:

^{1}H NMR (600 MHz, CD_3CN, 23 ºC, δ): 7.36 – 7.31 (m, 4H), 7.29 – 7.23 (m, 1H), 4.58 (dd, *J* = 9.8, 4.1 Hz, 1H), 3.62 (d, *J* = 12.4 Hz, 1H), 3.54 (d, *J* = 12.3 Hz, 1H), 3.29 (dd, *J* = 13.6, 4.1 Hz, 1H), 2.86 – 2.76 (m, 2H), 2.65 (d, *J* = 13.5 Hz, 1H), 1.00 (s, 3H), 0.89 (s, 3H).

^{13}C NMR (126 MHz, CD_3CN, 23 ºC, δ): 142.8, 129.2, 128.3, 126.9, 84.8, 80.1, 61.4, 59.1, 38.5, 25.6, 24.4.

HRMS-ESI(m/z) calc’d for $C_{13}H_{19}NO$ $[M+H]^+$, 206.1542; found, 206.1539; deviation: +1.1 ppm.

N-Phenylsulfonylpiperazine 46

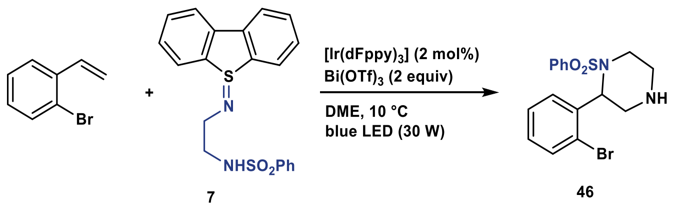

Under a nitrogen atmosphere, to a 4-mL borosilicate vial equipped with a magnetic stir bar were added sulfilimine **7** (153 mg, 0.400 mmol, 2.00 equiv.), [Ir(dFppy)$_3$] (3.0 mg, 4.0 µmol, 2.0 mol%), Bi(OTf)$_3$ (262 mg, 0.400 mmol, 2.00 equiv.), DME (1 mL, c = 0.2 M), and 2-bromostyrene (25 µL, 37 mg, 0.20 mmol, 1.0 equiv.). The vial was sealed with a septum-cap and irradiated for 6 h at 10 °C using a photoreactor equipped with a

blue LED module (KT-Elektronik, “100W Power LED blau 450 nm Aquarium”, 450 nm, 30 W), cooled with two Peltier-elements (TEC1-12706). Then, the reaction mixture was concentrated to dryness. The residue was dissolved in DCM (5 mL) and washed with saturated aqueous sodium carbonate solution (5 mL). The aqueous phase was extracted with DCM (2 × 5 mL). The organic phase was dried over Na_2SO_4, filtered, and the solvent was removed under reduced pressure. The residue was purified by chromatography on silica gel eluting with CH_2Cl_2/MeOH (50/1–10/1 (v/v)) to afford 22.0 mg of cyclization product **46** as a colorless solid (62% yield).

R*f* = 0.40 (DCM/MeOH = 10/1 (v/v)).

NMR Spectroscopy:

^{1}H NMR (500 MHz, $CDCl_3$, 23 °C, δ): 7.61 (dd, *J* = 8.4, 1.2 Hz, 2H), 7.54 – 7.46 (m, 2H), 7.39 (t, *J* = 7.9 Hz, 2H), 7.35 (dd, *J* = 7.7, 1.9 Hz, 1H), 7.10 (td, *J* = 7.5, 1.6 Hz, 1H), 7.06 (td, *J* = 7.6, 1.8 Hz, 1H), 5.05 (t, *J* = 5.0 Hz, 1H), 3.77 (ddd, *J* = 12.4, 8.4, 3.7 Hz, 1H), 3.53 (ddd, *J* = 12.8, 5.6, 4.0 Hz, 1H), 3.20 – 3.08 (m, 3H), 3.03 – 2.94 (m, 1H), 2.47 (br, 1H).

^{13}C NMR (126 MHz, $CDCl_3$, 23 °C, δ): 138.3, 133.2, 132.7, 129.6, 129.2, 129.0, 129.0, 127.6, 127.1, 122.6, 57.6, 45.4, 43.2, 29.8.

HRMS-ESI(m/z) calc’d for $C_{16}H_{18}BrN_2O_2S$ $[M+H]^+$, 381.0269; found, 381.0267; deviation: –0.6 ppm.

N-Cbz-piperazine 47

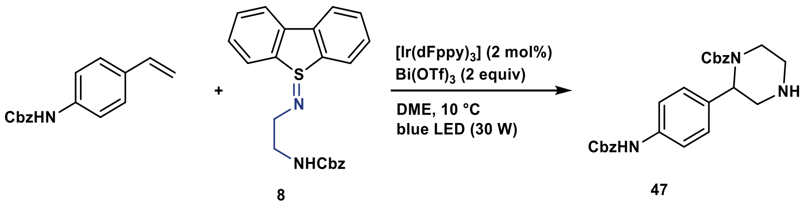

Under a nitrogen atmosphere, to a 4-mL borosilicate vial equipped with a magnetic stir bar were added benzyl (4-vinylphenyl)carbamate (50.6 mg, 0.200 mmol, 1.00 equiv.), sulfilimine **8** (150 mg, 0.400 mmol, 2.00 equiv.), [Ir(dFppy)$_3$] (3.0 mg, 4.0 µmol, 2.0 mol%), Bi(OTf)$_3$ (262 mg, 0.400 mmol, 2.00 equiv.), and DME (1 mL, c = 0.2 M). The vial was sealed with a septum-cap and irradiated for 6 h at 10 °C using a photoreactor equipped with a blue LED module (KT-Elektronik, “100W Power LED blau 450 nm Aquarium”, 450 nm, 30 W), cooled with two Peltier-elements (TEC1-12706). Then, the reaction mixture was concentrated to dryness. The residue was dissolved in DCM (5 mL) and washed with saturated aqueous sodium carbonate solution (5 mL). The aqueous phase was extracted with DCM (2 × 5 mL). The organic phase was dried over Na_2SO_4, filtered, and the solvent was removed under reduced pressure. The residue was purified by chromatography on silica gel eluting with CH_2Cl_2/MeOH (50/1–10/1 (v/v)) to afford 40.0 mg of cyclization product **47** as a colorless oil (45% yield).

R*f* = 0.35 (DCM/MeOH = 10/1 (v/v)).

NMR Spectroscopy:

^{1}H NMR (500 MHz, $CDCl_3$, 23 ºC, δ): 7.46 – 7.28 (m, 12H), 7.26 (d, *J* = 8.8 Hz, 2H), 6.70 (br, 1H), 5.26 (br, 1H), 5.20 (s, 2H), 5.18 (s, 2H), 4.00 (d, *J* = 12.7 Hz, 1H), 3.54 (d, *J* = 13.1 Hz, 1H), 3.16 (dd, *J* = 13.1, 4.3 Hz, 1H), 3.03 (td, *J* = 13.3, 12.7, 3.5 Hz, 1H), 2.99 – 2.93 (m, 1H), 2.83 (td, *J* = 12.3, 3.6 Hz, 1H).

^{13}C NMR (151 MHz, $CDCl_3$, 23 ºC, δ): 155.7, 155.7, 153.5, 137.3, 136.4, 136.1, 128.8, 128.7, 128.5, 128.5, 128.4, 128.3, 128.1, 127.6, 119.3, 67.8, 67.2, 52.5, 47.0, 44.7, 39.4.

HRMS-ESI(m/z) calc'd for $C_{26}H_{28}N_3O_4$ $[M+H]^+$, 446.2077; found, 446.2074; deviation: –0.6 ppm.

Dihydroimidazopyridinium 48

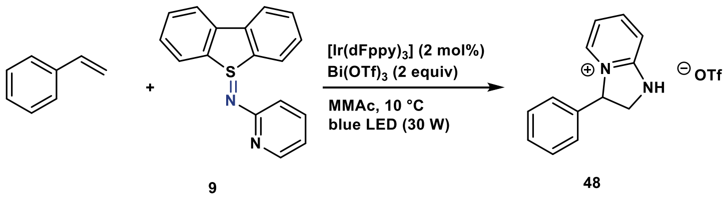

Under a nitrogen atmosphere, to a 4-mL borosilicate vial equipped with a magnetic stir bar were added sulfilimine **9** (110 mg, 0.400 mmol, 2.00 equiv.), [Ir(dFppy)$_3$] (3.0 mg, 4.0 µmol, 2.0 mol%), $Bi(OTf)_3$ (262 mg, 0.400 mmol, 2.00 equiv.), methyl methoxyacetate (MMAc) (1 mL, c = 0.2 M), and styrene (23 µL, 21 mg, 0.20 mmol, 1.0 equiv.). The vial was sealed with a septum-cap and irradiated for 6 h at 10 °C using a photoreactor equipped with a blue LED module (KT-Elektronik, "100W Power LED blau 450 nm Aquarium", 450 nm, 30 W), cooled with two Peltier-elements (TEC1-12706). Then, the reaction mixture was concentrated to dryness. The residue was purified by chromatography on silica gel eluting with CH_2Cl_2/MeOH (50/1–10/1 (v/v)) to afford 38.0 mg of cyclization product **48** as a colorless oil (55% yield).

R*f* = 0.15 (DCM/MeOH = 10/1 (v/v)).

NMR Spectroscopy:

^{1}H NMR (500 MHz, $CDCl_3$, 23 ºC, δ): 8.99 (s, 1H), 7.83 – 7.76 (m, 1H), 7.54 – 7.45 (m, 3H), 7.44 – 7.30 (m, 4H), 6.77 – 6.71 (m, 1H), 5.93 (t, *J* = 10.0 Hz, 1H), 4.52 (t, *J* = 11.0 Hz, 1H), 4.15 – 4.01 (m, 1H).

^{13}C NMR (126 MHz, $CDCl_3$, 23 ºC, δ): 156.0, 144.4, 136.0, 134.7, 130.7, 130.2, 127.8, 120.6 (d, *J* = 320.1 Hz), 114.1, 111.2, 67.1, 51.8.

^{19}F NMR (471 MHz, $CDCl_3$, 23 ºC, δ): –78.29.

HRMS-ESI(m/z) calc'd for $C_{13}H_{13}N_2$ $[M]^+$, 197.1072; found, 197.1073; deviation: +0.4 ppm.

Dihydroimidazopyridinium 49

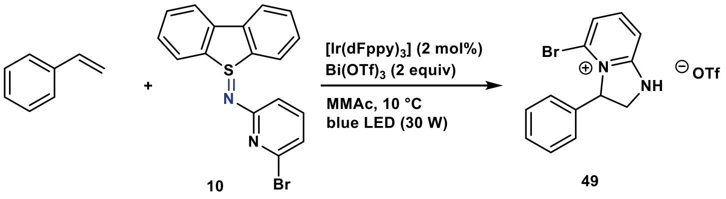

Under a nitrogen atmosphere, to a 4-mL borosilicate vial equipped with a magnetic stir bar were added sulfilimine **10** (142 mg, 0.400 mmol, 2.00 equiv.), [Ir(dFppy)$_3$] (3.0 mg, 4.0 μmol, 2.0 mol%), Bi(OTf)$_3$ (262 mg, 0.400 mmol, 2.00 equiv.), DME (1 mL, c = 0.2 M), and styrene (23 μL, 21 mg, 0.20 mmol, 1.0 equiv.). The vial was sealed with a septum-cap and irradiated for 6 h at 10 °C using a photoreactor equipped with a blue LED module (KT-Elektronik, "100W Power LED blau 450 nm Aquarium", 450 nm, 30 W), cooled with two Peltier-elements (TEC1-12706). Then, the reaction mixture was concentrated to dryness. The residue was purified by chromatography on silica gel eluting with CH_2Cl_2/MeOH (50/1–10/1 (v/v)) to afford 42.4 mg of cyclization product **49** as a colorless oil (50% yield).

R*f* = 0.15 (DCM/MeOH = 10/1 (v/v)).

NMR Spectroscopy:

^{1}H NMR (500 MHz, $CDCl_3$, 23 ºC, δ): 9.62 (br, 1H), 7.67 (dd, *J* = 9.0, 7.4 Hz, 1H), 7.47 (dd, *J* = 9.0, 1.0 Hz, 1H), 7.45 – 7.42 (m, 3H), 7.22 – 7.15 (m, 2H), 6.94 (dd, *J* = 7.4, 0.9 Hz, 1H), 6.02 (dd, *J* = 10.9, 3.9 Hz, 1H), 4.57 (dd, *J* = 11.1, 11.1 Hz, 1H), 3.97 (ddd, *J* = 11.2, 3.9, 1.2 Hz, 1H).

^{13}C NMR (151 MHz, $CDCl_3$, 23 ºC, δ): 158.3, 145.1, 136.8, 130.3, 130.1, 127.2, 126.1, 120.6 (q, *J* = 319.5 Hz), 118.4, 110.4, 68.9, 52.0.

^{19}F NMR (471 MHz, $CDCl_3$, 23 ºC, δ): –78.42.

HRMS-ESI(m/z) calc'd for $C_{13}H_{12}N_2Br$ $[M]^+$, 275.0175; found, 275.0178; deviation: +1.1 ppm.

Dihydrooxazole 50

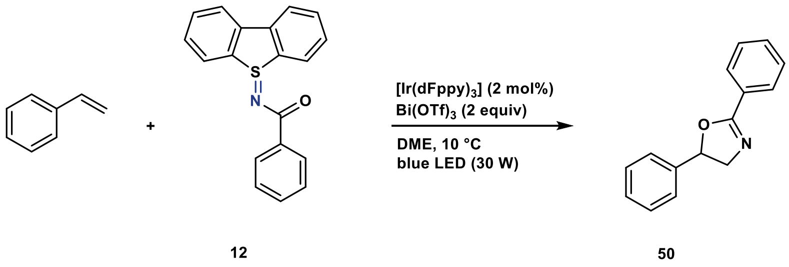

Under a nitrogen atmosphere, to a 4-mL borosilicate vial equipped with a magnetic stir bar were added sulfilimine **12** (121 mg, 0.400 mmol, 2.00 equiv.), [Ir(dFppy)$_3$] (3.0 mg, 4.0 μmol, 2.0 mol%), Bi(OTf)$_3$ (262 mg, 0.400 mmol, 2.00 equiv.), DME (1 mL, c = 0.2 M), and styrene (23 μL, 21 mg, 0.20 mmol, 1.0 equiv.). The vial was sealed with a septum-cap and irradiated for 6 h at 10 °C using a photoreactor equipped with a blue LED module (KT-Elektronik, "100W Power LED blau 450 nm Aquarium", 450 nm, 30 W), cooled with two Peltier-

elements (TEC1-12706). Then, the reaction mixture was concentrated to dryness. The residue was dissolved in EtOAc (5 mL) and washed with saturated aqueous sodium carbonate solution (5 mL). The aqueous phase was extracted with EtOAc (2 × 5 mL). The organic phase was dried over Na_2SO_4, filtered, and the solvent was removed under reduced pressure. The residue was purified by chromatography on silica gel eluting with hexane/EtOAc (10/1–4/1 (v/v)) to afford 27.6 mg of cyclization product **50** as a colorless oil (62% yield).

R*f* = 0.20 (hexane/EtOAc = 4/1 (v/v)).

NMR Spectroscopy:

^{1}H NMR (500 MHz, $CDCl_3$, 23 ºC, δ): 8.09 – 8.01 (m, 2H), 7.53 (t, *J* = 7.4 Hz, 1H), 7.45 (dd, *J* = 8.2, 6.8 Hz, 2H), 7.44 – 7.29 (m, 5H), 5.71 (dd, *J* = 10.2, 8.0 Hz, 1H), 4.51 (dd, *J* = 14.6, 10.2 Hz, 1H), 4.03 (dd, *J* = 14.7, 8.0 Hz, 1H).

^{13}C NMR (126 MHz, $CDCl_3$, 23 °C, δ): 164.2, 141.2, 131.6, 128.9, 128.5, 128.4, 128.4, 127.7, 125.9, 81.2, 63.3.

HRMS-ESI(m/z) calc'd for $C_{15}H_{14}NO$ $[M+H]^+$, 224.1071; found, 224.1070; deviation: –0.4 ppm.

Dihydrooxazole 51

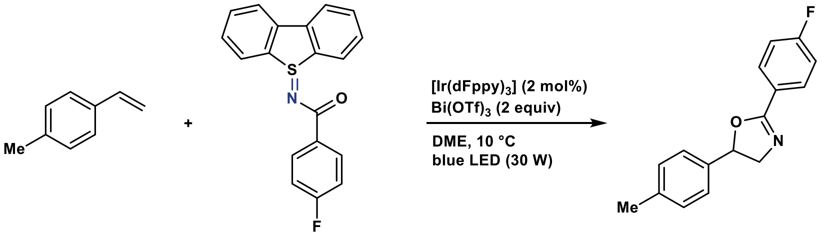

13 51

Under a nitrogen atmosphere, to a 4-mL borosilicate vial equipped with a magnetic stir bar were added sulfilimine **13** (128 mg, 0.400 mmol, 2.00 equiv.), $[Ir(dFppy)_3]$ (3.0 mg, 4.0 μmol, 2.0 mol%), $Bi(OTf)_3$ (262 mg, 0.400 mmol, 2.00 equiv.), DME (1 mL, c = 0.2 M), and 4-methylstyrene (24 μL, 26 mg, 0.20 mmol, 1.0 equiv.). The vial was sealed with a septum-cap and irradiated for 6 h at 10 °C using a photoreactor equipped with a blue LED module (KT-Elektronik, "100W Power LED blau 450 nm Aquarium", 450 nm, 30 W), cooled with two Peltier-elements (TEC1-12706). Then, the reaction mixture was concentrated to dryness. The residue was dissolved in EtOAc (5 mL) and washed with saturated aqueous sodium carbonate solution (5 mL). The aqueous phase was extracted with EtOAc (2 × 5 mL). The organic phase was dried over Na_2SO_4, filtered, and the solvent was removed under reduced pressure. The residue was purified by chromatography on silica gel eluting with hexane/EtOAc (10/1–4/1 (v/v)) to afford 29.0 mg of cyclization product **51** as a colorless oil (57% yield).

R*f* = 0.20 (hexane/EtOAc = 4/1 (v/v)).

NMR Spectroscopy:

^{1}H NMR (500 MHz, $CDCl_3$, 23 ºC, δ): 8.01 (dd, *J* = 8.7, 5.6 Hz, 2H), 7.24 (d, *J* = 7.9 Hz, 2H), 7.19 (d, *J* =

7.9 Hz, 2H), 7.11 (t, *J* = 8.6 Hz, 2H), 5.62 (dd, *J* = 10.1, 8.0 Hz, 1H), 4.44 (dd, *J* = 14.8, 10.1 Hz, 1H), 3.98 (dd, *J* = 14.7, 8.0 Hz, 1H), 2.36 (s, 3H).

^{13}C NMR (126 MHz, $CDCl_3$, 23 ºC, δ): 164.9 (d, *J* = 252.1 Hz), 163.3, 138.4, 137.9, 130.7 (d, *J* = 8.8 Hz), 129.6, 126.0, 124.1 (d, *J* = 3.0 Hz), 115.6 (d, *J* = 22.0 Hz), 81.5, 63.2, 21.3.

^{19}F NMR (471 MHz, $CDCl_3$, 23 ºC, δ): -108.10.

HRMS-EI(m/z) calc'd for $C_{16}H_{14}NOF$ $[M]^+$, 255.1050; found, 255.1054; deviation: +1.7 ppm.

Dihydroindeno[2,1-d]oxazole 52

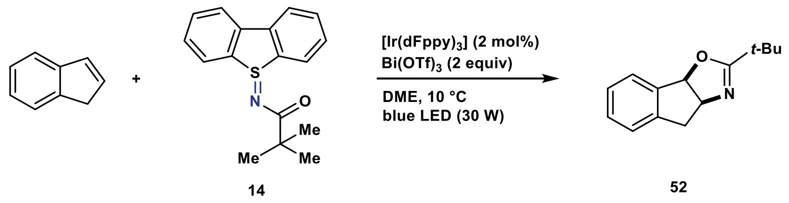

Under a nitrogen atmosphere, to a 4-mL borosilicate vial equipped with a magnetic stir bar were added sulfilimine **14** (113 mg, 0.400 mmol, 2.00 equiv.), $[Ir(dFppy)_3]$ (3.0 mg, 4.0 µmol, 2.0 mol%), $Bi(OTf)_3$ (262 mg, 0.400 mmol, 2.00 equiv.), DME (1 mL, c = 0.2 M), and indine (23 µL, 23 mg, 0.20 mmol, 1.0 equiv.). The vial was sealed with a septum-cap and irradiated for 6 h at 10 °C using a photoreactor equipped with a blue LED module (KT-Elektronik, "100W Power LED blau 450 nm Aquarium", 450 nm, 30 W), cooled with two Peltier-elements (TEC1-12706). Then, the reaction mixture was concentrated to dryness. The residue was dissolved in EtOAc (5 mL) and washed with saturated aqueous sodium carbonate solution (5 mL). The aqueous phase was extracted with EtOAc (2 × 5 mL). The organic phase was dried over Na_2SO_4, filtered, and the solvent was removed under reduced pressure. The residue was purified by chromatography on silica gel eluting with DCM/EtOAc (30/1–10/1 (v/v)) to afford 22.0 mg of cyclization product **52** as a colorless oil (51% yield, >20:1 dr).

R_f = 0.38 (DCM/EtOAc = 10/1 (v/v)).

NMR Spectroscopy:

^{1}H NMR (500 MHz, $CDCl_3$, 23 ºC, δ): 7.45 (d, *J* = 7.6 Hz, 1H), 7.35 (td, *J* = 7.2, 1.3 Hz, 1H), 7.33 – 7.26 (m, 2H), 5.98 (d, *J* = 7.8 Hz, 1H), 4.95 (td, *J* = 7.8, 2.0 Hz, 1H), 3.48 – 3.34 (m, 1H), 3.33 – 3.27 (m, 1H), 1.18 (s, 9H).

^{13}C NMR (151 MHz, CD_2Cl_2, 23 ºC, δ): 173.0, 142.5, 140.6, 129.7, 127.4, 126.4, 125.8, 86.5, 70.0, 39.7, 33.3, 27.9.

HRMS-EI(m/z) calc'd for $C_{14}H_{17}NO$ $[M]^+$, 215.1305; found, 215.1305; deviation: –0.2 ppm.

2,2-Diphenyltetrahydrobenzofuran 53

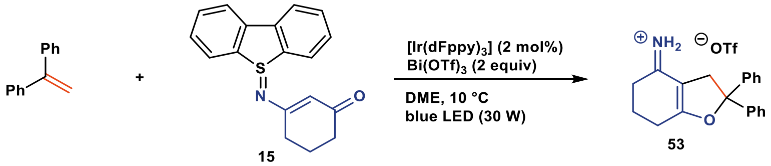

Under a nitrogen atmosphere, to a 4-mL borosilicate vial equipped with a magnetic stir bar were added sulfilimine **15** (117 mg, 0.400 mmol, 2.00 equiv.), [Ir(dFppy)$_3$] (3.0 mg, 4.0 µmol, 2.0 mol%), Bi(OTf)$_3$ (262 mg, 0.400 mmol, 2.00 equiv.), DME (1 mL, c = 0.2 M), and 1,1-diphenylethylene (36 µL, 36 mg, 0.20 mmol, 1.0 equiv.). The vial was sealed with a septum-cap and irradiated for 6 h at 10 °C using a photoreactor equipped with a blue LED module (KT-Elektronik, "100W Power LED blau 450 nm Aquarium", 450 nm, 30 W), cooled with two Peltier-elements (TEC1-12706). Then, the reaction mixture was concentrated to dryness. The residue was purified by chromatography on silica gel eluting with hexane/EtOAc (10/1–2/1 (v/v)) to afford 78.0 mg of cyclization product **53** as a colorless oil (89% yield).

R*f* = 0.20 (hexane/EtOAc = 2/1 (v/v)).

NMR Spectroscopy:

^{1}H NMR (500 MHz, CD$_3$CN, 23 ºC, δ): 8.71 (br, 2H), 7.44 – 7.37 (m, 8H), 7.41 – 7.31 (m, 2H), 3.67 (t, *J* = 1.5 Hz, 2H), 2.78 – 2.69 (m, 4H), 2.08 (p, *J* = 6.4 Hz, 2H).

^{13}C NMR (126 MHz, CD$_3$CN, 23 ºC, δ): 187.1, 178.6, 144.2, 129.8, 129.5, 126.5, 122.0 (q, *J* = 320.2 Hz), 108.3, 100.4, 40.4, 29.5, 25.0, 21.6.

^{19}F NMR (471 MHz, CD$_3$CN, 23 ºC, δ): –79.34.

HRMS-ESI(m/z) calc'd for $C_{20}H_{20}NO$ [M]$^+$, 290.1539; found, 290.1539; deviation: +0.10 ppm.

Dihydroimidazopyrimidinium 54

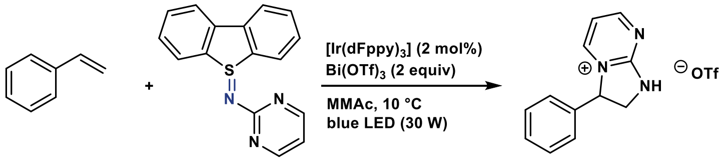

Under a nitrogen atmosphere, to a 4-mL borosilicate vial equipped with a magnetic stir bar were added sulfilimine **16** (114 mg, 0.400 mmol, 2.00 equiv.), [Ir(dFppy)$_3$] (3.0 mg, 4.0 µmol, 2.0 mol%), Bi(OTf)$_3$ (262 mg, 0.400 mmol, 2.00 equiv.), MMAc (1 mL, c = 0.2 M), and styrene (23 µL, 21 mg, 0.20 mmol, 1.0 equiv.). The vial was sealed with a septum-cap and irradiated for 6 h at 10 °C using a photoreactor equipped with a blue LED module (KT-Elektronik, "100W Power LED blau 450 nm Aquarium", 450 nm, 30 W), cooled with two Peltier-elements (TEC1-12706). Then, the reaction mixture was concentrated to dryness. The residue was purified by chromatography on silica gel eluting with CH_2Cl_2/MeOH (50/1–10/1 (v/v)) to afford 42.3 mg of cyclization product **54** as a colorless solid (61% yield).

R*f* = 0.10 (DCM/MeOH = 10/1 (v/v)).

NMR Spectroscopy:

^{1}H NMR (500 MHz, $CDCl_3$, 23 ºC, δ): 8.11 – 8.07 (m, 1H), 7.48 – 7.28 (m, 6H), 6.99 (d, *J* = 5.1 Hz, 1H), 5.64 (s, 1H), 5.20 (t, *J* = 10.2 Hz, 1H), 4.35 (dd, *J* = 14.4, 11.4 Hz, 1H), 3.84 (dd, *J* = 14.4, 8.8 Hz, 1H).

^{13}C NMR (126 MHz, $CDCl_3$, 23 ºC, δ): 165.2, 158.5, 143.3, 140.0, 129.6, 129.1, 128.8, 127.2, 65.2, 60.2.

^{19}F NMR (471 MHz, $CDCl_3$, 23 ºC, δ): –78.30.

HRMS-ESI(m/z) calc'd for $C_{12}H_{12}N_3$ $[M]^+$, 198.1026; found, 198.1026; deviation: –0.2 ppm.

Dihydroimidazo[1,3,5]triazinone 55

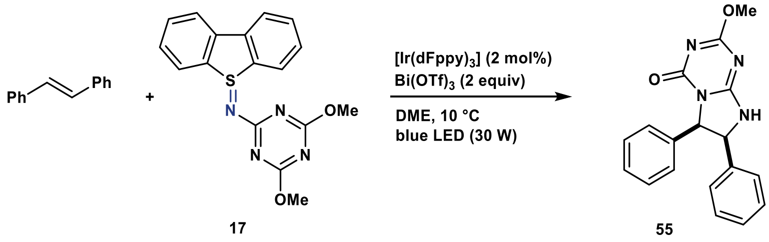

Under a nitrogen atmosphere, to a 4-mL borosilicate vial equipped with a magnetic stir bar were added sulfilimine **17** (68.0 mg, 0.200 mmol, 2.00 equiv.), $[Ir(dFppy)_3]$ (1.5 mg, 2.0 µmol, 2.0 mol%), $Bi(OTf)_3$ (131 mg, 0.200 mmol, 2.00 equiv.), DME (1 mL, c = 0.1 M), and (E)-stilbene (18.0 mg, 0.100 mmol, 1.00 equiv.). The vial was sealed with a septum-cap and irradiated for 6 h at 10 °C using a photoreactor equipped with a blue LED module (KT-Elektronik, "100W Power LED blau 450 nm Aquarium", 450 nm, 30 W), cooled with two Peltier-elements (TEC1-12706). Then, the reaction mixture was concentrated to dryness. The residue was dissolved in DCM (5 mL) and washed with saturated aqueous sodium carbonate solution (5 mL). The aqueous phase was extracted with DCM (2 × 5 mL). The organic phase was dried over Na_2SO_4, filtered, and the solvent was removed under reduced pressure. The residue was purified by chromatography on silica gel eluting with CH_2Cl_2/MeOH (50/1–10/1 (v/v)) to afford 17.8 mg of cyclization product **55** as a colorless oil (56% yield, >20:1 dr).

R*f* = 0.30 (DCM/MeOH = 10/1 (v/v)).

NMR Spectroscopy:

^{1}H NMR (500 MHz, CD_2Cl_2, 23 ºC, δ): 7.47 – 7.38 (m, 6H), 7.32 – 7.24 (m, 4H), 5.22 (d, *J* = 5.6 Hz, 1H), 4.88 (d, *J* = 5.6 Hz, 1H), 3.90 (s, 3H).

^{13}C NMR (126 MHz, d_6-DMSO, 23 ºC, δ): 172.1, 169.1, 138.1, 137.8, 133.0, 130.2, 128.9, 128.1, 126.5, 126.1, 123.2, 66.8, 64.6, 54.0.

HRMS-ESI(m/z) calc'd for $C_{18}H_{17}N_4O_2$ $[M+H]^+$, 321.1350; found, 321.1346; deviation: –1.4 ppm.

Recycling of DBT

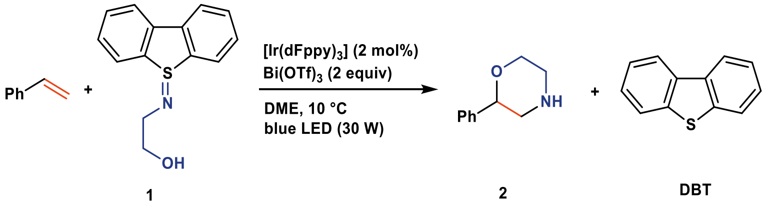

Under a nitrogen atmosphere, to a 4-mL borosilicate vial equipped with a magnetic stir bar were added sulfilimine **1** (97.2 mg, 0.400 mmol, 2.00 equiv.), [Ir(dFppy)$_3$] (3.0 mg, 4.0 µmol, 2.0 mol%), Bi(OTf)$_3$ (262 mg, 0.400 mmol, 2.00 equiv.), DME (1 mL, c = 0.2 M), and styrene (23 µL, 21 mg, 0.20 mmol, 1.0 equiv.). The vial was sealed with a septum-cap and irradiated for 6 h at 10 °C using a photoreactor equipped with a blue LED module (KT-Elektronik, "100W Power LED blau 450 nm Aquarium", 450 nm, 30 W), cooled with two Peltier-elements (TEC1-12706). Then, the reaction mixture was concentrated to dryness. The residue was dissolved in DCM (5 mL) and washed with saturated aqueous sodium carbonate solution (5 mL). The aqueous phase was extracted with DCM (2 × 5 mL). The organic phase was dried over Na_2SO_4, filtered, and the solvent was removed under reduced pressure. The residue was purified by chromatography on silica gel eluting with hexane/EtOAc (20/1 (v/v)) to afford 67.7 mg of **DBT** as a colorless solid (92% yield). The NMR spectra are in accordance with the literature[7].

Reaction of DBT=NH

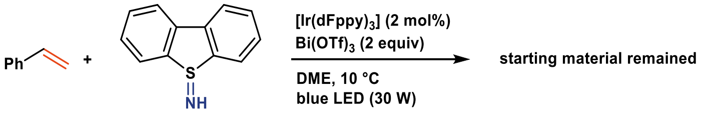

Under a nitrogen atmosphere, to a 4-mL borosilicate vial equipped with a magnetic stir bar were added dibenzothiophen-5-imine **11** (80.0 mg, 0.400 mmol, 2.00 equiv.), [Ir(dFppy)$_3$] (3.0 mg, 4.0 µmol, 2.0 mol%), Bi(OTf)$_3$ (262 mg, 0.400 mmol, 2.00 equiv.), DME (1 mL, c = 0.2 M), and styrene (23 µL, 21 mg, 0.20 mmol, 1.0 equiv.). The vial was sealed with a septum-cap and irradiated for 6 h at 10 °C using a photoreactor equipped with a blue LED module (KT-Elektronik, "100W Power LED blau 450 nm Aquarium", 450 nm, 30 W), cooled with two Peltier-elements (TEC1-12706). Then, the reaction mixture was concentrated to dryness. The crude NMR shows no consumption of starting materials.

Unsuccessful synthesis of bifunctional sulfilimines:

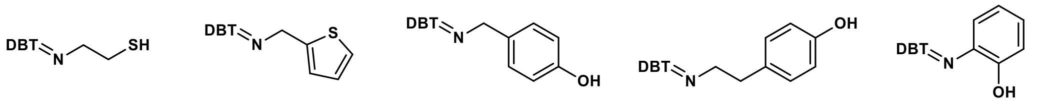

Unsuccessful sulfilimines for synthesis of N-heterocycles:

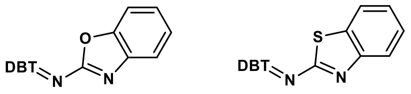

Unsuccessful alkenes for morpholine synthesis:

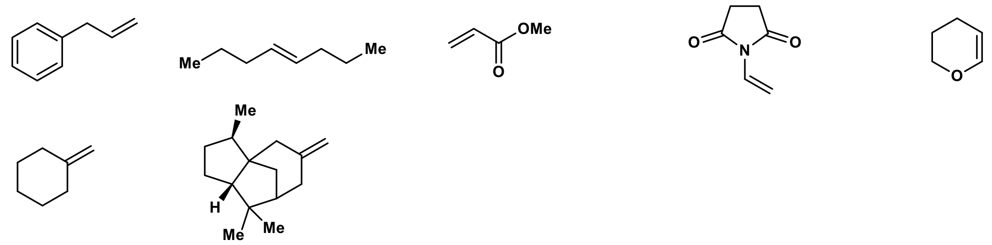

Figure 1. List of unsuccessful substrates. The cyclization reactions of sulfilimines with five-membered heteroarenes were unsuccessful (less than 10% yield), presumably due to more strained ring systems of a 5-5 ring system. Allylic amination was observed for most alkyl-substituted alkenes that feature allylic hydrogen atoms.

Mechanistic investigations for cyclization

Stern-Volmer Luminescence Quenching Studies

Visible light luminescence intensities were recorded using an Edinburgh Instruments FS5 spectrofluorometer. All luminescence measurements were recorded using a screw-top quartz cuvette (Hellma fluorescence quartz cuvette, 10 x 10 mm, 3.5 mL). All solutions of Ir(dFppy)$_3$, sulfilimines (**3**, **9**, **12**), Bi(OTf)$_3$, and styrene were prepared in DME in a nitrogen-filled glovebox. The solutions were transferred to the screw-top cuvette inside the glovebox, the cuvette was sealed, and then brought out of the glovebox for visible light luminescence measurements.

In a typical procedure, **3** (103 mg, 0.400 mmol) was dissolved and diluted to a final volume of 10 mL (c = 0.040 M) with a stock solution of Ir(dFppy)$_3$ in DME (c = 18 μM). Serial dilution of this 0.040 M **3** solution was carried out by dilution of 7 mL of the 0.040 M **3** solution to 10 mL (0.028 M) with the 18 μM stock solution of Ir(dFppy)$_3$. All subsequent solutions were prepared by dilution of 7 mL of the preceding solution to a final volume of 10 mL. All solutions were excited at 400 nm and the emission was measured from 450 to 600 nm.

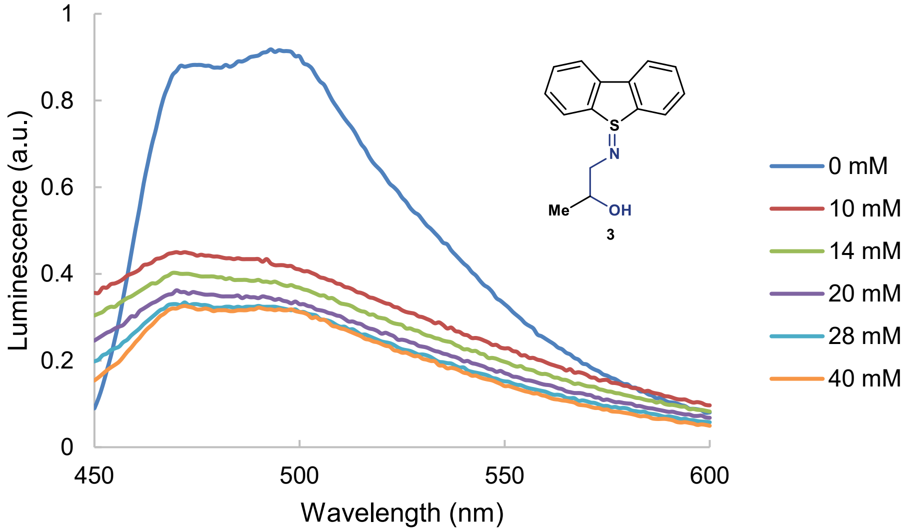

Figure 2. Emission spectra for Ir(dFppy)$_3$ luminescence quenching by sulfilimine **3** with Bi(OTf)$_3$ (1 equiv) as additive. The spectra shows an obvious shift after addition of quenching reagent, which is in agreement with a change of structure of the photocatalyst[8]. A plausible explanation is the addition of the nitrogen-centered radical to an phenyl group of ppy ligand in Ir(dFppy)$_3$ according to previous reports[8,9].

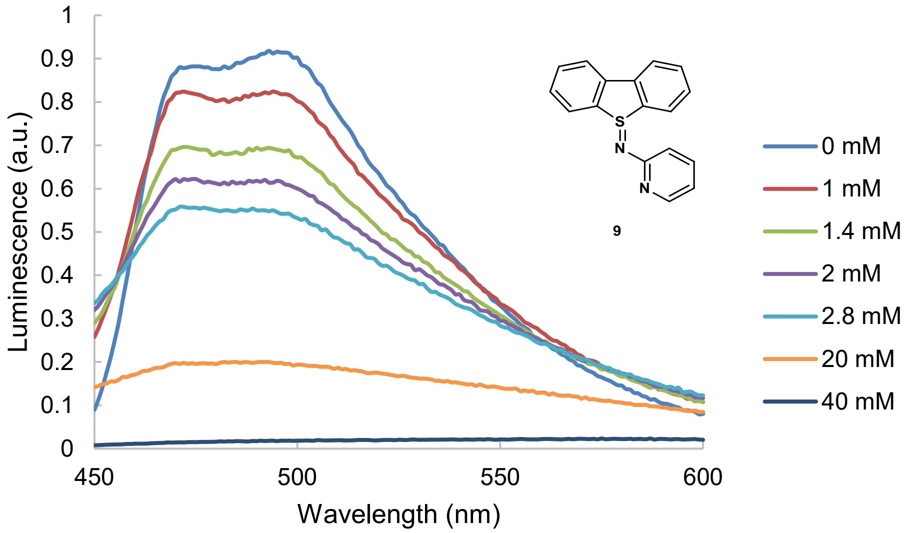

Figure 3. Emission spectra for Ir(dFppy)$_3$ luminescence quenching by sulfilimine **9** with Bi(OTf)$_3$ (1 equiv) as additive. The spectrum also shows a shift after addition of quenching reagent, which is in agreement with a change of structure of the photocatalyst. The more effective quenching when compared to sulfilimine **3** may be caused by the extended π system that will lead to easier electron transfer with photocatalyst in the excited state[10].

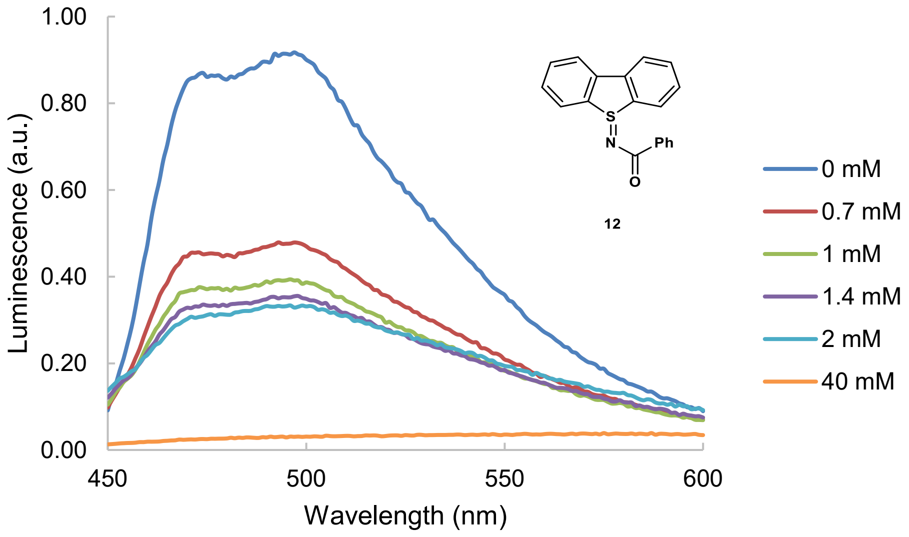

Figure 4. Emission spectra for Ir(dFppy)$_3$ luminescence quenching by sulfilimine **12** with Bi(OTf)$_3$ (1 equiv) as additive. The spectrum also shows a shift after addition of quenching reagent, which is in agreement with a change of structure. The more effective quenching when compared to sulfilimine **3** may be caused by the extended π system that will lead to easier electron transfer with photocatalyst in the excited state[10].

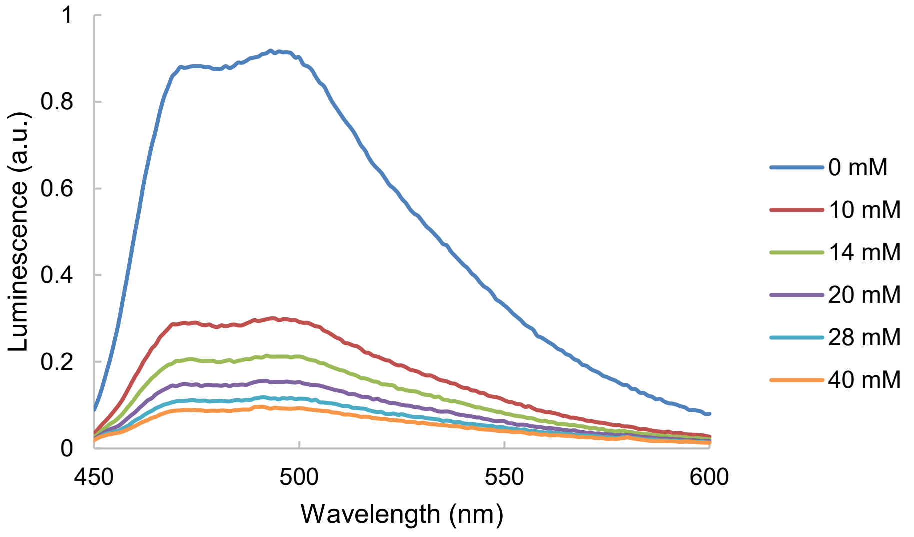

Figure 5. Emission spectra for $Ir(dFppy)_3$ luminescence quenching by $Bi(OTf)_3$.

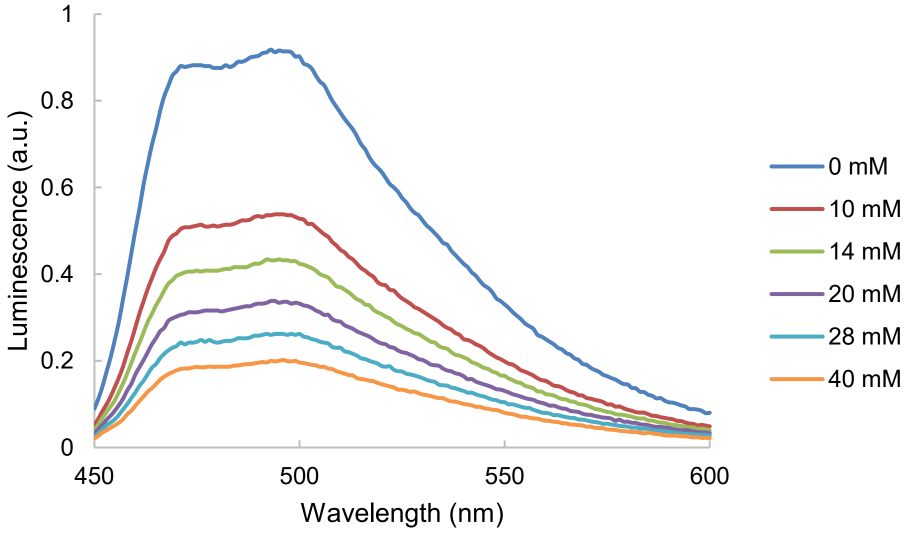

Figure 6. Emission spectra for $Ir(dFppy)_3$ luminescence quenching by styrene.

Because the structure of photocatalyst may have changed when adding substrate **3**, **9**, or **12** in combination with $Bi(OTf)_3$, the Stern-Volmer luminescence quenching studies are not reliable to determine which species is the quenching reagent in the reaction system.

Cyclic voltammograms of 1, $Bi(OTf)_3$, and a combination of 1 and $Bi(OTf)_3$

Cyclic voltammograms were recorded using an Autolab PGSTAT204 potentiostat and a Pt working electrode, a Ag/AgCl reference electrode and a Pt sheet auxiliary electrode. The voltammograms were recorded at 25 °C in 0.1 M tetrabutylammonium hexafluorophosphate in acetonitrile (3 mL) containing sulfilimine **1** (7.3 mg, 30 μmol), $Bi(OTf)_3$ (20 mg, 30 μmol). The scan rate was 100 $mV\cdot s^{-1}$.

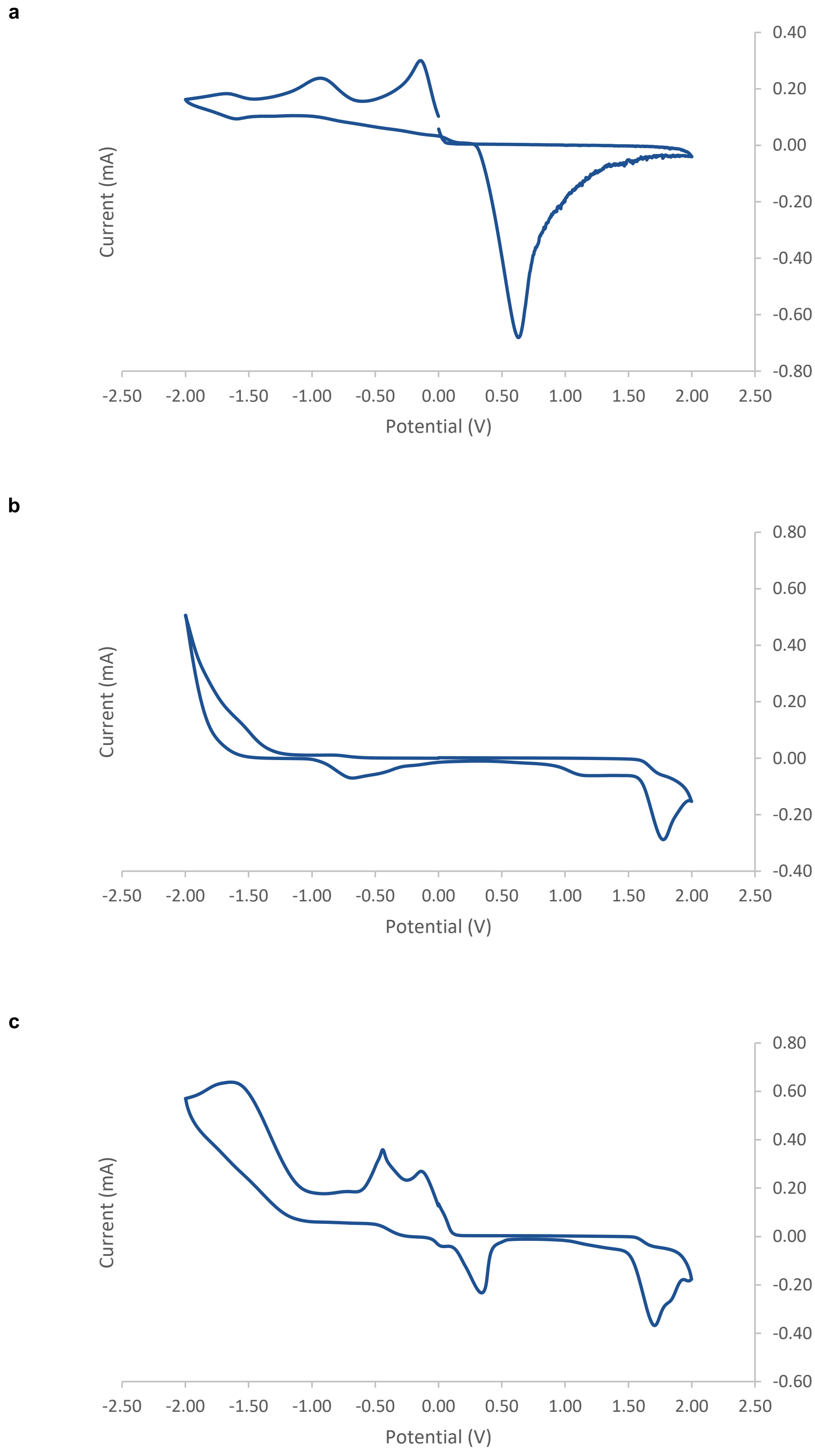

Figure 7. Cyclic voltammetry for $Bi(OTf)_3$ (**a**), sulfilimine **1** (**b**), and 1:1 mixture of $Bi(OTf)_3$ and **1** (**c**) in acetonitrile under scan rate of 100 $mV{\cdot}s^{-1}$. A new reduction peak (**c**) at E_p = –0.4 V was observed when using 1:1 mixture of $Bi(OTf)_3$ and **1**.

UV-vis spectra

All UV-vis measurements were recorded on a Shimadzu UV-vis Spectrophotometer UV-2600 with temperature controller using a quartz cuvette (10 × 10 mm, 3.5 mL) in DME as solvent. UV-vis spectra of sulfilimine **1** (2.5×10^{-5} M), $Bi(OTf)_3$ (2.5×10^{-5} M), and a mixture of **1** (2.5×10^{-5} M) and $Bi(OTf)_3$ (2.5×10^{-5} M) were recorded, respectively (Figure S7). A new peak absorbance at 326 nm was observed in the spectrum of a 1:1 mixture of $Bi(OTf)_3$ and **1** (Figure 8), which is assigned as intermediate **A**. UV-vis spectra of 1:1 mixtures of **1** and $Bi(OTf)_3$ with different concentrations were also recorded to obtain the molar attenuation coefficient (ε) of **A** at 326 nm according to Beer–Lambert law ($A = \varepsilon lc$, A is the absorbance, ε is the molar attenuation coefficient, l is the optical path length, which is 1 cm, and c is the concentration) (Figure 9).

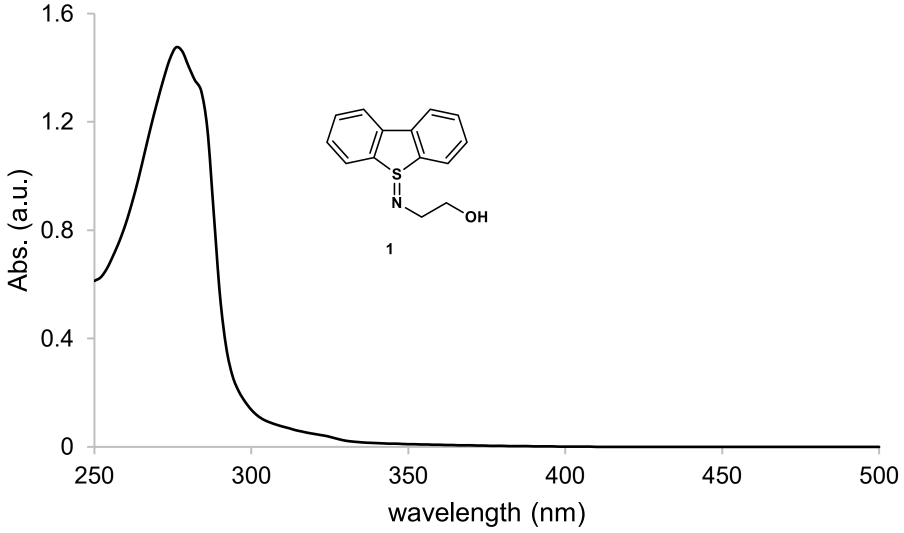

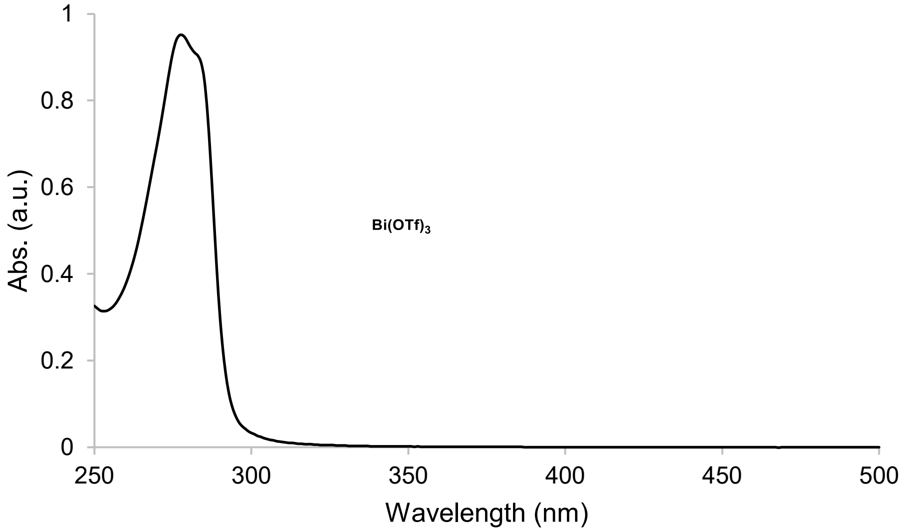

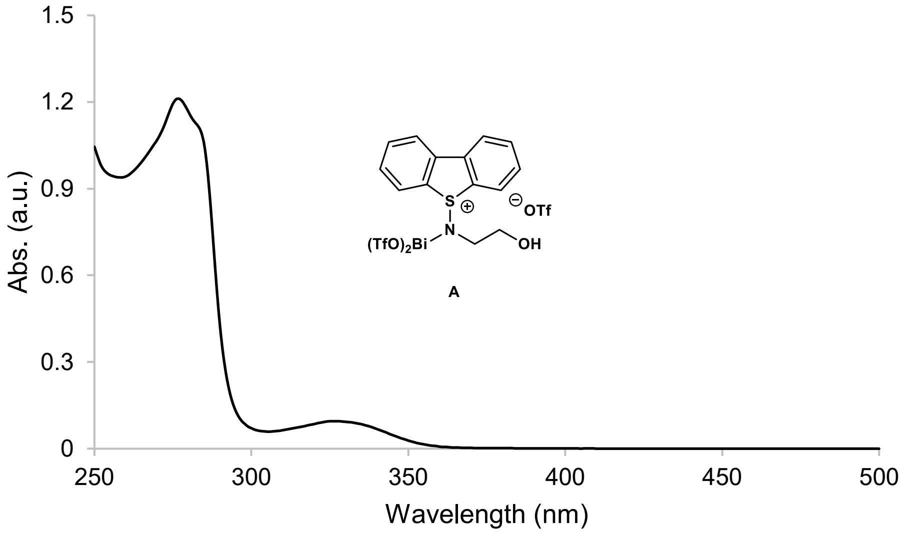

Figure 8. UV-Vis spectra of sulfilimine **1** (top), $Bi(OTf)_3$ (middle), and 1:1 mixture of $Bi(OTf)_3$ and **1** (**A**, bottom) in DME (2.5×10^{-5} M).

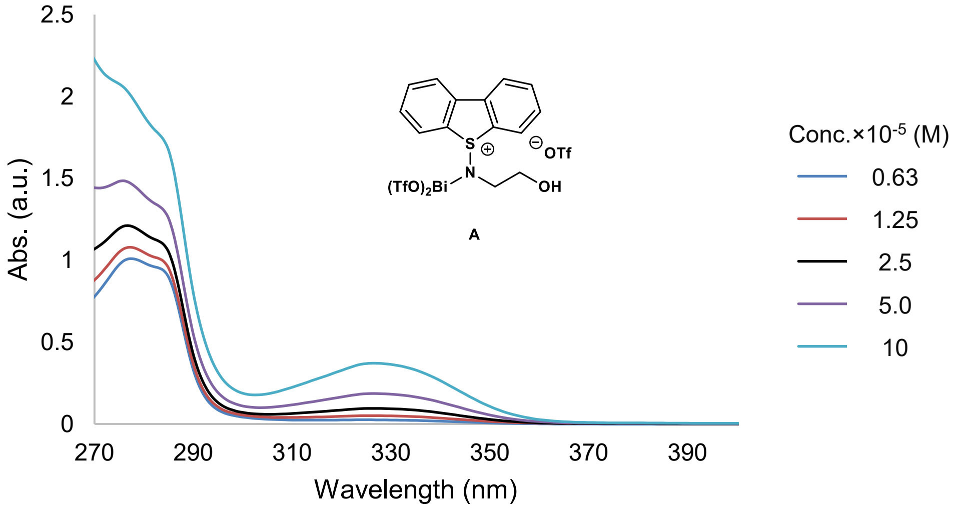

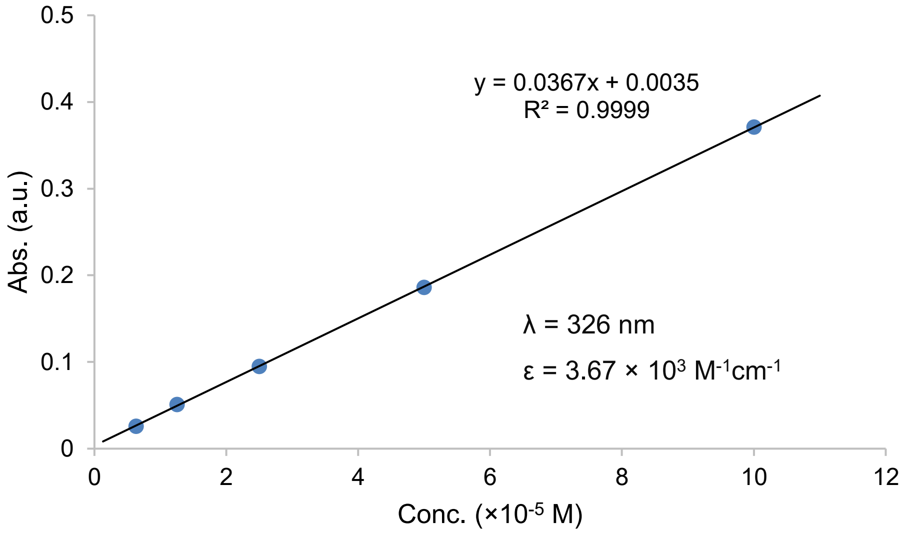

Figure 9. Linear relationship of absorbance at 326 nm of **A**. According to Beer–Lambert law, the molar attenuation coefficient (ε) of **A** at 326 nm can be read out in the graph as 3.67 × 10^3 $M^{-1}cm^{-1}$.

Radical trap and radical clock experiments

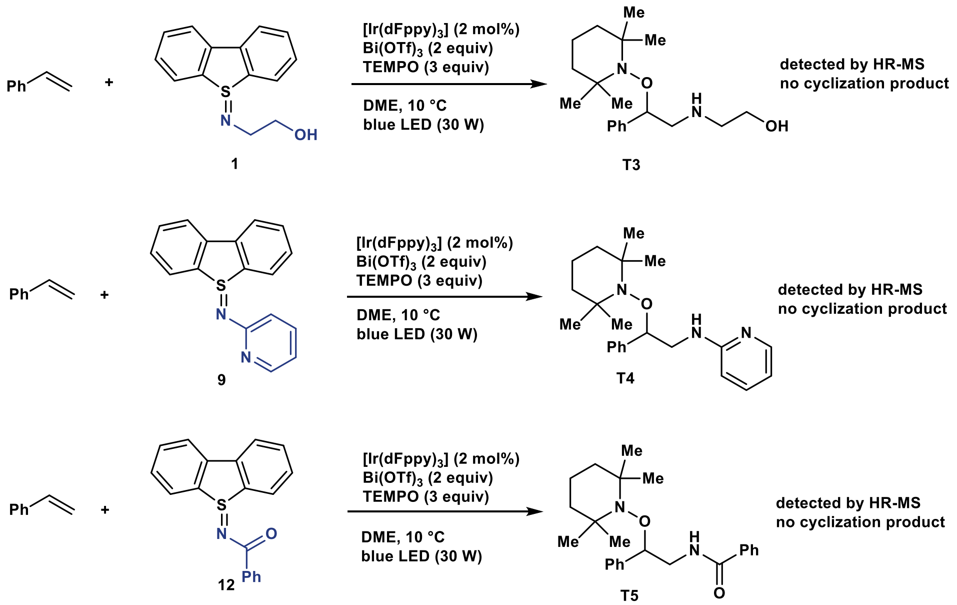

Figure 10. Trap of carbon radical intermediates by TEMPO.

Under a nitrogen atmosphere, to a 4-mL borosilicate vial equipped with a magnetic stir bar were added sulfilimine **1** (46.8 mg, 0.200 mmol, 2.00 equiv.), or **9** (55.2 mg, 0.200 mmol, 2.00 equiv.), or **12** (60.6 mg, 0.200 mmol, 2.00 equiv.), [Ir(dFppy)$_3$] (1.5 mg, 2.0 µmol, 2.0 mol%), TEMPO (47.0 mg, 0.300 mmol, 3.00 equiv.), Bi(OTf)$_3$ (130 mg, 0.200 mmol, 2.00 equiv.), DME (1 mL, c = 0.1 M), and styrene (12 µL, 10 mg, 0.10 mmol, 1.0 equiv.). The vial was sealed with a septum-cap and irradiated for 6 h at 10 °C using a photoreactor equipped with a blue LED module (KT-Elektronik, "100W Power LED blau 450 nm Aquarium", 450 nm, 30 W), cooled with two Peltier-elements (TEC1-12706). The mixture was analyzed by LRMS and HRMS (see

below).

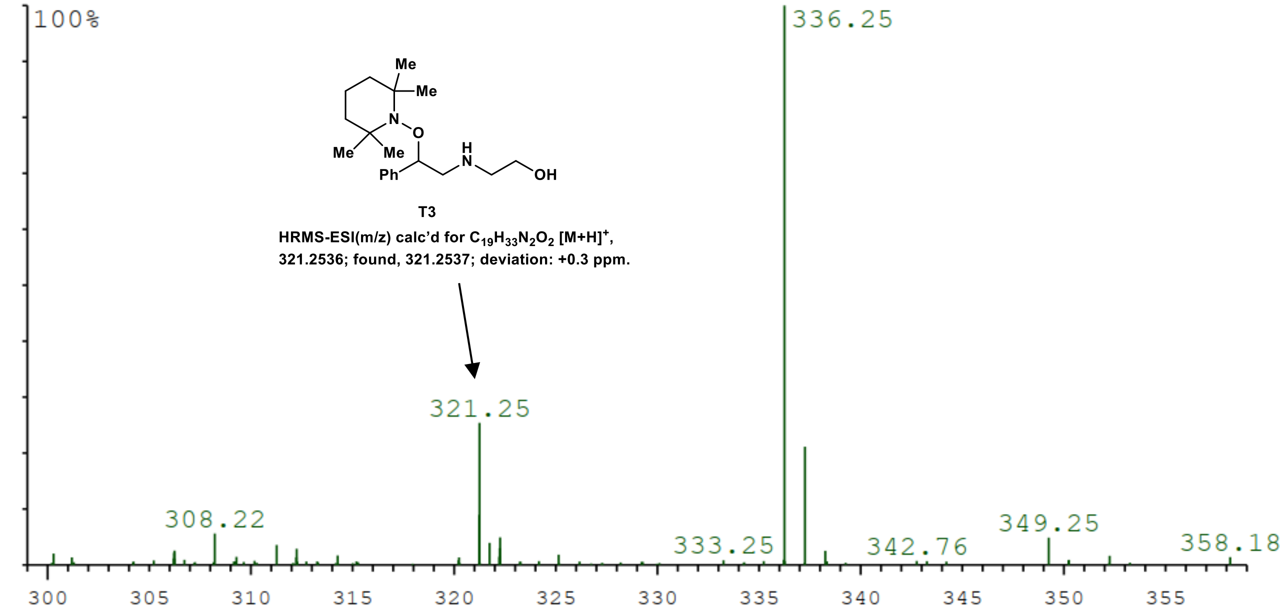
100%
336.25
Me
Me
N
O
Me
Me
H
N
Ph
OH
T3
HRMS-ESI(m/z) calc'd for $C_{19}H_{33}N_2O_2$ $[M+H]^+$, 321.2536; found, 321.2537; deviation: +0.3 ppm.
321.25
308.22
333.25
342.76
349.25
358.18
300 305 310 315 320 325 330 335 340 345 350 355

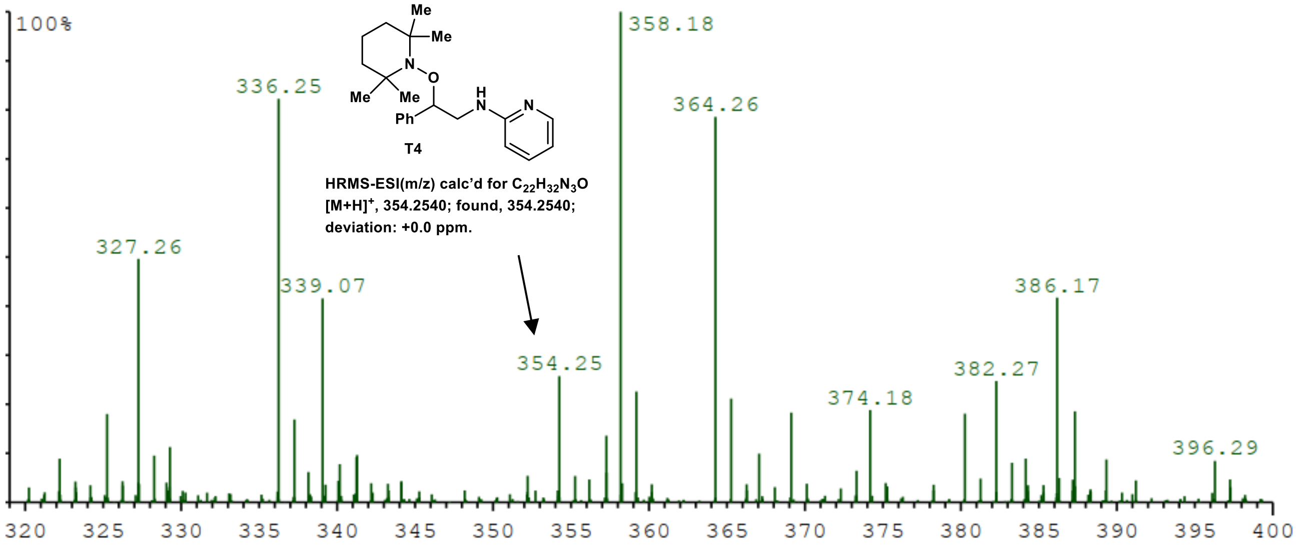
100%
358.18
Me
Me
N
O
Me
Me
H
N
N
Ph
T4
336.25
364.26
HRMS-ESI(m/z) calc'd for $C_{22}H_{32}N_3O$ $[M+H]^+$, 354.2540; found, 354.2540; deviation: +0.0 ppm.
327.26
339.07
386.17
354.25
382.27
374.18
396.29
320 325 330 335 340 345 350 355 360 365 370 375 380 385 390 395 400

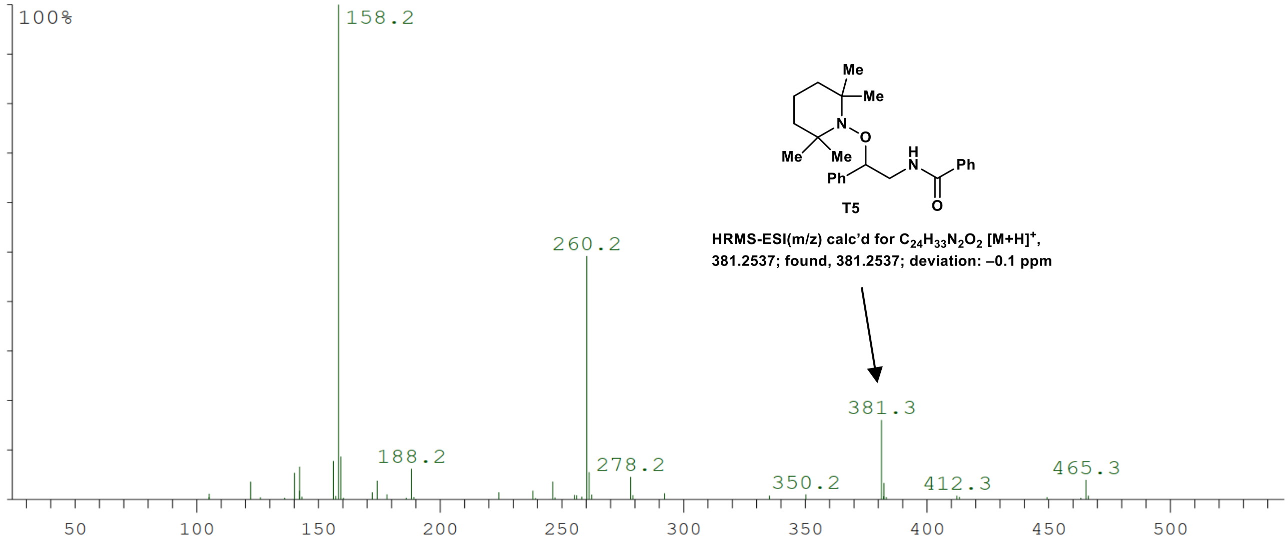

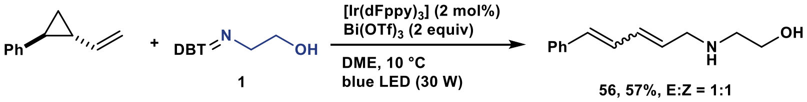

Figure 11. (1-cyclopropylvinyl)benzene as radical clock substrate.

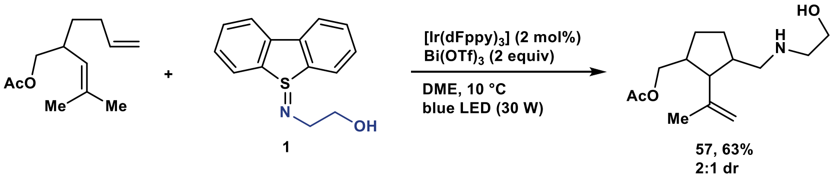

Figure 12. 1,6-Diene as radical clock substrate.

Cyclopropane ring opened product 56

Under a nitrogen atmosphere, to a 4-mL borosilicate vial equipped with a magnetic stir bar were added sulfilimine **1** (48.6 mg, 0.200 mmol, 2.00 equiv.), [Ir(dFppy)$_3$] (1.5 mg, 2.0 µmol, 2.0 mol%), Bi(OTf)$_3$ (131.2 mg, 0.200 mmol, 2.00 equiv.), DME (1 mL, c = 0.1 M), (1-(cyclopropyl)vinyl)benzene[11] (14.4 mg, 0.100 mmol, 1.00 equiv.). The vial was sealed with a septum-cap and irradiated for 6 h at 10 °C using a photoreactor equipped with a blue LED module (KT-Elektronik, "100W Power LED blau 450 nm Aquarium", 450 nm, 30 W), cooled with two Peltier-elements (TEC1-12706). Then, the reaction mixture was concentrated to dryness. The residue was dissolved in DCM (5 mL) and washed with saturated aqueous sodium carbonate solution (5 mL). The aqueous phase was extracted with DCM (2 × 5 mL). The organic phase was dried over Na_2SO_4, filtered, and the solvent was removed under reduced pressure. The residue was purified by chromatography on silica gel eluting with CH_2Cl_2/MeOH (40/1–10/1 (v/v)) to afford 11.5 mg of cyclization product **56** as yellow oil (57% yield, 1:1 E:Z).

R*f* = 0.22 (CH_2Cl_2/MeOH = 10/1 (v/v)).

NMR Spectroscopy:

^{1}H NMR (500 MHz, CD_2Cl_2, 23 ºC, δ): 7.45 – 7.15 (m, 5H), 6.80 (dd, *J* = 16.0, 11.5 Hz, 0.5H), 6.75 – 6.65 (m, 0.5H), 6.53 (d, *J* = 15.7 Hz, 0.5H), 6.41 (d, *J* = 11.7 Hz, 0.5H), 6.38 – 6.33 (m, 0.5H), 6.26 (dd, J = 11.4, 11.4 Hz, 0.5H), 6.00 – 5.83 (m, 1H), 3.67 – 3.54 (m, 2H), 3.43 – 3.28 (m, 2H), 2.82 – 2.72 (m, 2H).

^{13}C NMR (126 MHz, CD_2Cl_2, 23 ºC, δ): 137.9, 137.7, 135.1, 132.7, 132.5, 132.2, 130.0, 129.7, 129.3, 129.0, 128.9, 128.7, 128.3, 127.9, 127.3, 126.7, 61.1, 51.3, 50.9.

HRMS-ESI(m/z) calc'd for $C_{24}H_{21}NO$ $[M+H]^+$, 340.1699; found, 340.1696; deviation: –0.8 ppm.

1,6-Diene radical trap product 57

Under a nitrogen atmosphere, to a 4-mL borosilicate vial equipped with a magnetic stir bar were added sulfilimine **1** (48.6 mg, 0.200 mmol, 2.00 equiv.), $[Ir(dFppy)_3]$ (1.5 mg, 2.0 µmol, 2.0 mol%), $Bi(OTf)_3$ (131.2 mg, 0.200 mmol, 2.00 equiv.), DME (1 mL, c = 0.1 M), 2-(2-methylprop-1-en-1-yl)hex-5-en-1-yl acetate (19.6 mg, 0.100 mmol, 1.00 equiv.). The vial was sealed with a septum-cap and irradiated for 6 h at 10 °C using a photoreactor equipped with a blue LED module (KT-Elektronik, "100W Power LED blau 450 nm Aquarium", 450 nm, 30 W), cooled with two Peltier-elements (TEC1-12706). Then, the reaction mixture was concentrated to dryness. The residue was dissolved in DCM (5 mL) and washed with saturated aqueous sodium carbonate solution (5 mL). The aqueous phase was extracted with DCM (2 × 5 mL). The organic phase was dried over Na_2SO_4, filtered, and the solvent was removed under reduced pressure. The residue was purified by chromatography on silica gel eluting with CH_2Cl_2/MeOH (50/1–10/1 (v/v)) to afford 16.0 mg of cyclization product **57** as yellow oil (63% yield, 2:1 dr).

R_f = 0.20 (DCM/MeOH = 10/1 (v/v)).

NMR Spectroscopy:

^{1}H NMR (500 MHz, $CDCl_3$, 23 ºC, δ): 4.87 (s, 1H), 4.76 (s, 0.5H), 4.74 (s, 1.5H), 4.13 (dd, *J* = 10.8, 5.0 Hz, 1H), 4.09 (dd, *J* = 10.8, 5.3 Hz, 0.5H), 4.03 (td, *J* = 11.1, 6.0 Hz, 1H), 3.87 – 3.82 (m, 1.5H), 3.78 (dd, *J* = 10.8, 9.6 Hz, 0.5H), 3.65 – 3.56 (m, 4.5H), 2.96 – 2.91 (m, 0.5H), 2.85 – 2.78 (m, 0.5H), 2.76 – 2.69 (m, 3H), 2.64 (dd, *J* = 11.6, 5.0 Hz, 0.5H), 2.57 (dd, *J* = 12.1, 3.7 Hz, 0.5H), 2.50 (d, *J* = 6.7 Hz, 0.5H), 2.44 (dd, *J* = 11.5, 8.3 Hz, 0.5H), 2.41 – 2.23 (m, 6H), 2.02 (s, 3H), 2.01 (s, 1.5H), 1.94 (dd, *J* = 8.1, 5.0 Hz, 0.5H), 1.87 – 1.80 (m, 1H), 1.74 (s, 3H), 1.72 (s, 1.5H), 1.53 (dt, *J* = 7.3, 4.7 Hz, 0.5H), 1.43 – 1.33 (m, 1H).

^{13}C NMR (126 MHz, $CDCl_3$, 23 ºC, δ): 171.4, 171.4, 145.7, 144.3, 113.1, 111.6, 68.0, 67.3, 64.9, 60.7, 56.4, 54.0, 51.9, 51.3, 50.9, 50.4, 43.5, 41.9, 41.3, 40.1, 29.8, 29.3, 28.2, 27.0, 23.9, 22.1, 21.1, 18.3.

HRMS-ESI(m/z) calc'd for $C_{14}H_{26}NO_3$ $[M+H]^+$, 256.1908; found, 256.1907; deviation: –0.2 ppm.

Rationale for the formation of product 53

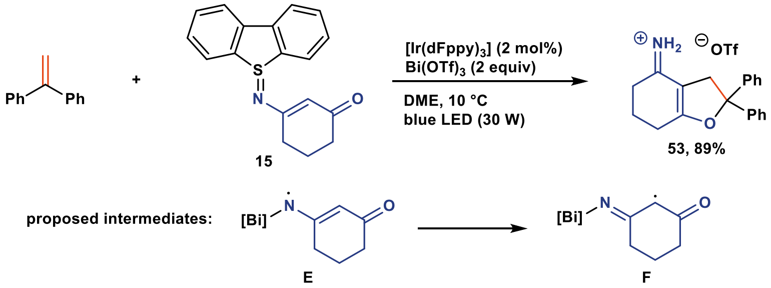

Figure 13. Cyclization reaction of diph enylethylene with sulfilimine **15**.

X-RAY CRYSTALLOGRAPHIC ANALYSIS

X-Ray Chrystallographic Data 1 (CCDC 2101016)

Experimental

Sulfilimine **1** was crystallized from a dichloromethane solution. The atoms are depicted with 50% probability ellipsoids. The crystallographic data are summarized in the following tables.

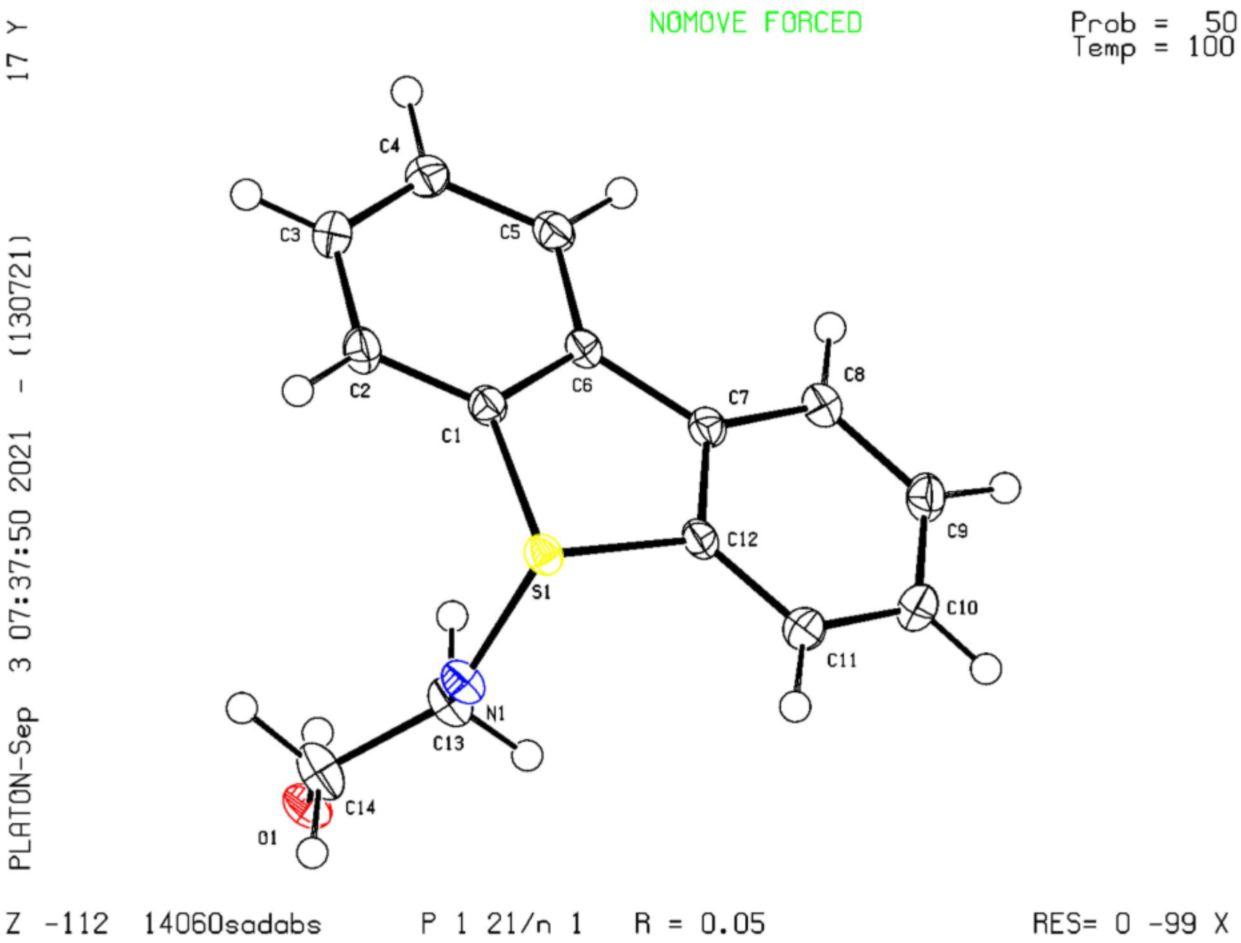

Table 7. Crystal data and structure refinement.

Identification code	14060
Empirical formula	$C_{14} H_{13} N O S$
Color	colourless
Formula weight	243.31 g · mol^{-1}
Temperature	100(2) K
Wavelength	0.71073 Å
Crystal system	MONOCLINIC

Space group	**$P2_1/n$, (no. 14)**	
Unit cell dimensions	a = 15.659(3) Å	α= 90°.
	b = 4.967(3) Å	β= 113.600(15)°.
	c = 16.651(2) Å	γ = 90°.
Volume	1186.9(7) $Å^3$	
Z	4	
Density (calculated)	1.362 Mg · m^{-3}	
Absorption coefficient	0.254 mm^{-1}	
F(000)	512 e	
Crystal size	0.14 x 0.065 x 0.04 mm^3	
θ range for data collection	2.670 to 33.185°.	
Index ranges	$-24 \le h \le 24$, $-7 \le k \le 7$, $-23 \le l \le 25$	
Reflections collected	17847	
Independent reflections	4531 [R_{int} = 0.0550]	
Reflections with $I>2\sigma(I)$	3248	
Completeness to θ = 25.242°	99.9 %	
Absorption correction	Gaussian	
Max. and min. transmission	0.99 and 0.98	
Refinement method	Full-matrix least-squares on F^2	
Data / restraints / parameters	4531 / 0 / 206	
Goodness-of-fit on F^2	1.050	
Final R indices [$I>2\sigma(I)$]	R_1 = 0.0485	wR^2 = 0.0990
R indices (all data)	R_1 = 0.0817	wR^2 = 0.1108
Largest diff. peak and hole	0.4 and -0.4 e · $Å^{-3}$	

Table 8. Bond lengths [Å] and angles [°].

S(1)-N(1)	1.5882(13)	S(1)-C(1)	1.8013(15)
S(1)-C(12)	1.7985(15)	O(1)-H(1)	0.86(2)
O(1)-C(14)	1.4180(19)	N(1)-C(13)	1.473(2)
C(1)-C(2)	1.385(2)	C(1)-C(6)	1.398(2)
C(2)-H(2)	0.965(18)	C(2)-C(3)	1.391(2)
C(3)-H(3)	0.96(2)	C(3)-C(4)	1.394(2)
C(4)-H(4)	0.989(18)	C(4)-C(5)	1.394(2)
C(5)-H(5)	0.95(2)	C(5)-C(6)	1.393(2)
C(6)-C(7)	1.470(2)	C(7)-C(8)	1.395(2)
C(7)-C(12)	1.398(2)	C(8)-H(8)	0.980(18)
C(8)-C(9)	1.396(2)	C(9)-H(9)	0.96(2)
C(9)-C(10)	1.391(2)	C(10)-H(10)	0.92(2)
C(10)-C(11)	1.393(2)	C(11)-H(11)	0.98(2)
C(11)-C(12)	1.384(2)	C(13)-H(13A)	1.009(19)
C(13)-H(13B)	1.00(2)	C(13)-C(14)	1.506(2)
C(14)-H(14A)	1.06(2)	C(14)-H(14B)	0.98(2)
N(1)-S(1)-C(1)	111.60(7)	N(1)-S(1)-C(12)	111.74(7)
C(12)-S(1)-C(1)	88.96(7)	C(14)-O(1)-H(1)	106.2(16)
C(13)-N(1)-S(1)	117.06(10)	C(2)-C(1)-S(1)	124.16(11)
C(2)-C(1)-C(6)	122.44(13)	C(6)-C(1)-S(1)	113.39(11)
C(1)-C(2)-H(2)	120.9(10)	C(1)-C(2)-C(3)	117.97(14)
C(3)-C(2)-H(2)	121.2(10)	C(2)-C(3)-H(3)	118.5(12)
C(2)-C(3)-C(4)	120.26(15)	C(4)-C(3)-H(3)	121.2(12)
C(3)-C(4)-H(4)	119.9(11)	C(5)-C(4)-C(3)	121.50(14)
C(5)-C(4)-H(4)	118.6(11)	C(4)-C(5)-H(5)	123.3(12)
C(6)-C(5)-C(4)	118.53(14)	C(6)-C(5)-H(5)	118.2(12)
C(1)-C(6)-C(7)	112.02(12)	C(5)-C(6)-C(1)	119.29(14)
C(5)-C(6)-C(7)	128.67(14)	C(8)-C(7)-C(6)	128.41(13)
C(8)-C(7)-C(12)	119.40(14)	C(12)-C(7)-C(6)	112.16(13)
C(7)-C(8)-H(8)	121.5(11)	C(7)-C(8)-C(9)	118.49(14)
C(9)-C(8)-H(8)	120.0(11)	C(8)-C(9)-H(9)	120.9(11)
C(10)-C(9)-C(8)	121.29(15)	C(10)-C(9)-H(9)	117.8(11)

C(9)-C(10)-H(10)	120.2(13)	C(9)-C(10)-C(11)	120.56(15)
C(11)-C(10)-H(10)	119.2(13)	C(10)-C(11)-H(11)	122.4(12)
C(12)-C(11)-C(10)	117.83(14)	C(12)-C(11)-H(11)	119.8(12)
C(7)-C(12)-S(1)	113.43(11)	C(11)-C(12)-S(1)	124.15(12)
C(11)-C(12)-C(7)	122.41(14)	N(1)-C(13)-H(13A)	111.4(10)
N(1)-C(13)-H(13B)	112.3(11)	N(1)-C(13)-C(14)	109.48(13)
H(13A)-C(13)-H(13B)	105.2(16)	C(14)-C(13)-H(13A)	110.1(11)
C(14)-C(13)-H(13B)	108.3(12)	O(1)-C(14)-C(13)	112.70(13)
O(1)-C(14)-H(14A)	110.6(12)	O(1)-C(14)-H(14B)	108.5(12)
C(13)-C(14)-H(14A)	108.2(12)	C(13)-C(14)-H(14B)	110.5(13)
H(14A)-C(14)-H(14B)	106.3(17)		

SPECTROSCOPIC DATA

^{1}H NMR of sulfilimine 1

CDCl$_3$, 23 ºC

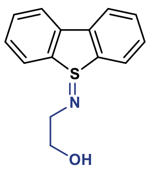

1

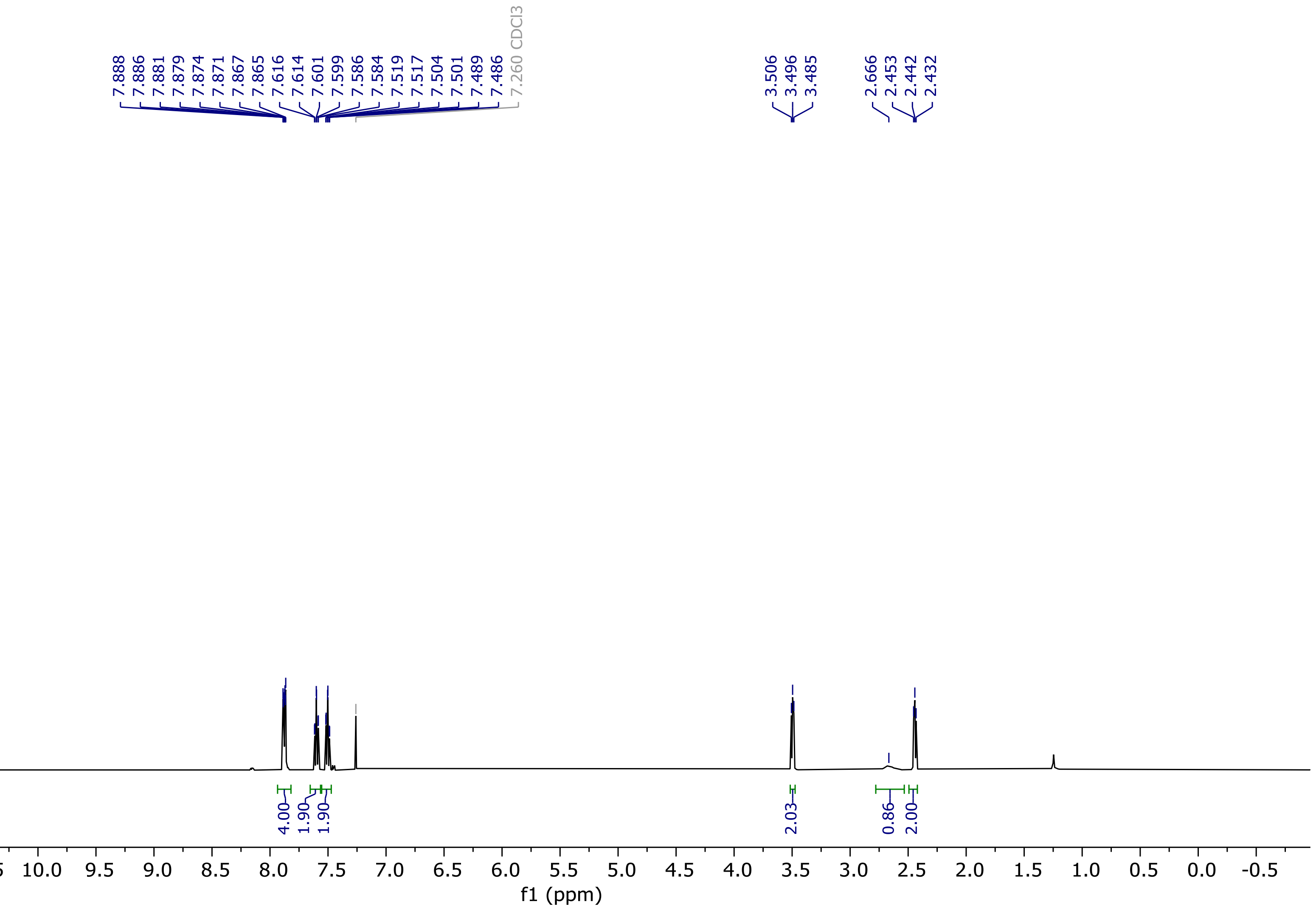

^{13}C NMR of sulfilimine 1

$CDCl_3$, 23 ºC

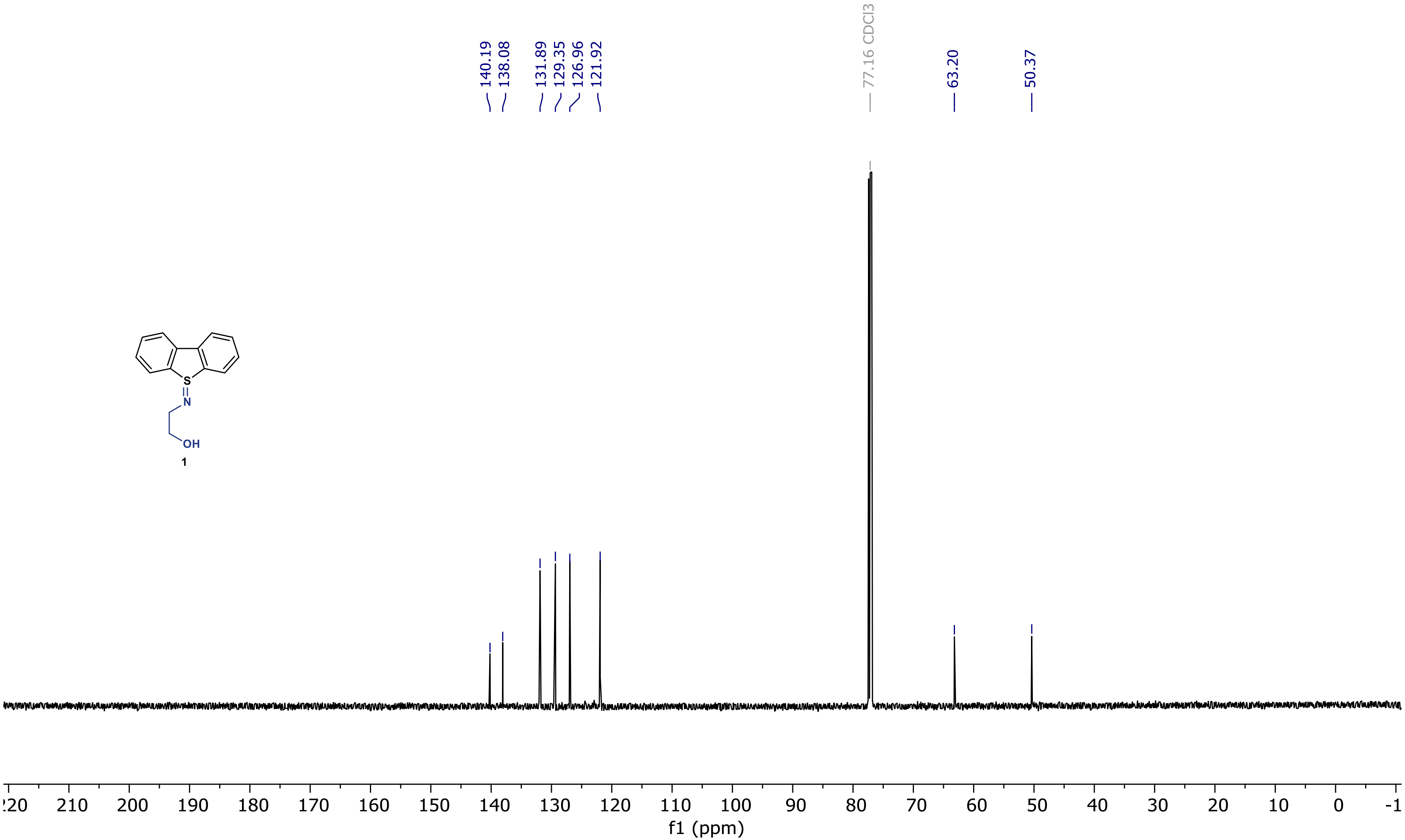

^{1}H NMR of 2-phenylmorpholine 2

$CDCl_3$, 23 ºC

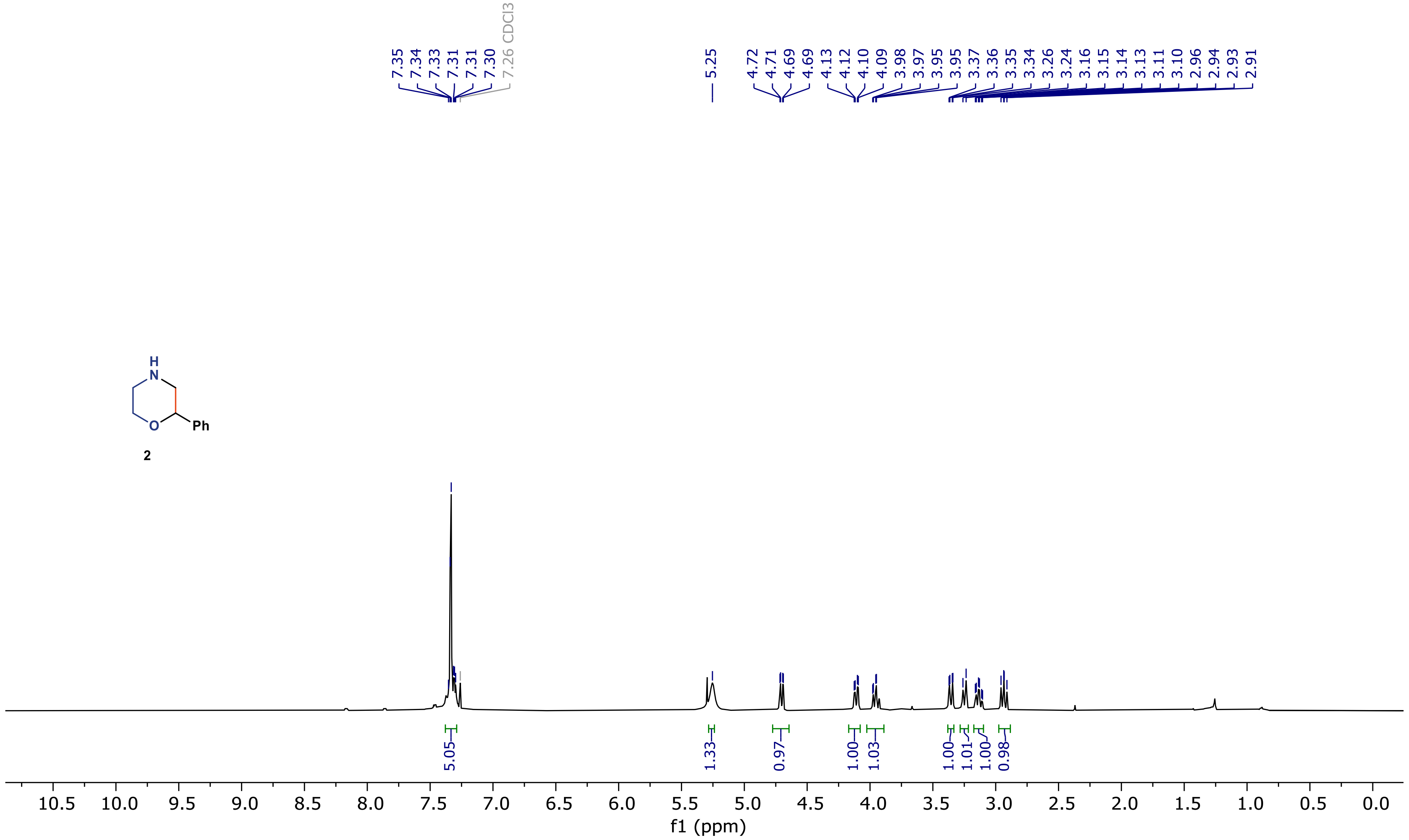

^{13}C NMR of 2-phenylmorpholine 2

$CDCl_3$, 23 ºC

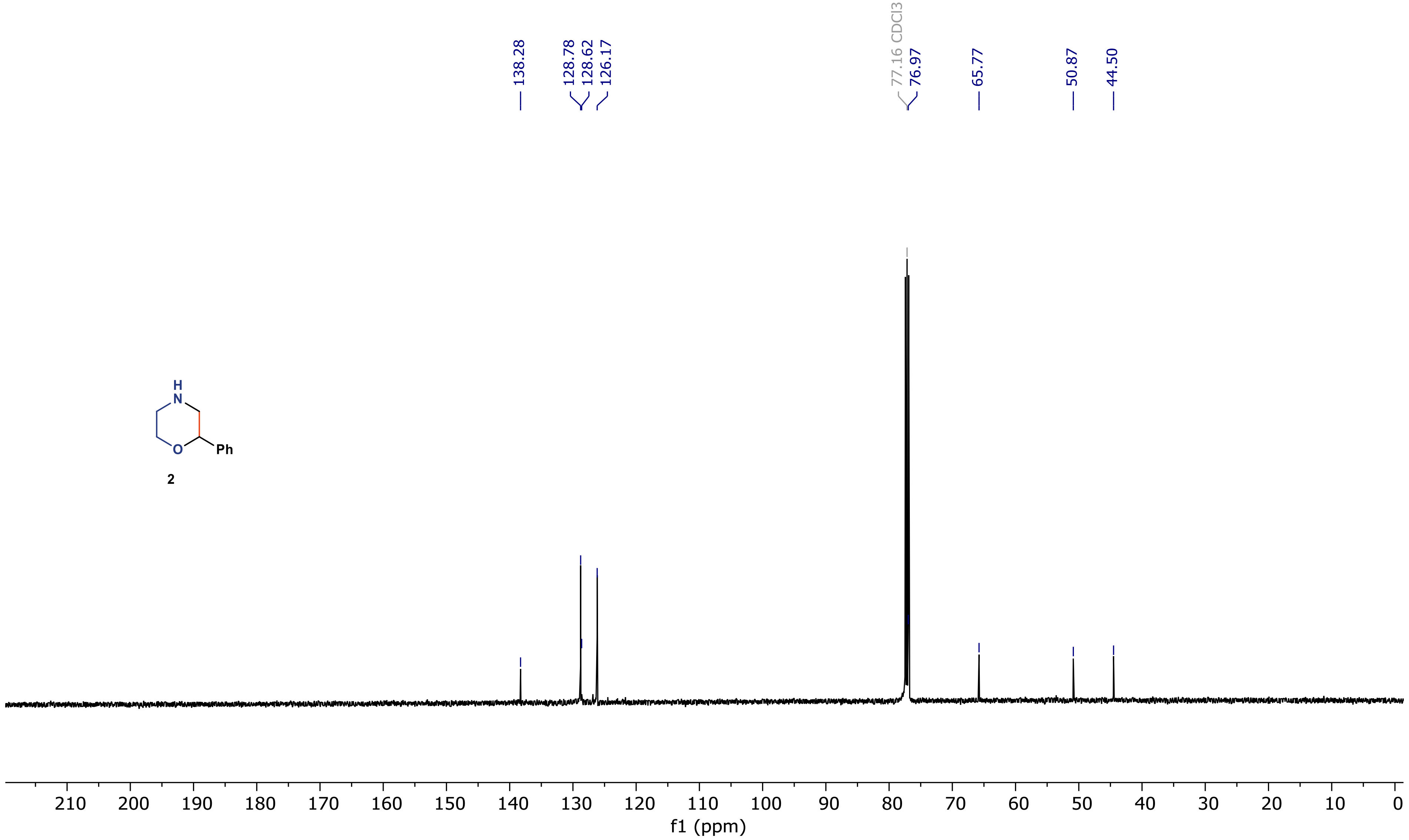

^{1}H NMR of sulfilimine 3

$CDCl_3$, 23 ºC

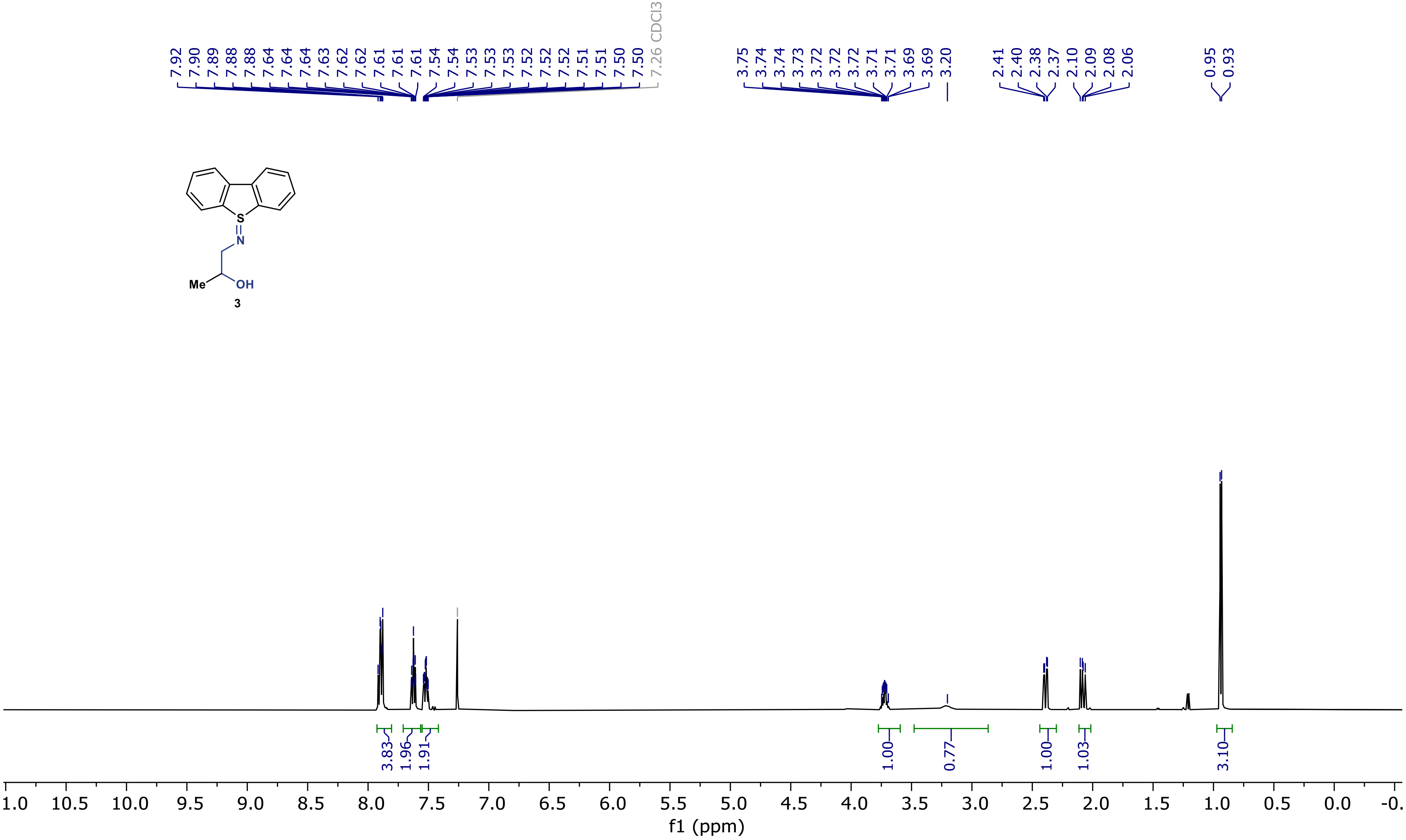

^{13}C NMR of sulfilimine 3

$CDCl_3$, 23 ºC

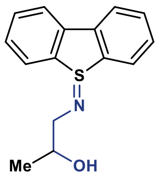

3

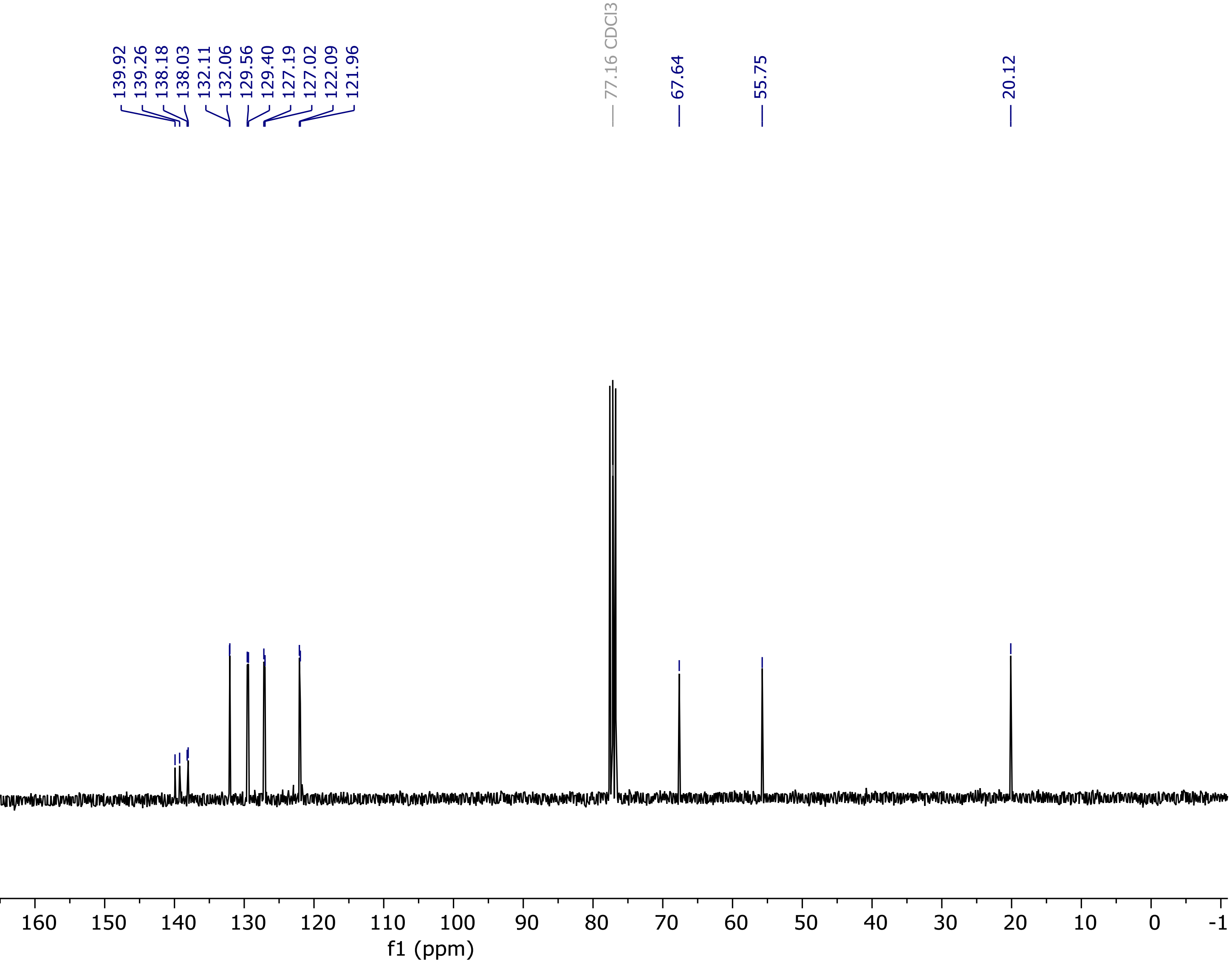

^{1}H NMR of sulfilimine 4

CD_3OD, 23 ºC

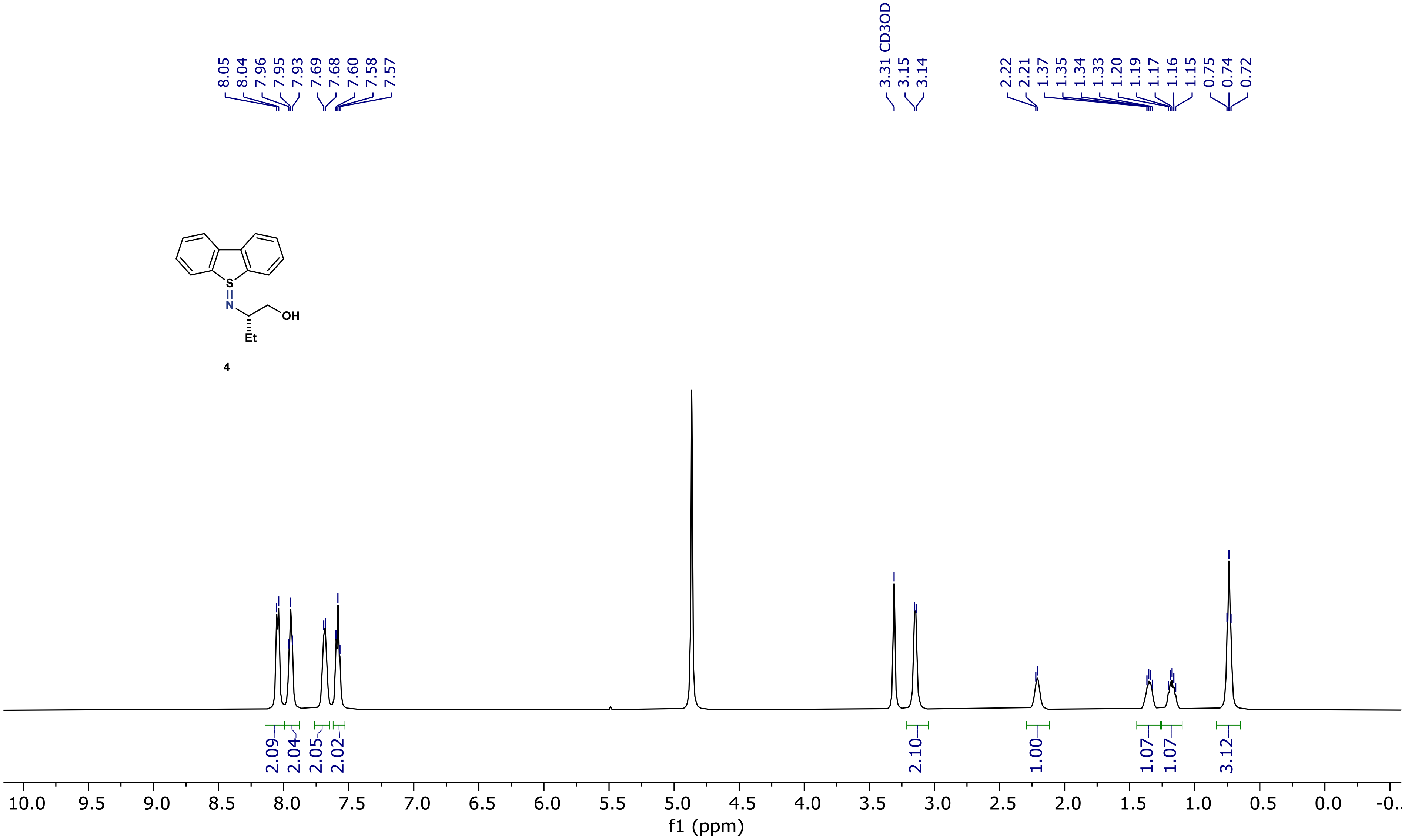

^{13}C NMR of sulfilimine 4

CD_3OD, 23 ºC

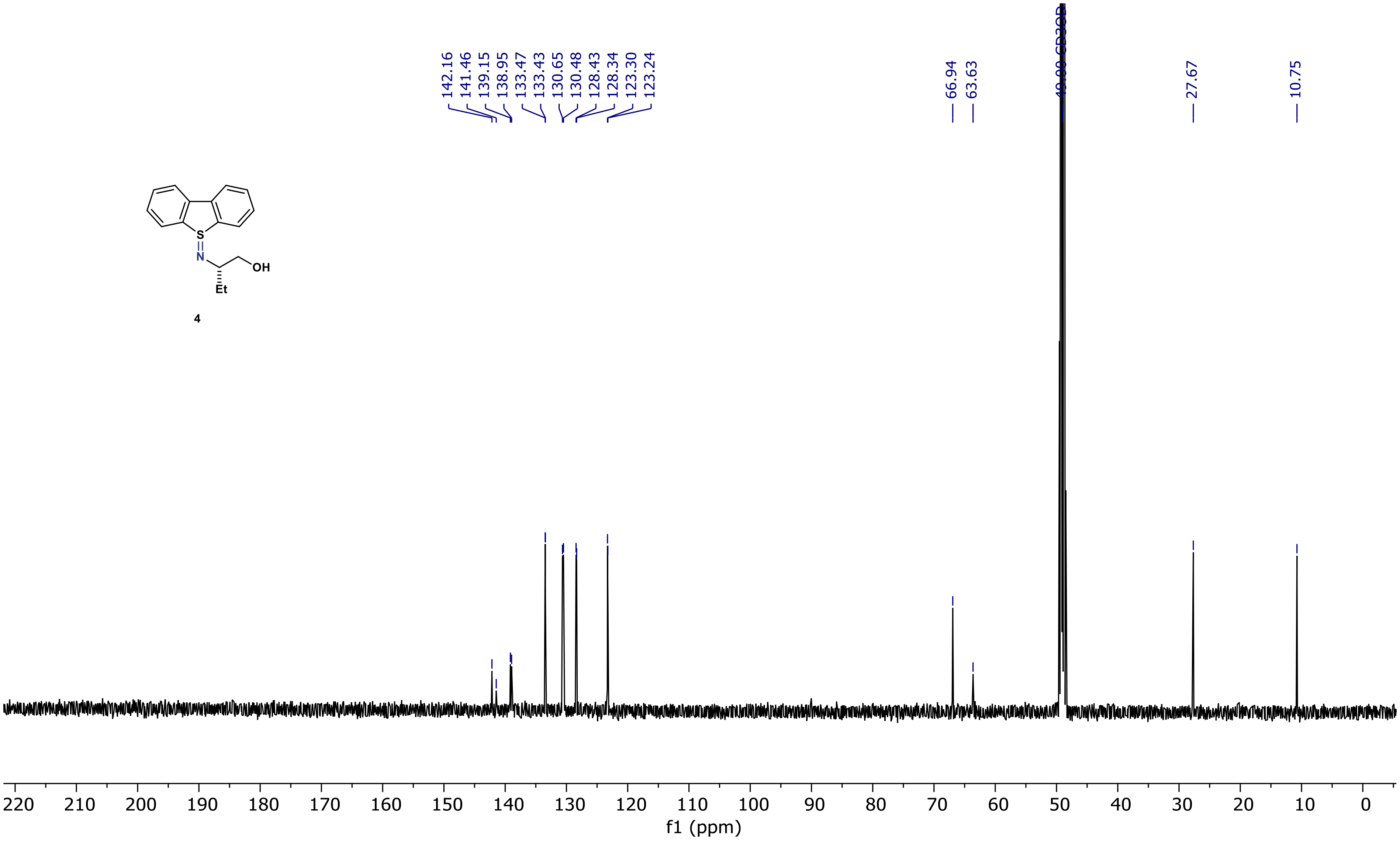

4

^{1}H NMR of sulfilimine 5

CD_2Cl_2, 23 ºC

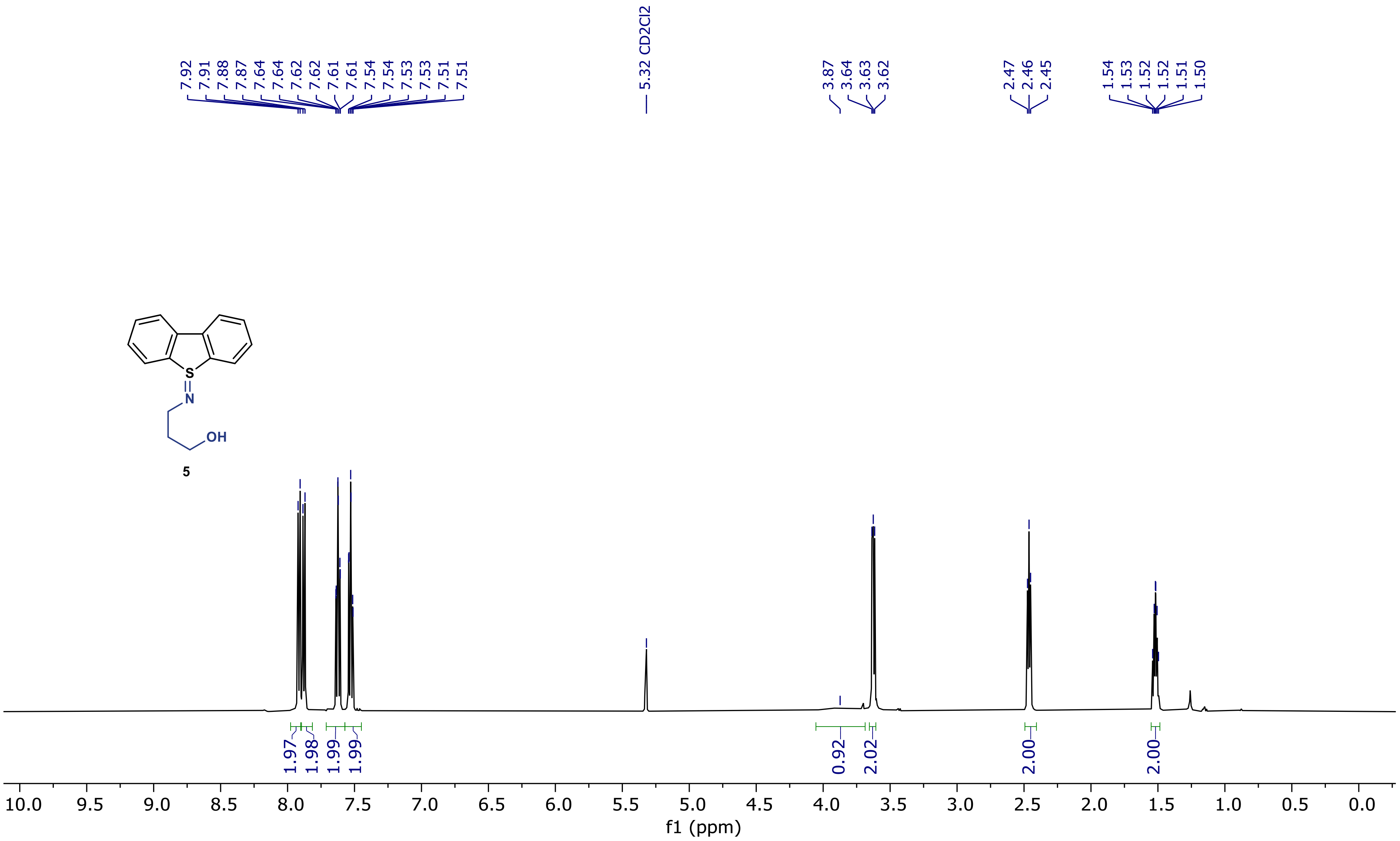

^{13}C NMR of sulfilimine 5

CD_2Cl_2, 23 ºC

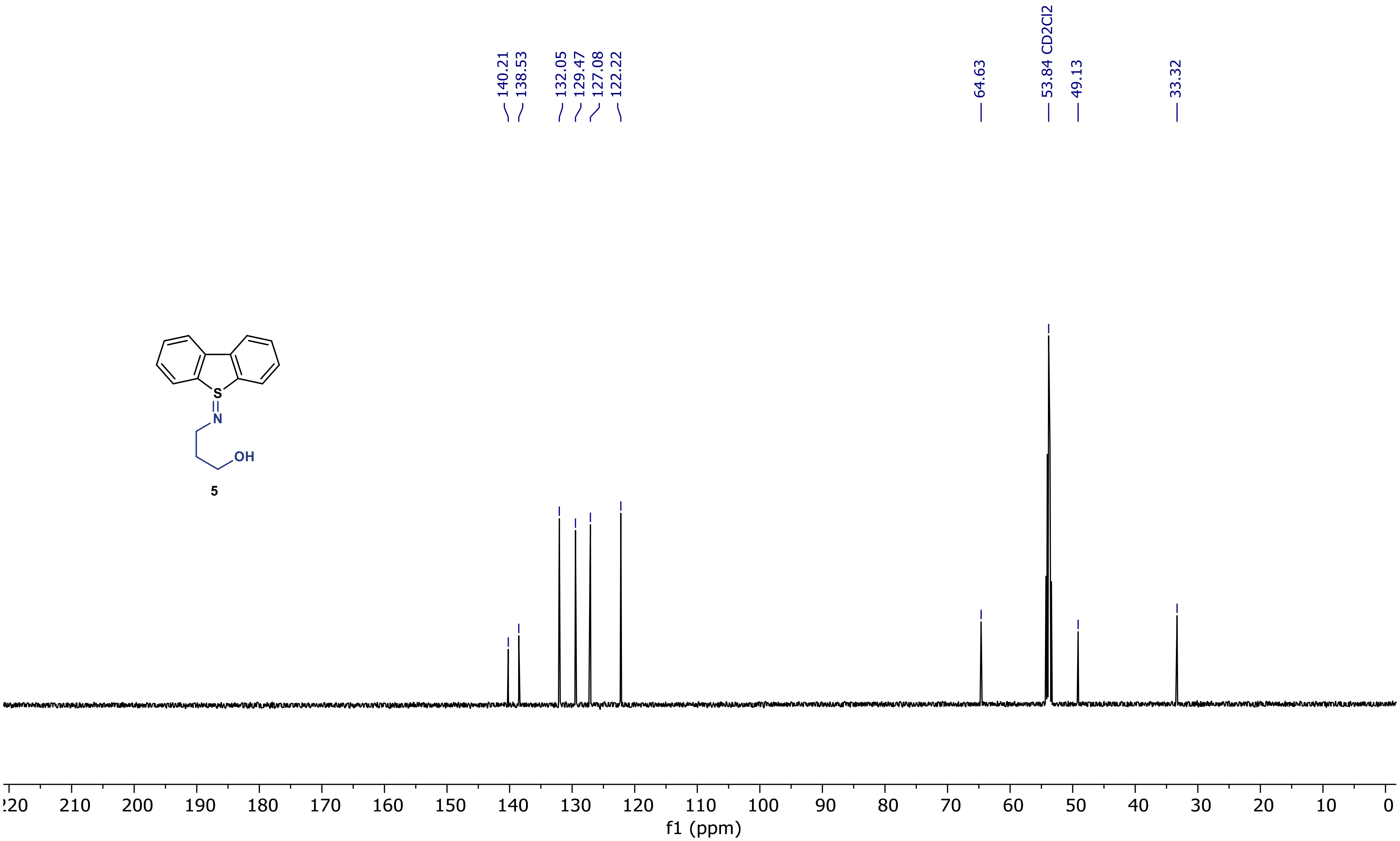

^{1}H NMR of sulfilimine 6

CDCl3, 23 ºC

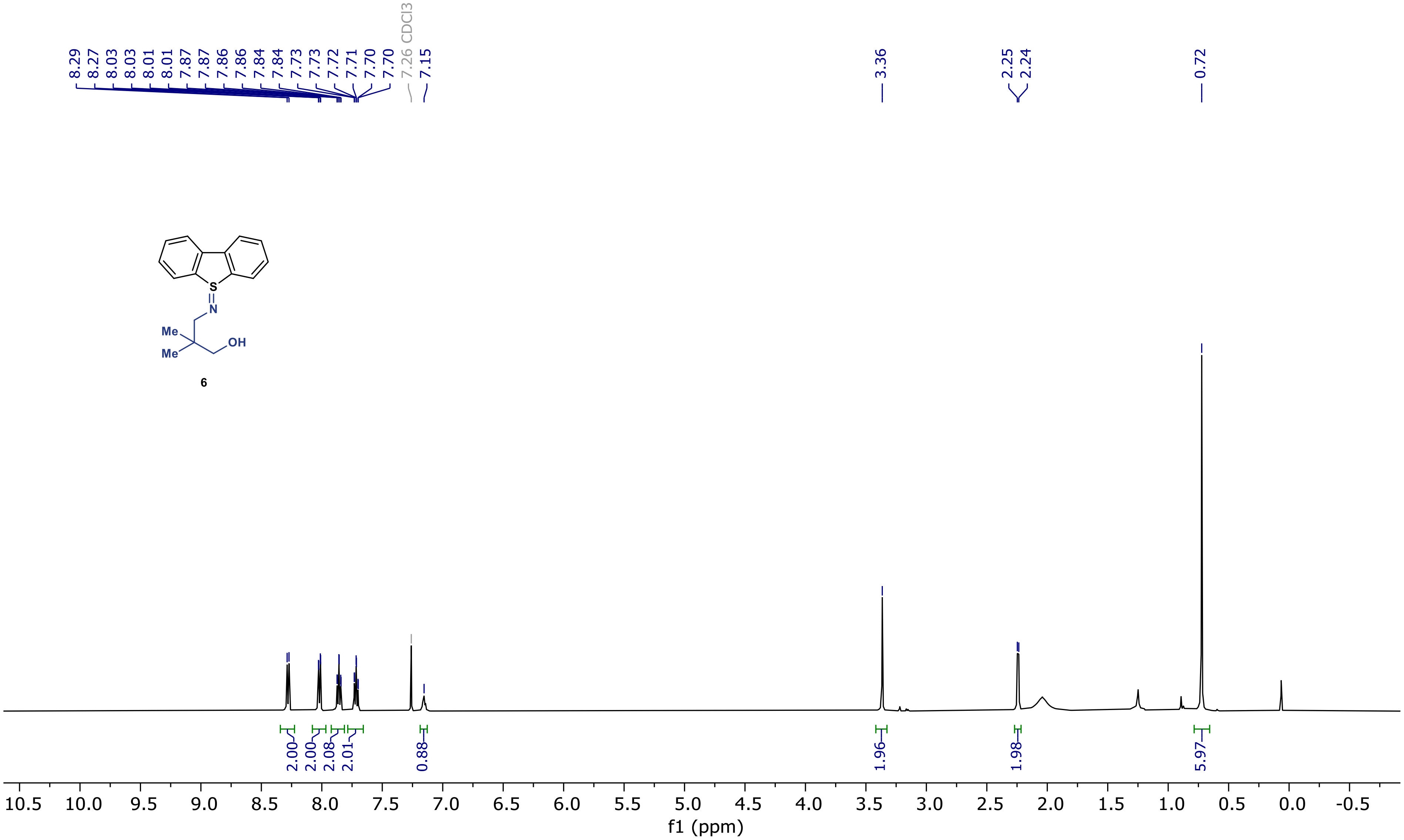

6

^{13}C NMR of sulfilimine 6

CDCl3, 23 ºC

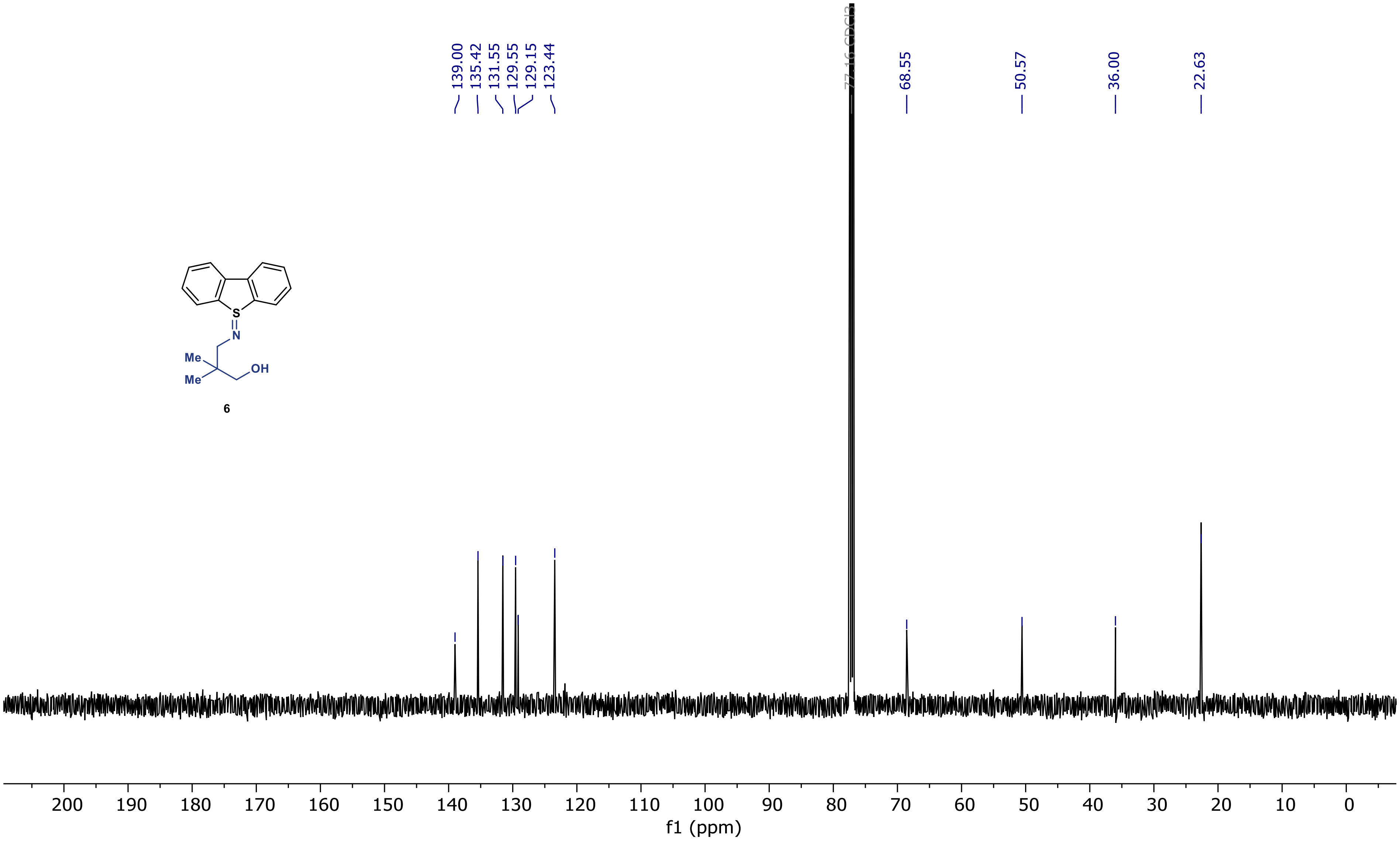

^{1}H NMR of sulfilimine 7

CDCl$_3$, 23 ºC

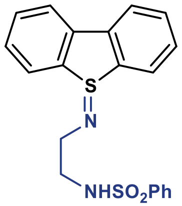

7

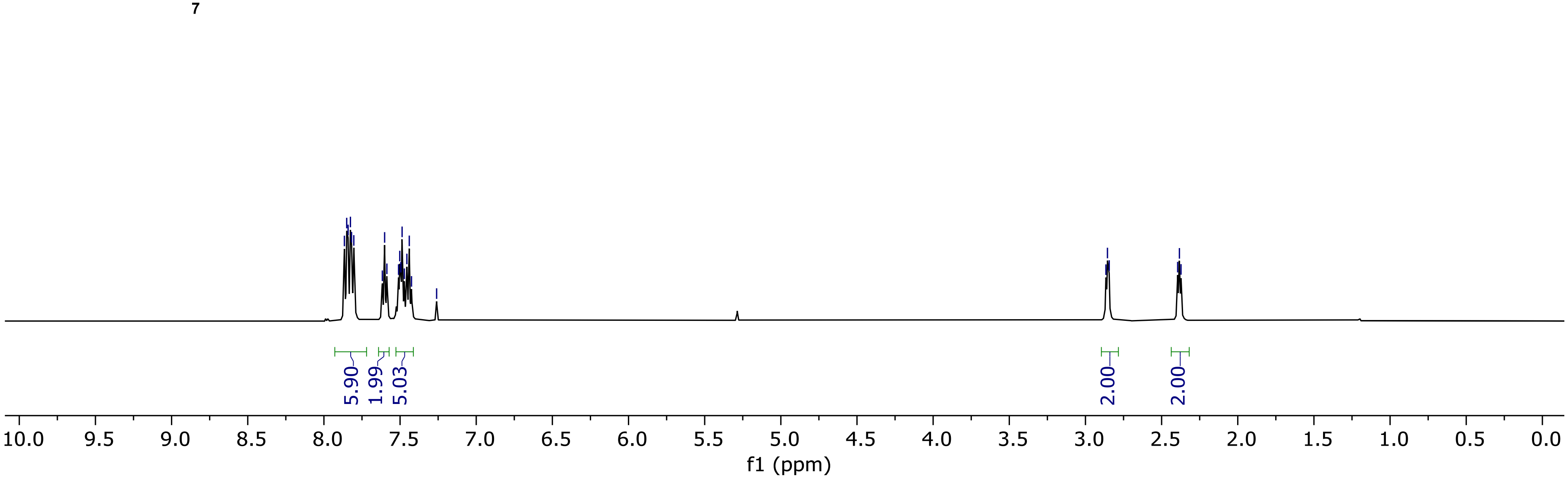

^{13}C NMR of sulfilimine 7

CDCl₃, 23 ºC

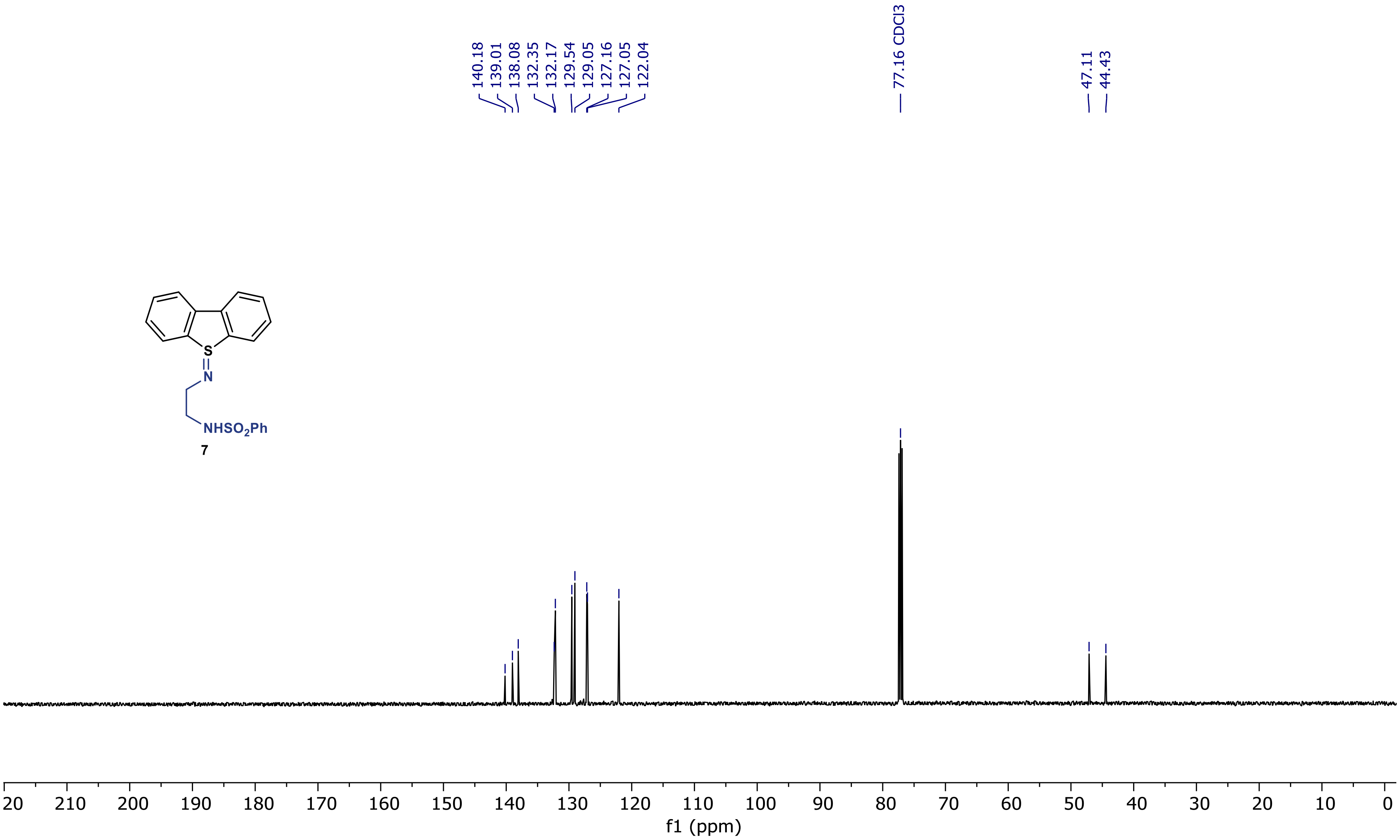

7

^{1}H NMR of sulfilimine 8

CDCl3, 23 ºC

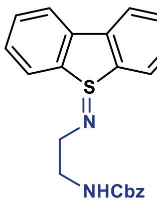

8

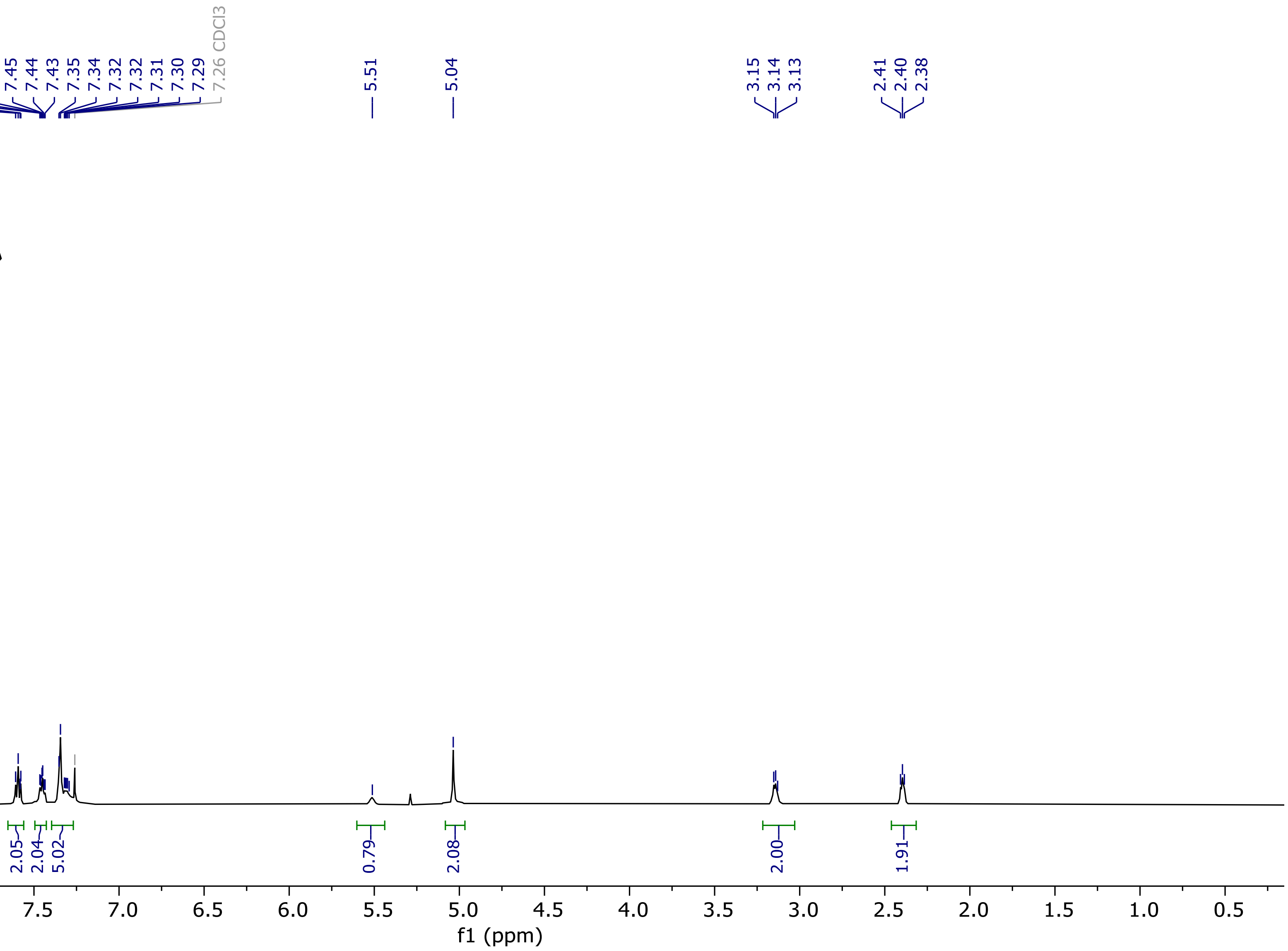

^{13}C NMR of sulfilimine 8

CDCl$_3$, 23 ºC

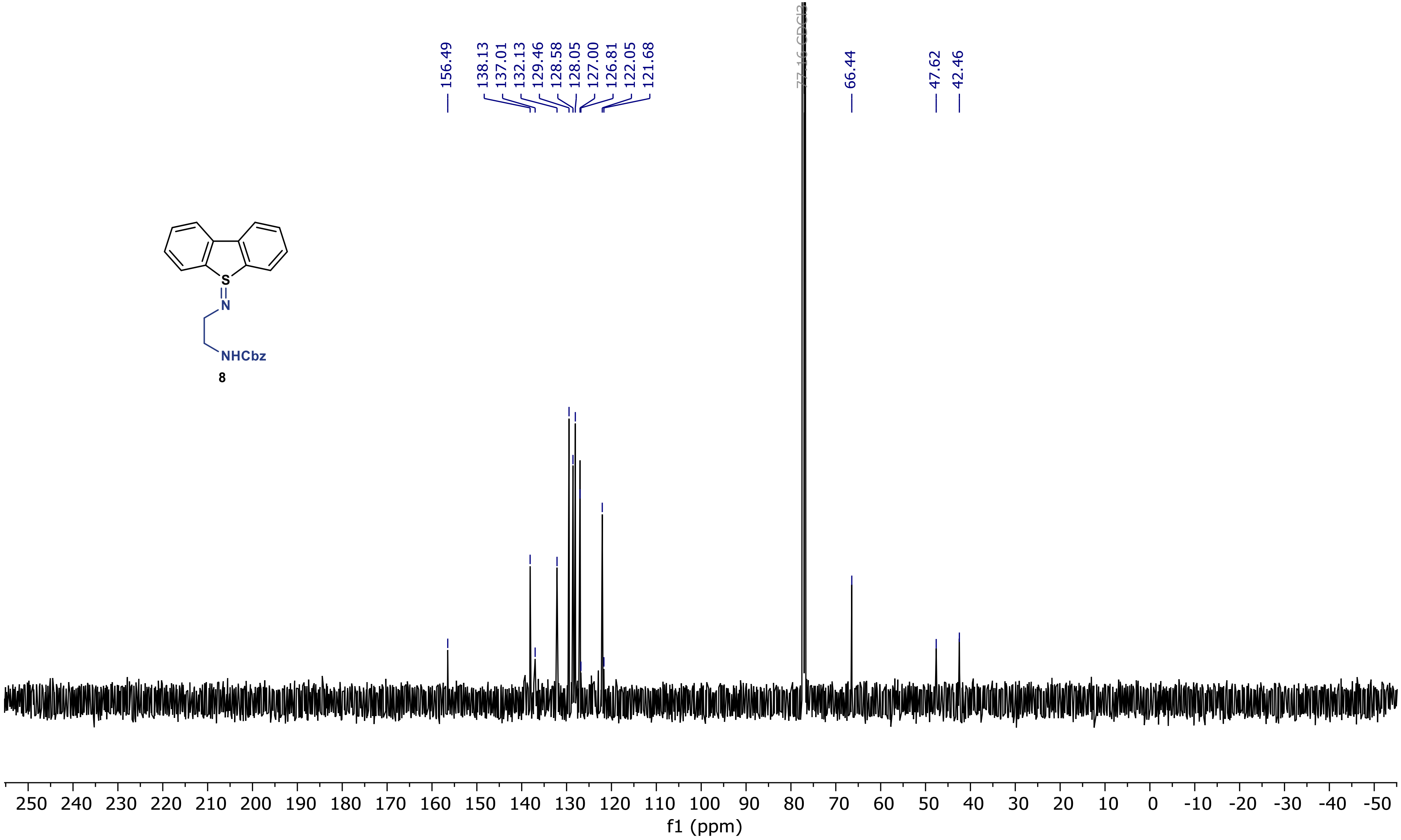

^{1}H NMR of sulfilimine 9

CDCl3, 23 ºC

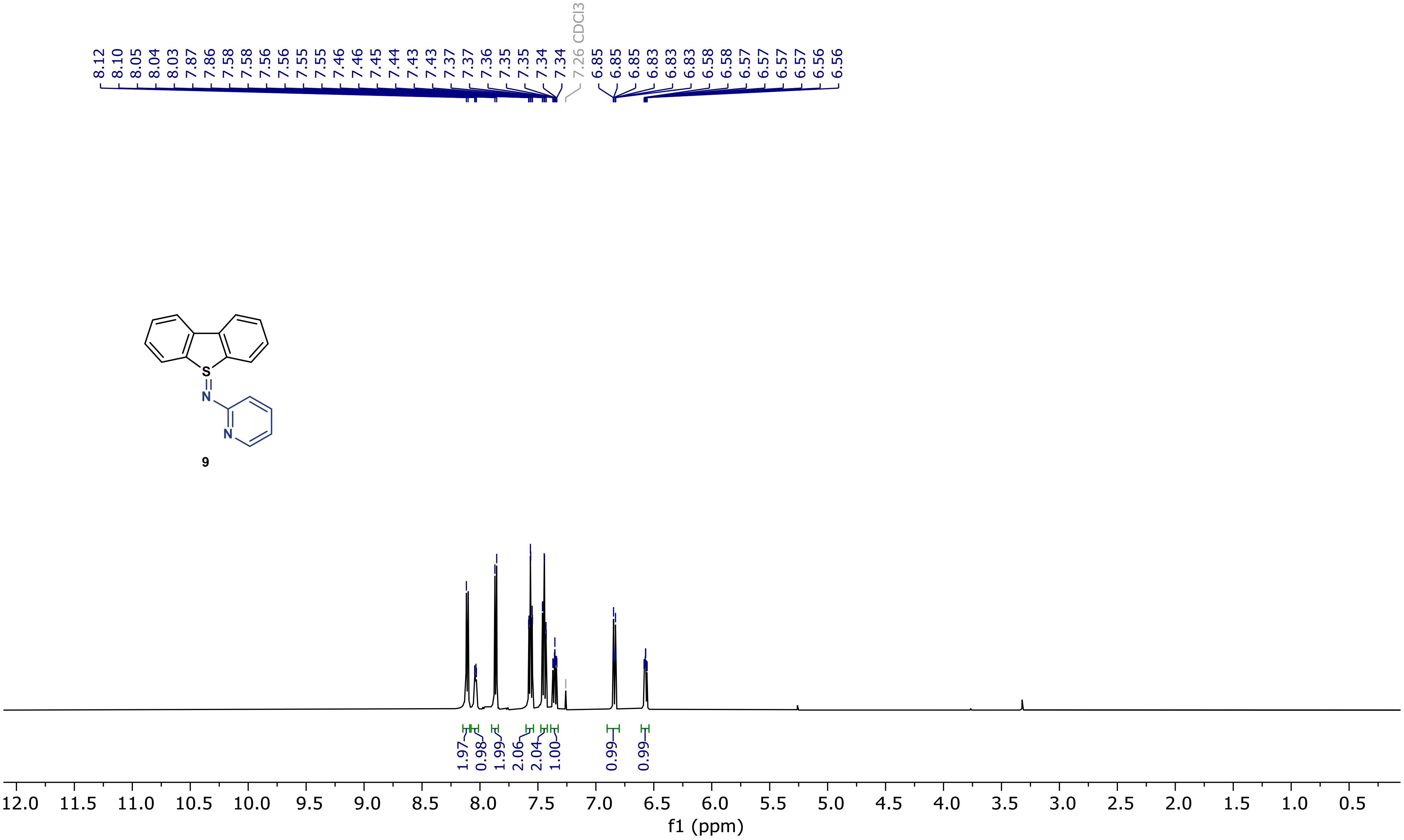

^{13}C NMR of sulfilimine 9

$CDCl_3$, 23 ºC

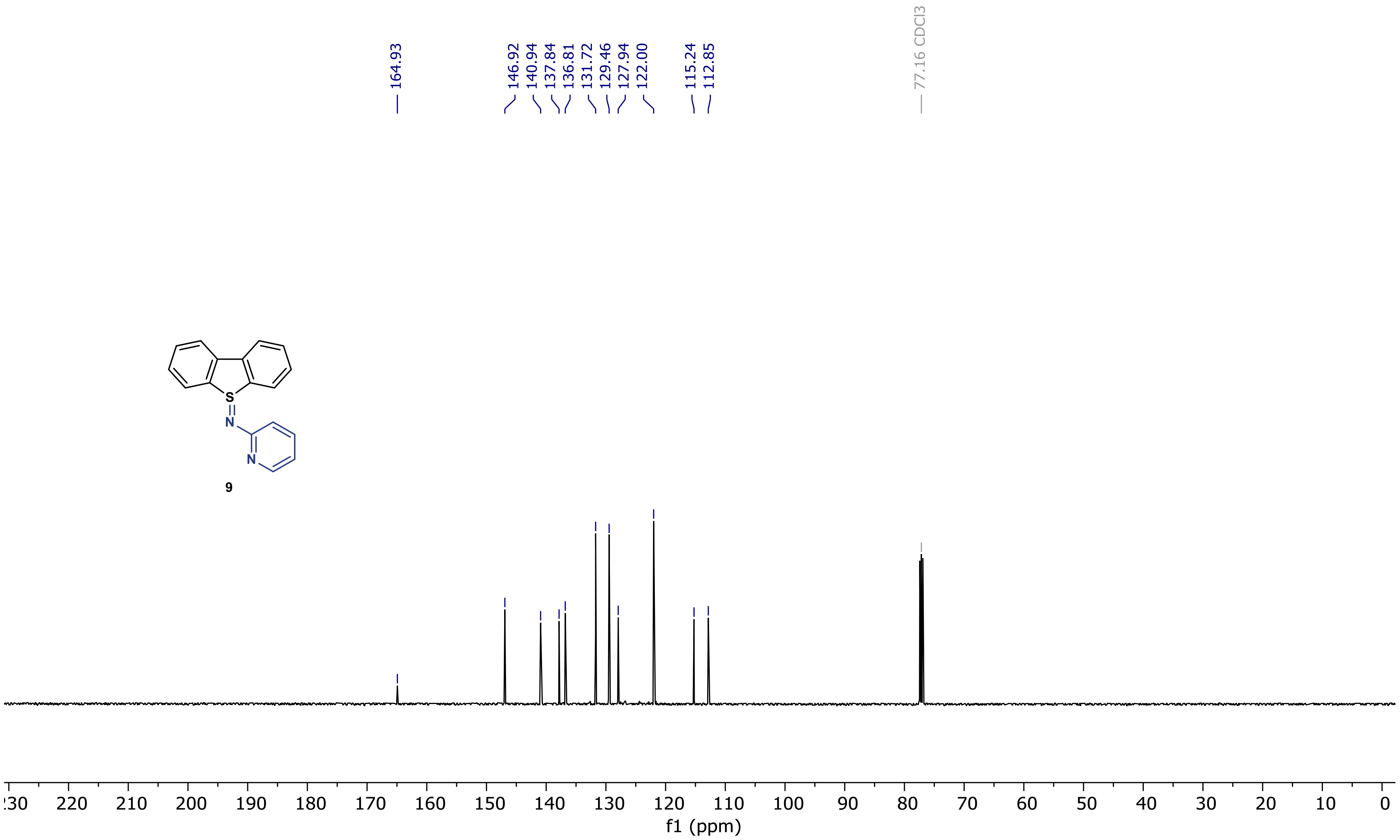

^{1}H NMR of sulfilimine 10

CDCl$_3$, 23 ºC

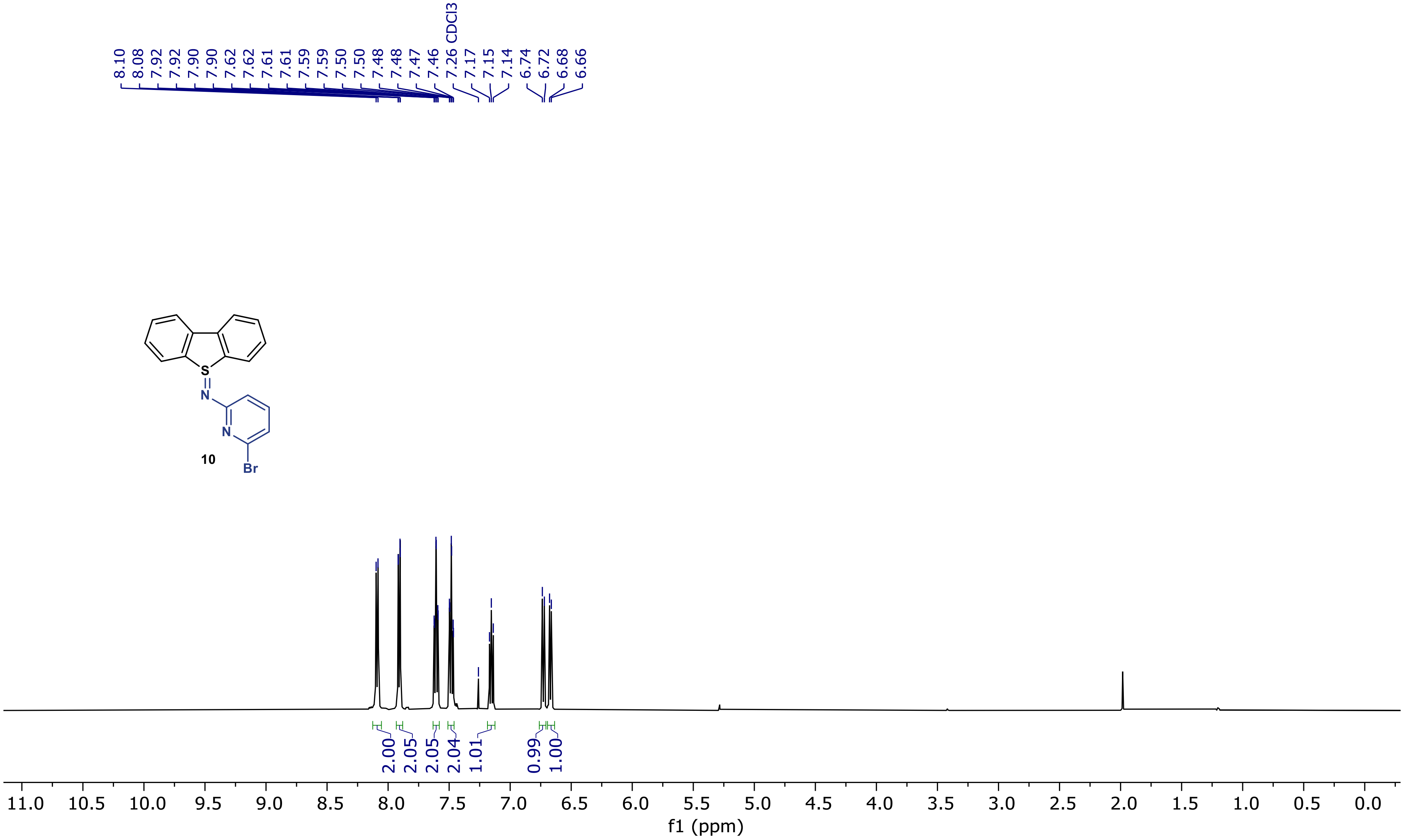

^{13}C NMR of sulfilimine 10

$CDCl_3$, 23 ºC

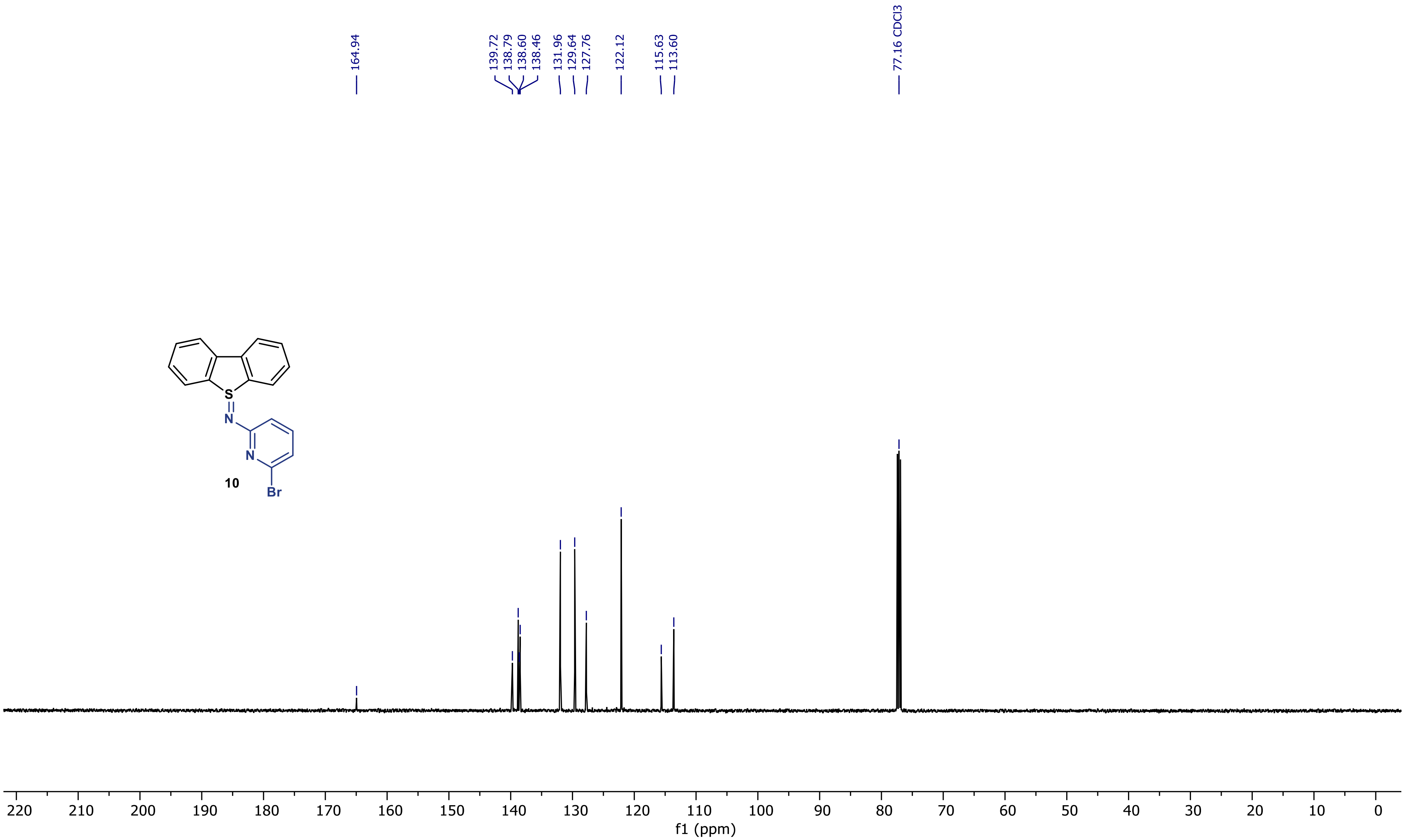

^{1}H NMR of dibenzothiophen-5-imine 11

CD_2Cl_2, 23 ºC

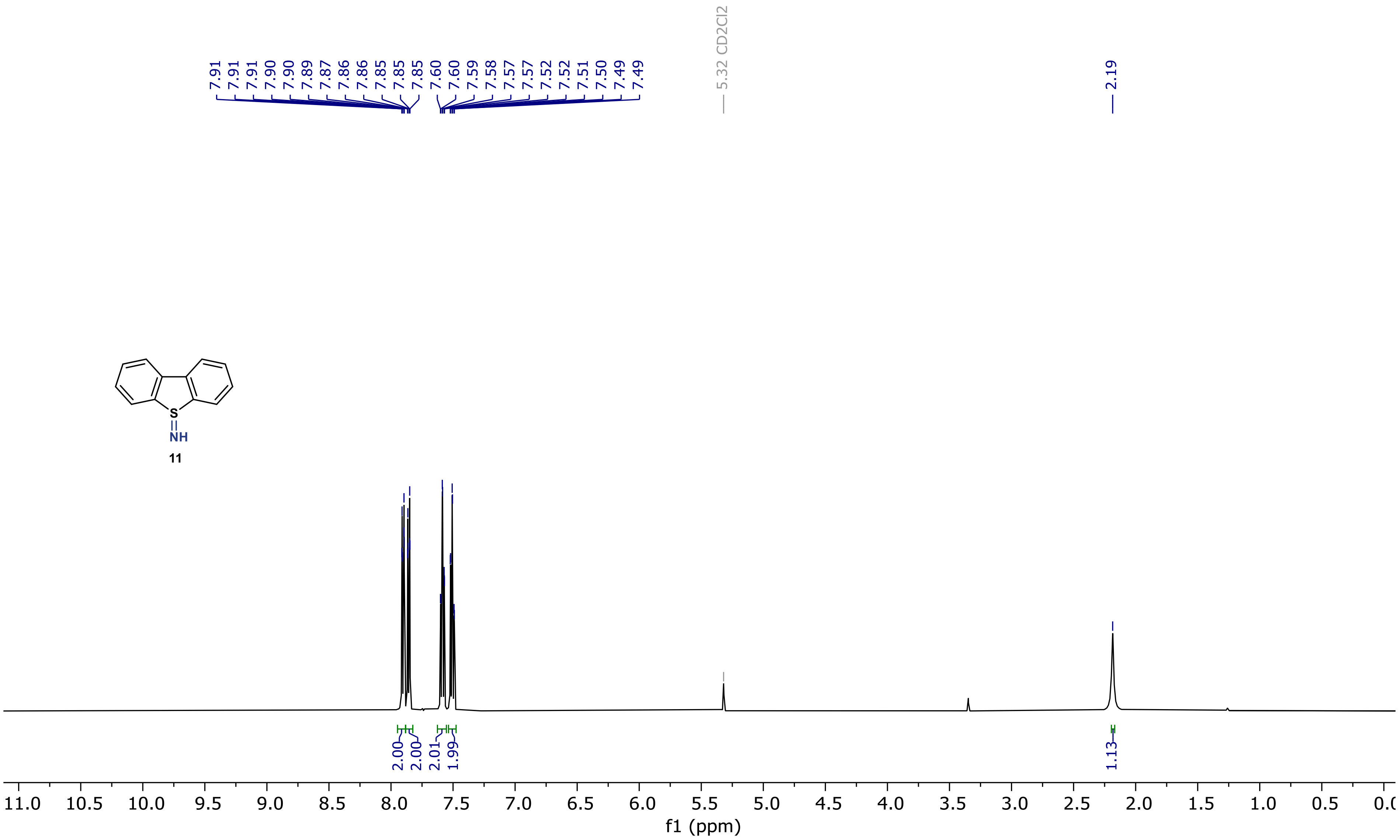

^{13}C NMR of dibenzothiophen-5-imine 11

CD_2Cl_2, 23 ºC

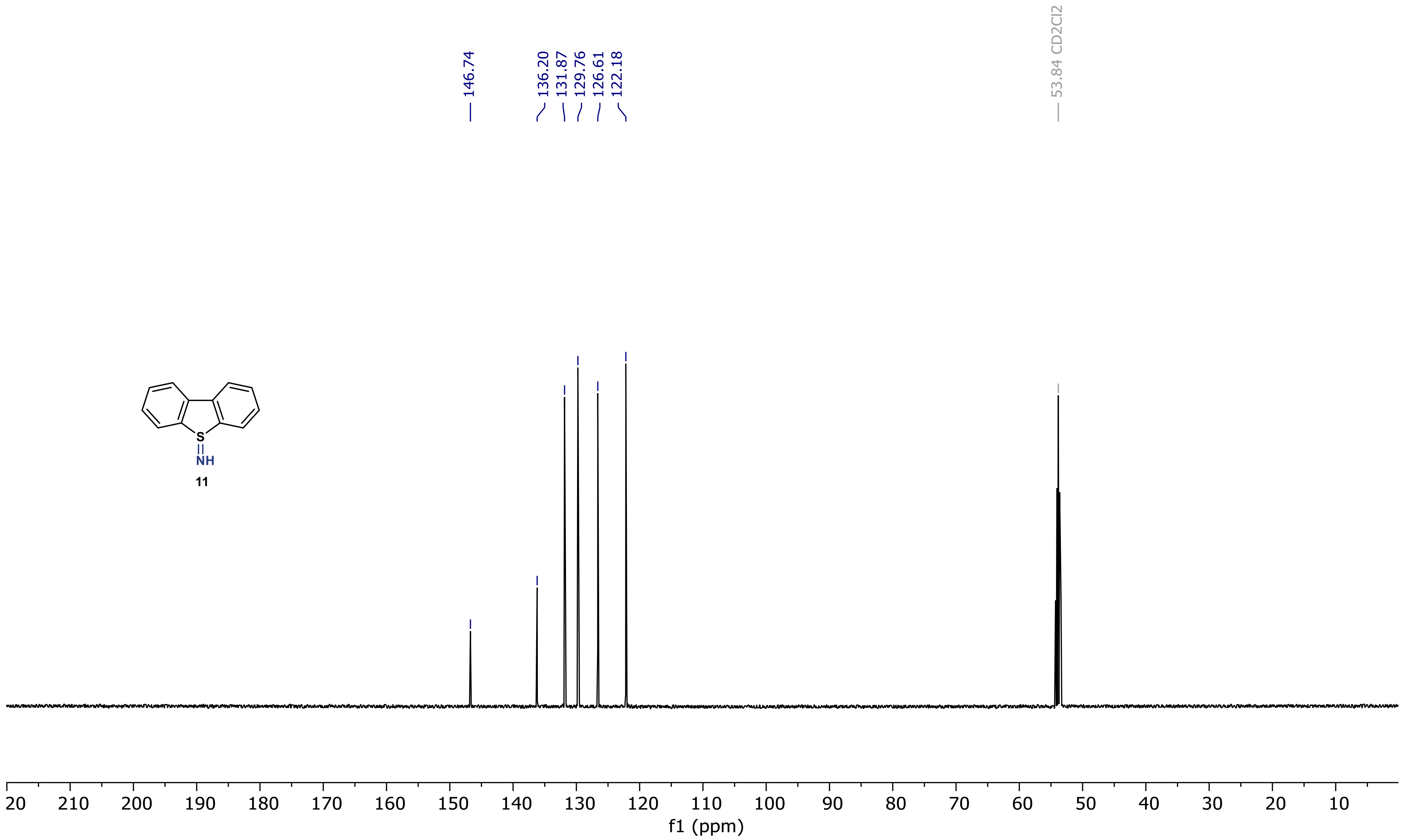

^{1}H NMR of sulfilimine 12

$CDCl_3$, 23 ºC

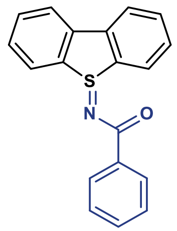

12

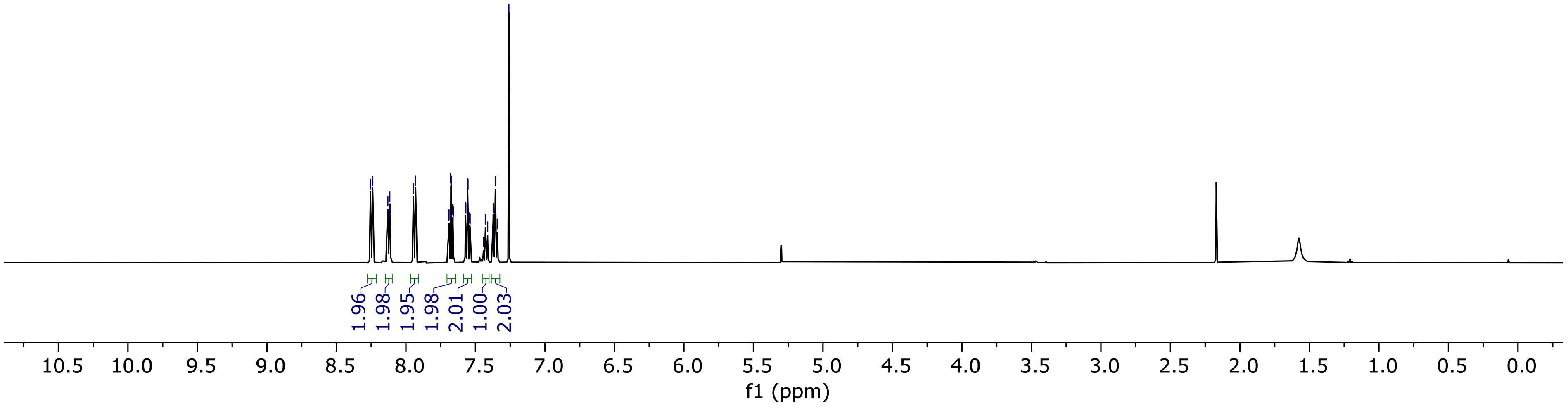

^{13}C NMR of sulfilimine 12

CDCl$_3$, 23 ºC

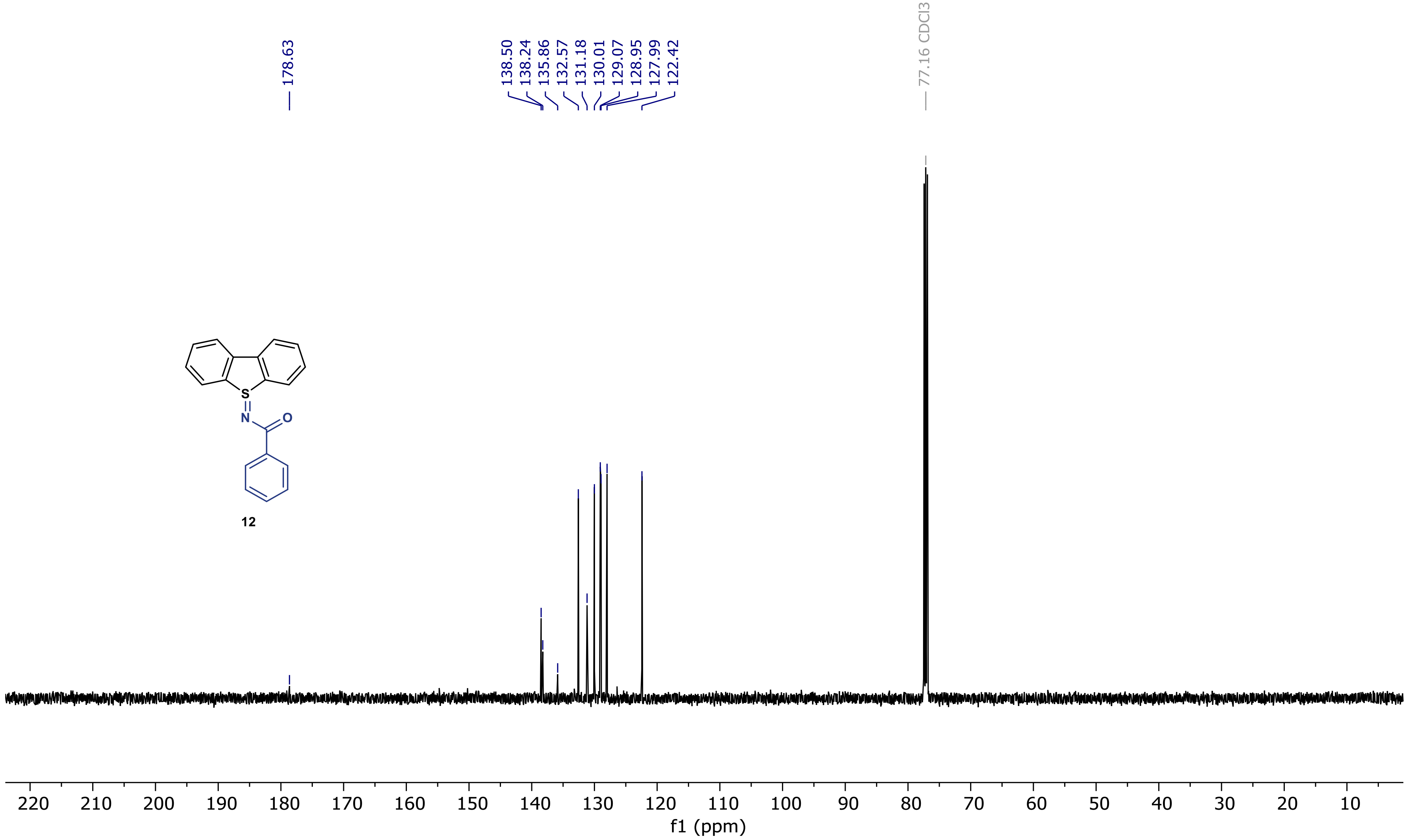

¹H NMR of sulfilimine 13

CDCl₃, 23 ºC

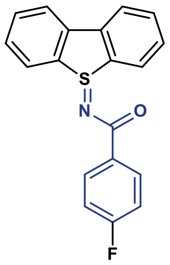

13

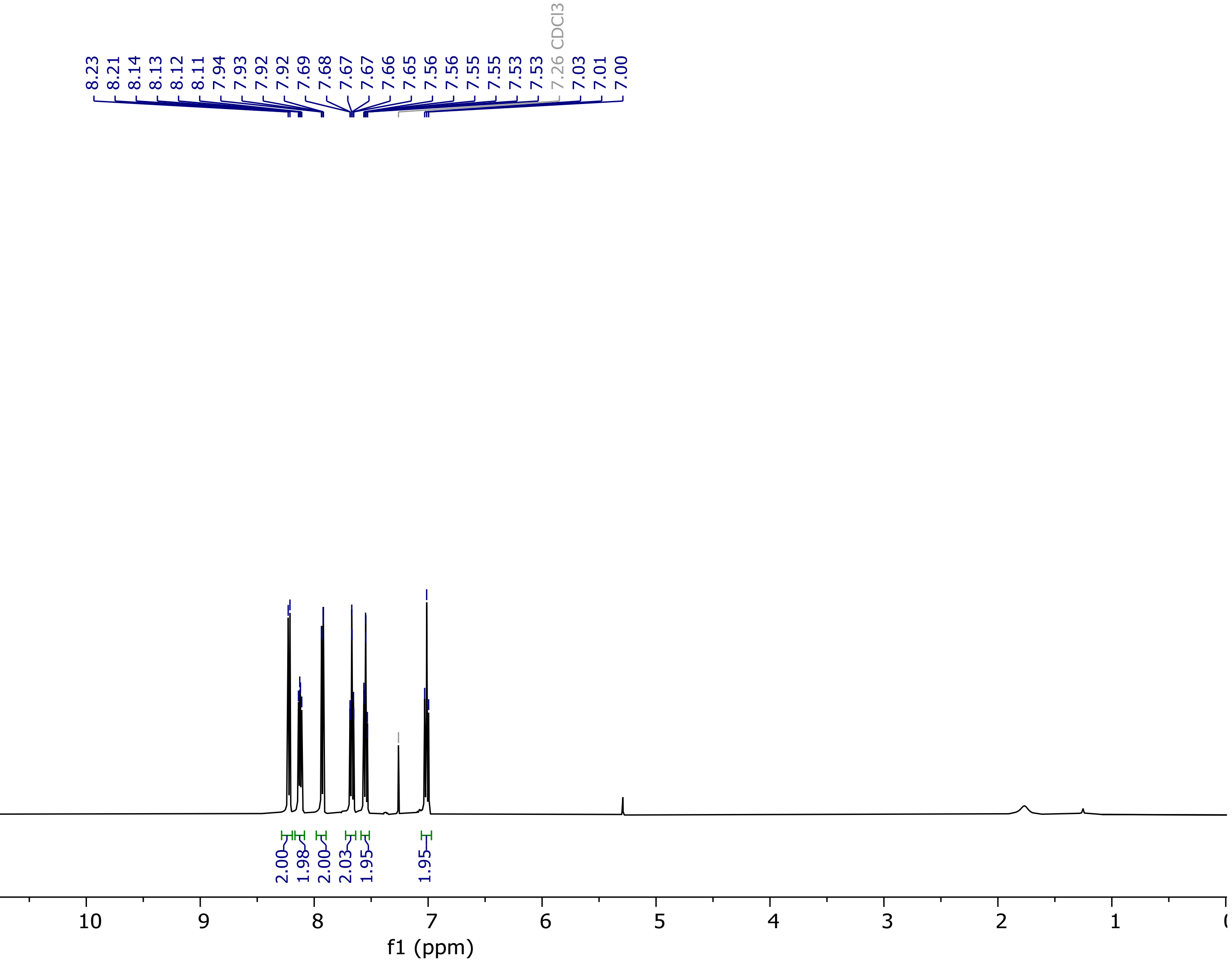

^{13}C NMR of sulfilimine 13

$CDCl_3$, 23 ºC

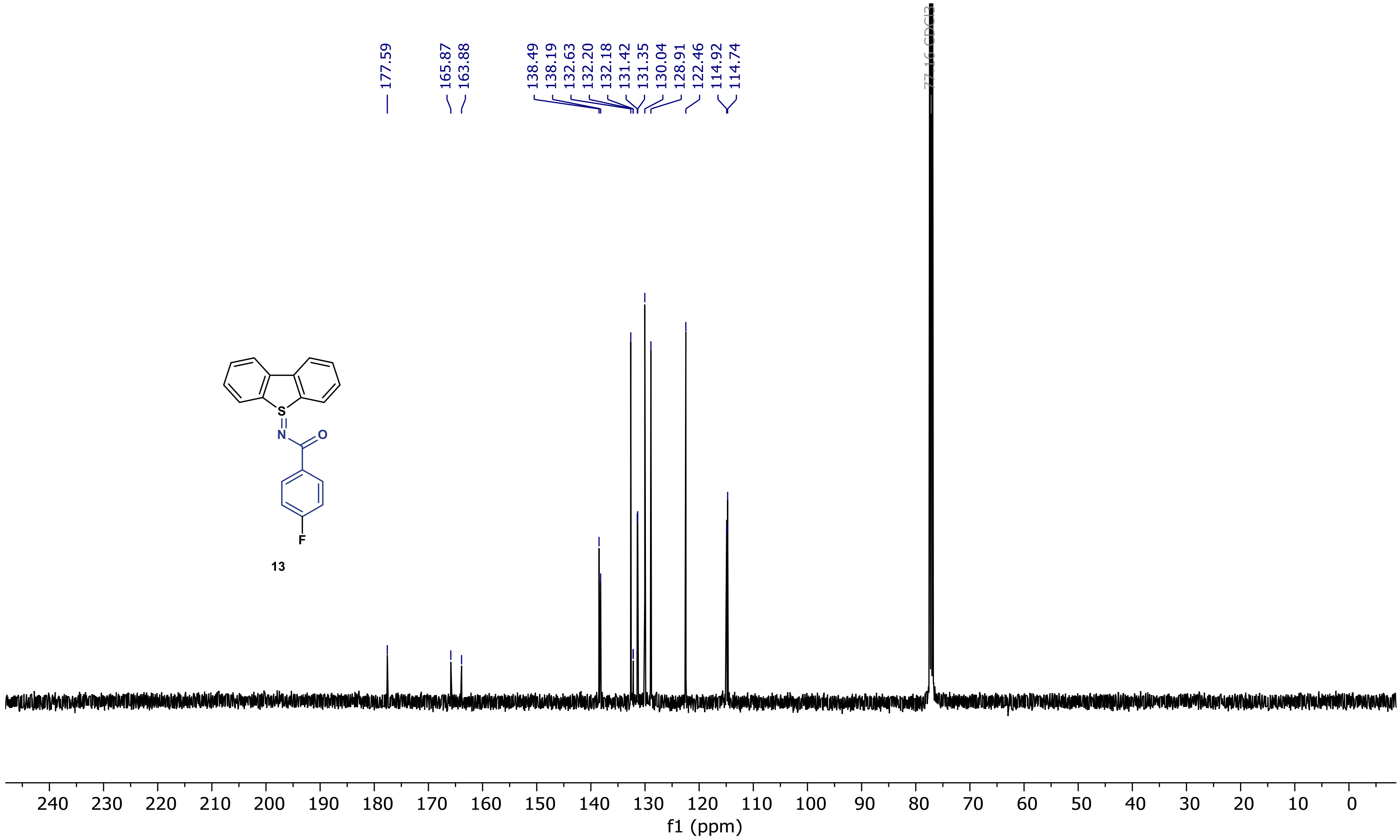

^{19}F NMR of sulfilimine 13

$CDCl_3$, 23 ºC

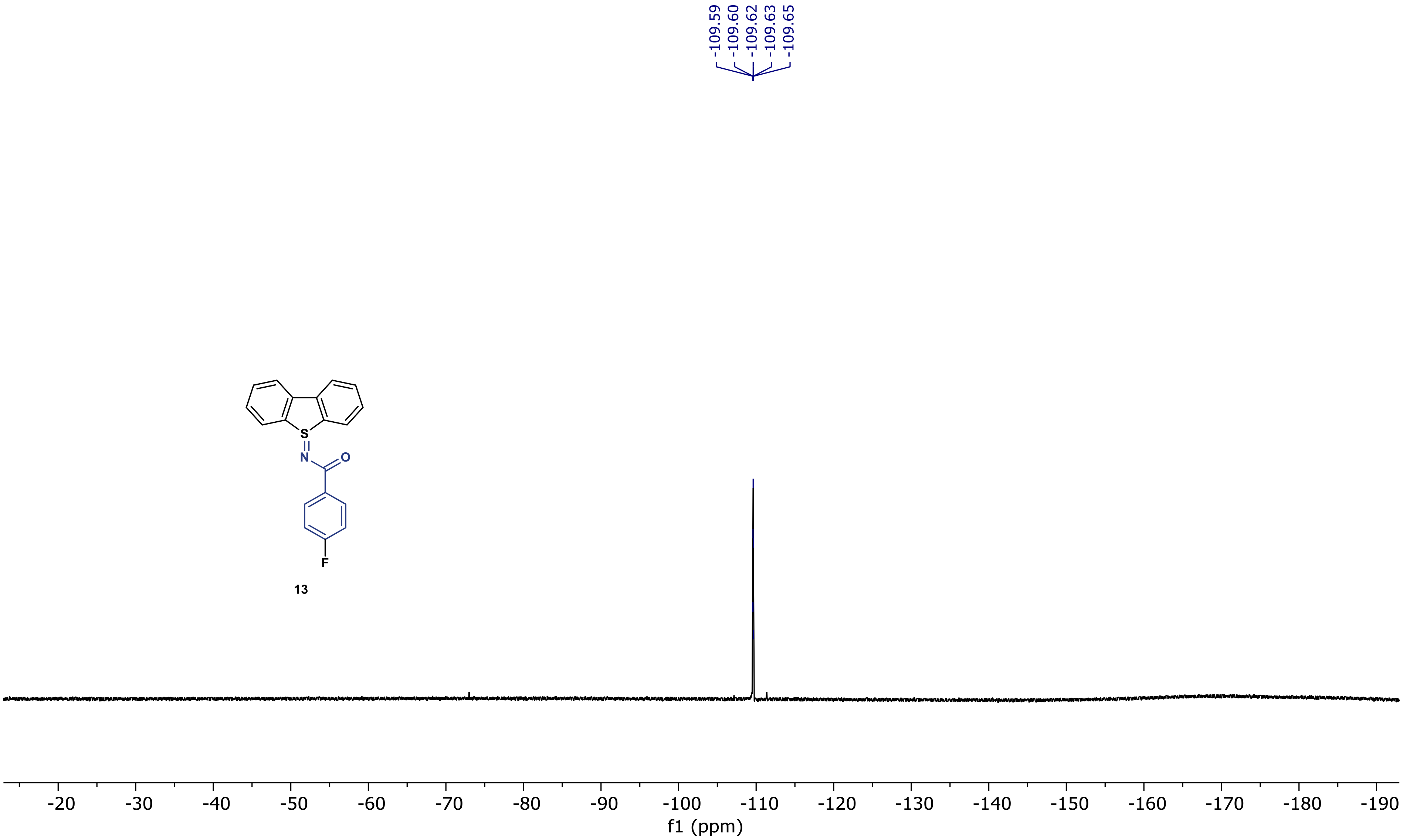

^{1}H NMR of sulfilimine 14

CDCl3, 23 ºC

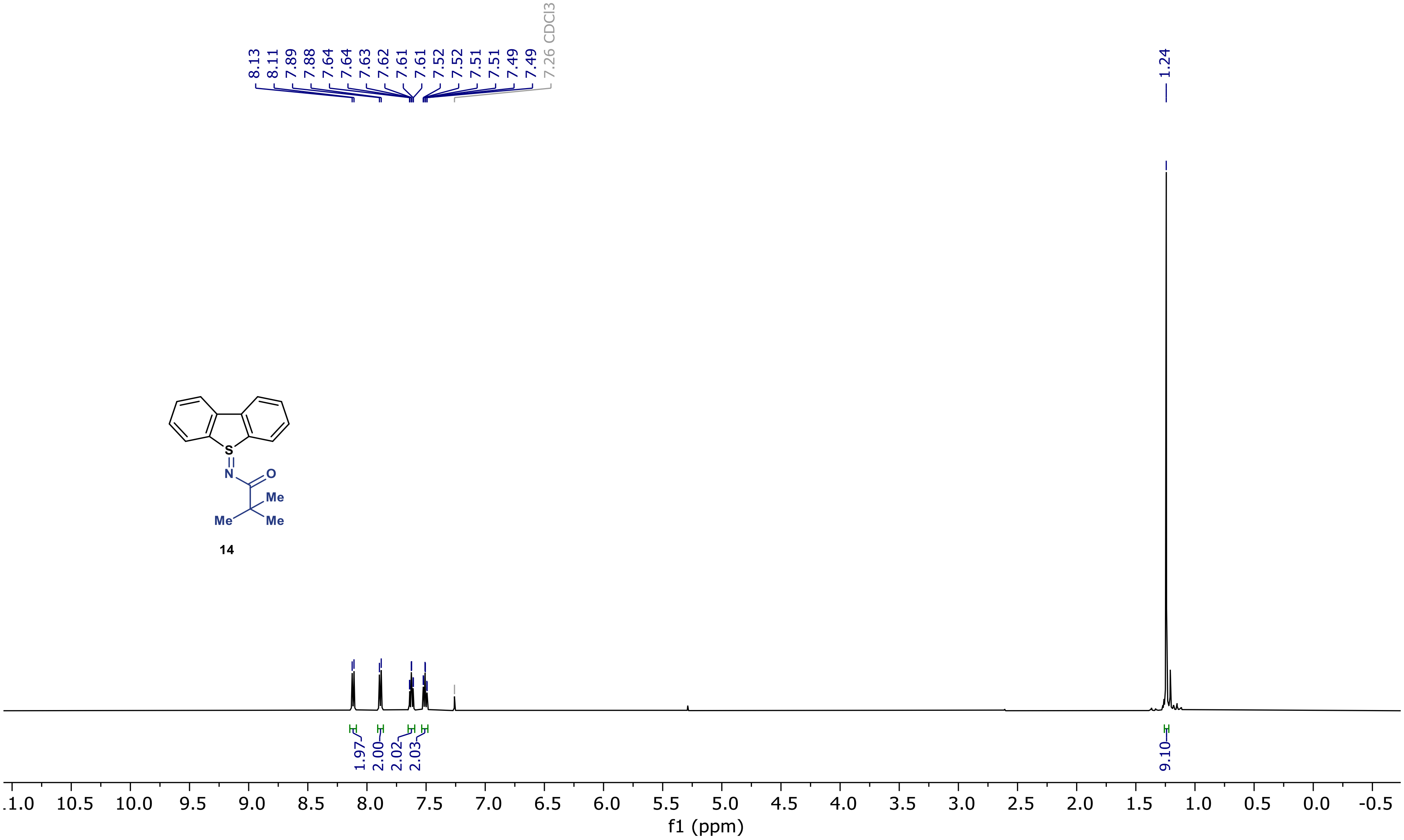

^{13}C NMR of sulfilimine 14

$CDCl_3$, 23 ºC

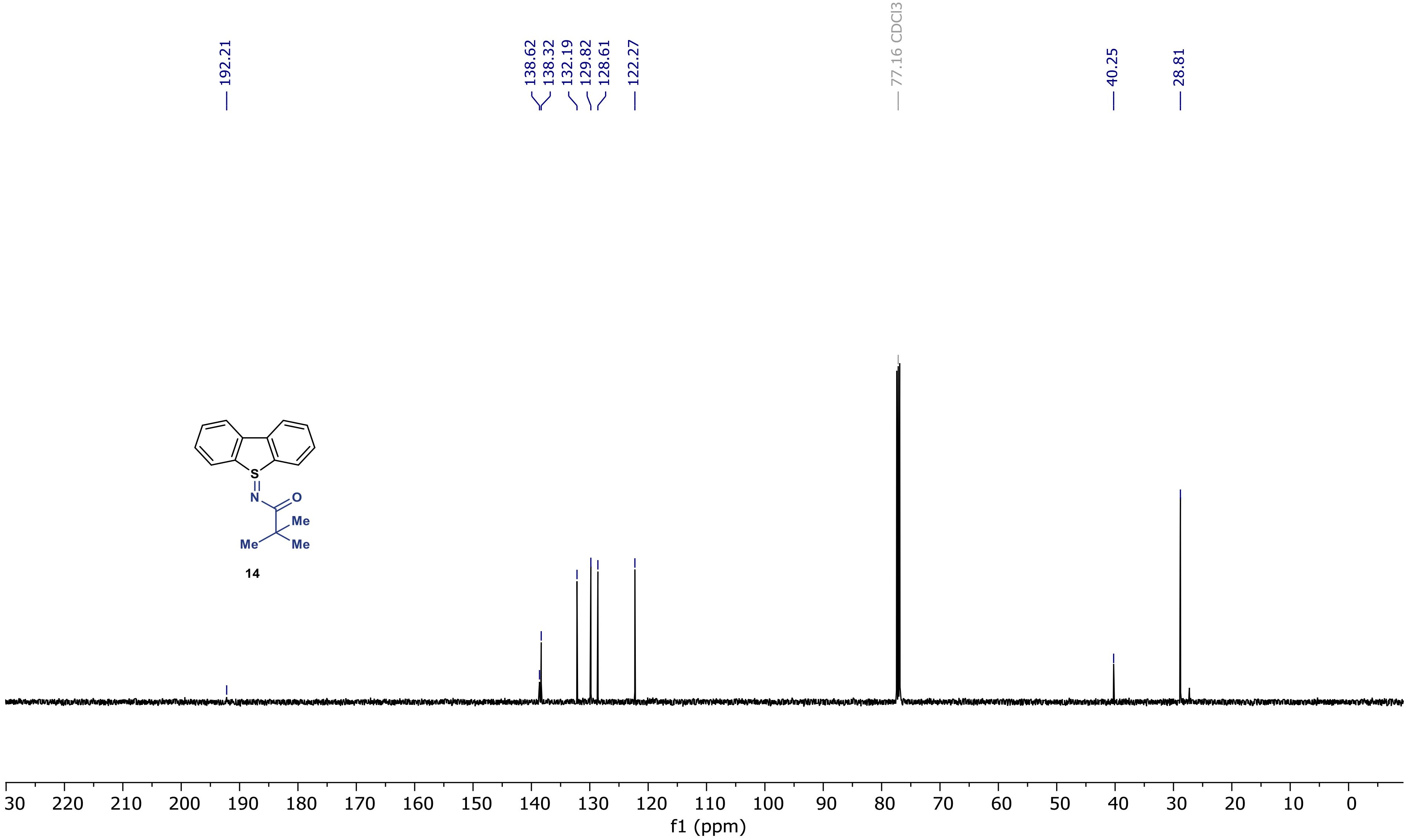

^{1}H NMR of sulfilimine 15

CDCl$_3$, 23 ºC

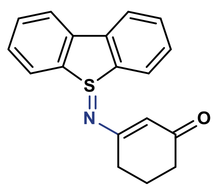

15

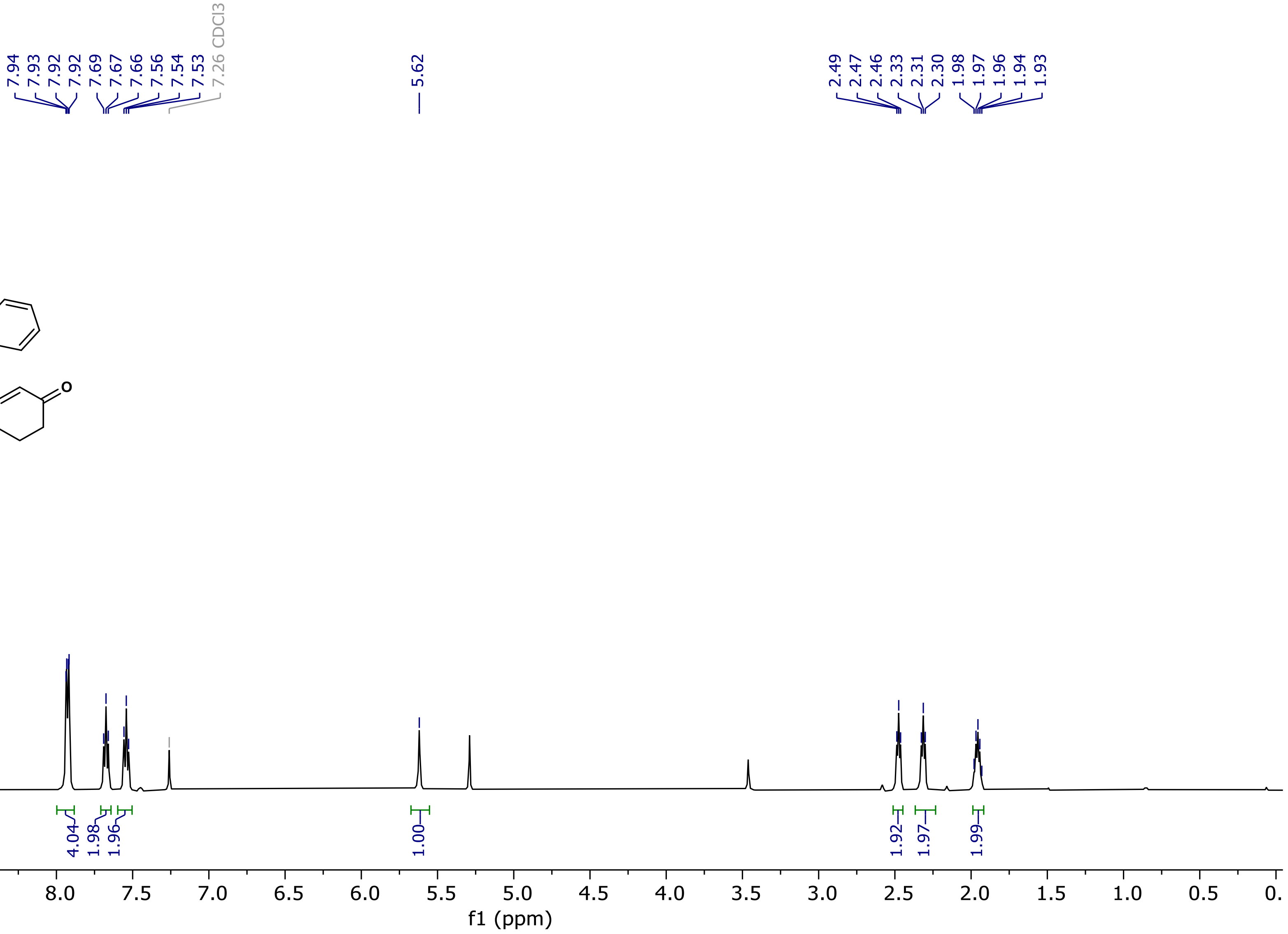

^{13}C NMR of sulfilimine 15

CDCl₃, 23 ºC

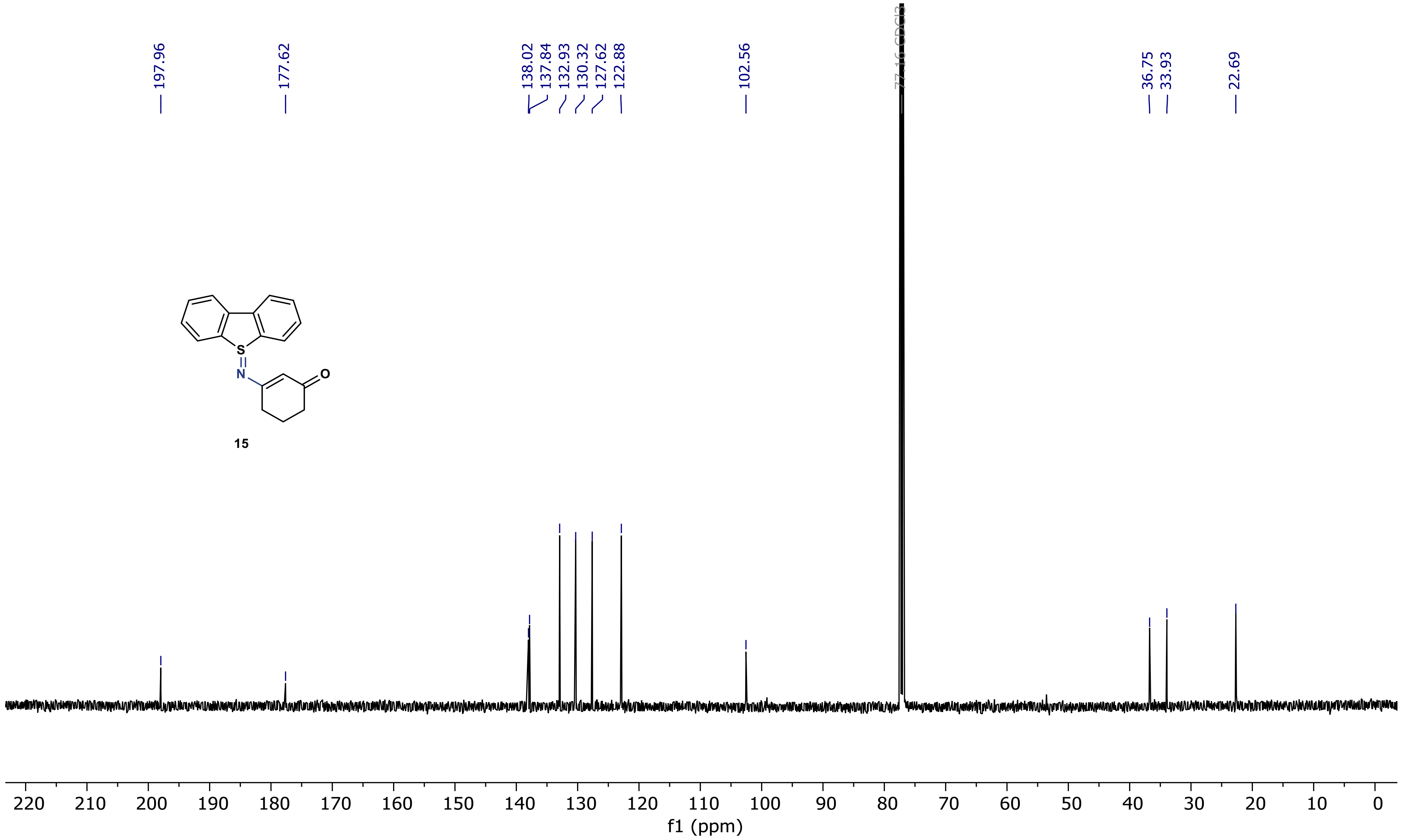

^{1}H NMR of sulfilimine 16

CDCl₃, 23 ºC

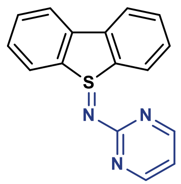

16

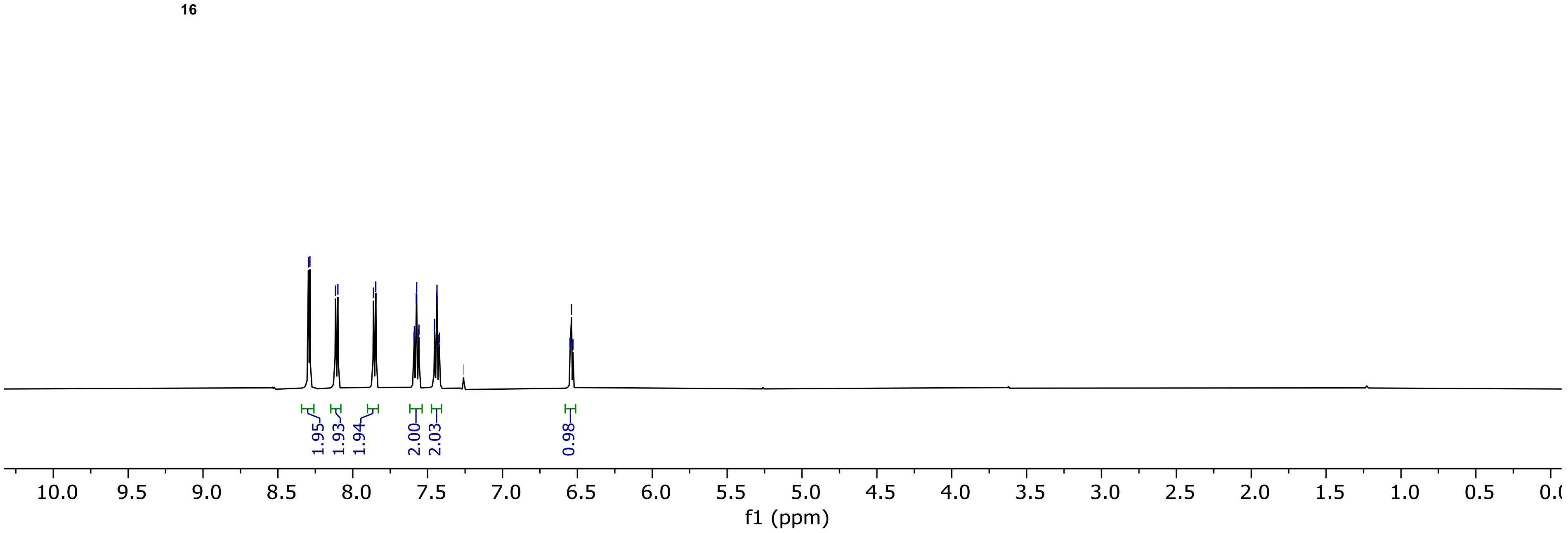

^{13}C NMR of sulfilimine 16

$CDCl_3$, 23 ºC

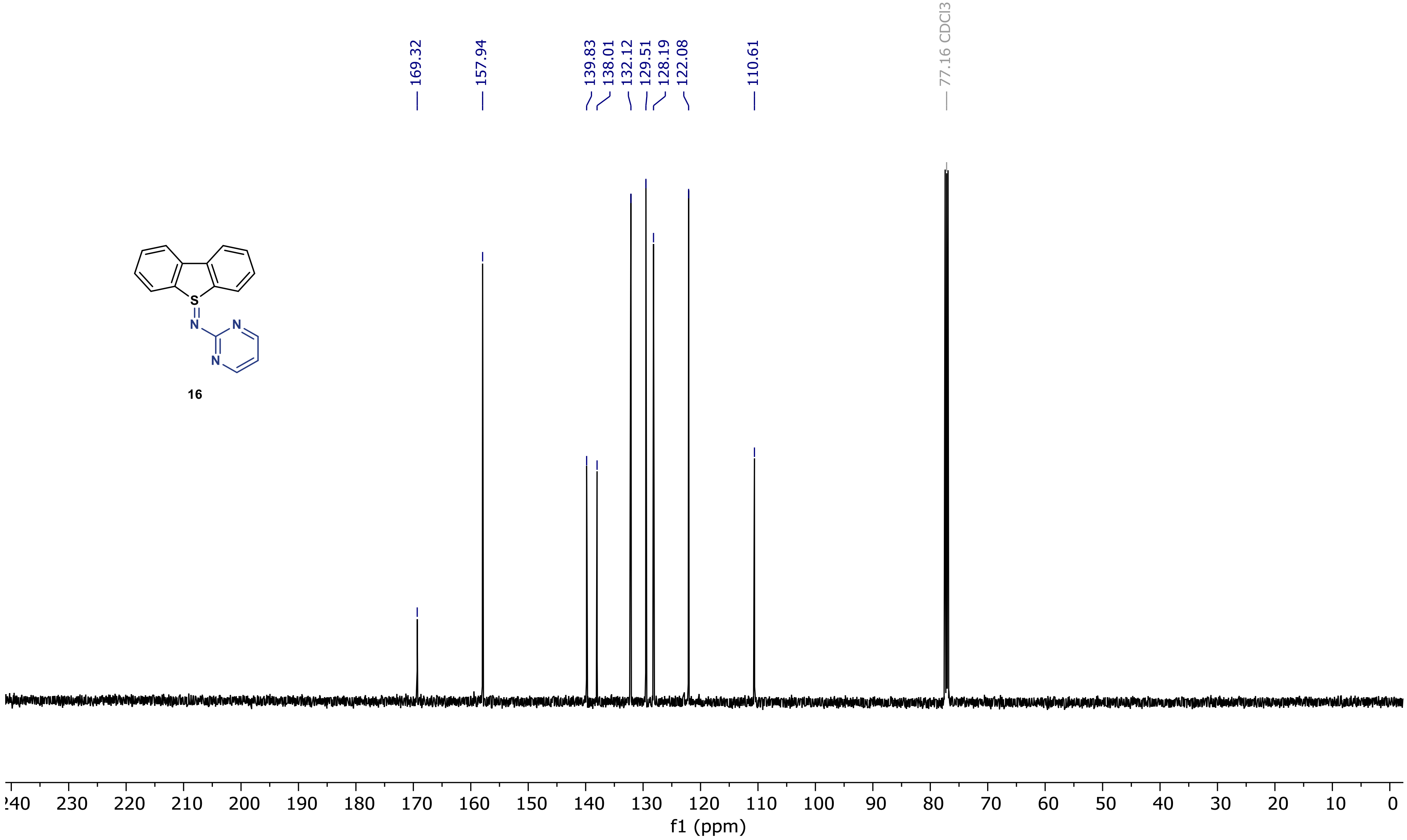

^{1}H NMR of sulfilimine 17

CDCl$_3$, 23 ºC

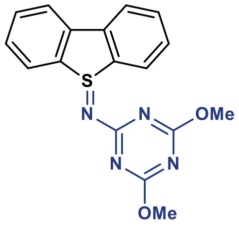

17

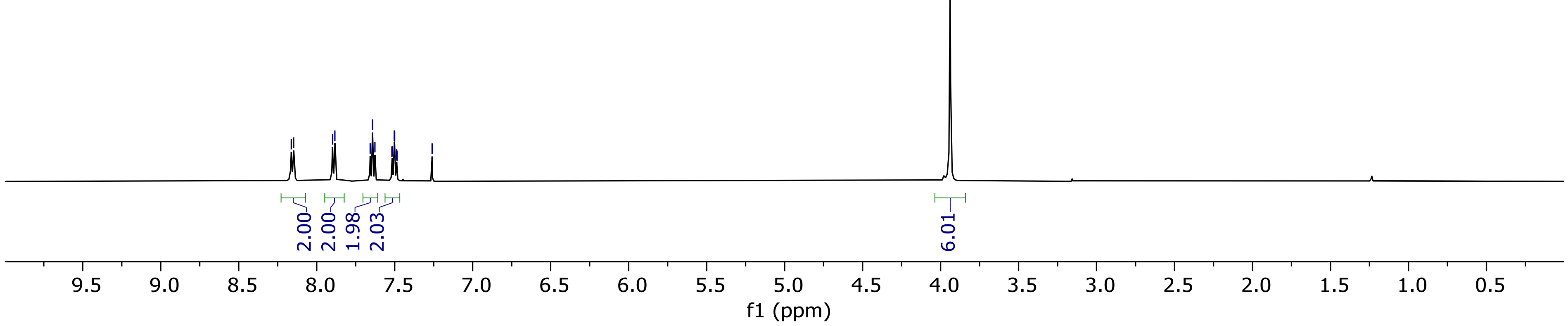

^{13}C NMR of sulfilimine 17

$CDCl_3$, 23 ºC

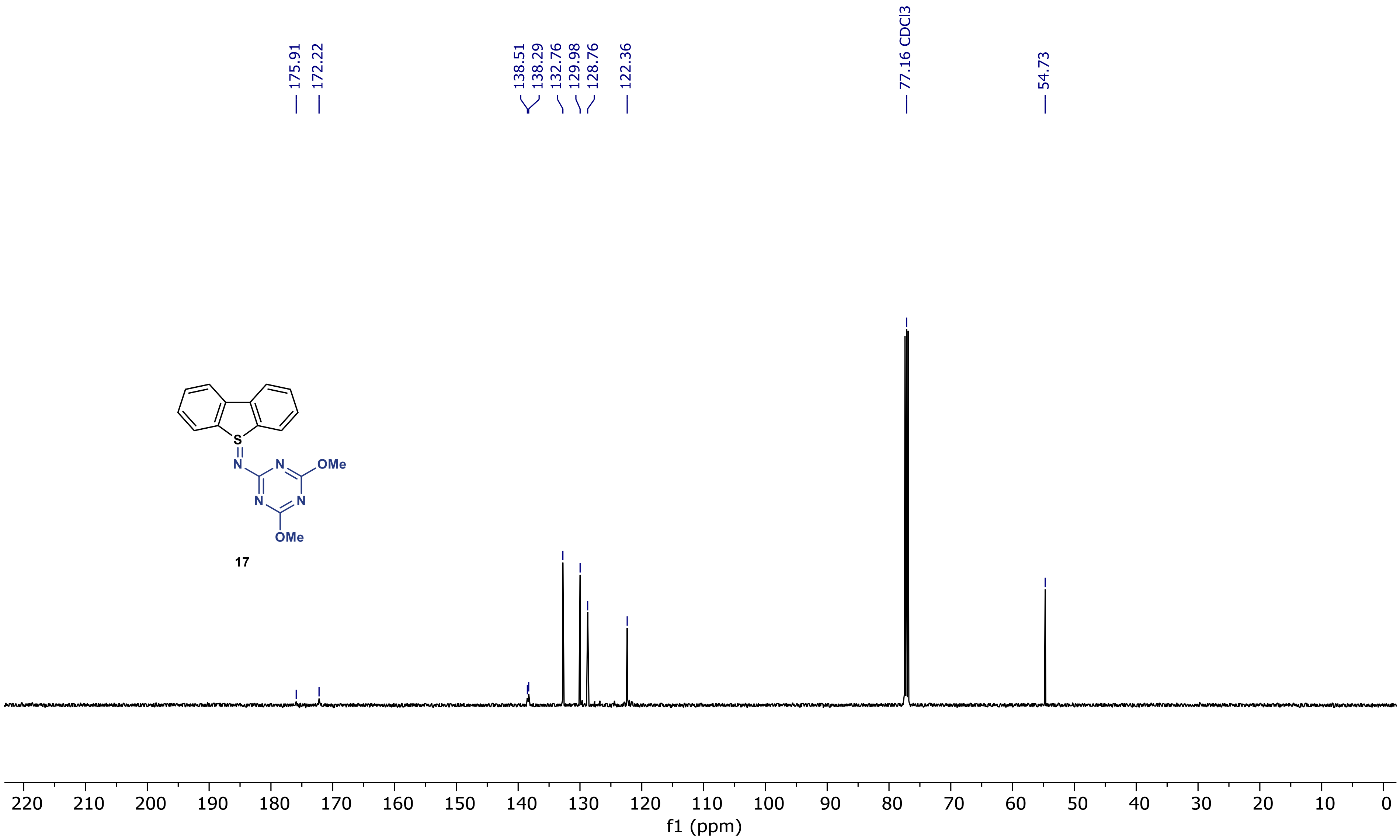

^{1}H NMR of N-Boc-2-phenylmorpholine 18

CDCl3, 23 ºC

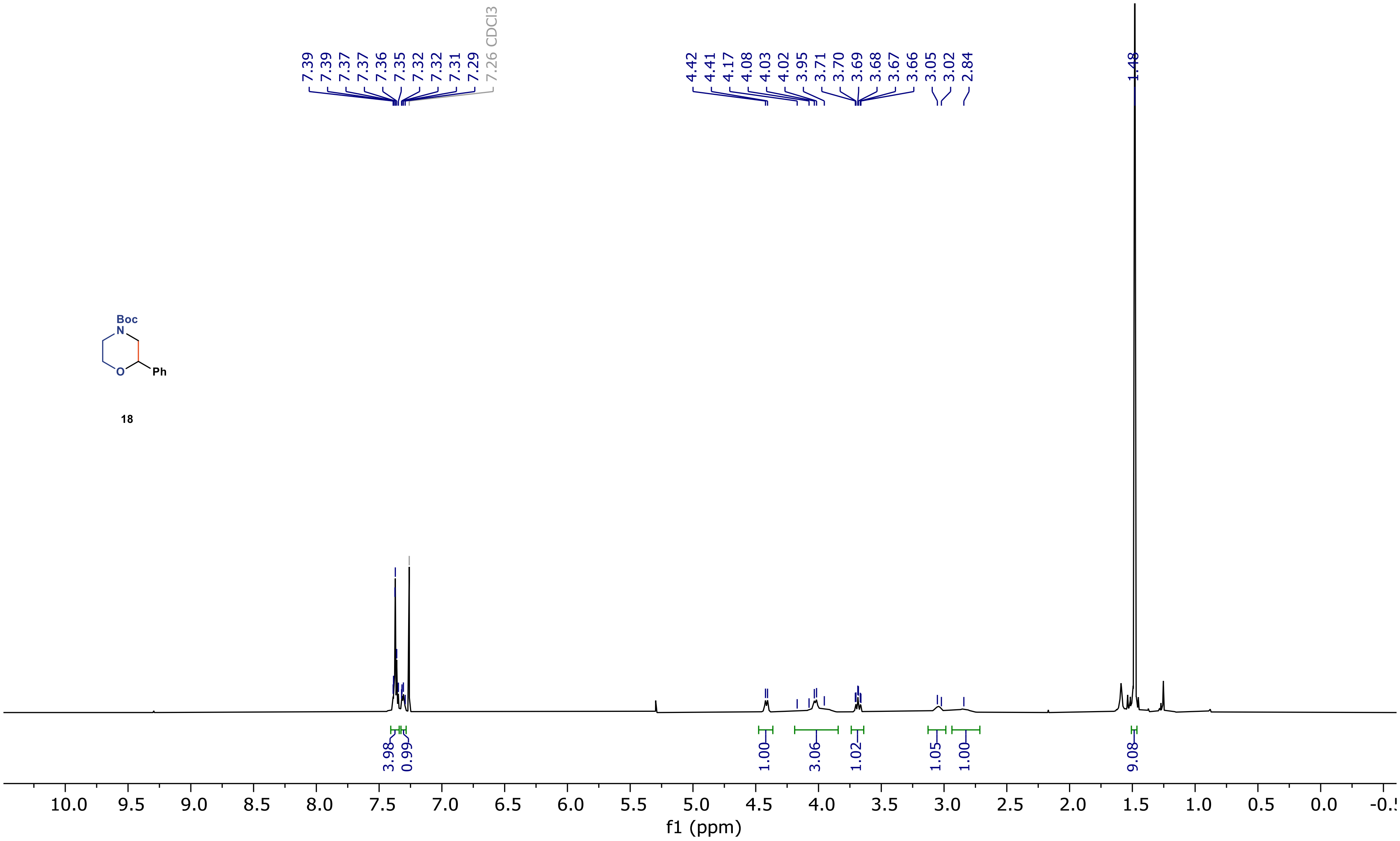

^{13}C NMR of N-Boc-2-phenylmorpholine 18

$CDCl_3$, 23 ºC

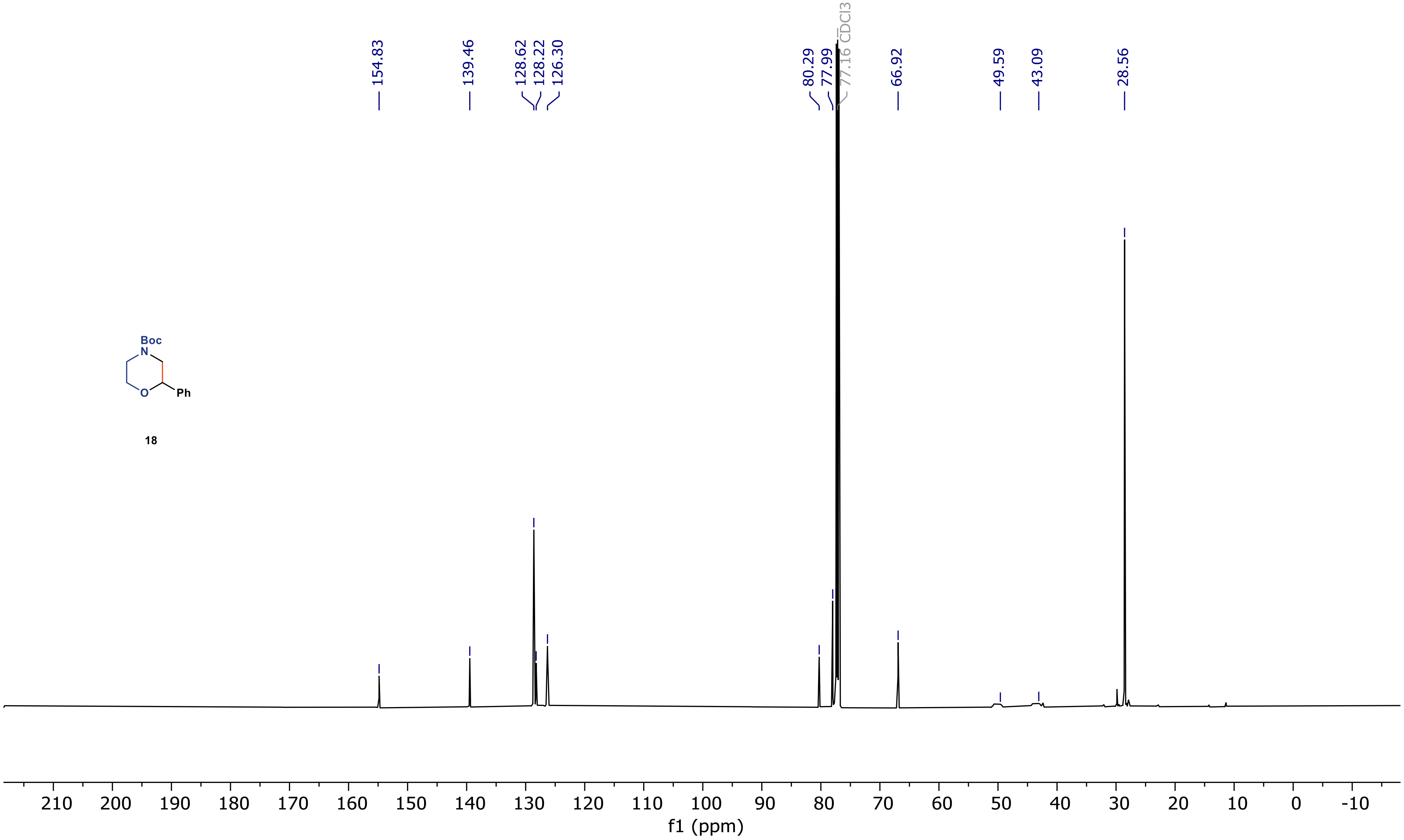

^{1}H NMR of 2-(4-methoxyphenyl)morpholine 19

CDCl3, 23 ºC

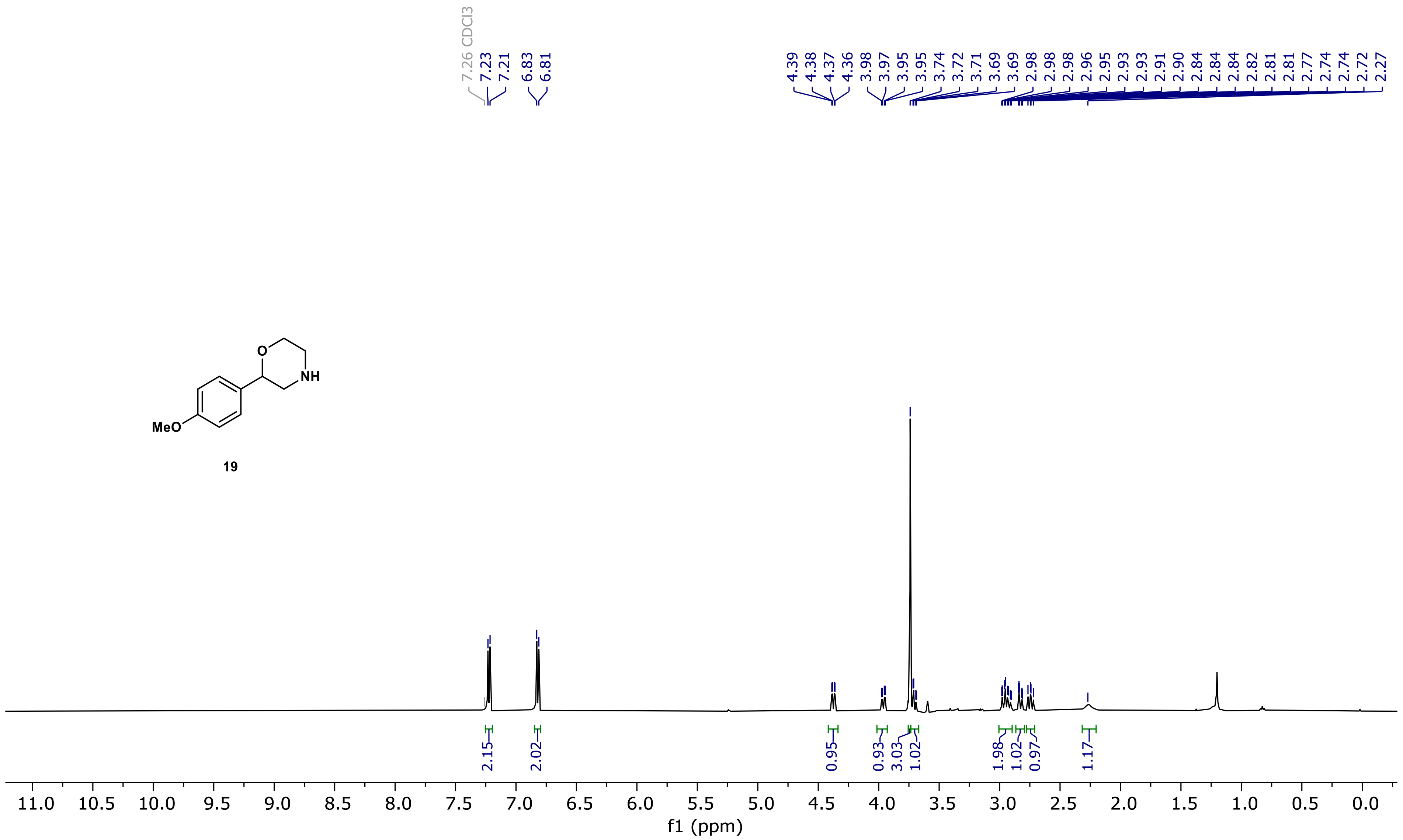

^{13}C NMR of 2-(4-methoxyphenyl)morpholine 19

$CDCl_3$, 23 ºC

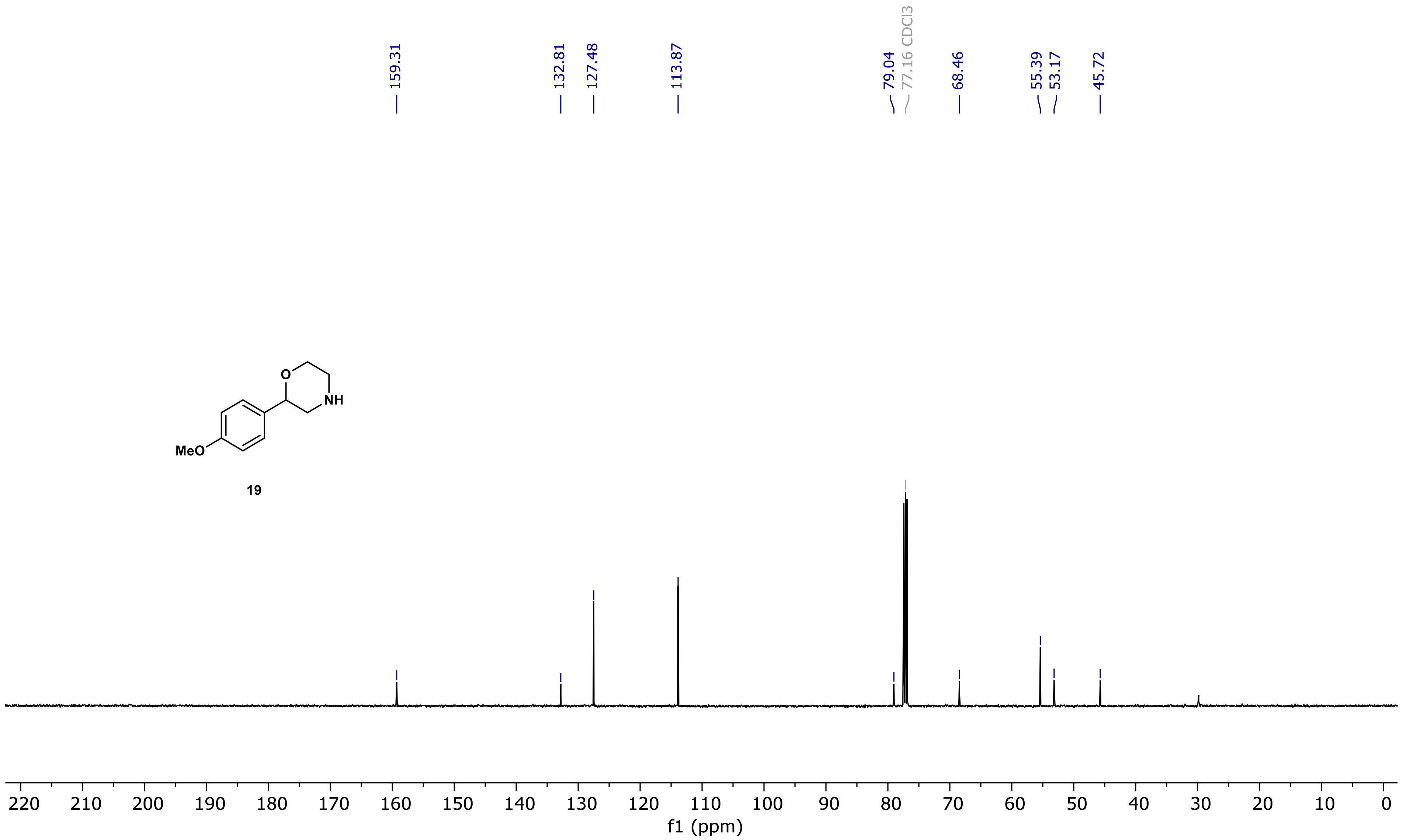

^{1}H NMR of 2-(4-fluorophenyl)morpholine 20

$CDCl_3$, 23 ºC

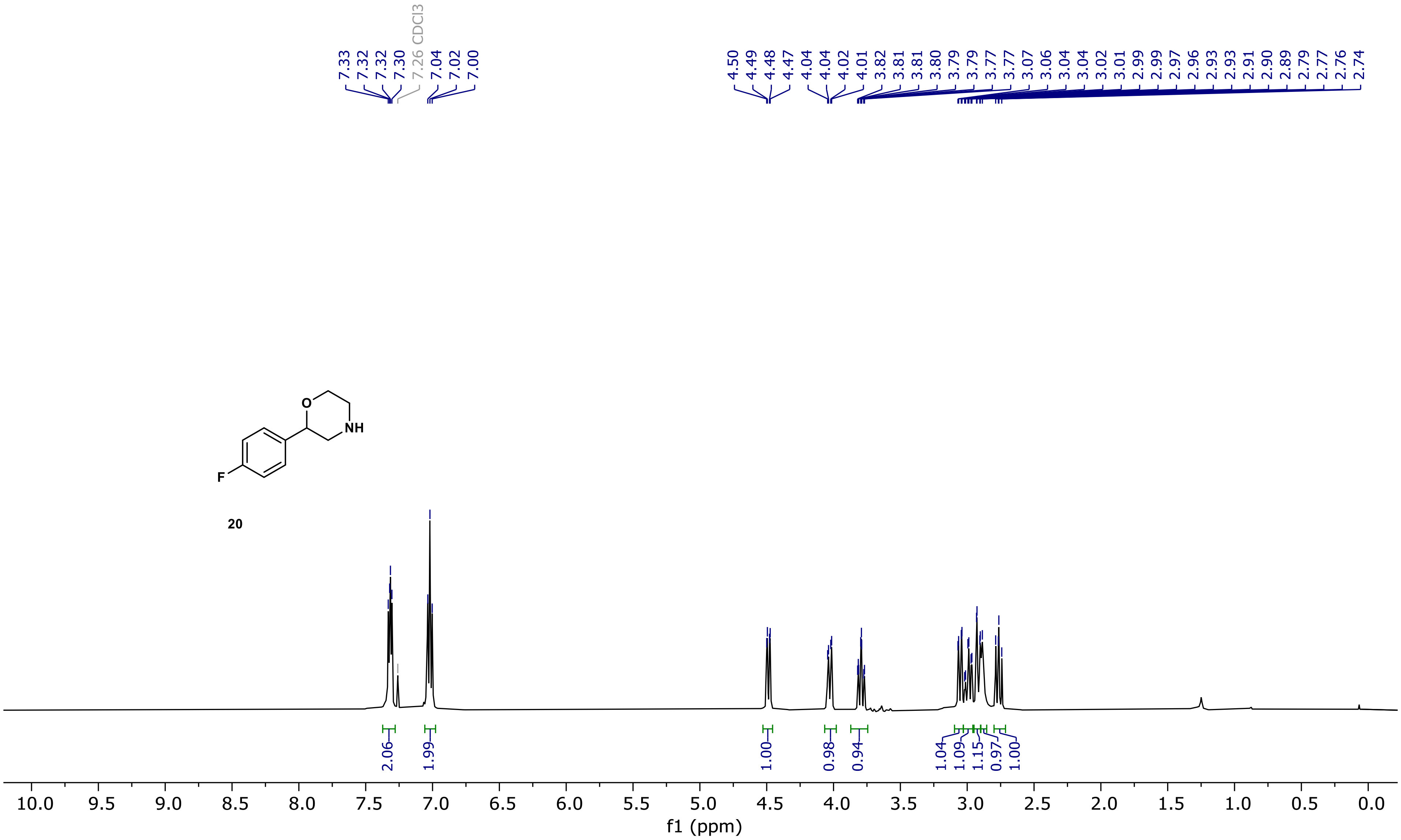

^{13}C NMR of 2-(4-fluorophenyl)morpholine 20

$CDCl_3$, 23 ºC

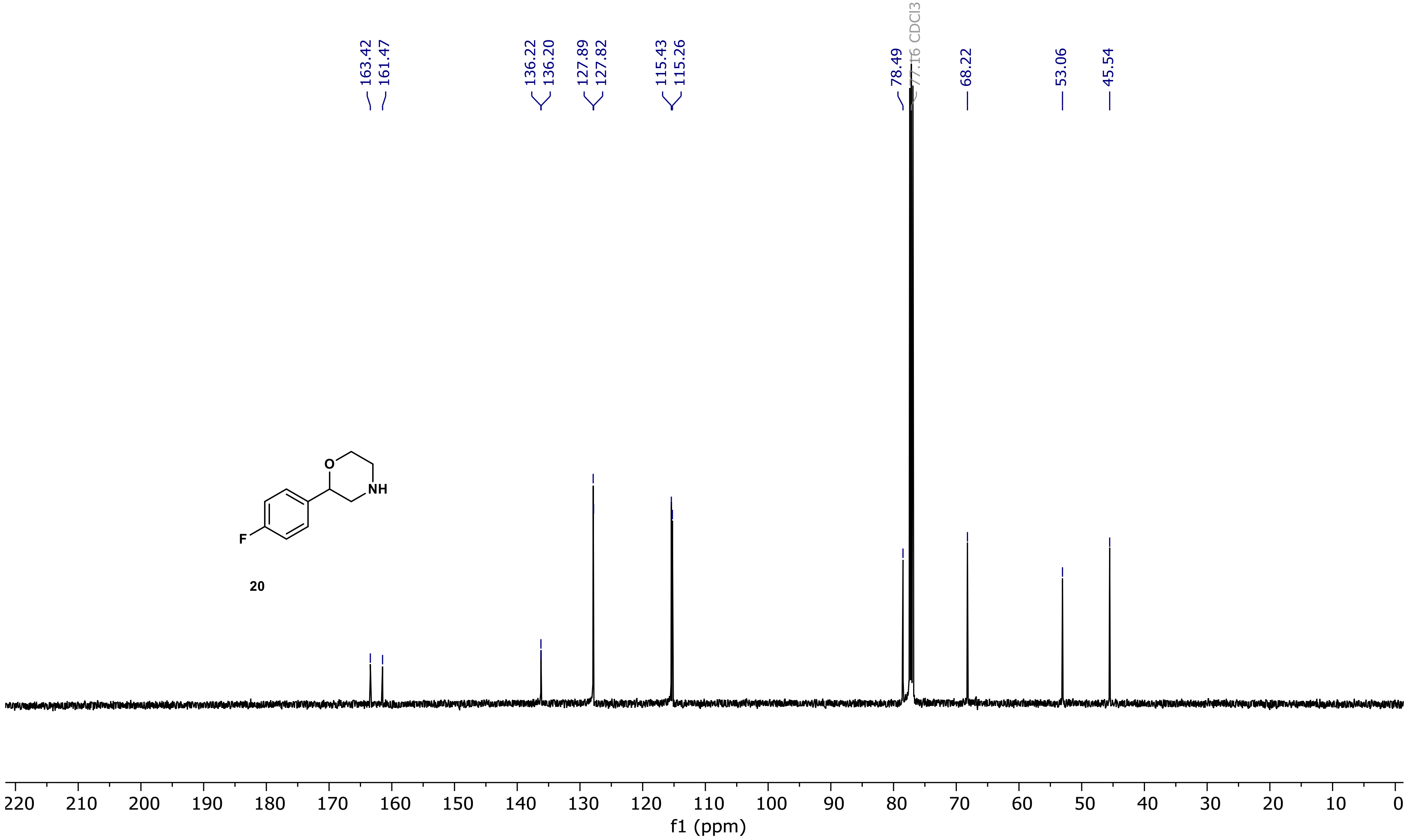

^{19}F NMR of 2-(4-fluorophenyl)morpholine 20

CDCl$_3$, 23 ºC

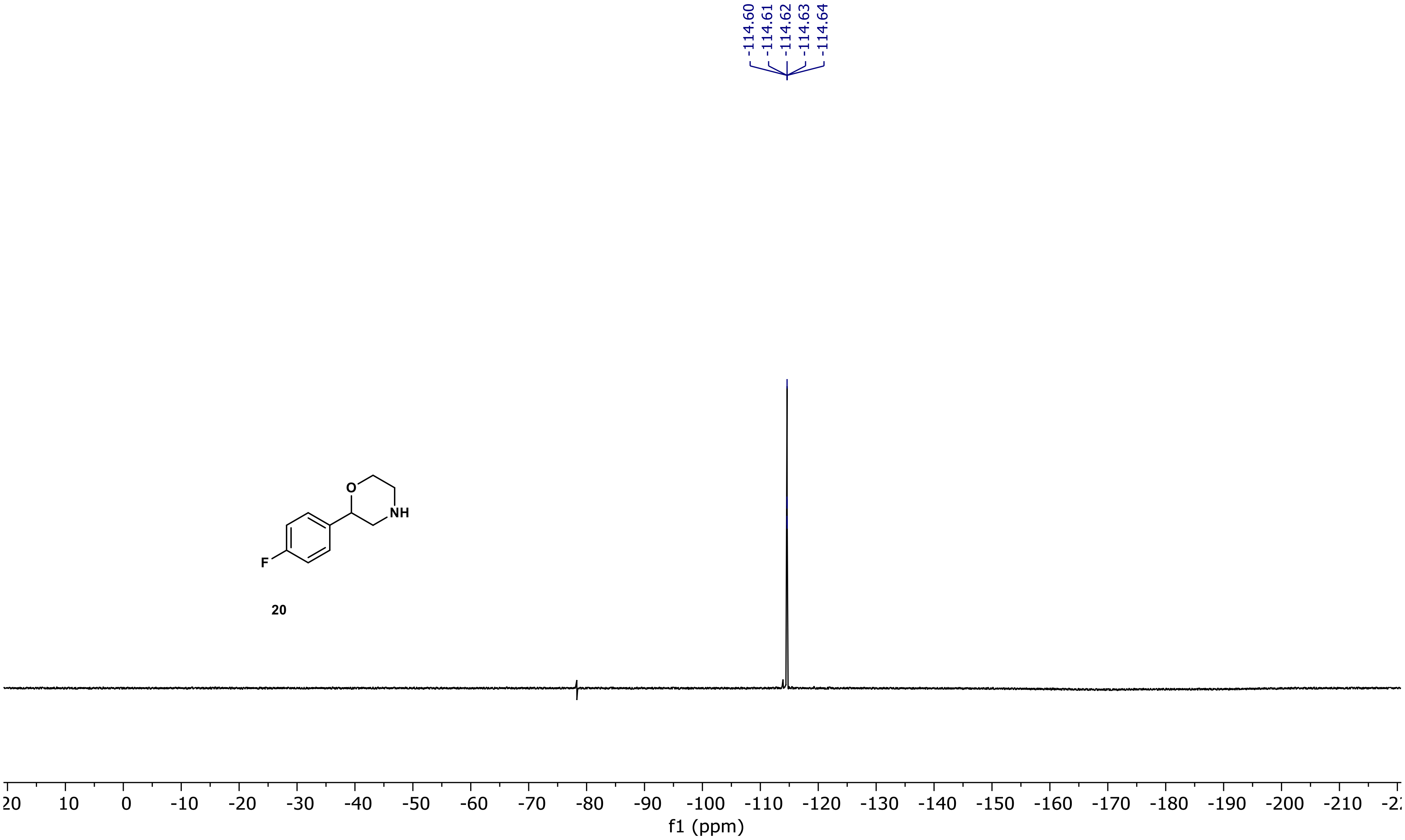

^{1}H NMR of 2,2-diphenylmorpholine 21

CDCl₃, 23 ºC

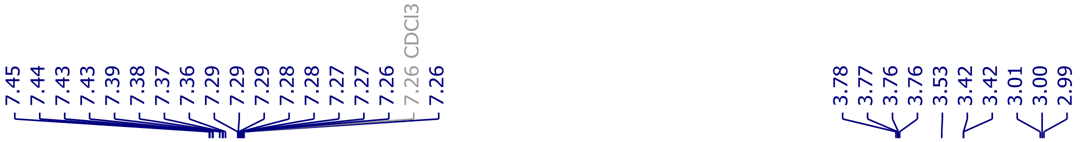

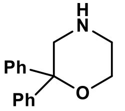

21

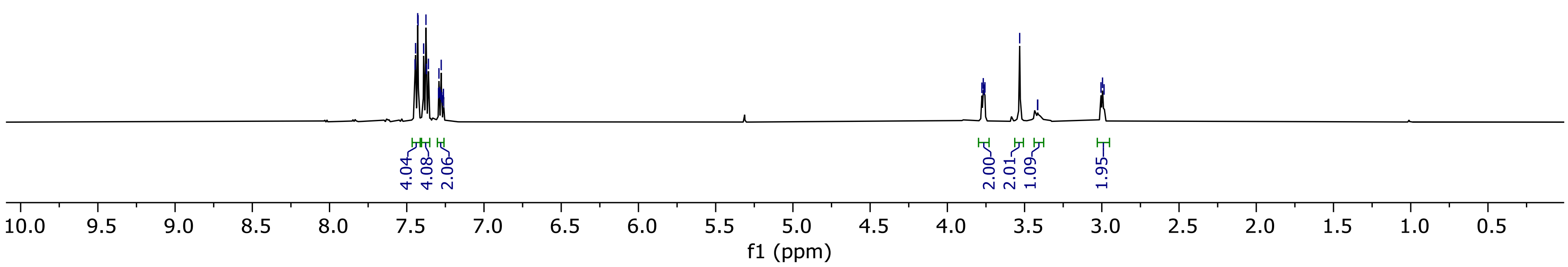

^{13}C NMR of 2,2-diphenylmorpholine 21

CDCl₃, 23 ºC

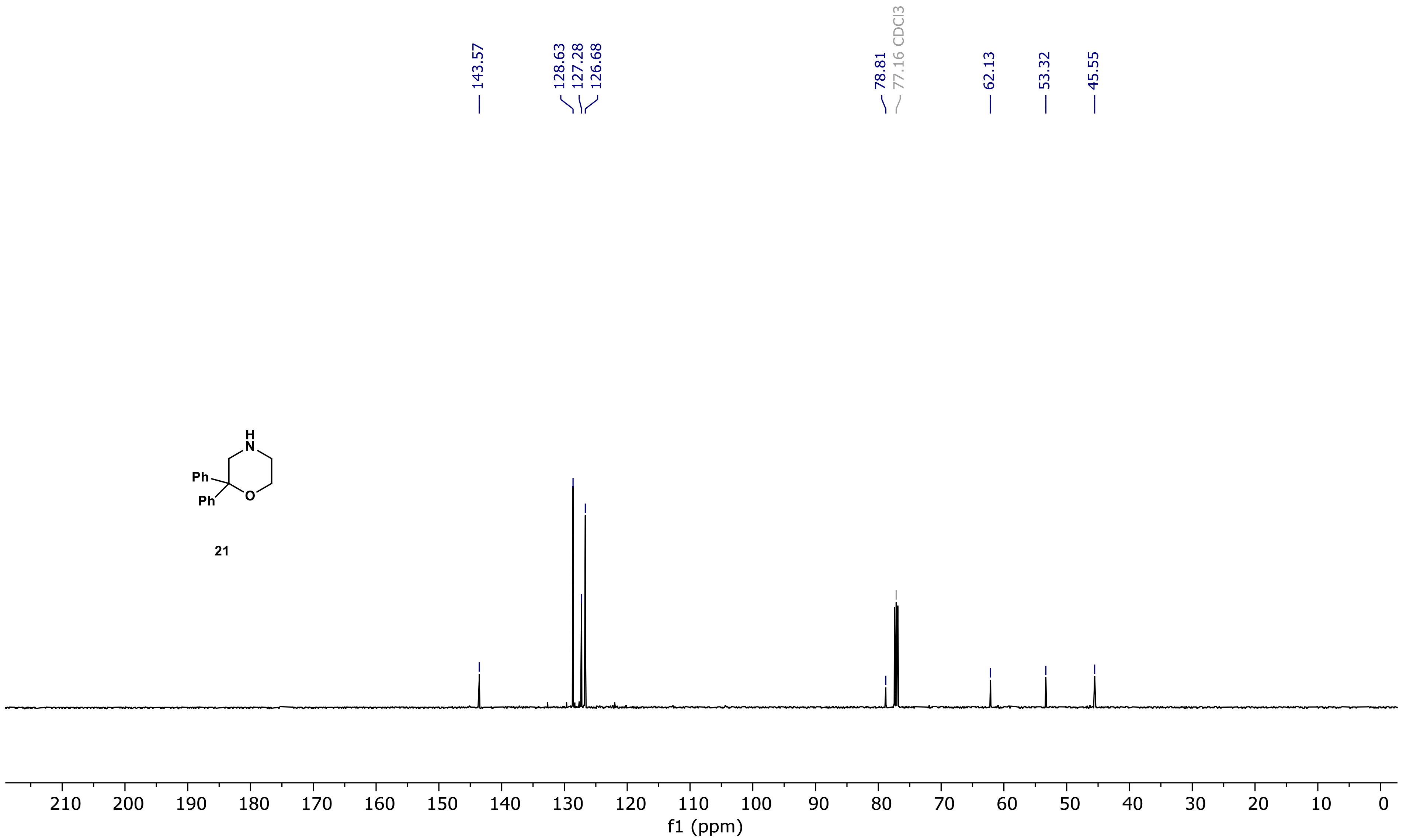

^{1}H NMR of 2-(naphthalen-2-yl)morpholine 22

$CDCl_3$, 23 ºC

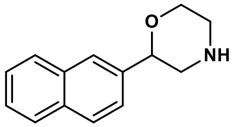

22

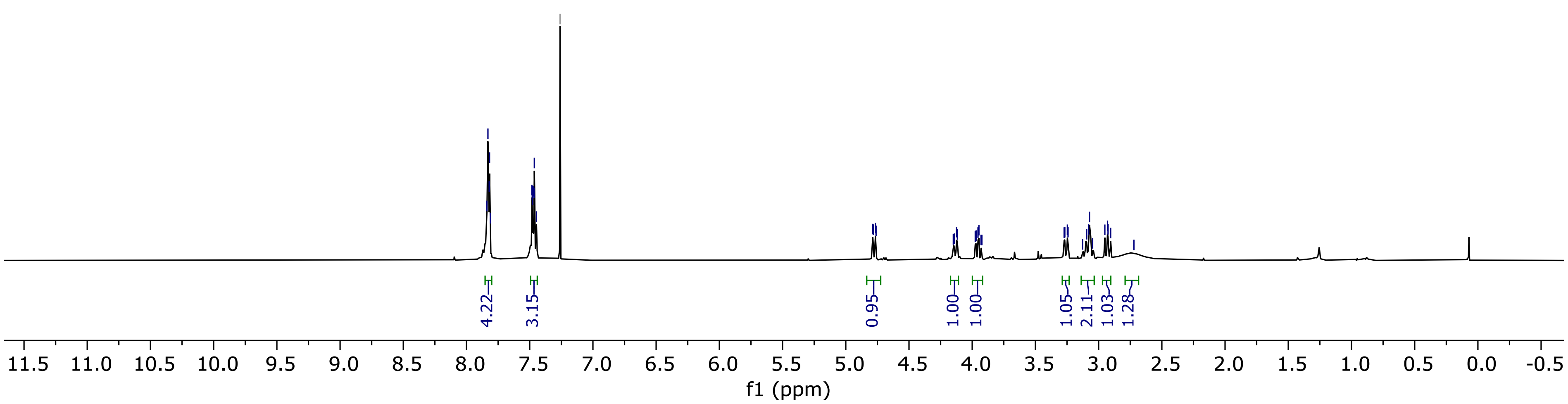

^{13}C NMR of 2-(naphthalen-2-yl)morpholine 22

CDCl3, 23 ºC

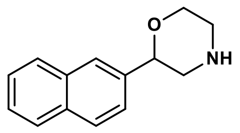

22

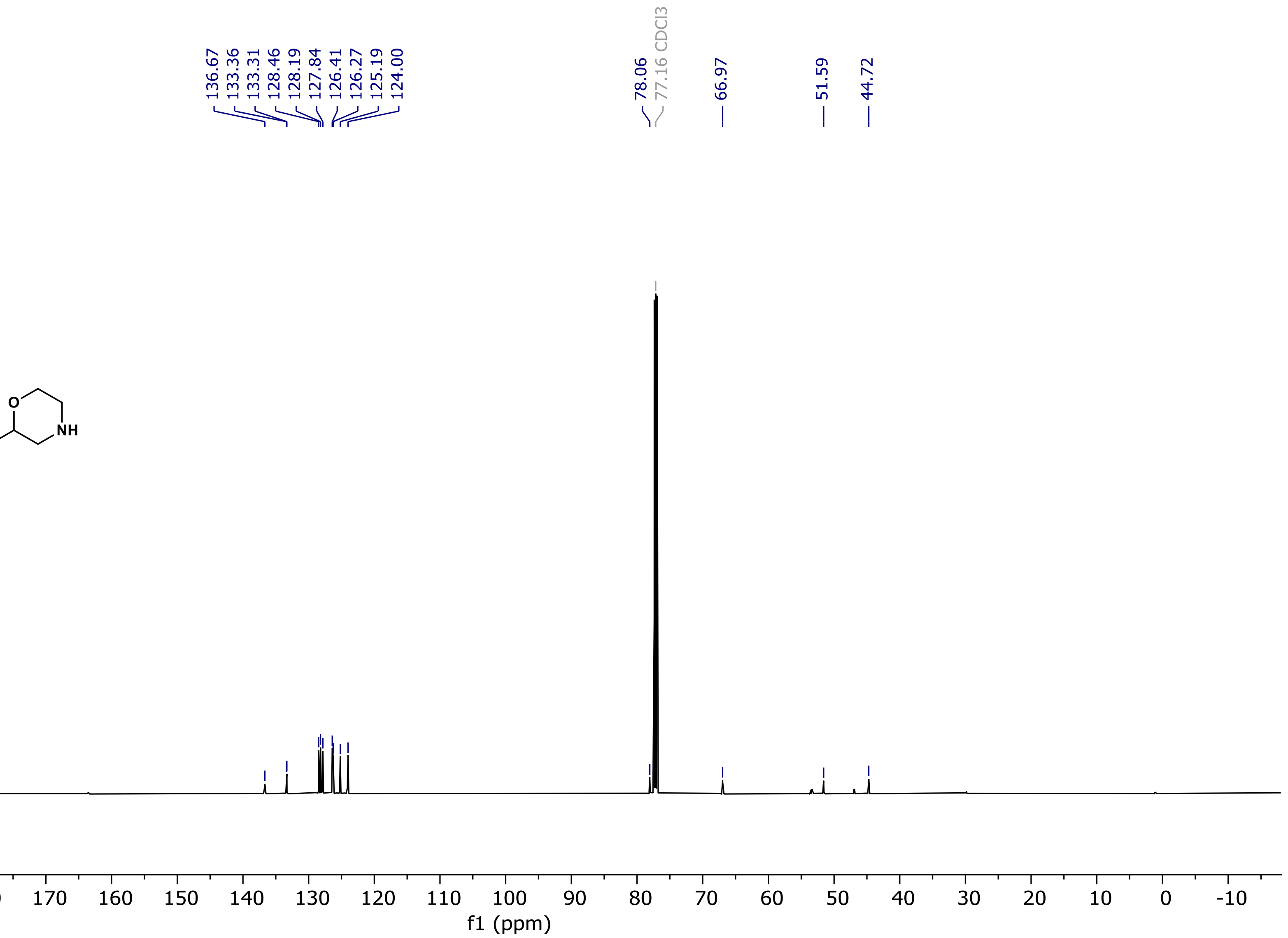

^{1}H NMR of 2-methyl-2-phenylmorpholine 23

CDCl3, 23 ºC

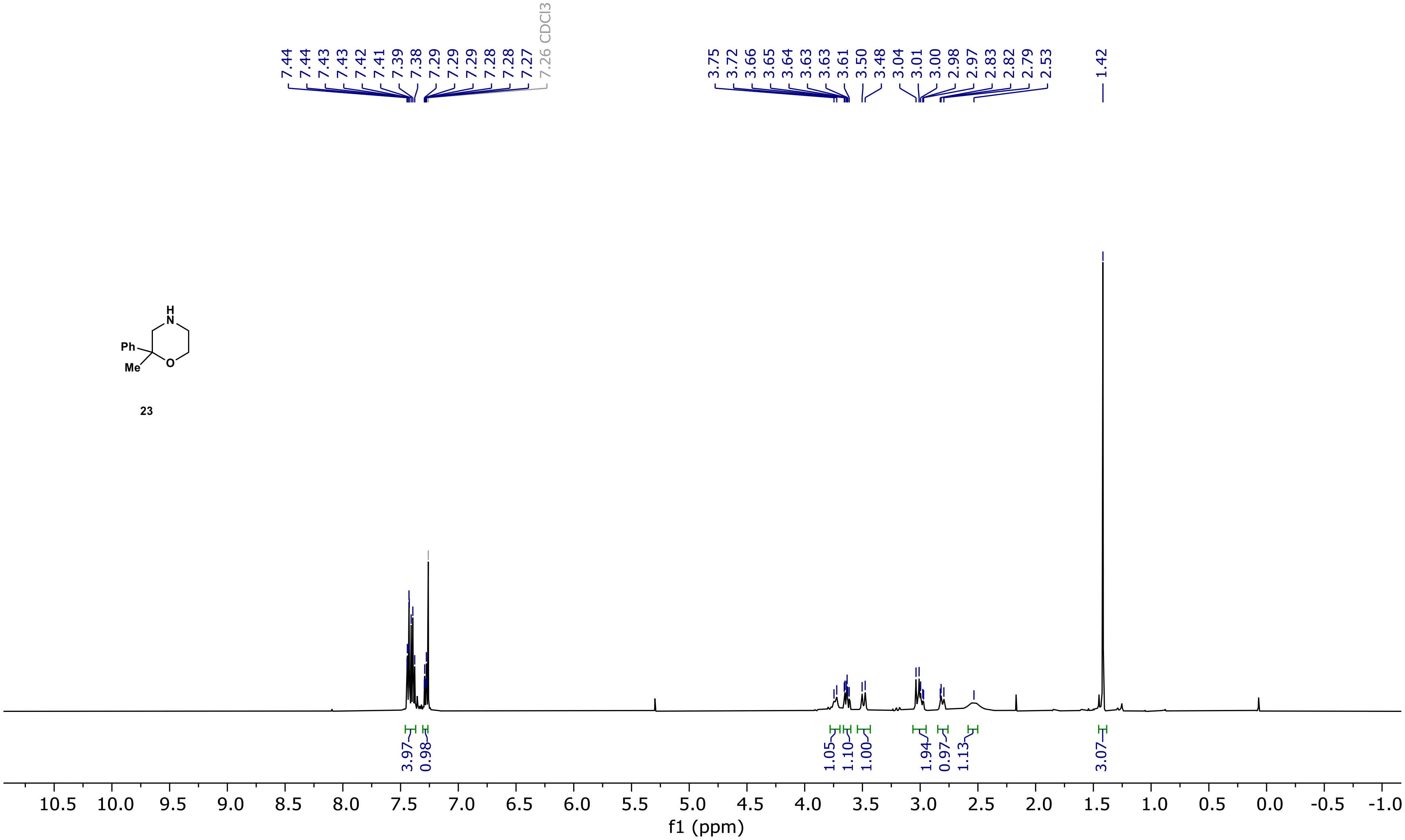

^{13}C NMR of 2-methyl-2-phenylmorpholine 23

$CDCl_3$, 23 ºC

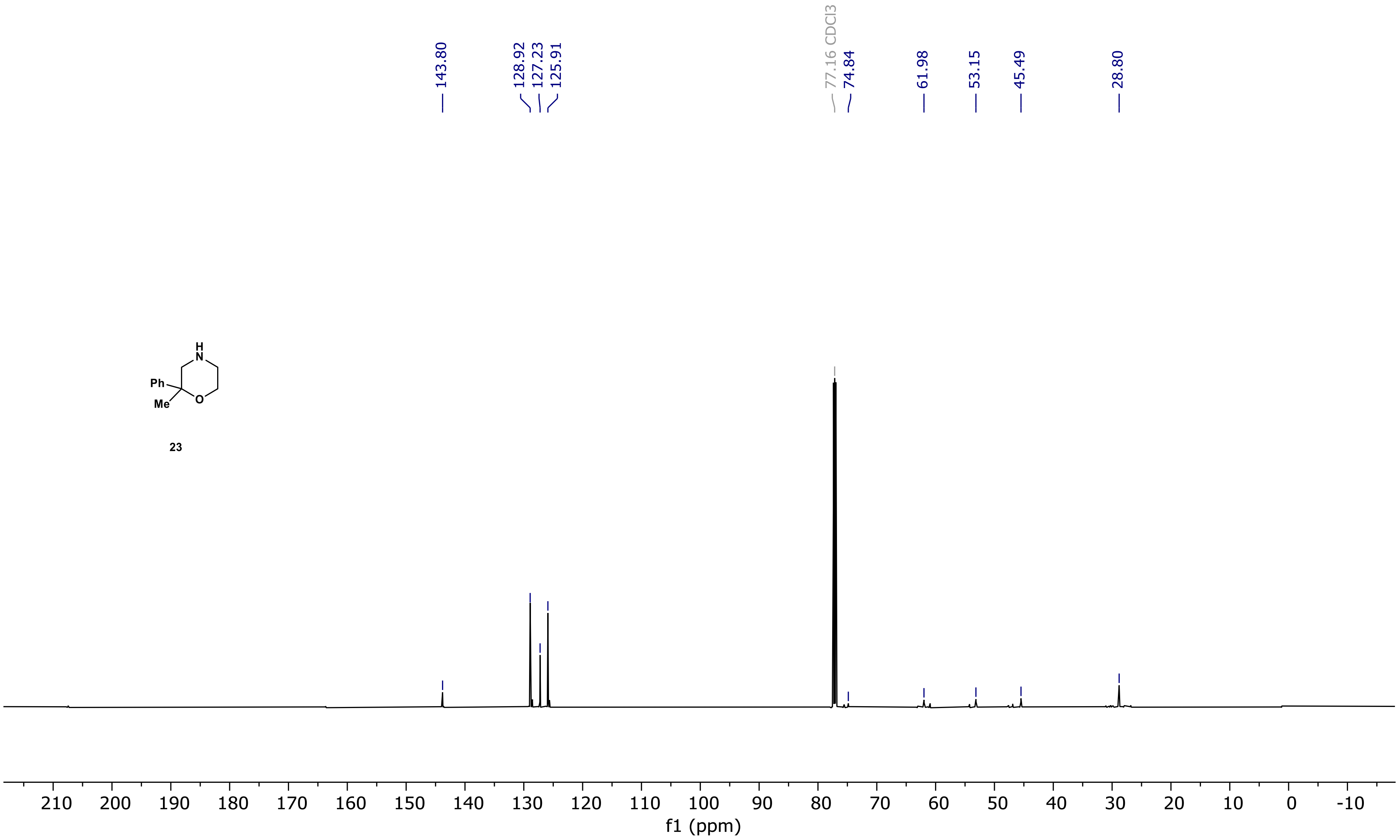

^{1}H NMR of indole-3-morpholine 24

CDCl3, 23 ºC

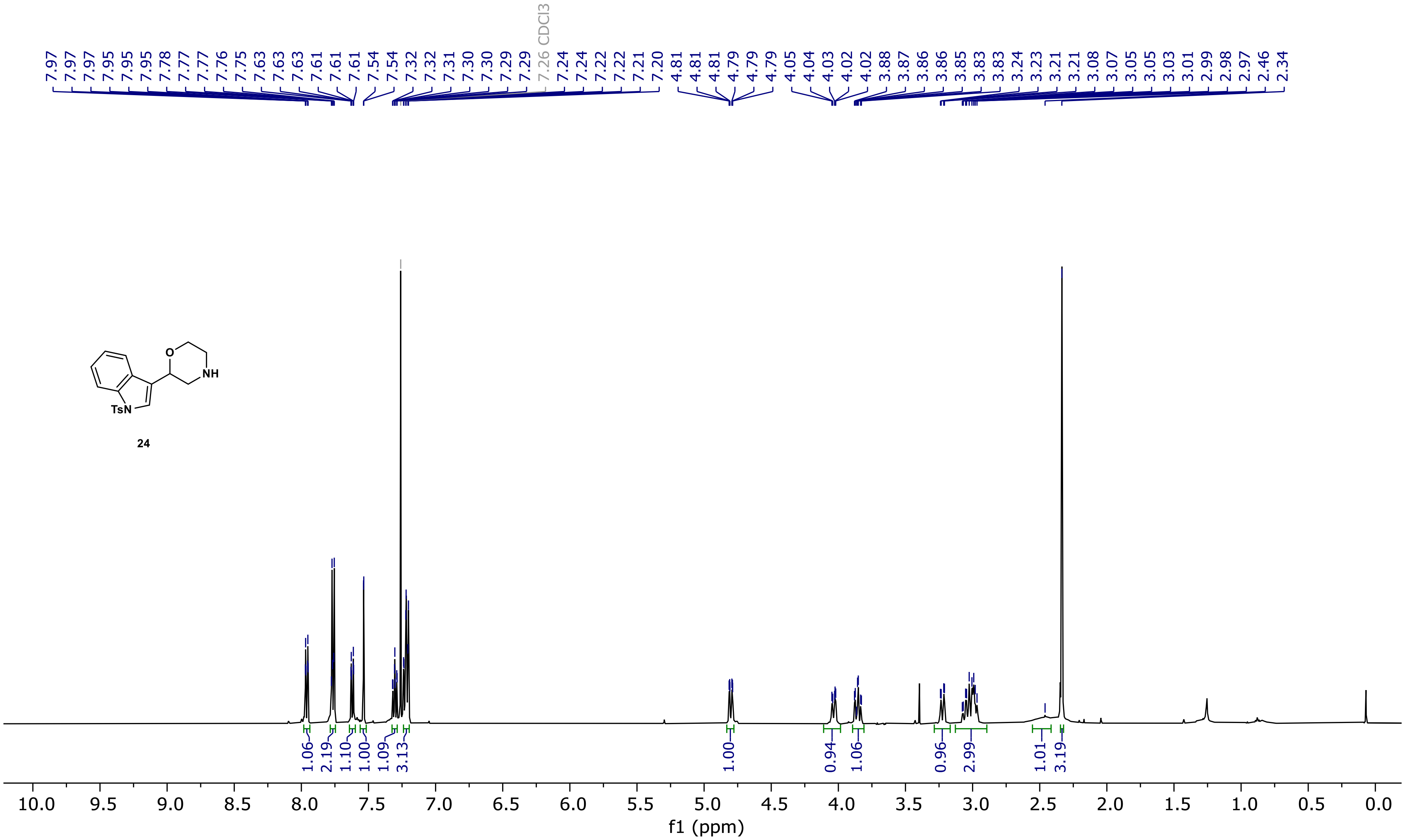

^{13}C NMR of indole-3-morpholine 24

CD_2Cl_2, 23 ºC

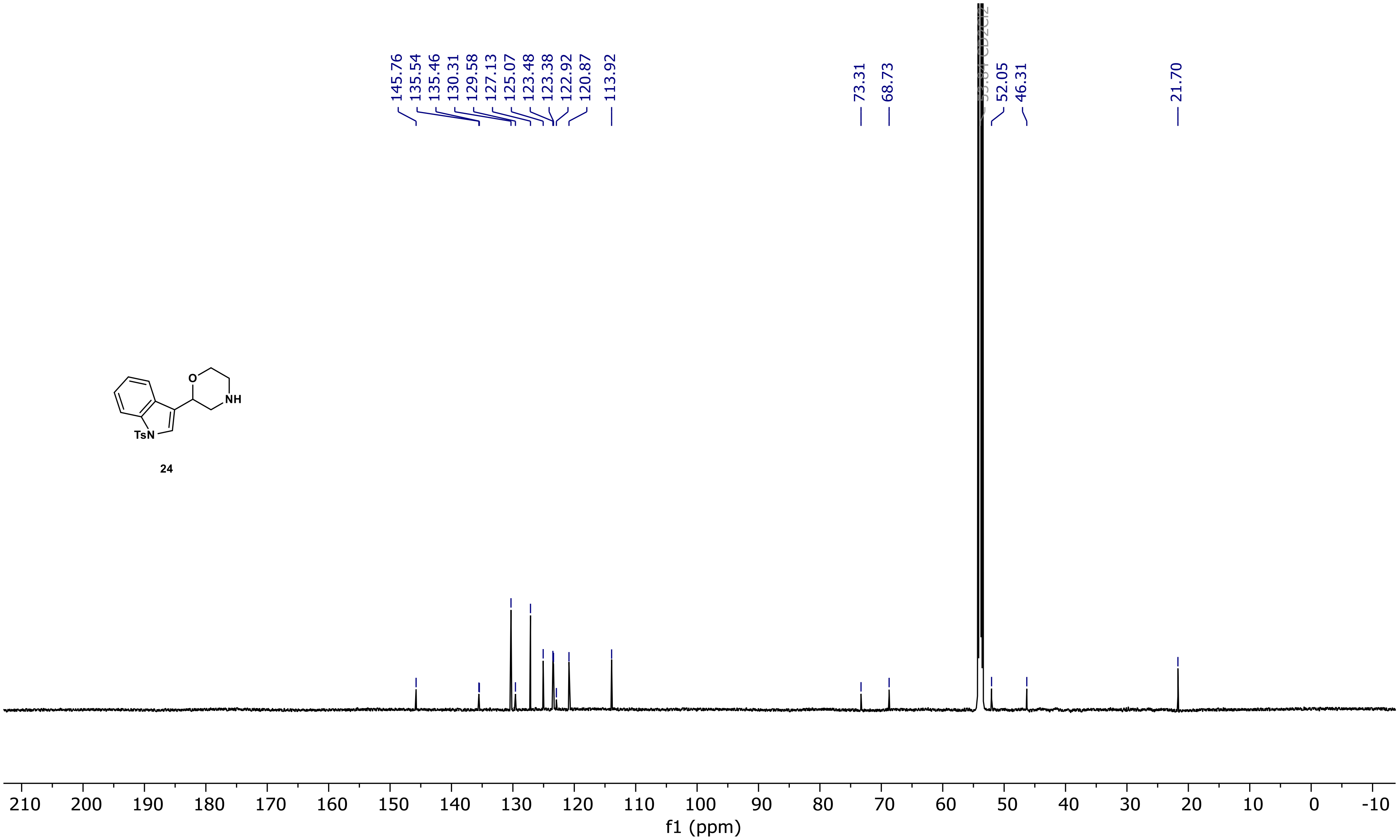

24

^{1}H NMR of 2-(6-methoxypyridin-3-yl)morpholine 25

CDCl$_3$, 23 ºC

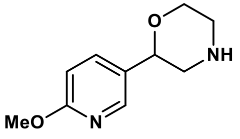

25

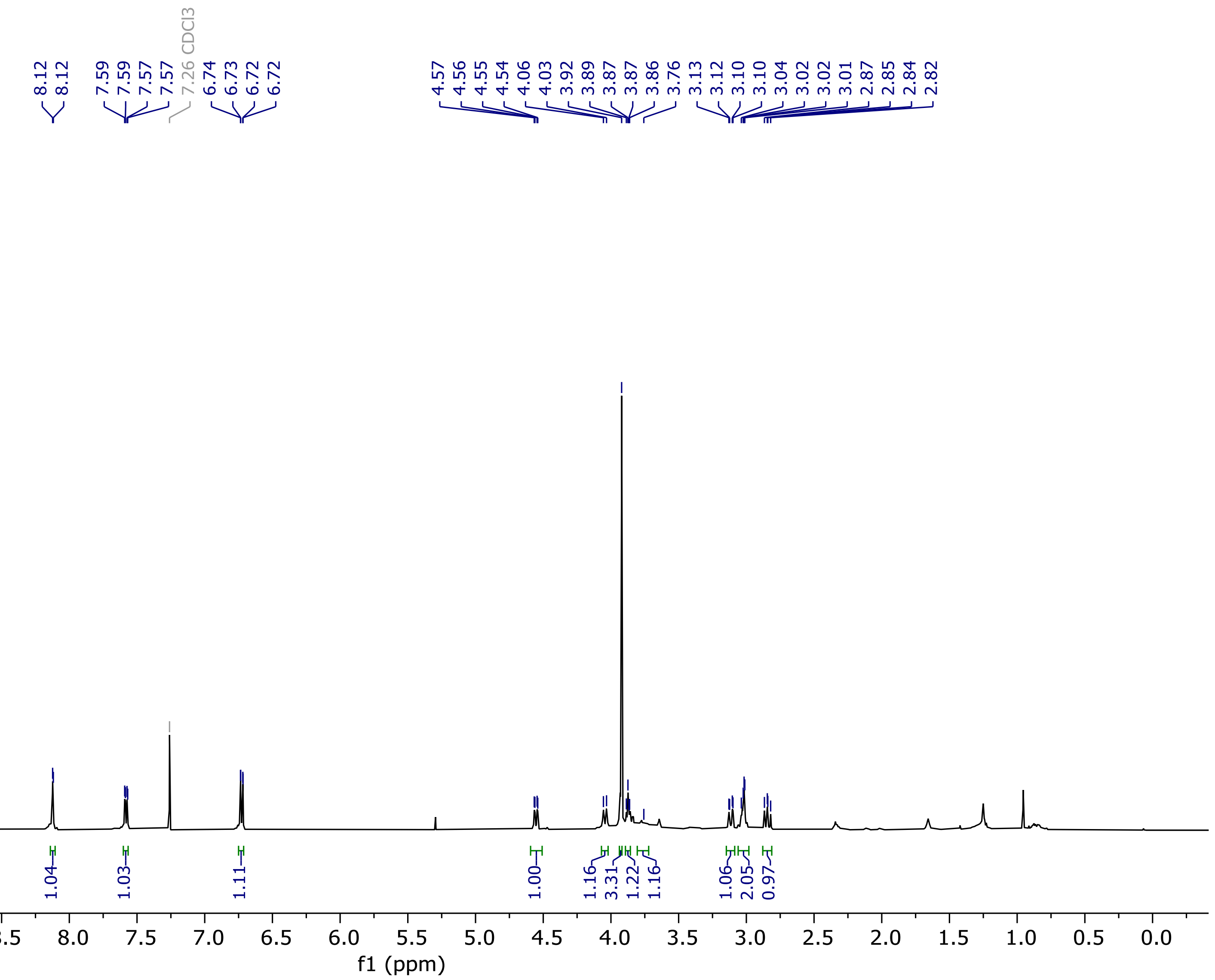

^{13}C NMR of 2-(6-methoxypyridin-3-yl)morpholine 25

CDCl$_3$, 23 ºC

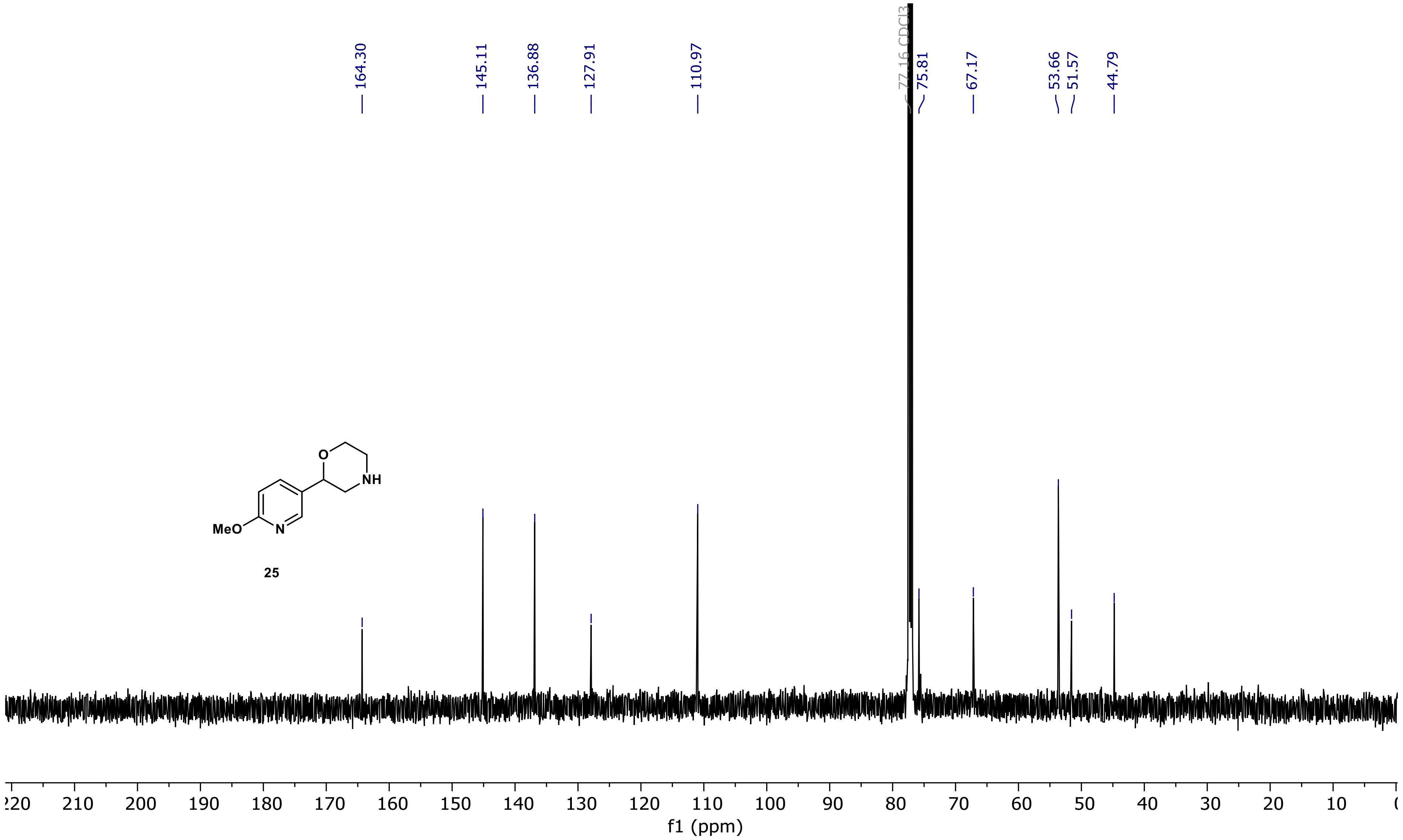

^{1}H NMR of 2,3-diphenylmorpholine 26

CD_3CN, 23 ºC

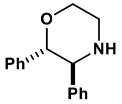

26

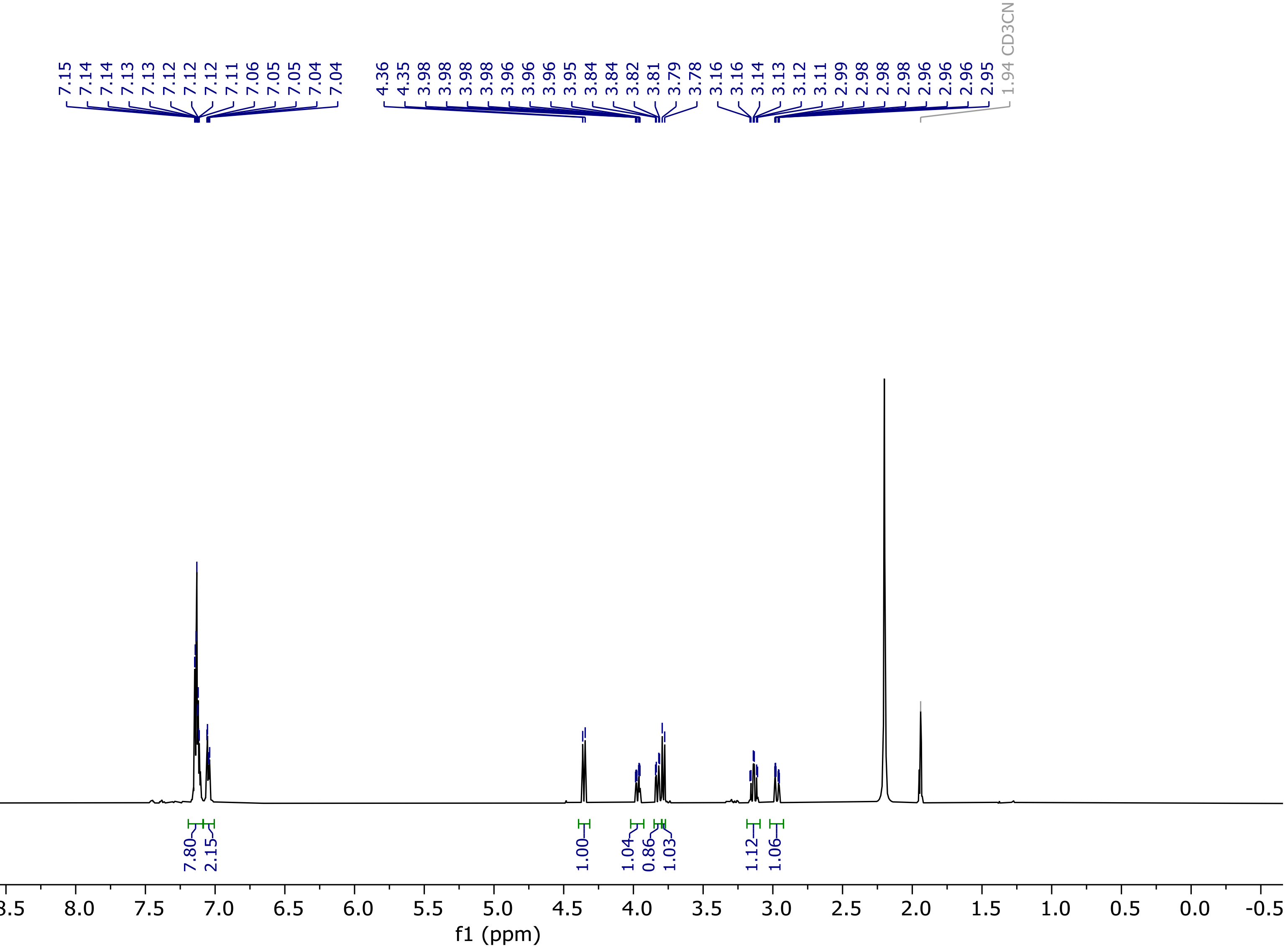

^{13}C NMR of 2,3-diphenylmorpholine 26

CD_3CN, 23 ºC

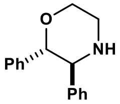

26

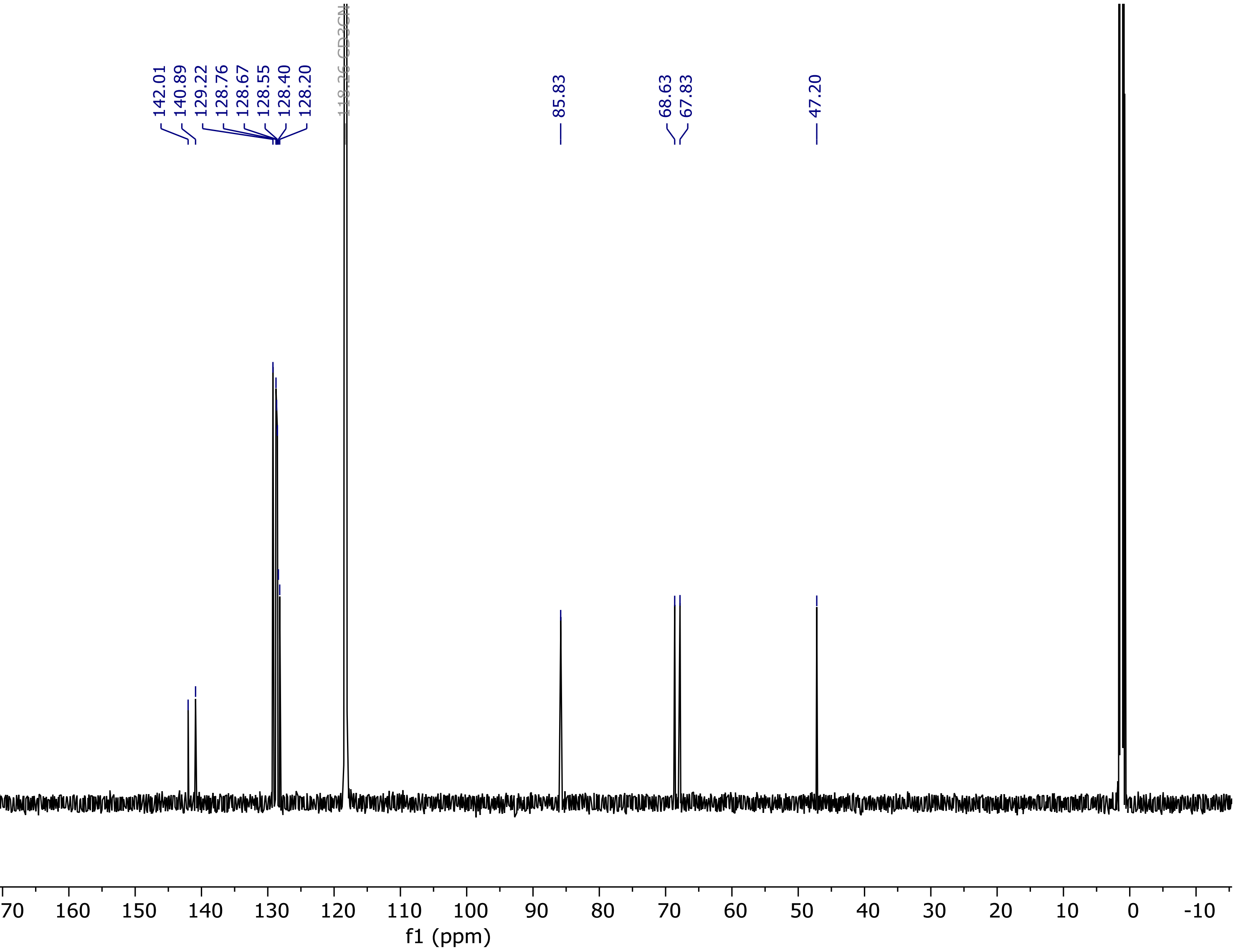

^{1}H NMR of 3-methyl-2-phenylmorpholine 27

$CDCl_3$, 23 ºC

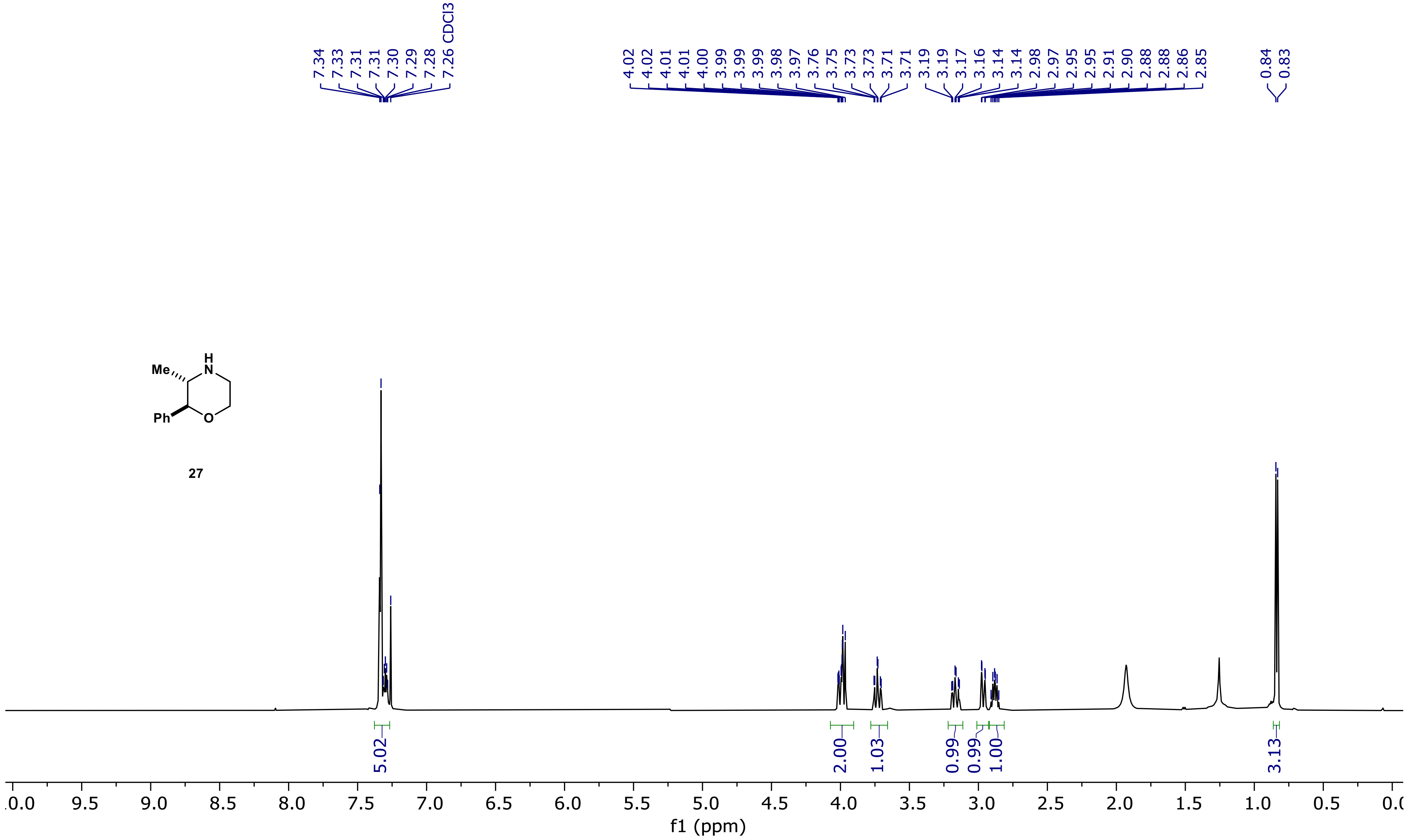

^{13}C NMR of 3-methyl-2-phenylmorpholine 27

CDCl$_3$, 23 ºC

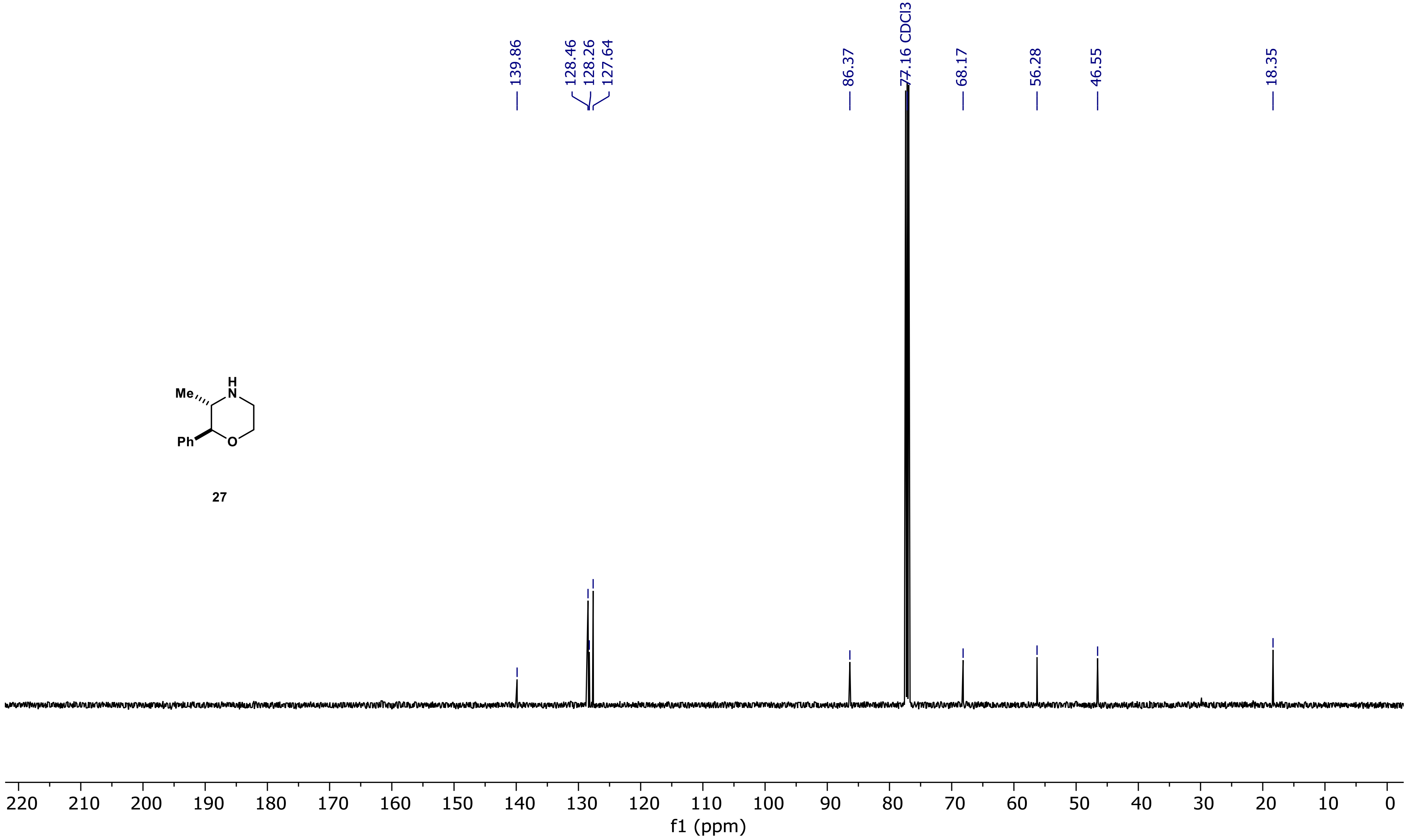

27

^{1}H NMR of 2-(benzothiophen-3-yl)morpholine 28

CDCl$_3$, 23 ºC

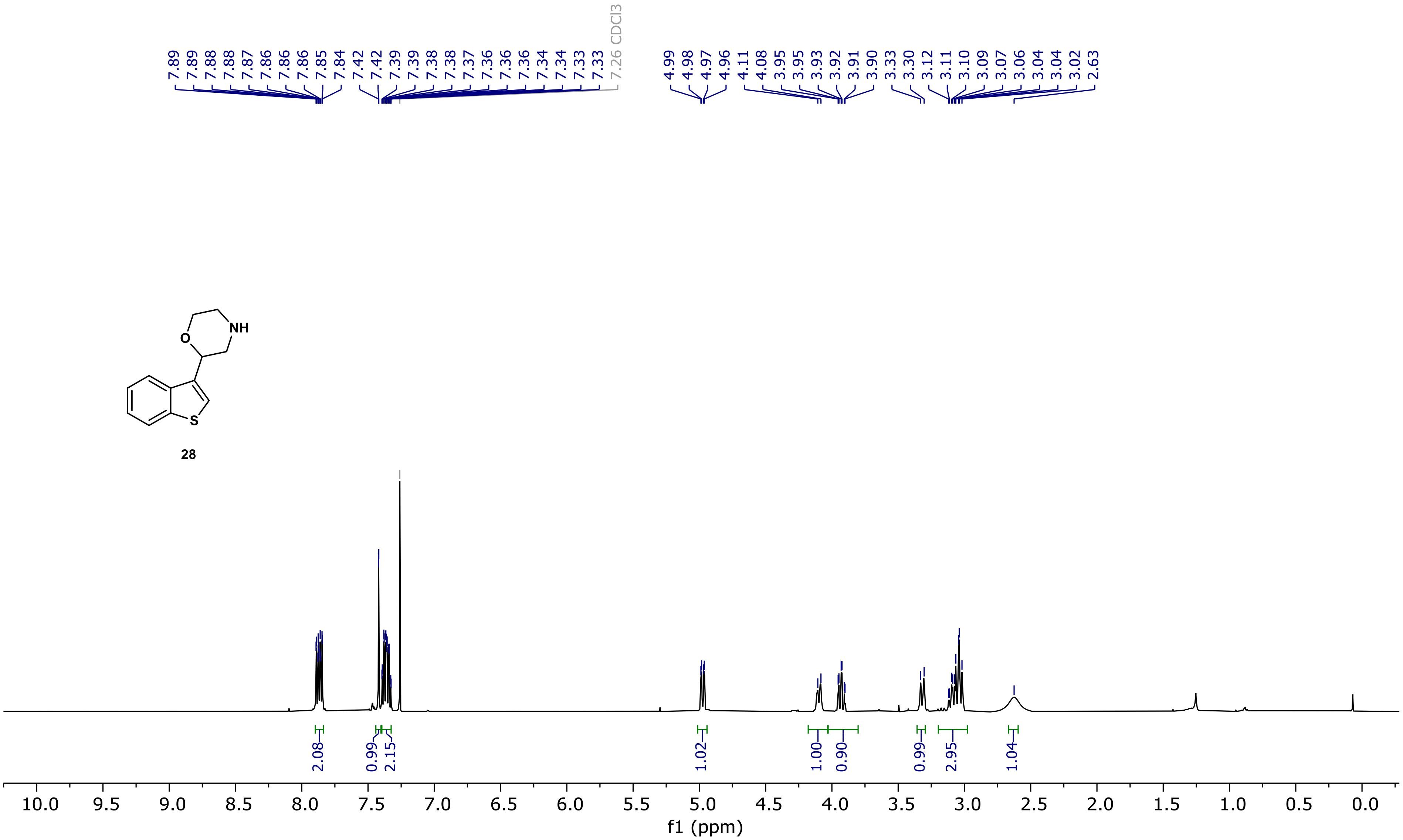

^{13}C NMR of 2-(benzothiophen-3-yl)morpholine 28

CDCl3, 23 ºC

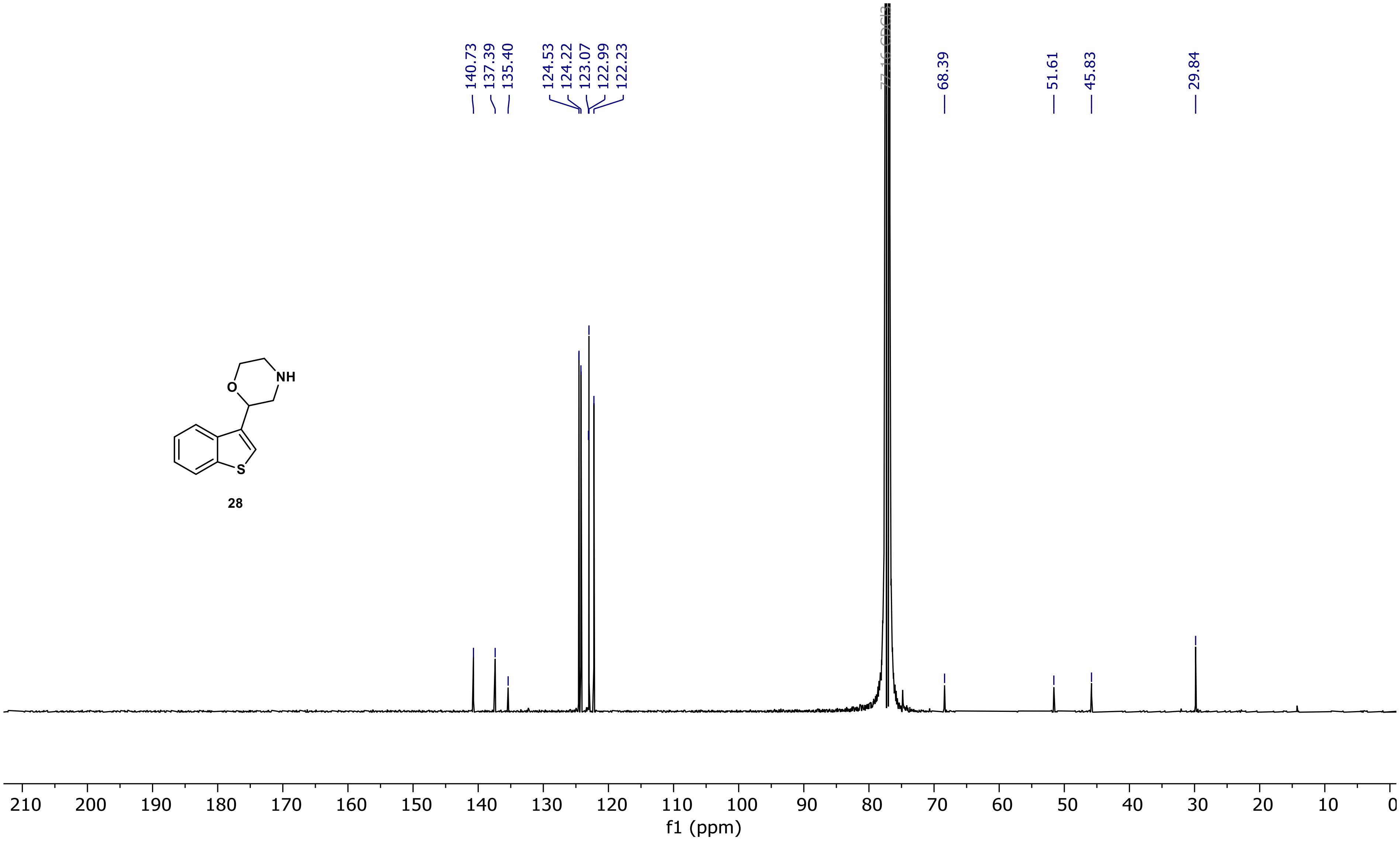

^{1}H NMR of bicyclo[2.2.1]heptane derived morpholine 29

CDCl$_3$, 23 ºC

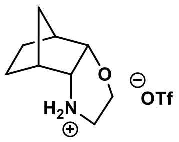

29

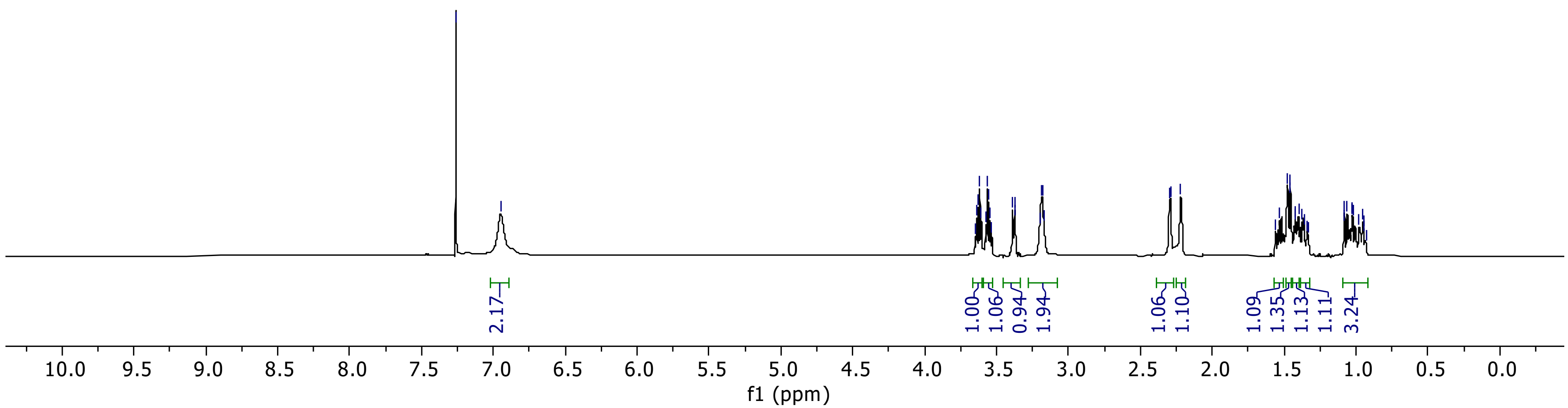

^{13}C NMR of bicyclo[2.2.1]heptane derived morpholine 29

CD3CN, 23 ºC

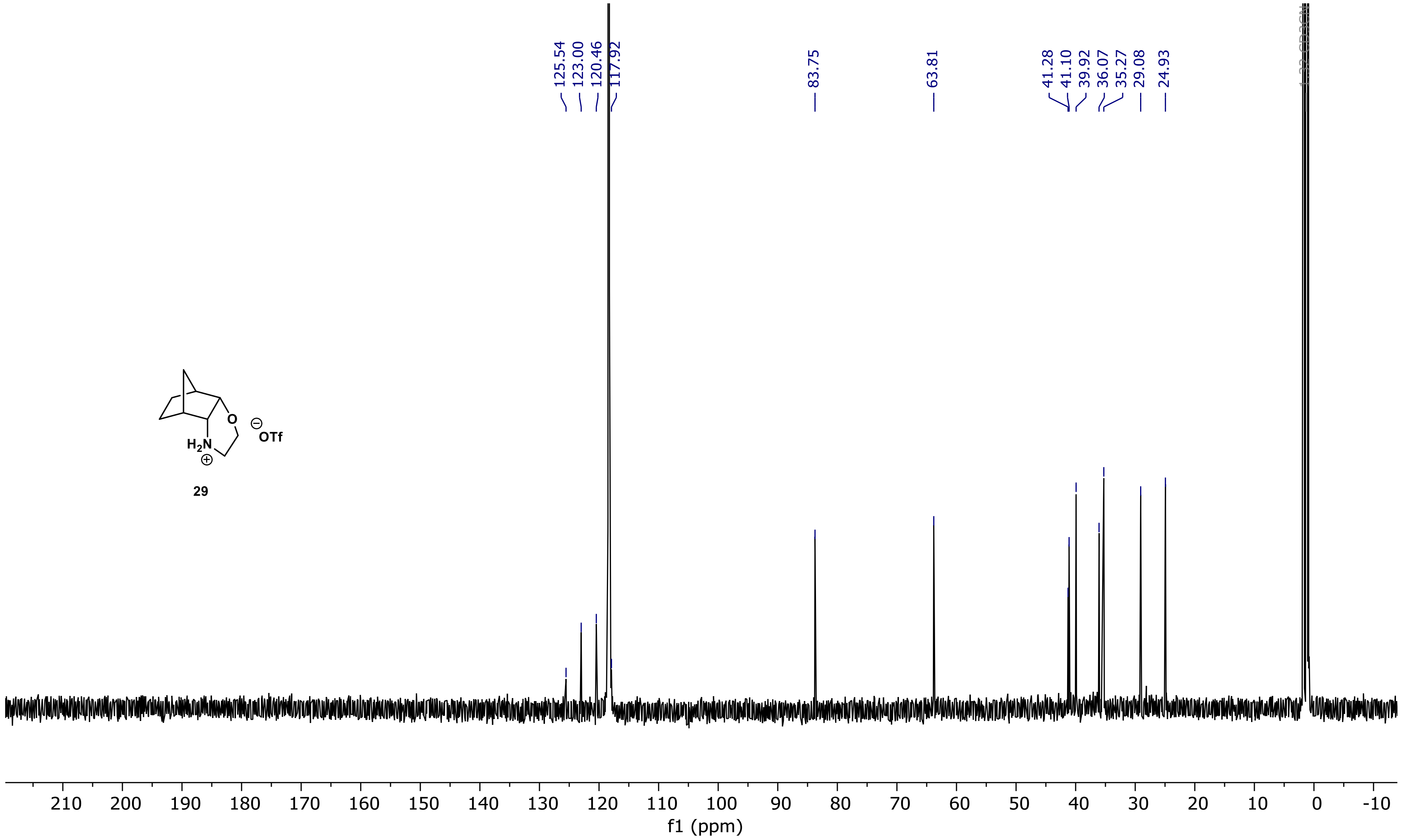

^{19}F NMR of bicyclo[2.2.1]heptane derived morpholine 29

CDCl$_3$, 23 ºC

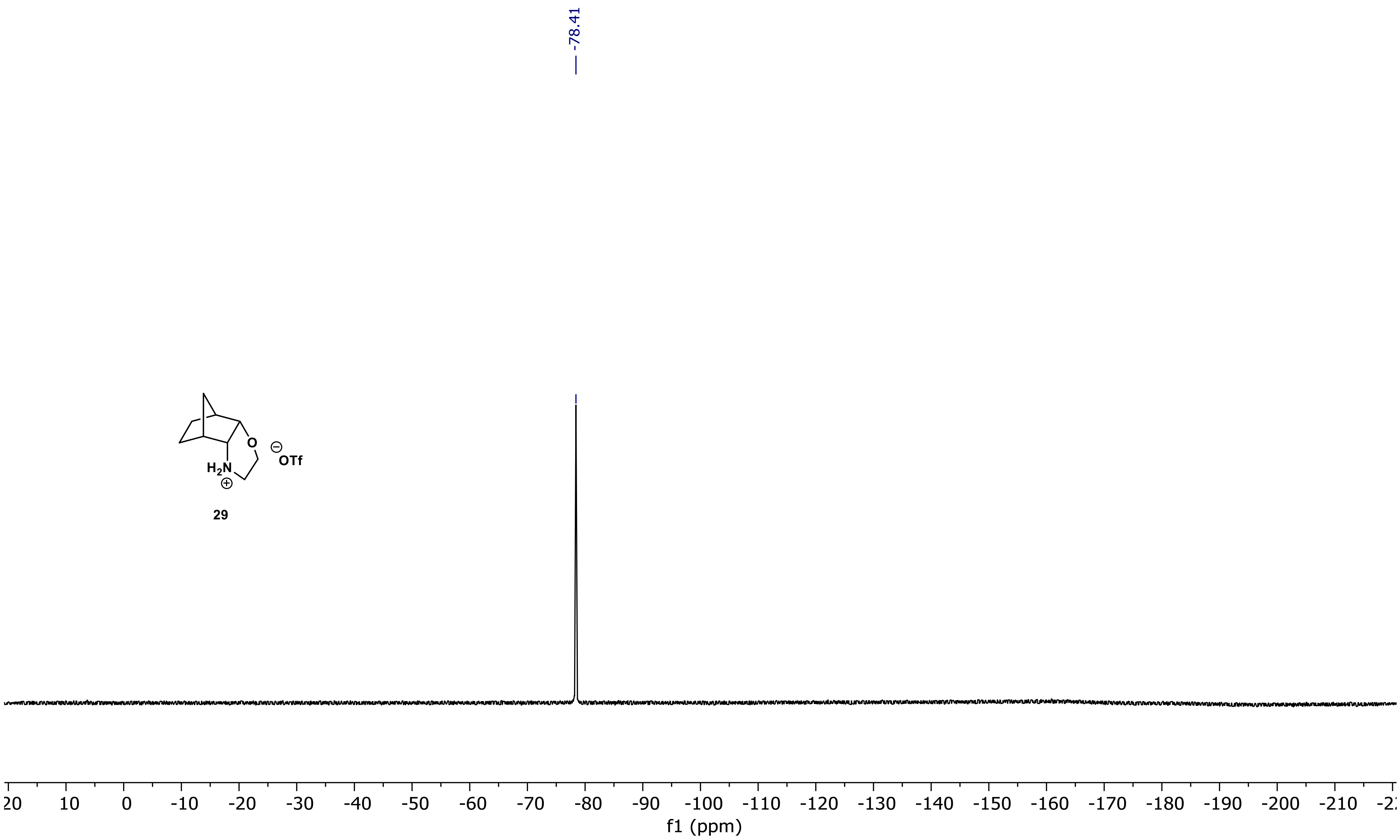

COSY of bicyclo[2.2.1]heptane derived morpholine 29

$CDCl_3$, 23 ºC

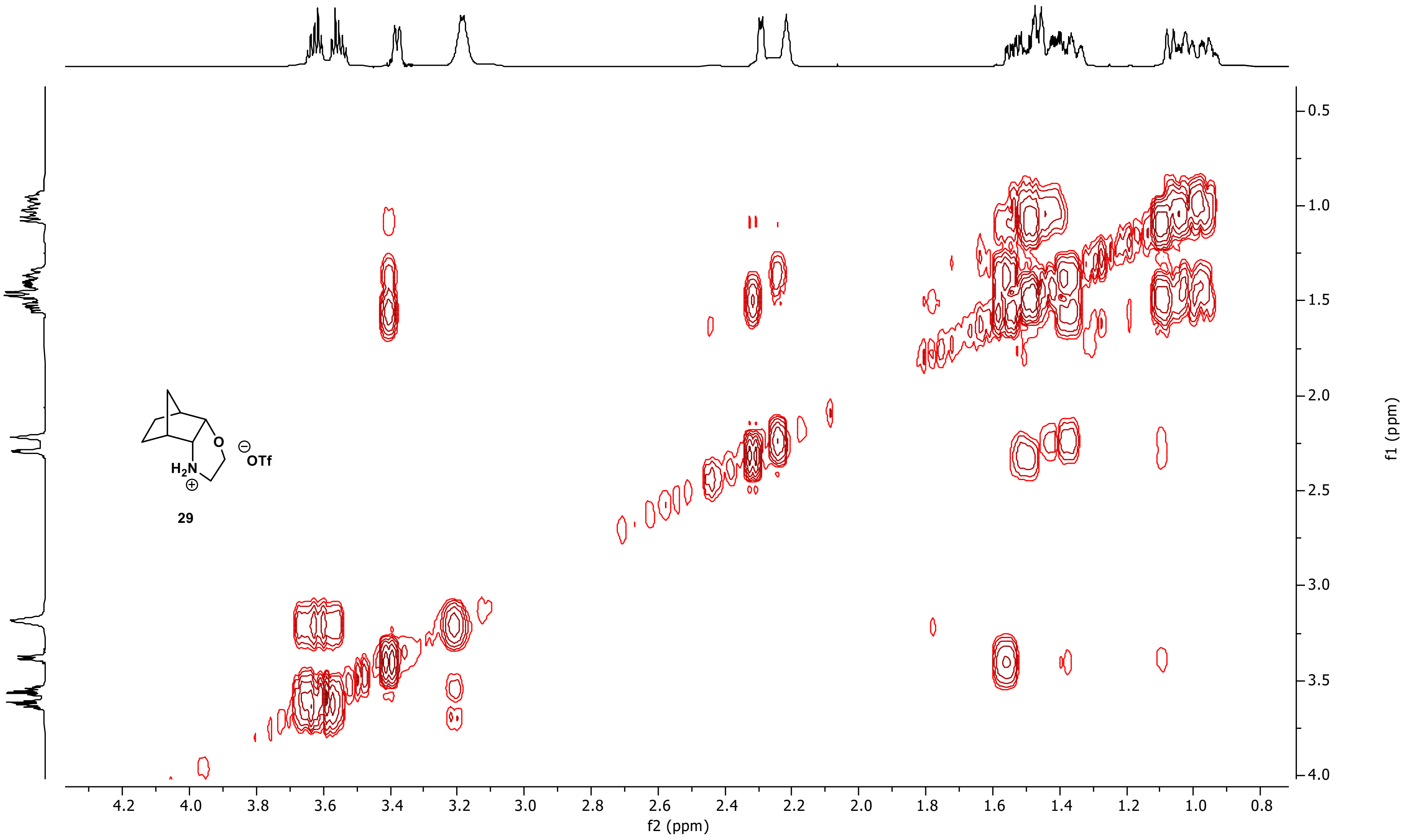

HSQC of bicyclo[2.2.1]heptane derived morpholine 29

$CDCl_3$, 23 ºC

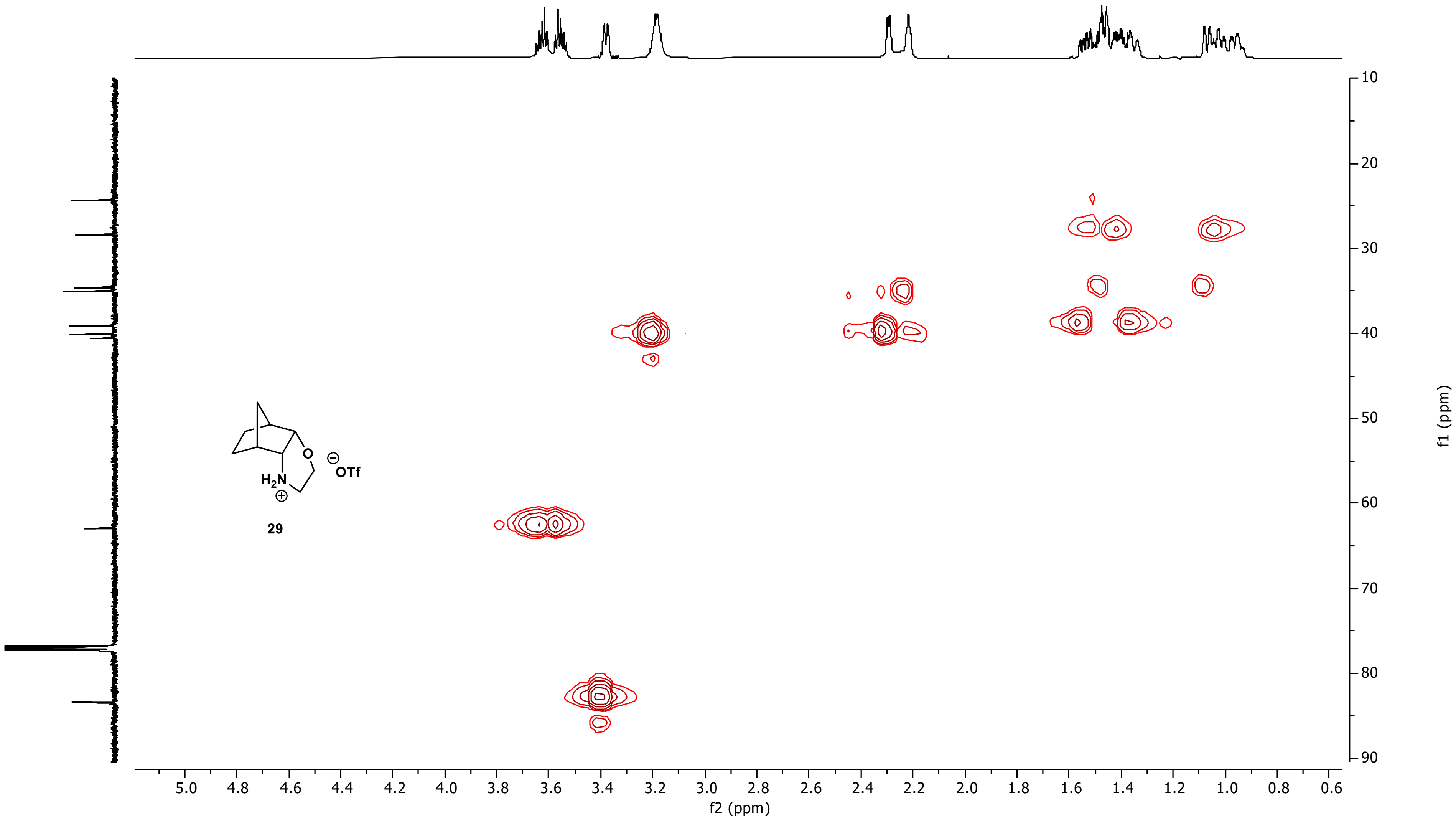

HMBC of bicyclo[2.2.1]heptane derived morpholine 29

$CDCl_3$, 23 ºC

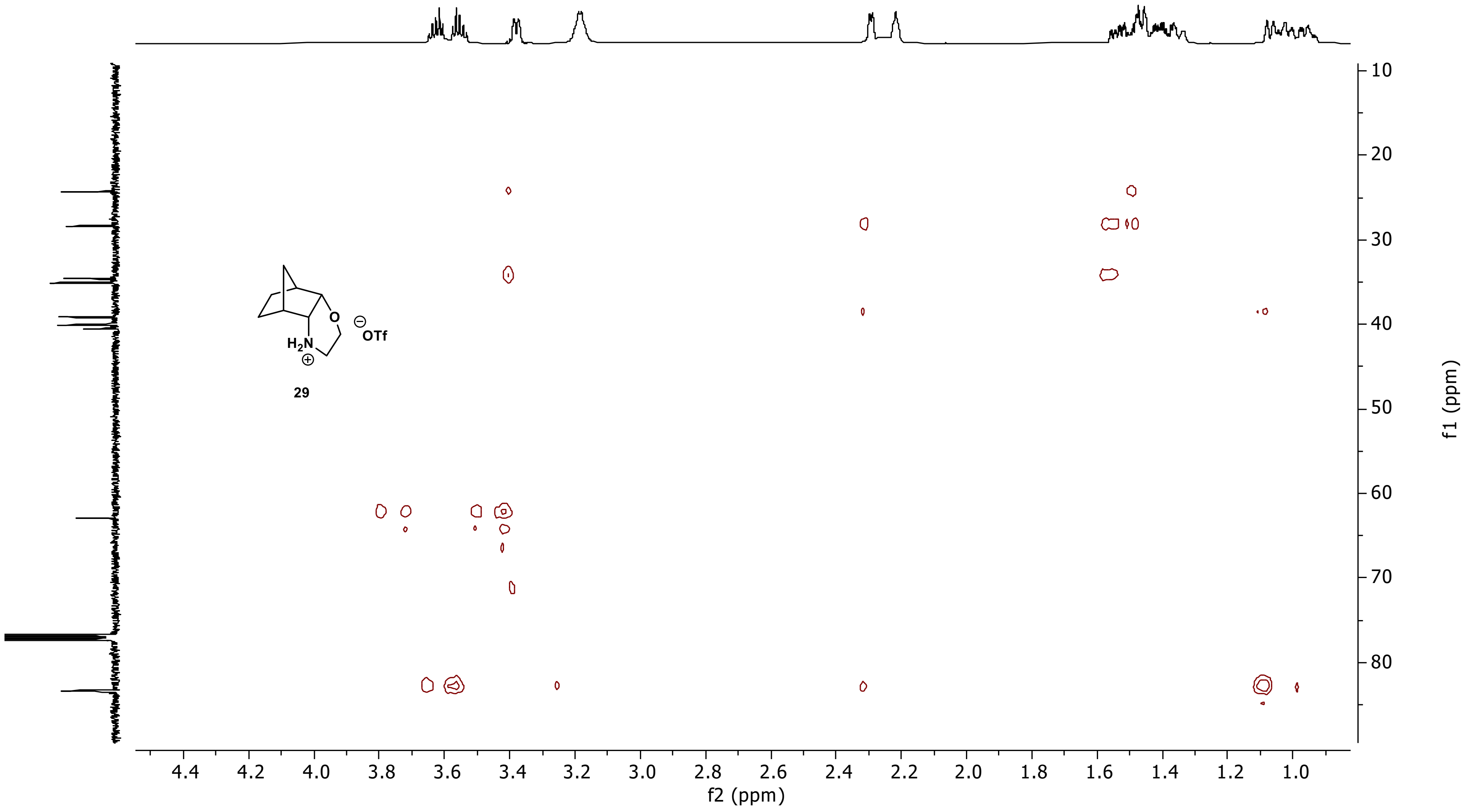

NOESY of bicyclo[2.2.1]heptane derived morpholine 29

$CDCl_3$, 23 ºC

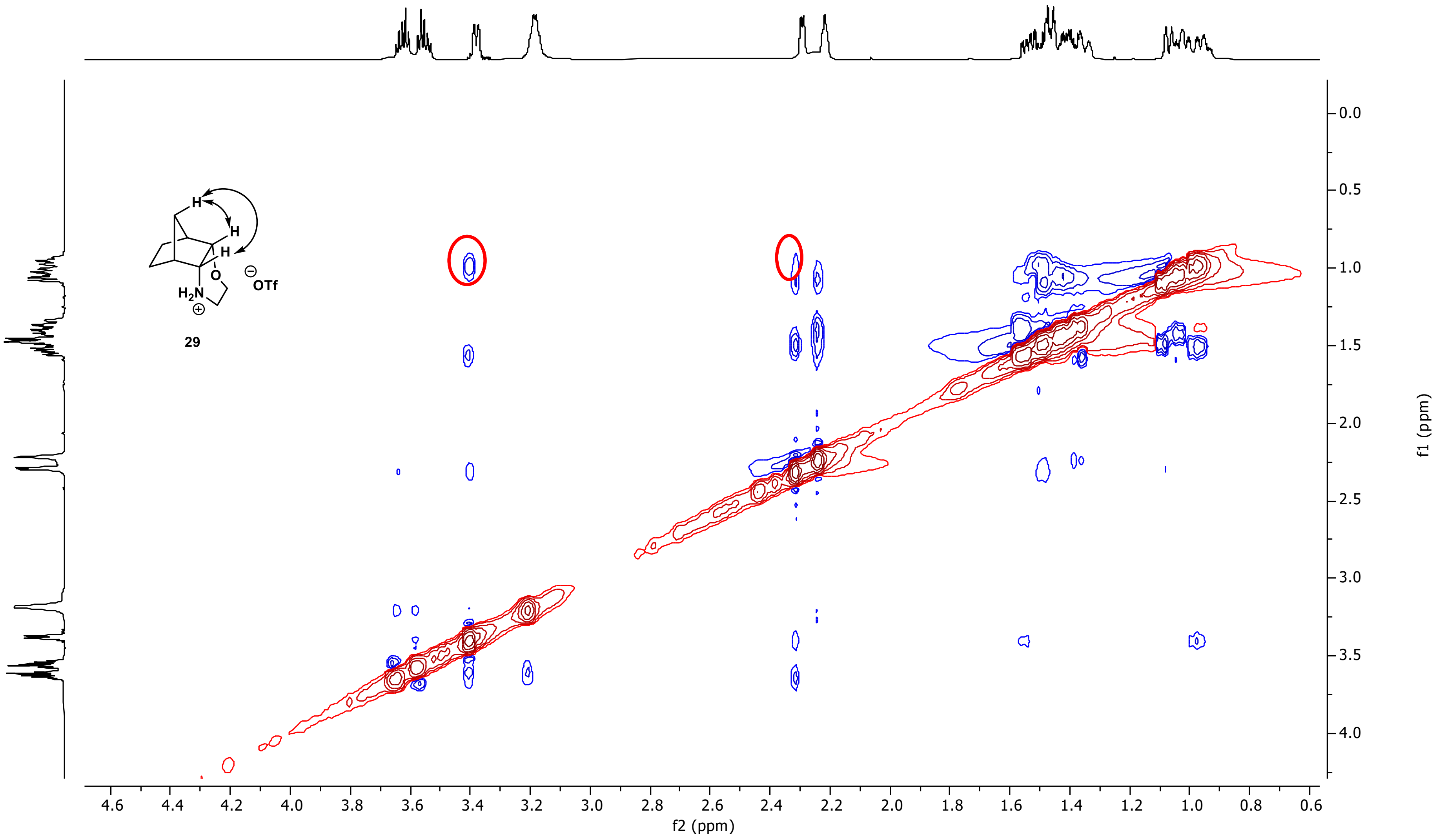

^{1}H NMR of octahydrobenzo[1,4]oxazine 30

CDCl$_3$, 23 ºC

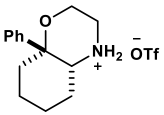

30

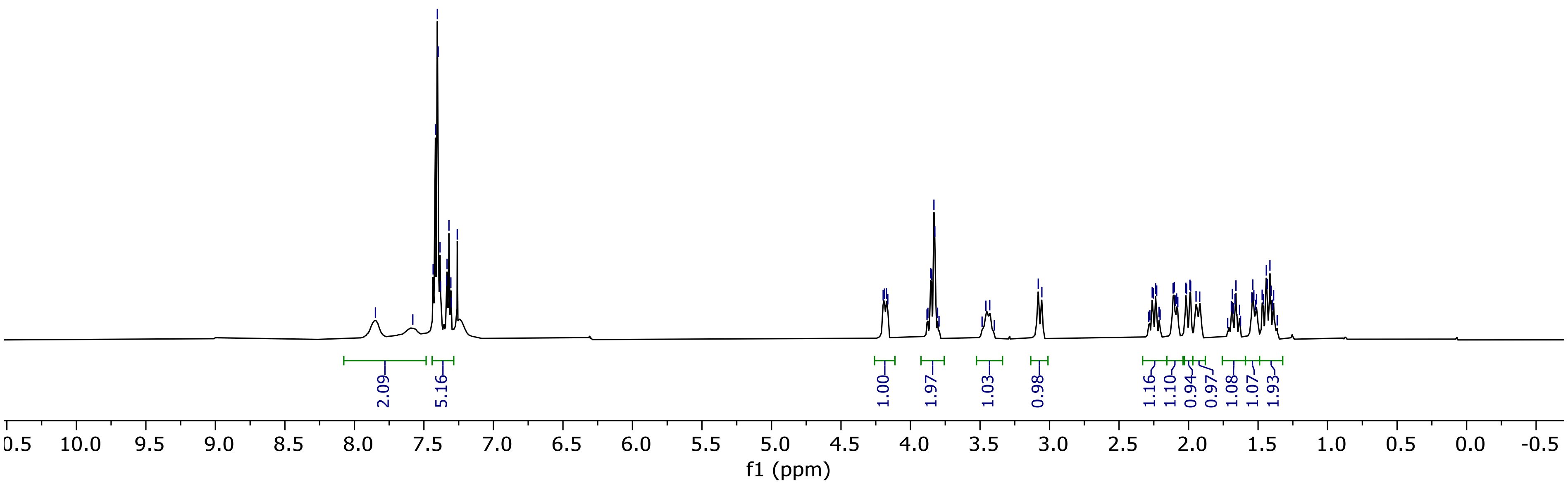

^{13}C NMR of octahydrobenzo[1,4]oxazine 30

$CDCl_3$, 23 ºC

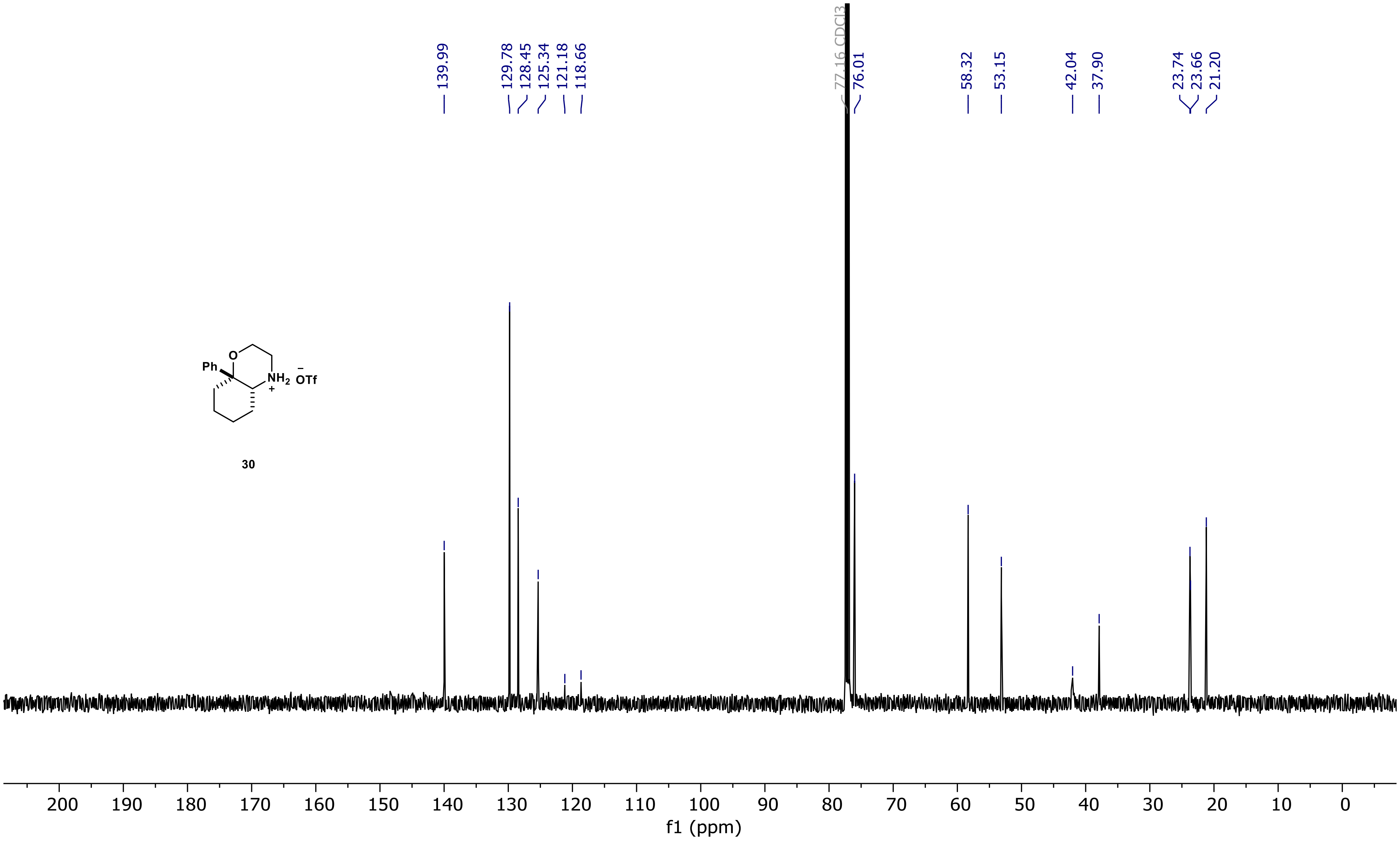

^{19}F NMR of octahydrobenzo[1,4]oxazine 30

$CDCl_3$, 23 ºC

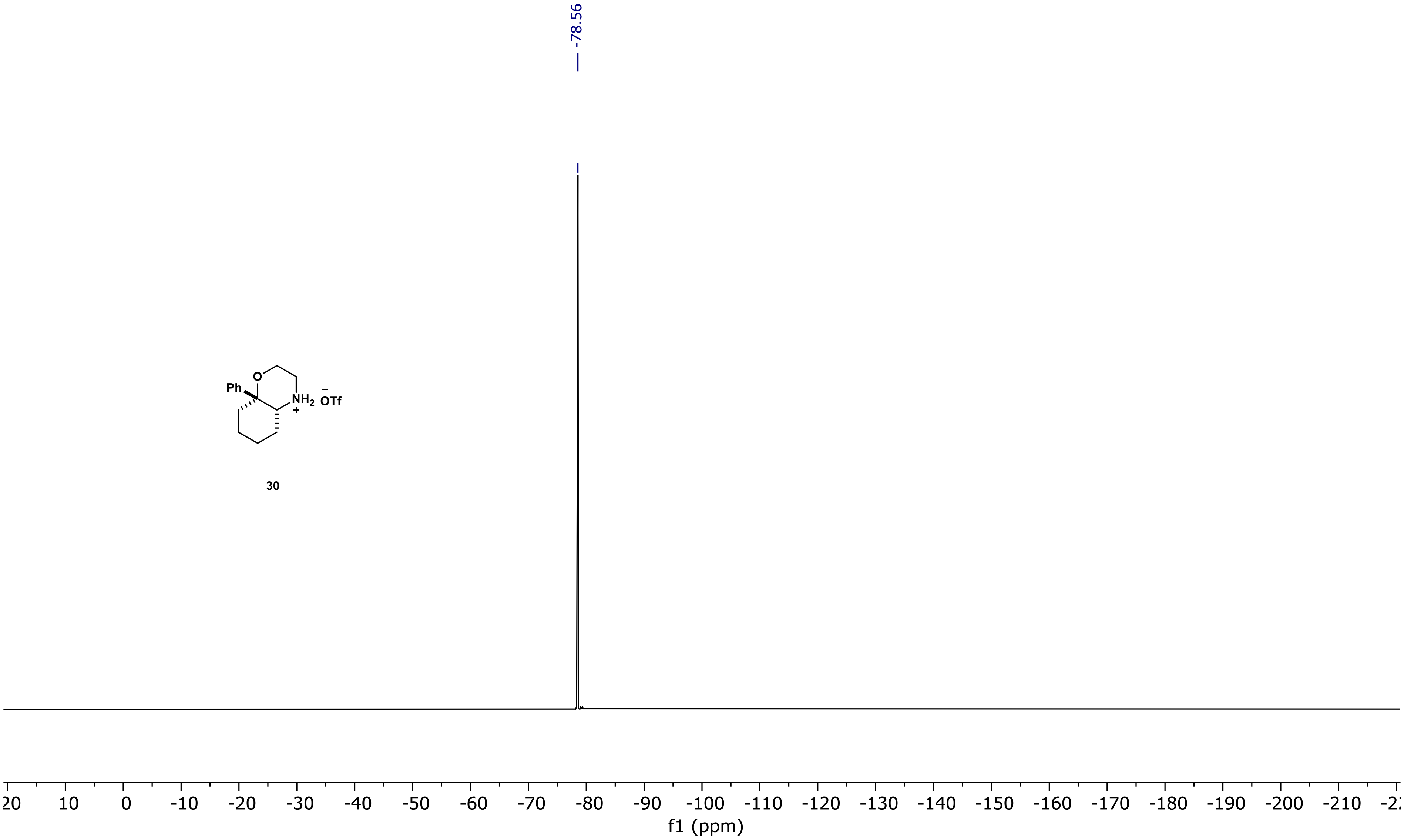

COSY of octahydrobenzo[1,4]oxazine 30

CD_3CN, 23 ºC

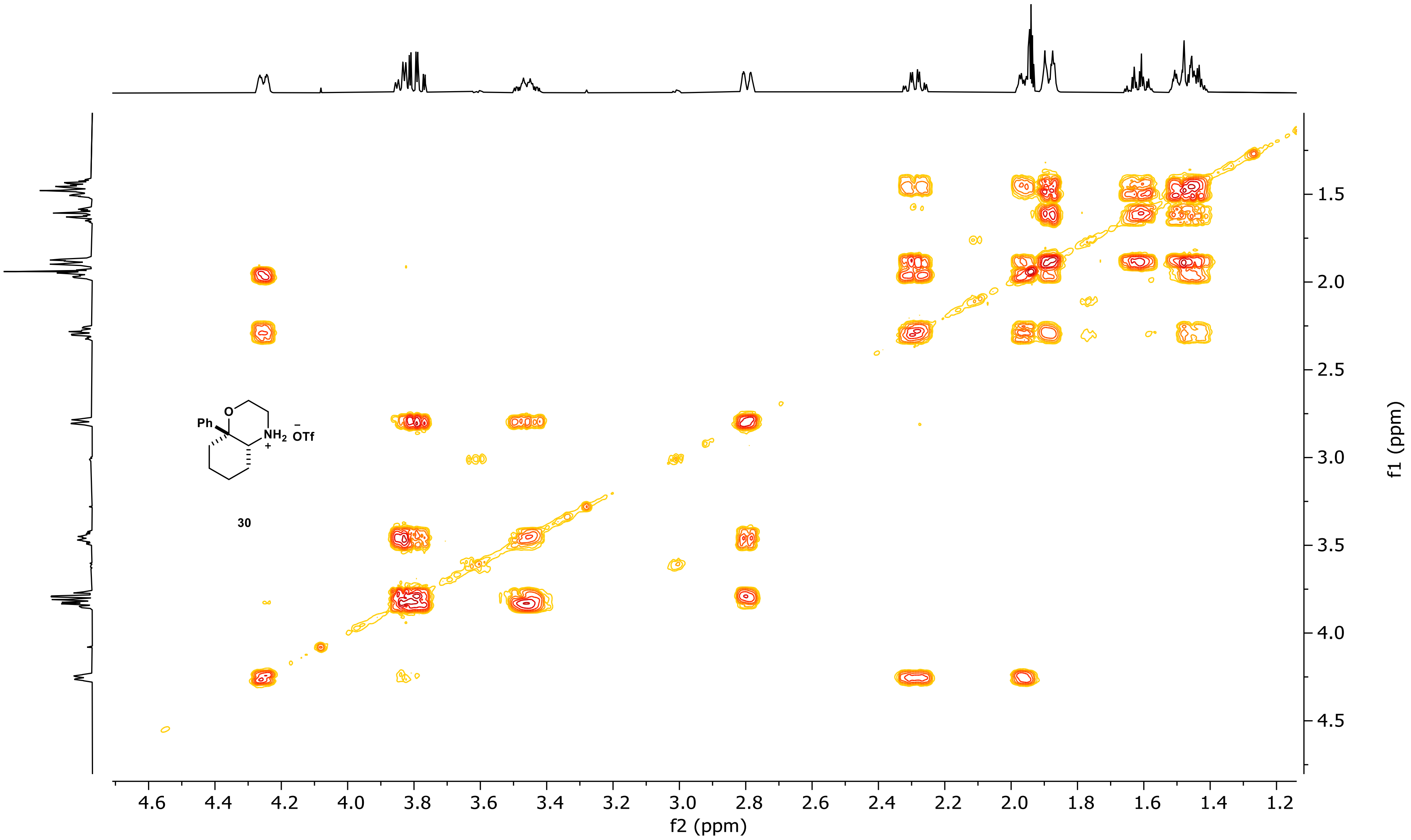

HSQC of octahydrobenzo[1,4]oxazine 30

CD_3CN, 23 ºC

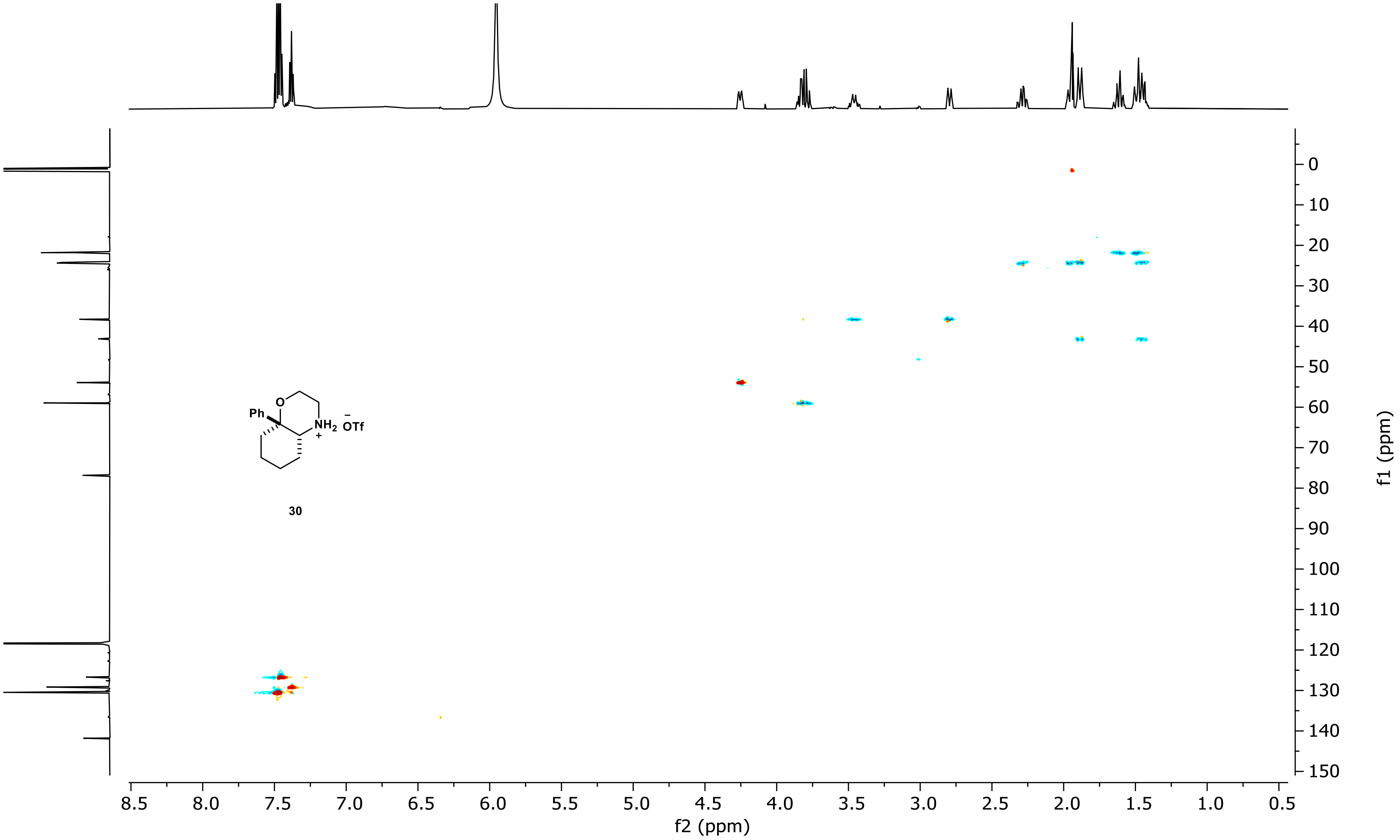

HMBC of octahydrobenzo[1,4]oxazine 30

CD_3CN, 23 ºC

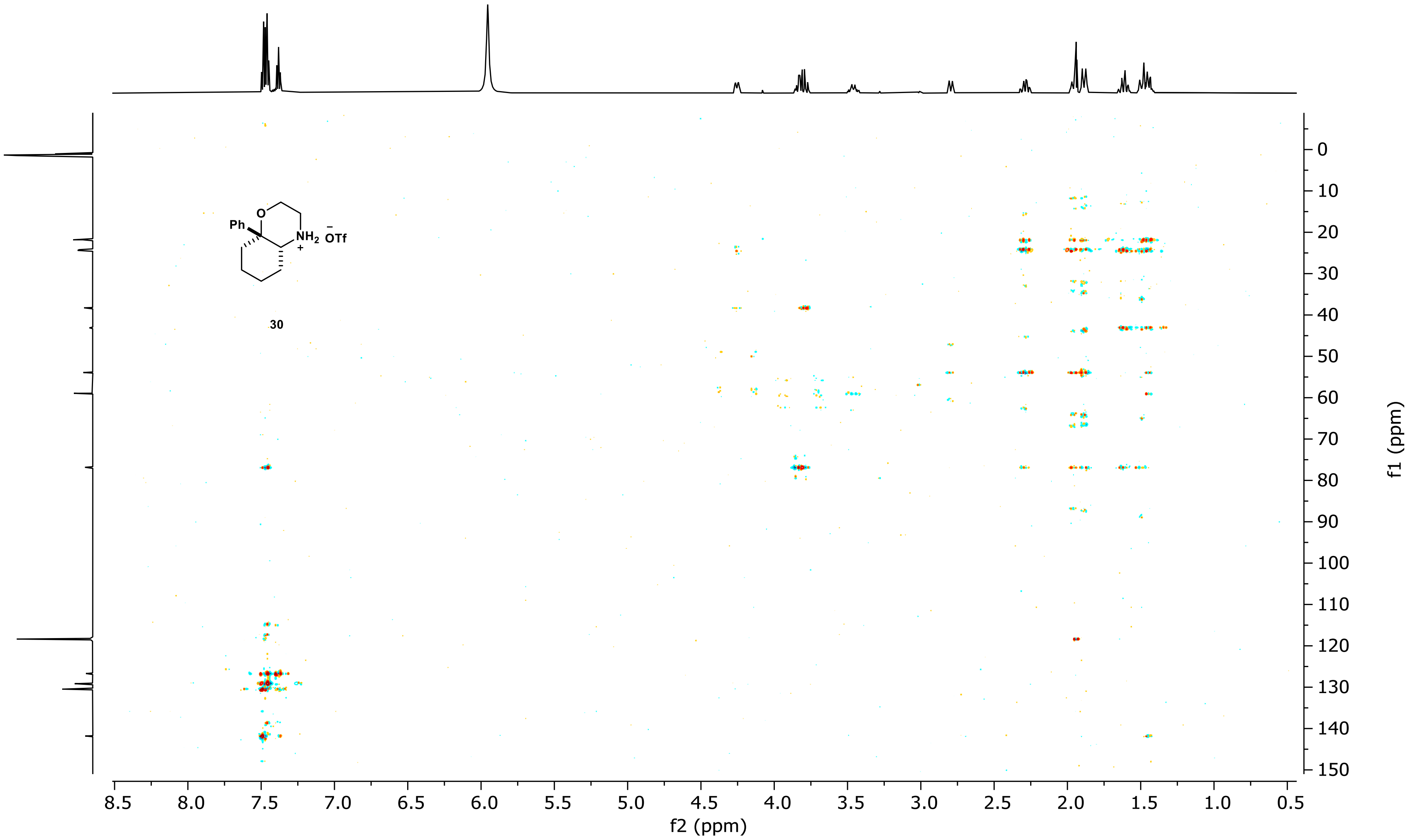

NOESY of octahydrobenzo[1,4]oxazine 30

CD_3CN, 23 ºC

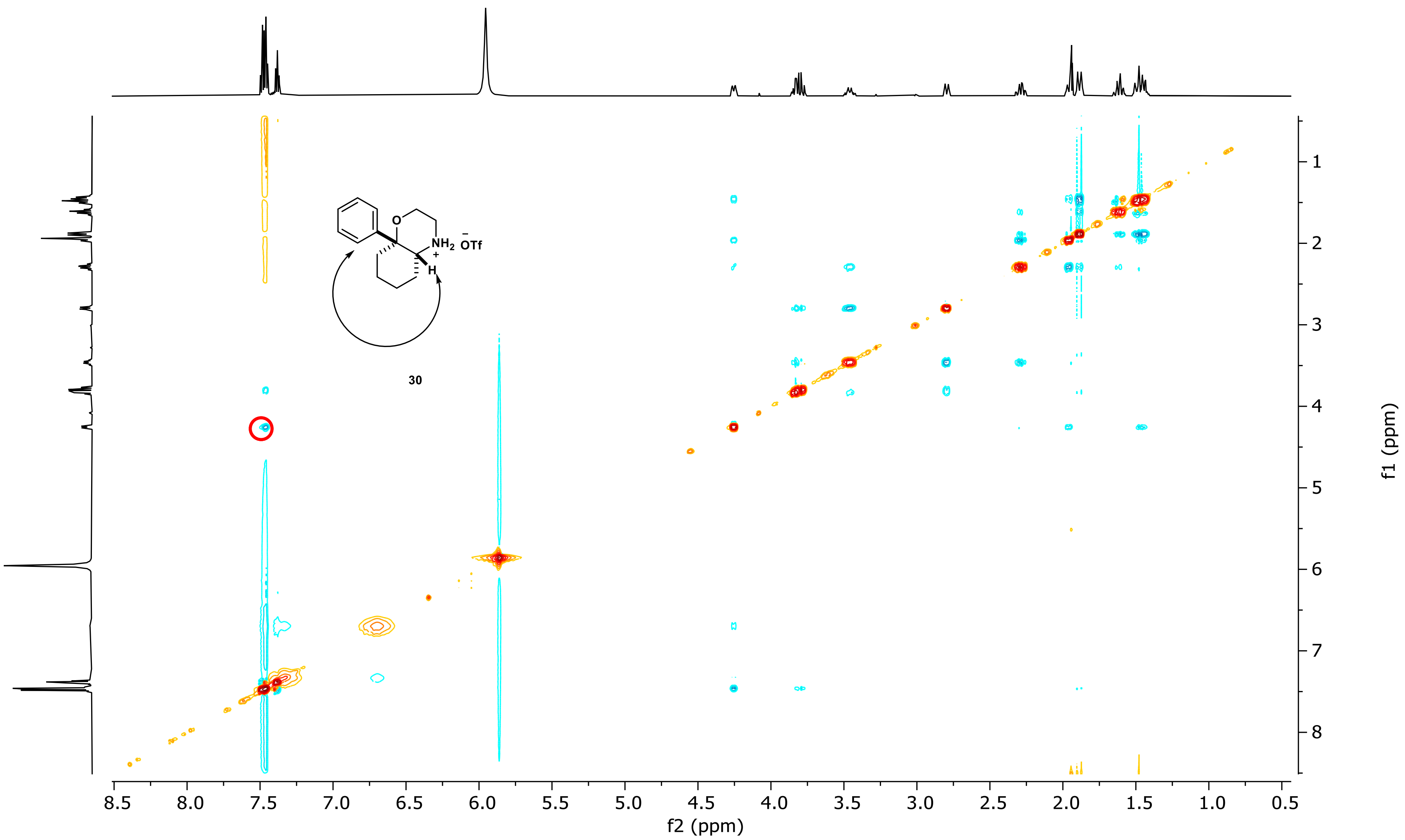

^{1}H NMR of 9b-methyl hexahydroindeno[1,4]oxazine 31

CDCl₃, 23 ºC

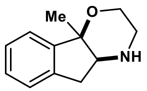

31

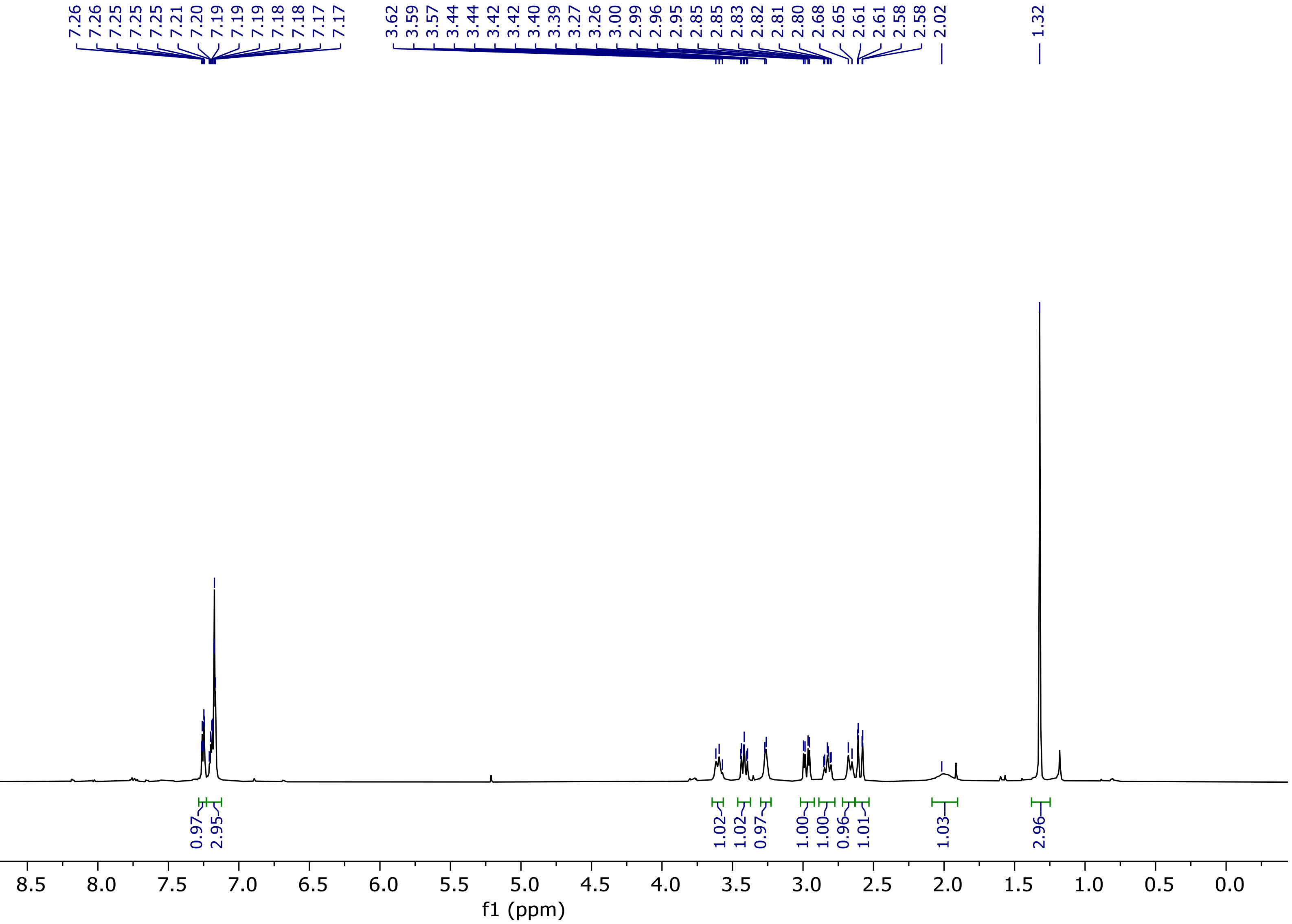

^{13}C NMR of 9b-methyl hexahydroindeno[1,4]oxazine 31

CDCl$_3$, 23 ºC

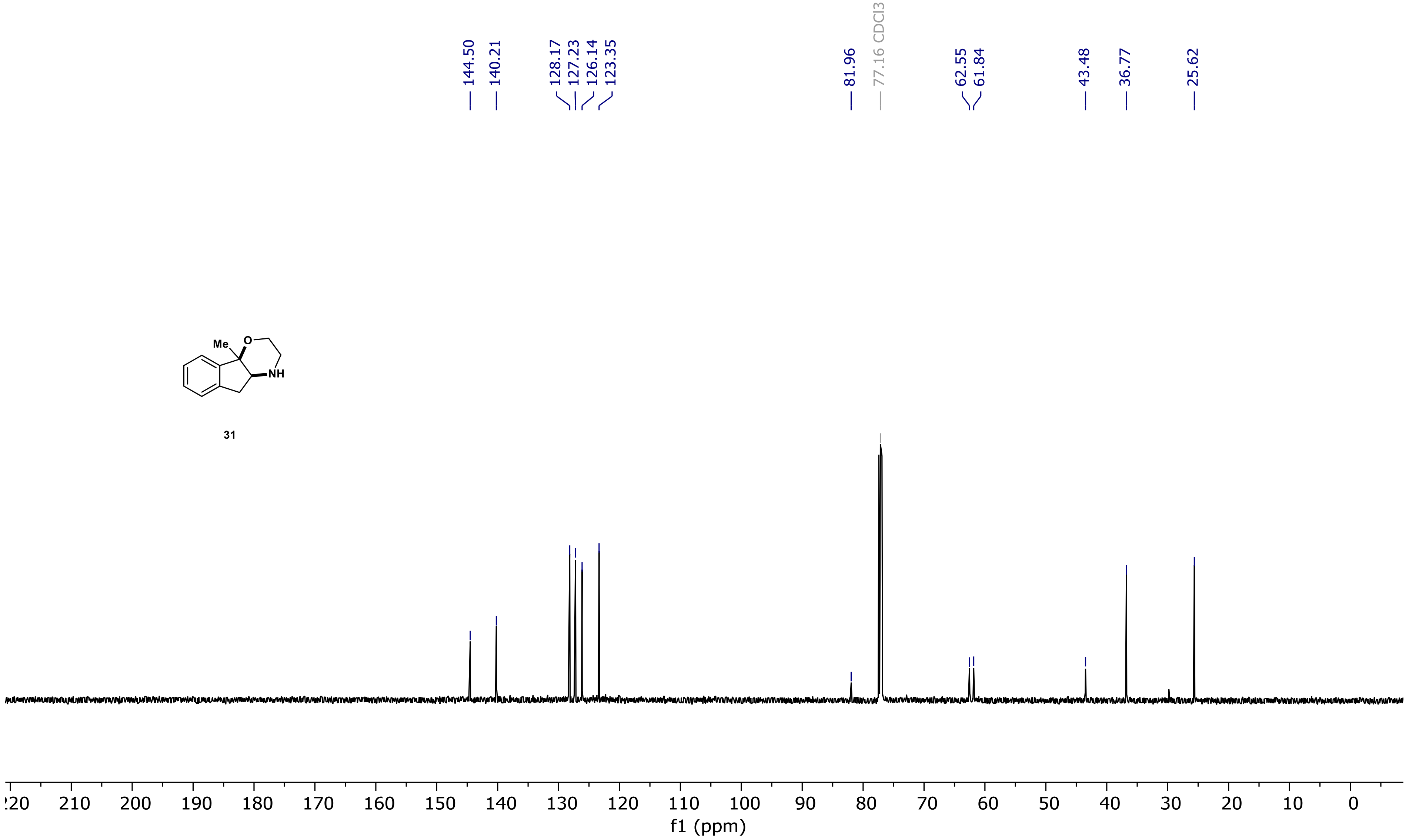

HSQC of 9b-methyl hexahydroindeno[1,4]oxazine 31

$CDCl_3$, 23 ºC

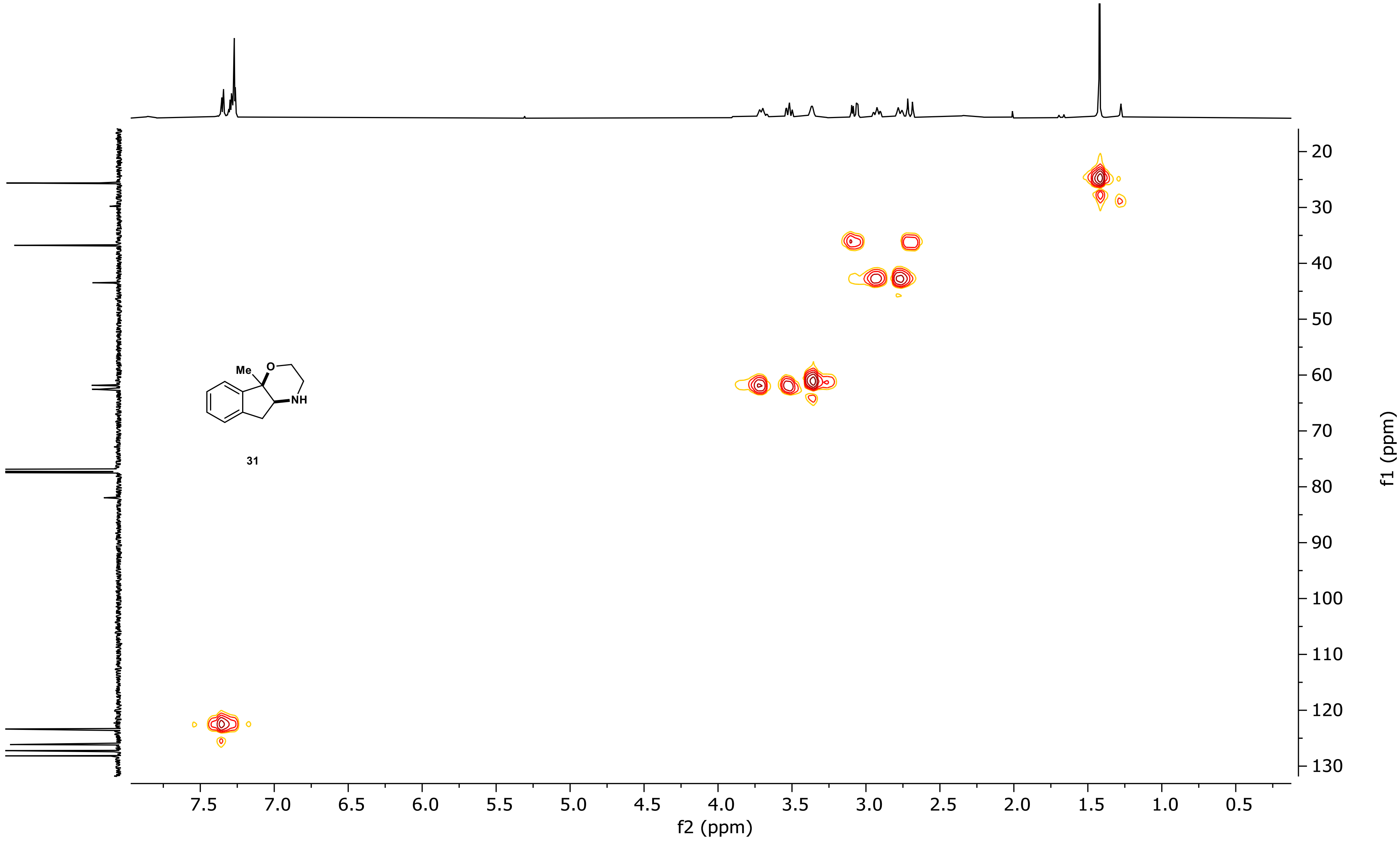

NOESY of 9b-methyl hexahydroindeno[1,4]oxazine 31

CDCl3, 23 ºC

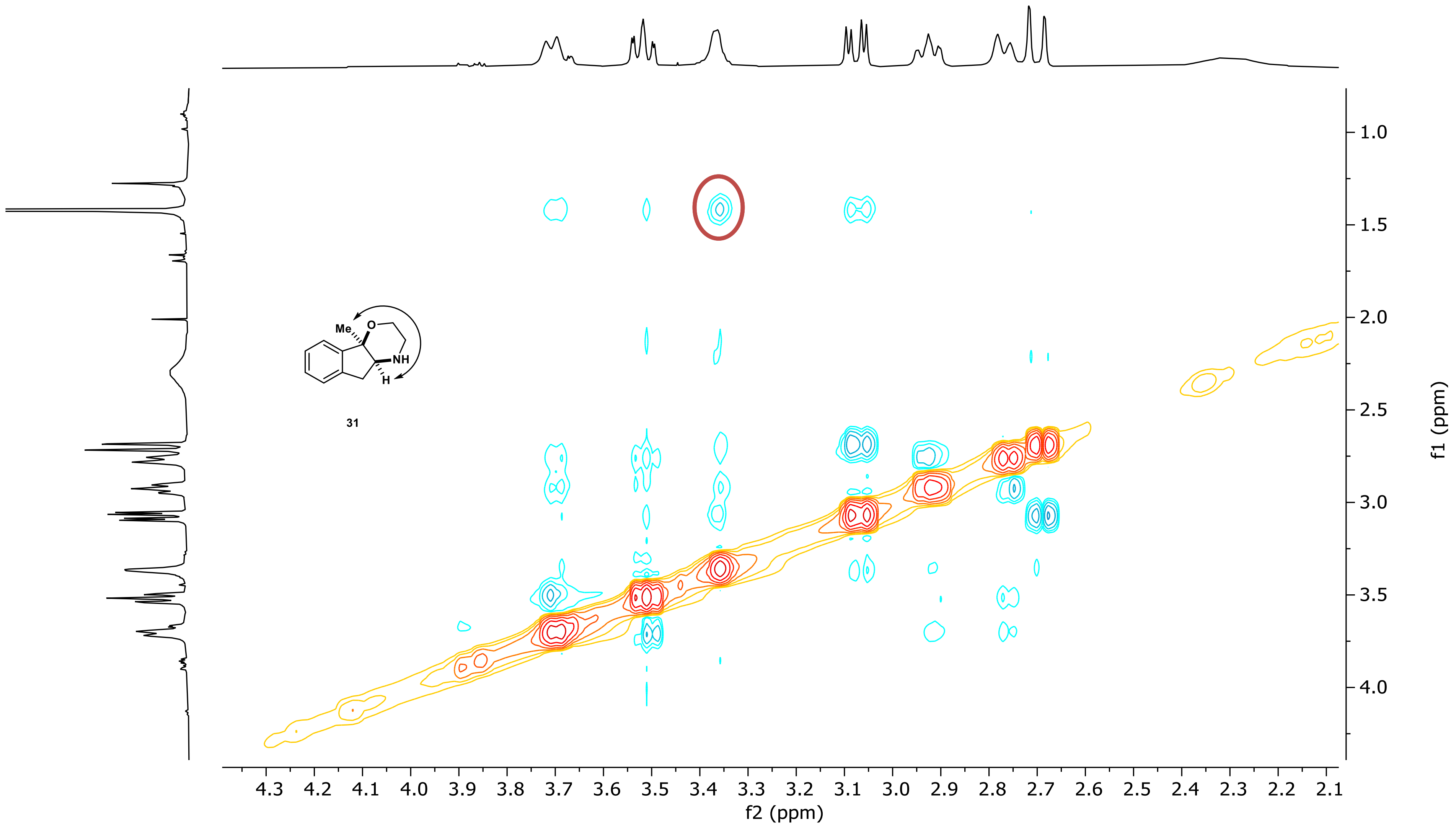

^{1}H NMR of hexahydroindeno[1,4]oxazine 32

CDCl$_3$, 23 ºC

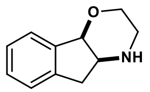

32

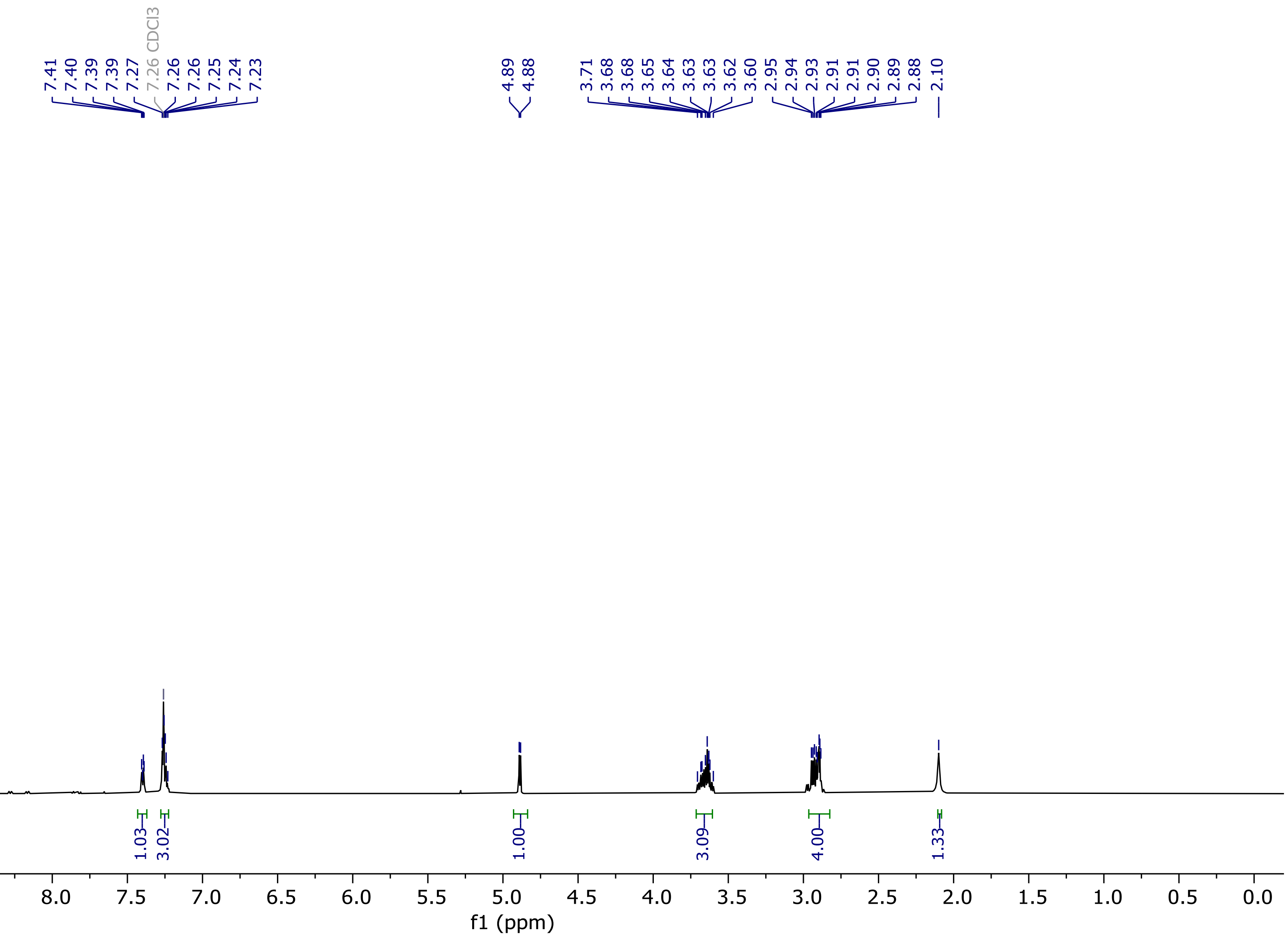

^{13}C NMR of hexahydroindeno[1,4]oxazine 32

CDCl3, 23 ºC

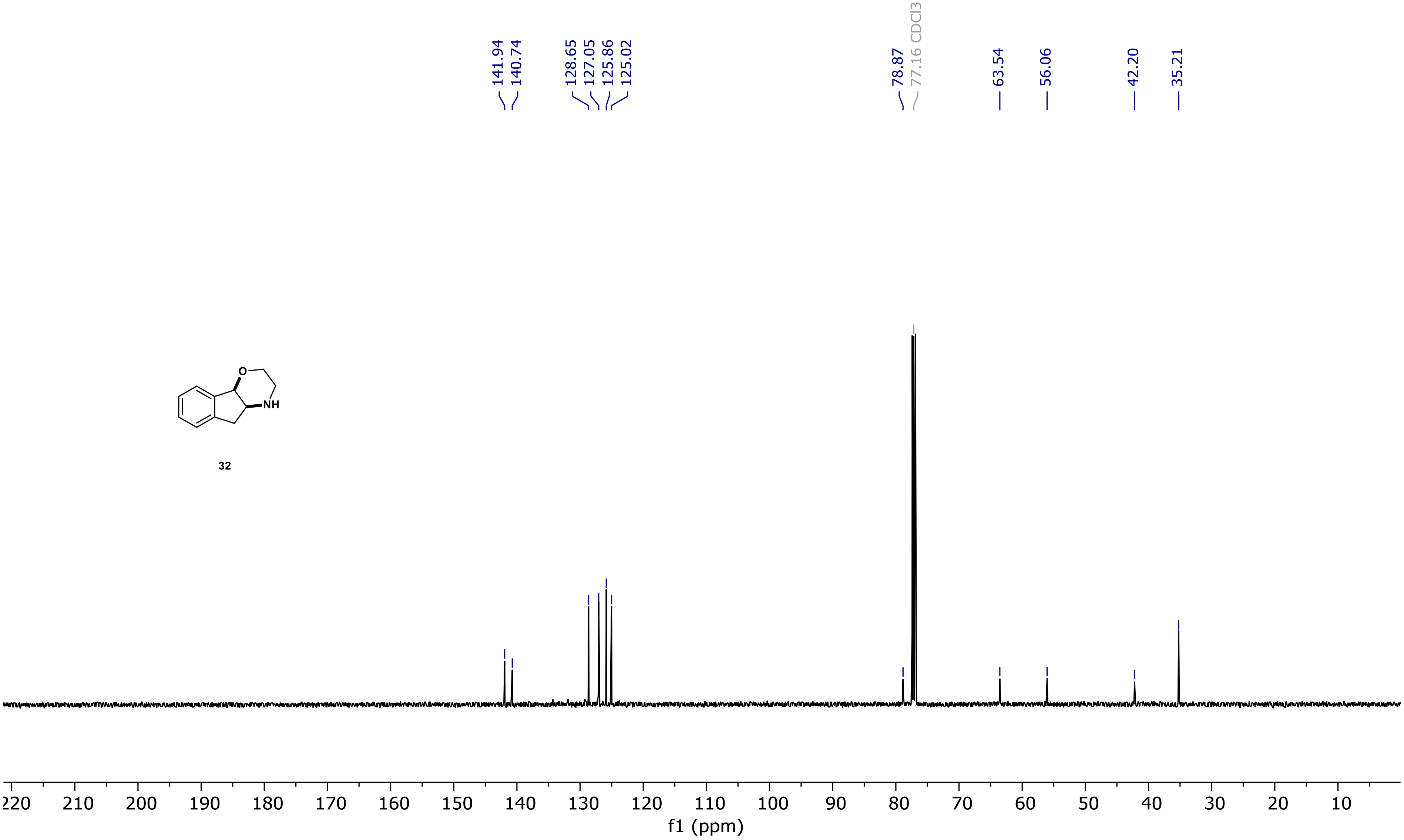

32

^{1}H NMR of spiro[chromane-4,2'-morpholine] 33

$CDCl_3$, 23 ºC

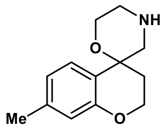

33

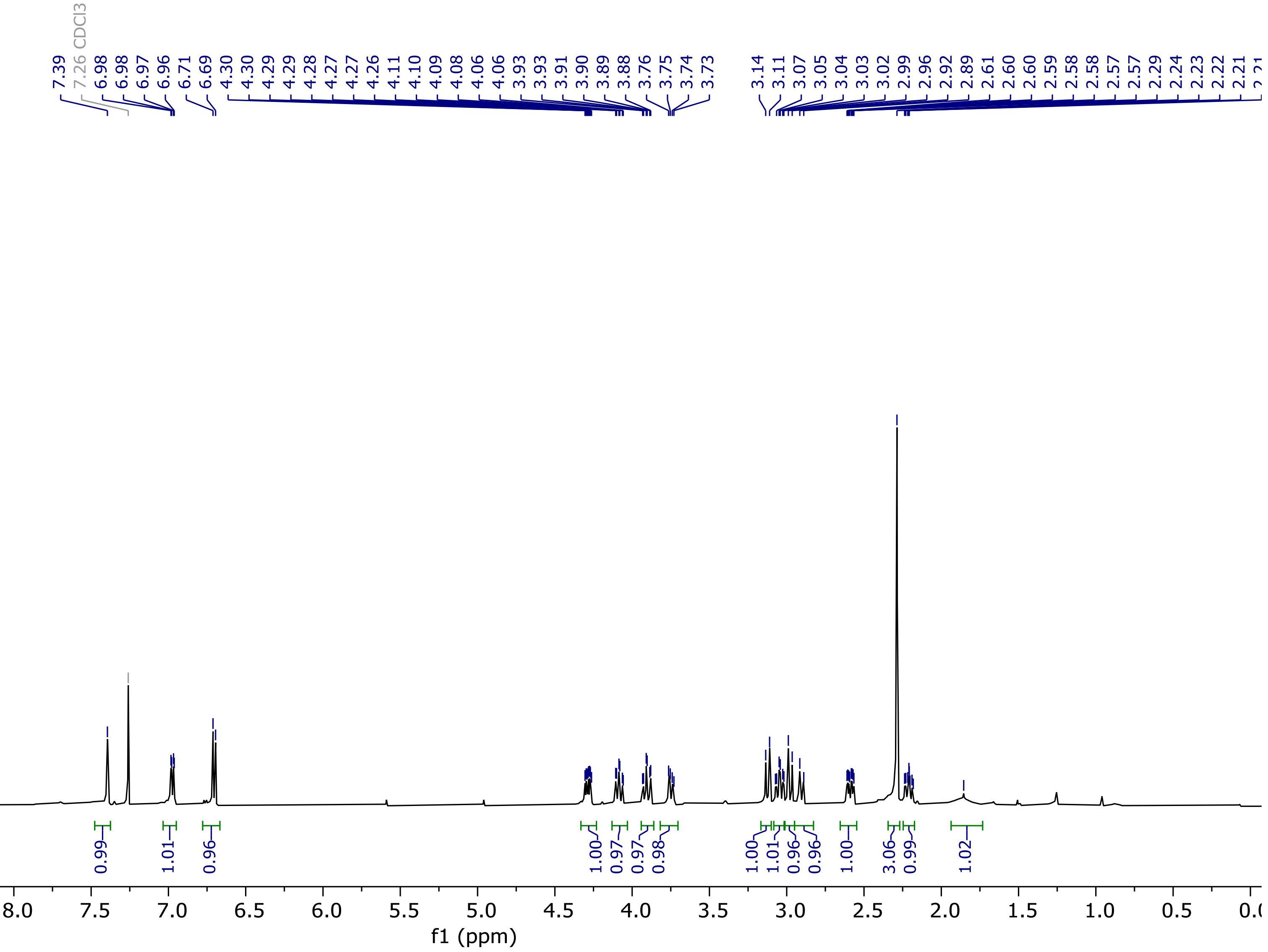

^{13}C NMR of spiro[chromane-4,2'-morpholine] 33

CDCl$_3$, 23 ºC

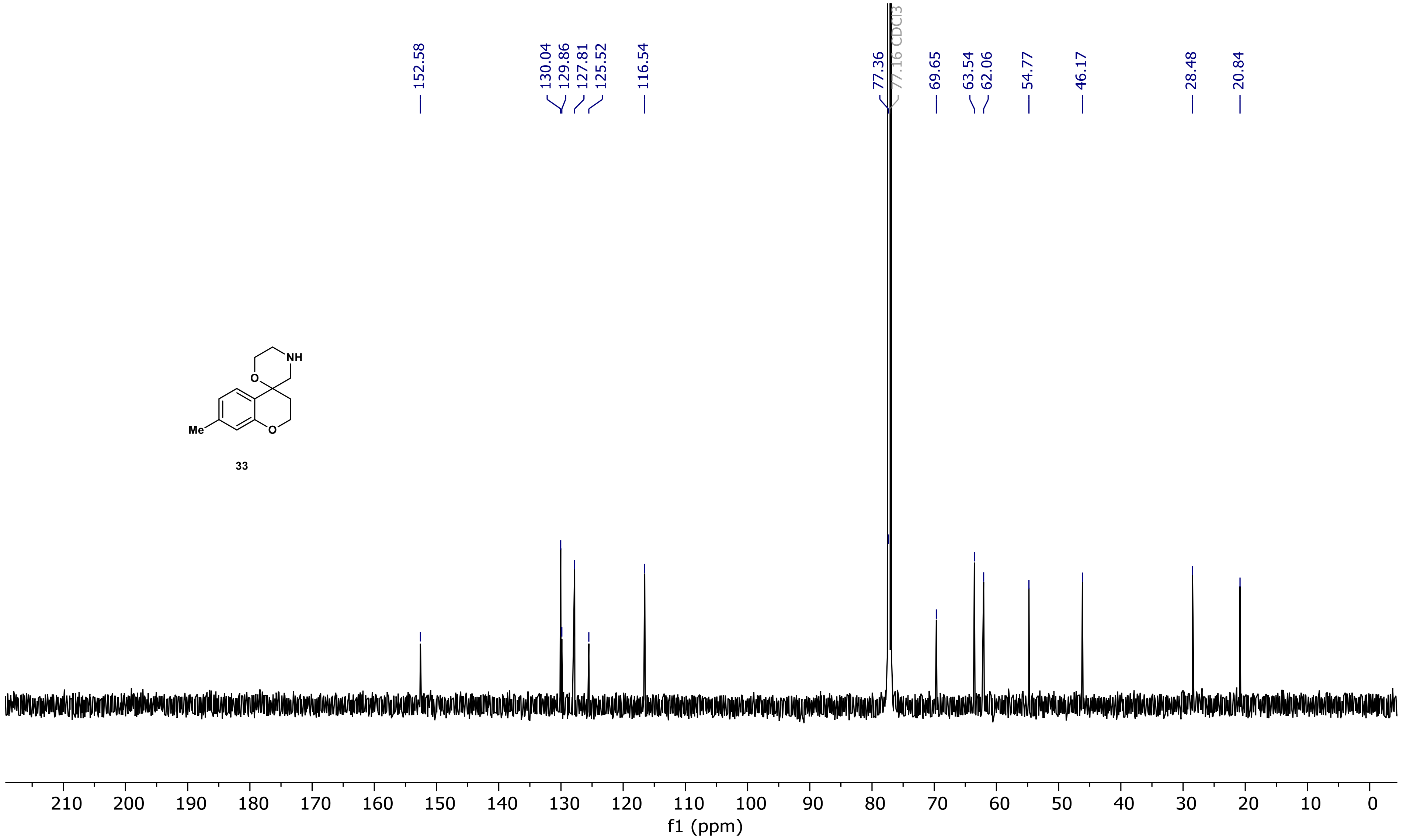

33

^{1}H NMR of 2-styrylmorpholine 34

CDCl$_3$, 23 ºC

a mixture of stereoisomers with 1:1 E:Z

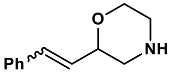

34

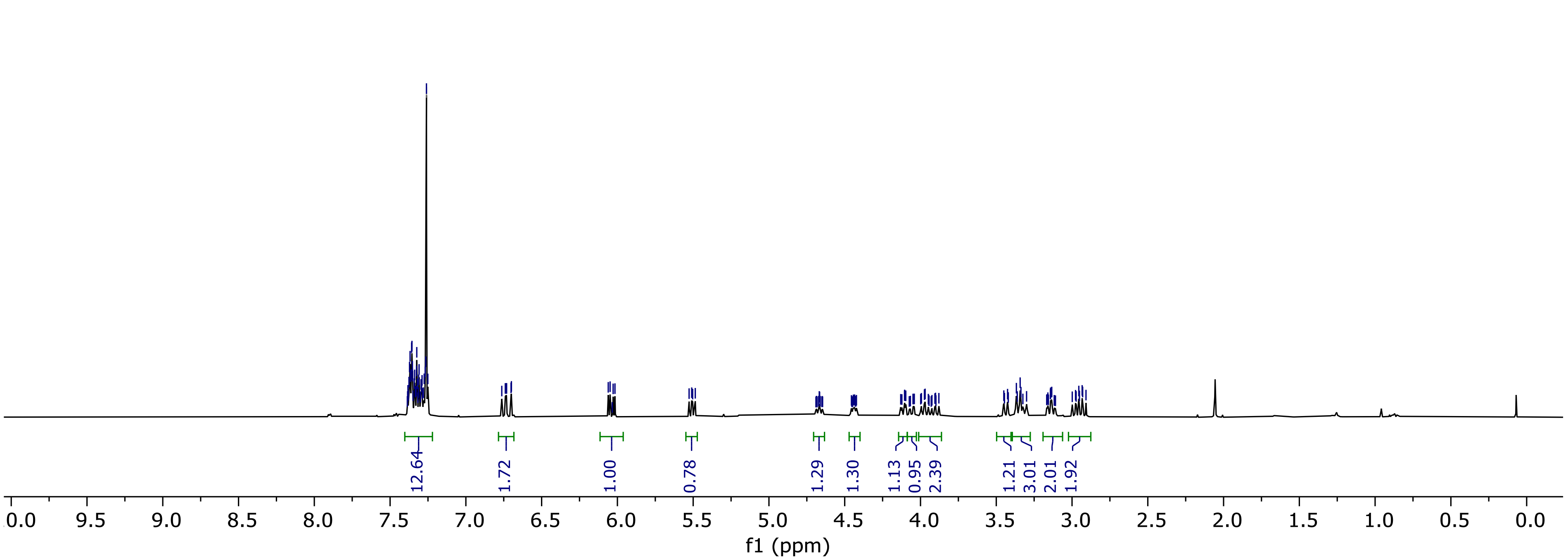

^{13}C NMR of 2-styrylmorpholine 34

$CDCl_3$, 23 ºC

a mixture of stereoisomers with 1:1 E:Z

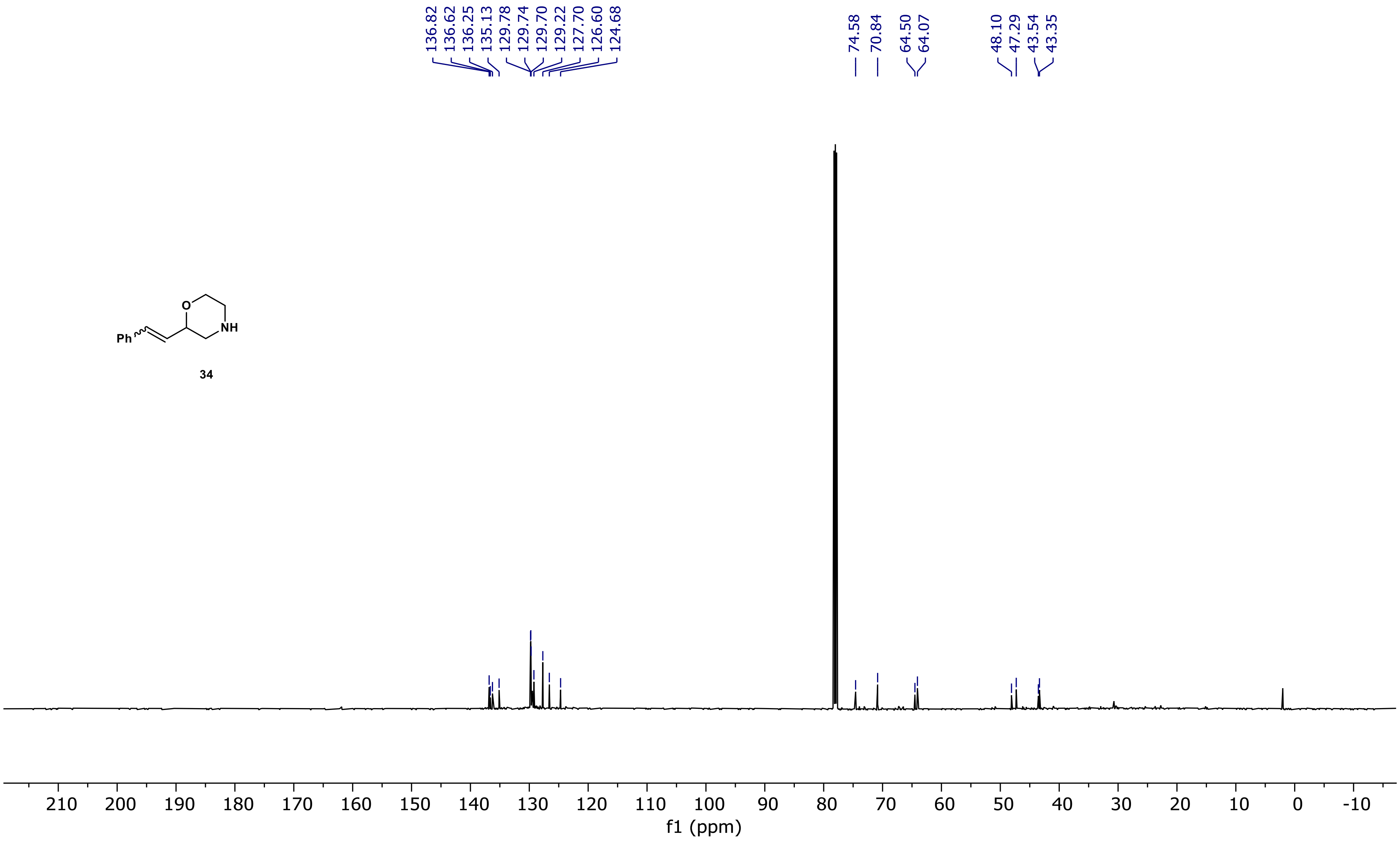

^{1}H NMR of (E)-2-(Propen-1-yl)morpholine 35

$CDCl_3$, 23 ºC

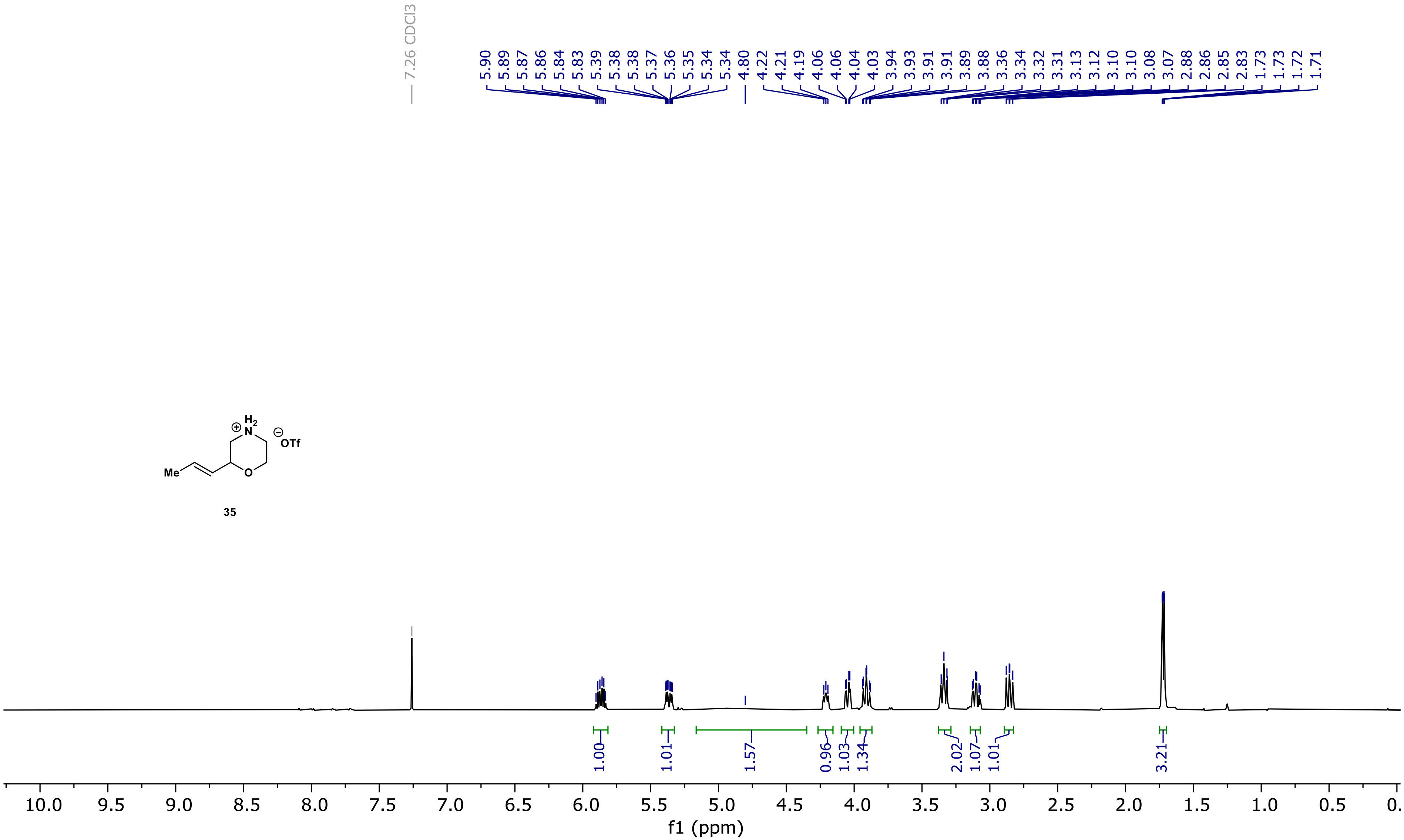

^{13}C NMR of (E)-2-(Propen-1-yl)morpholine 35

CDCl$_3$, 23 ºC

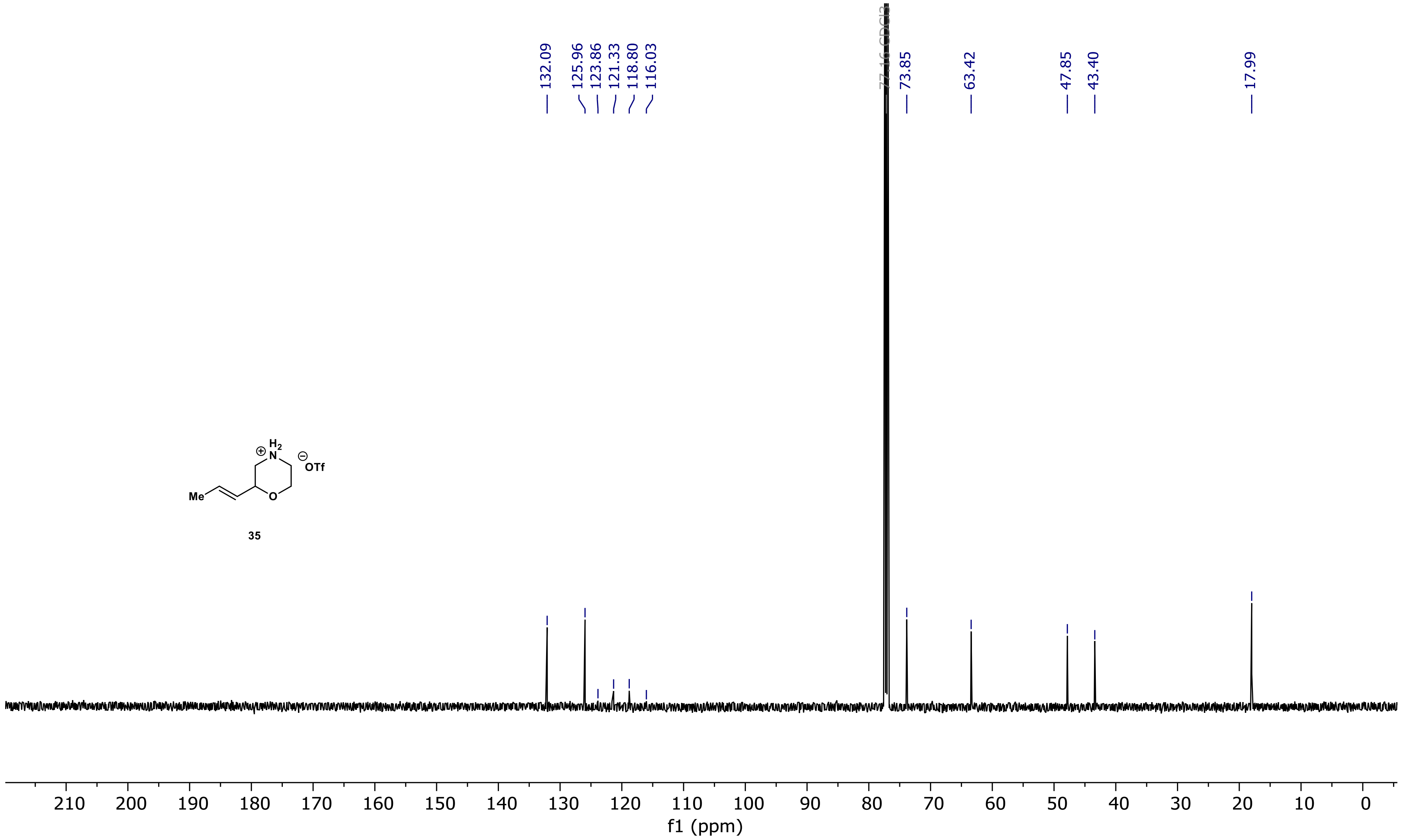

^{19}F NMR of (E)-2-(Propen-1-yl)morpholine 35

$CDCl_3$, 23 ºC

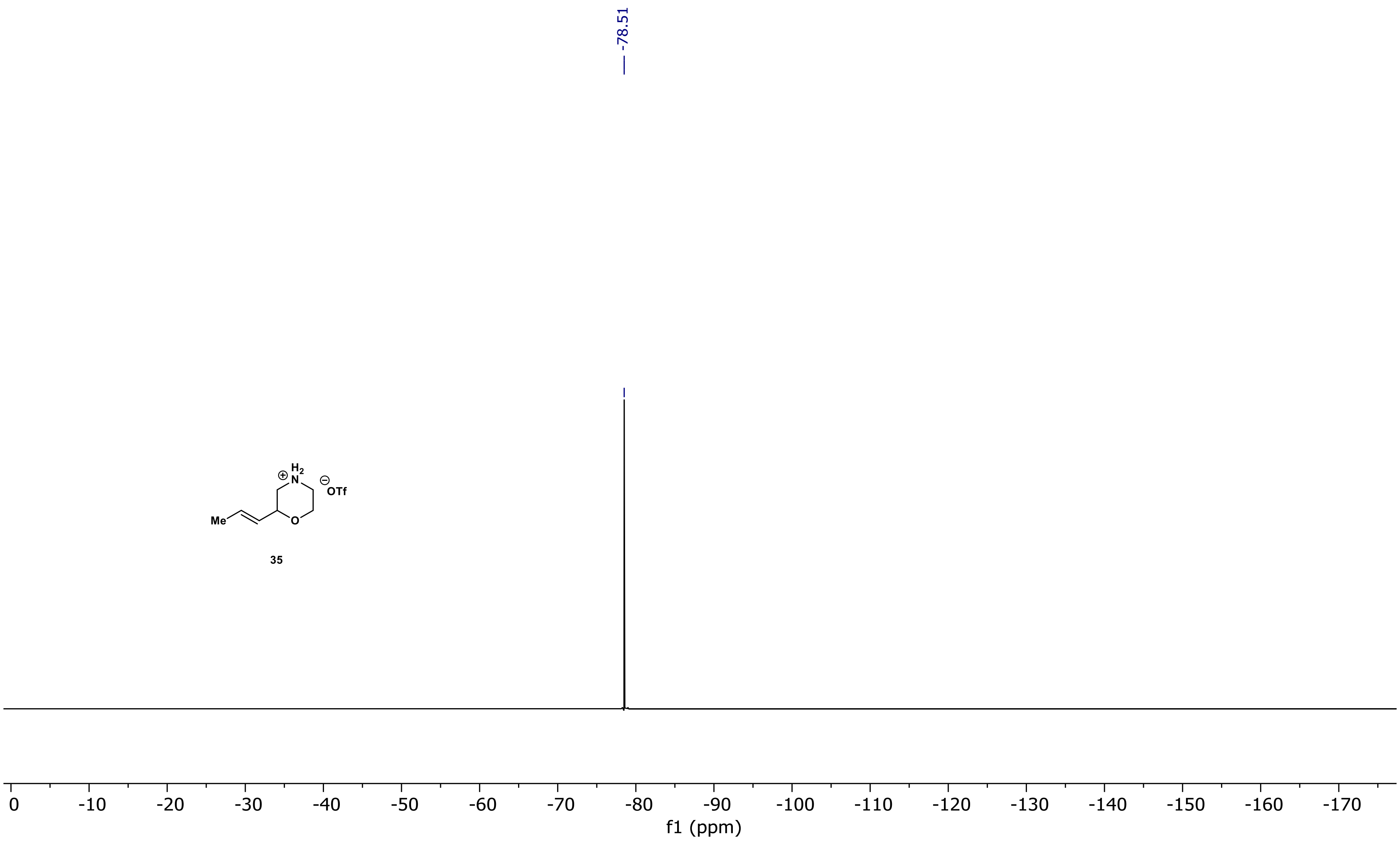

^{1}H NMR of 2-(4-benzamide)phenylmorpholine 36

d6-DMSO, 23 ºC

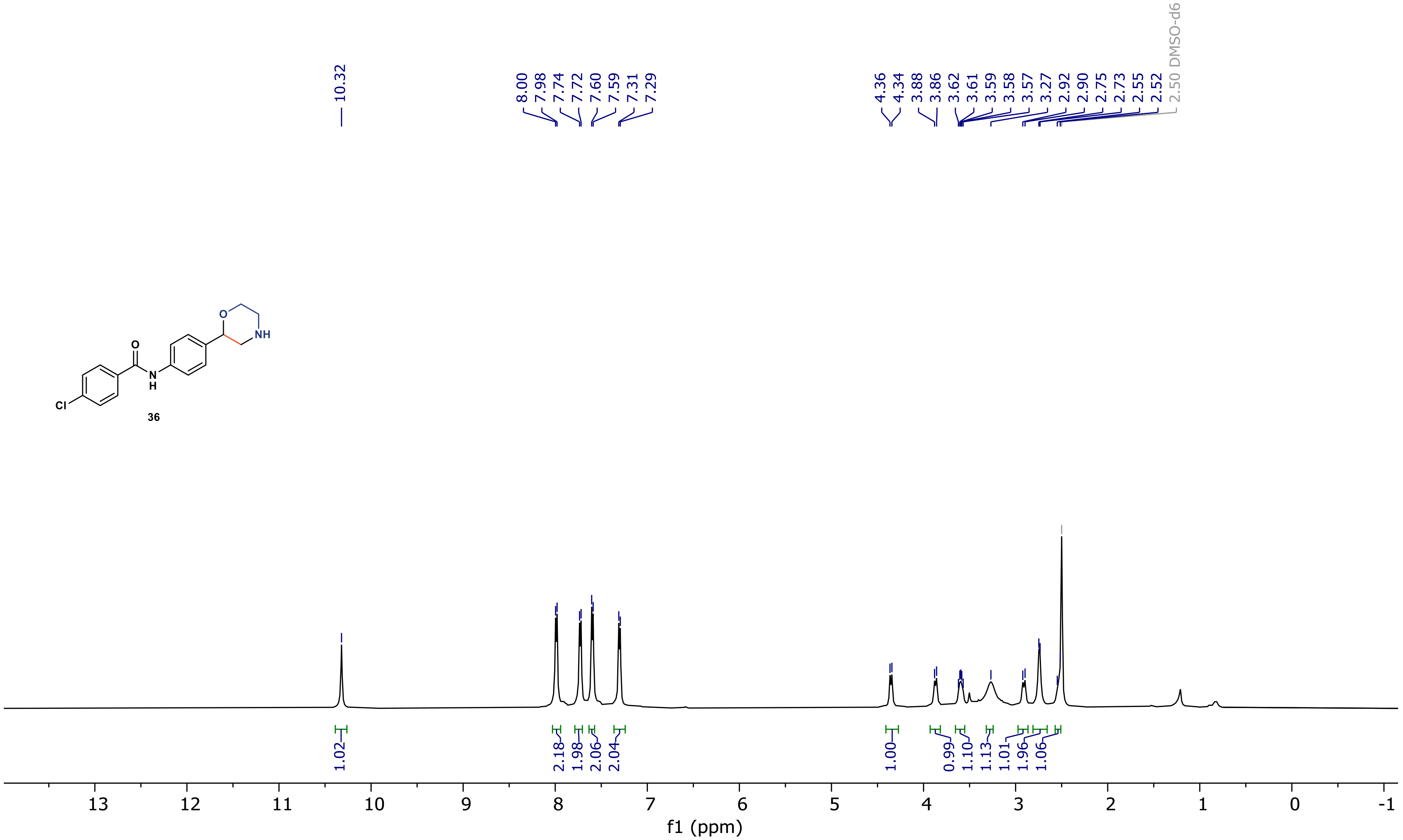

^{13}C NMR of 2-(4-benzamide)phenylmorpholine 36

d_6-DMSO, 23 ºC

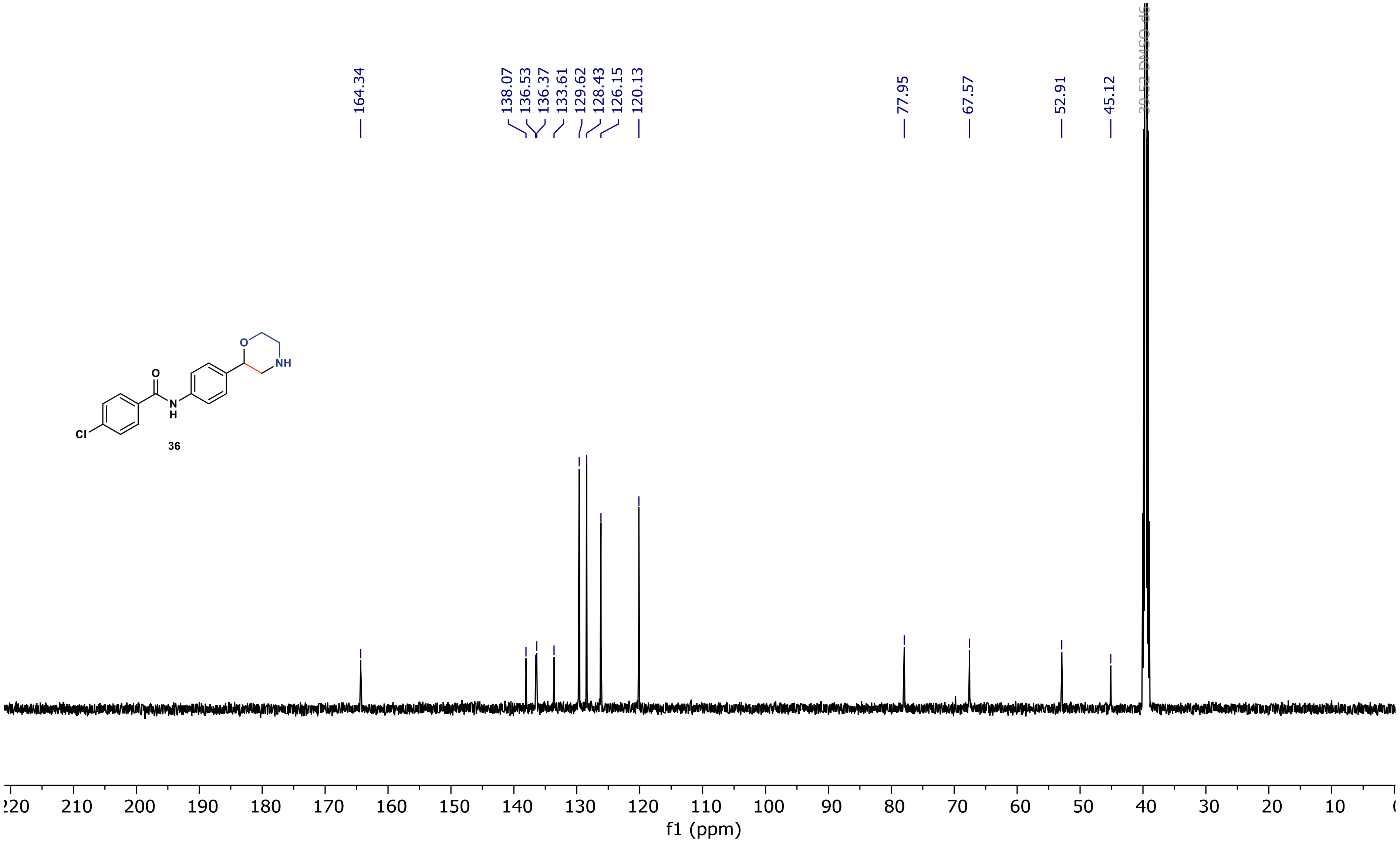

36

^{1}H NMR of spirobicyclo[2.2.1]heptane-2,2'-morpholine 37

$CDCl_3$, 23 ºC

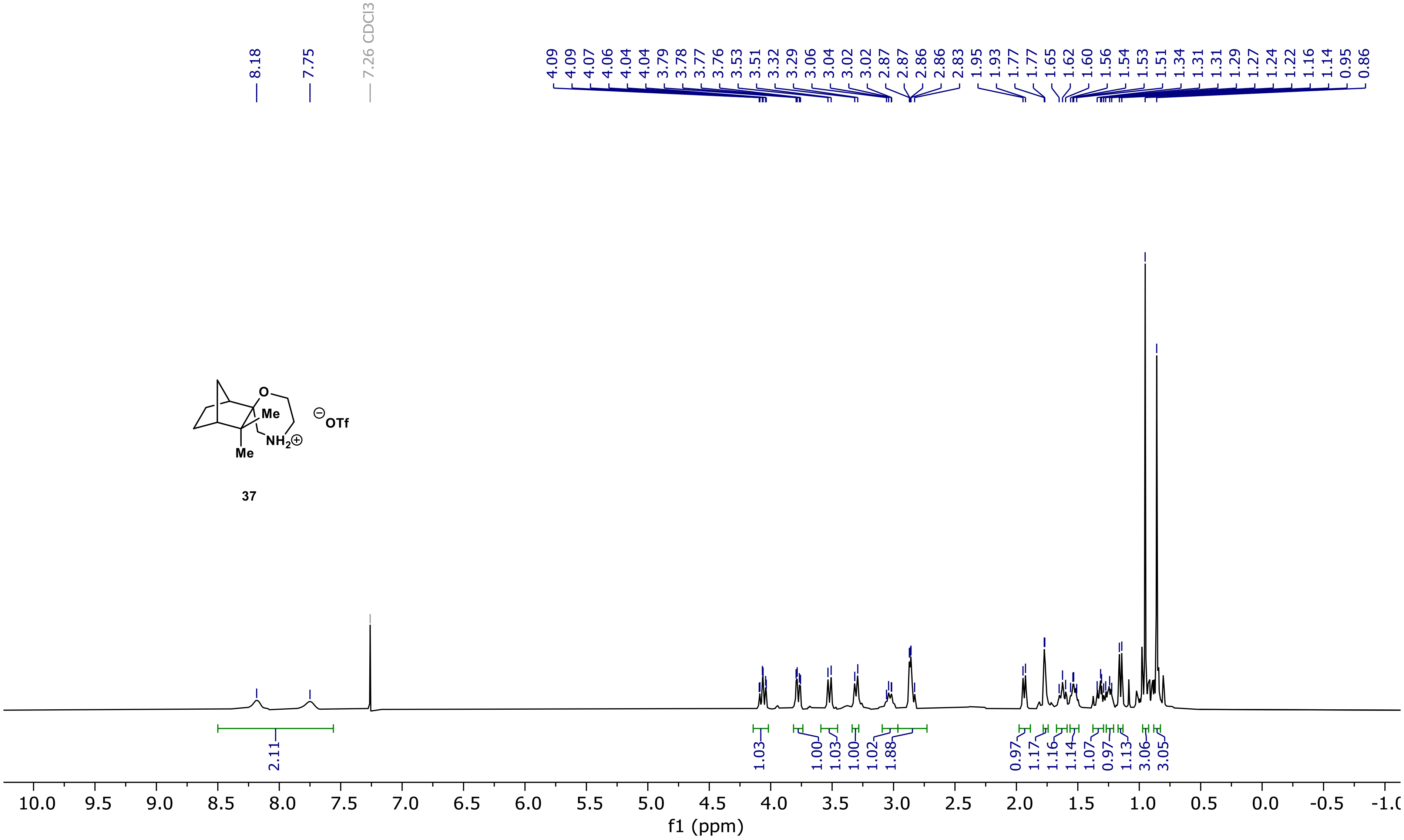

^{13}C NMR of spirobicyclo[2.2.1]heptane-2,2'-morpholine 37

CDCl$_3$, 23 ºC

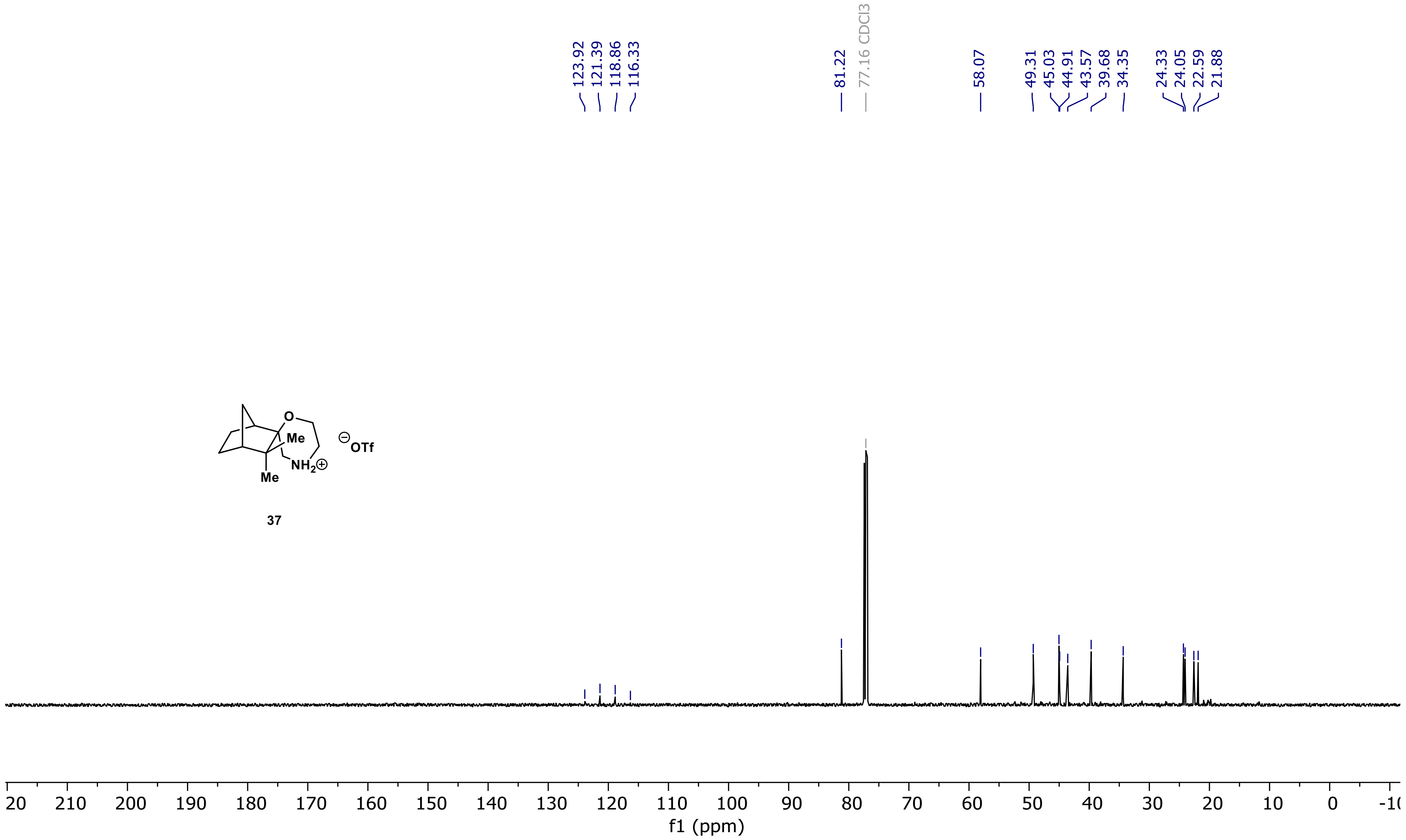

^{19}F NMR of spirobicyclo[2.2.1]heptane-2,2'-morpholine 37

$CDCl_3$, 23 ºC

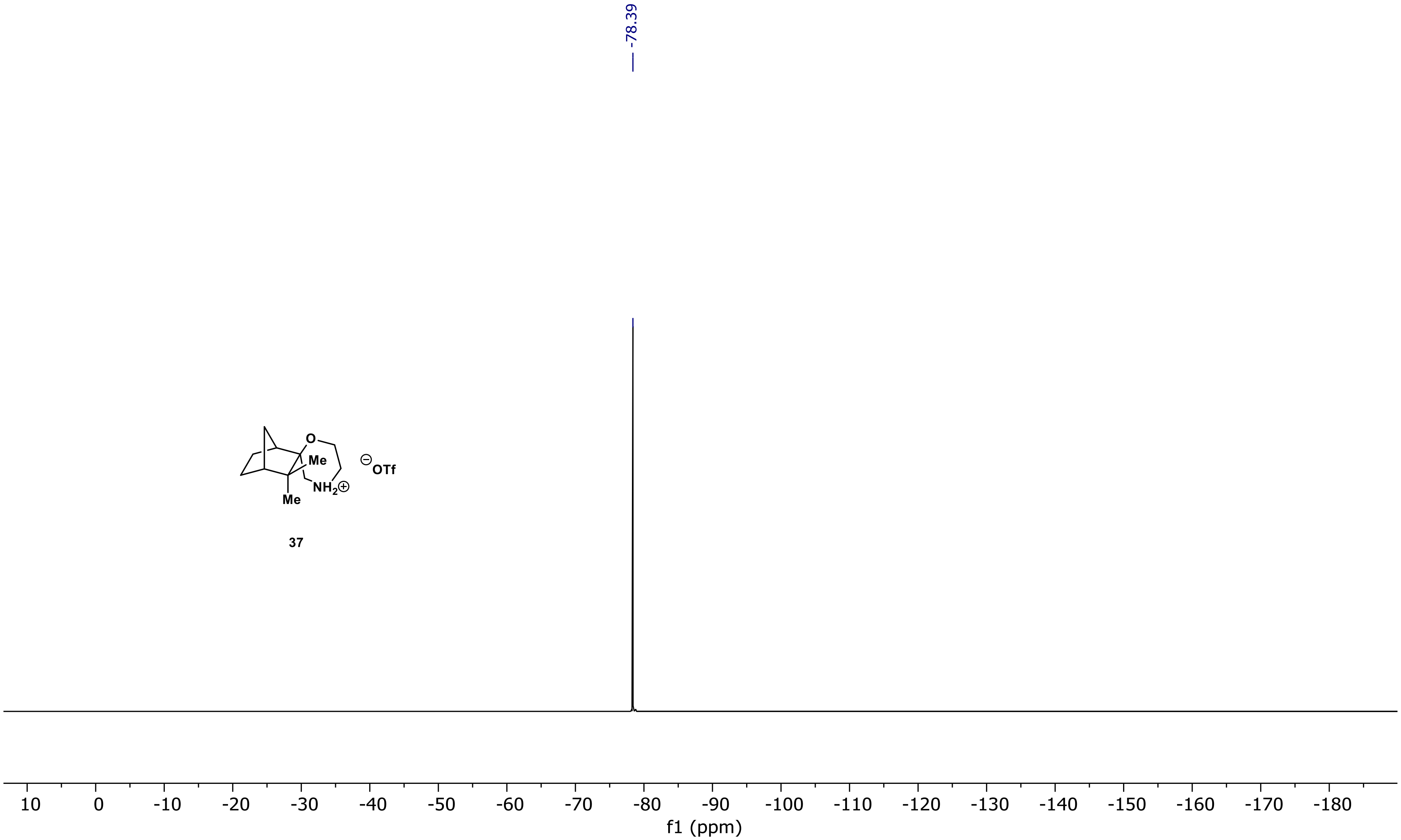

COSY of spirobicyclo[2.2.1]heptane-2,2'-morpholine 37

CDCl₃, 23 ºC

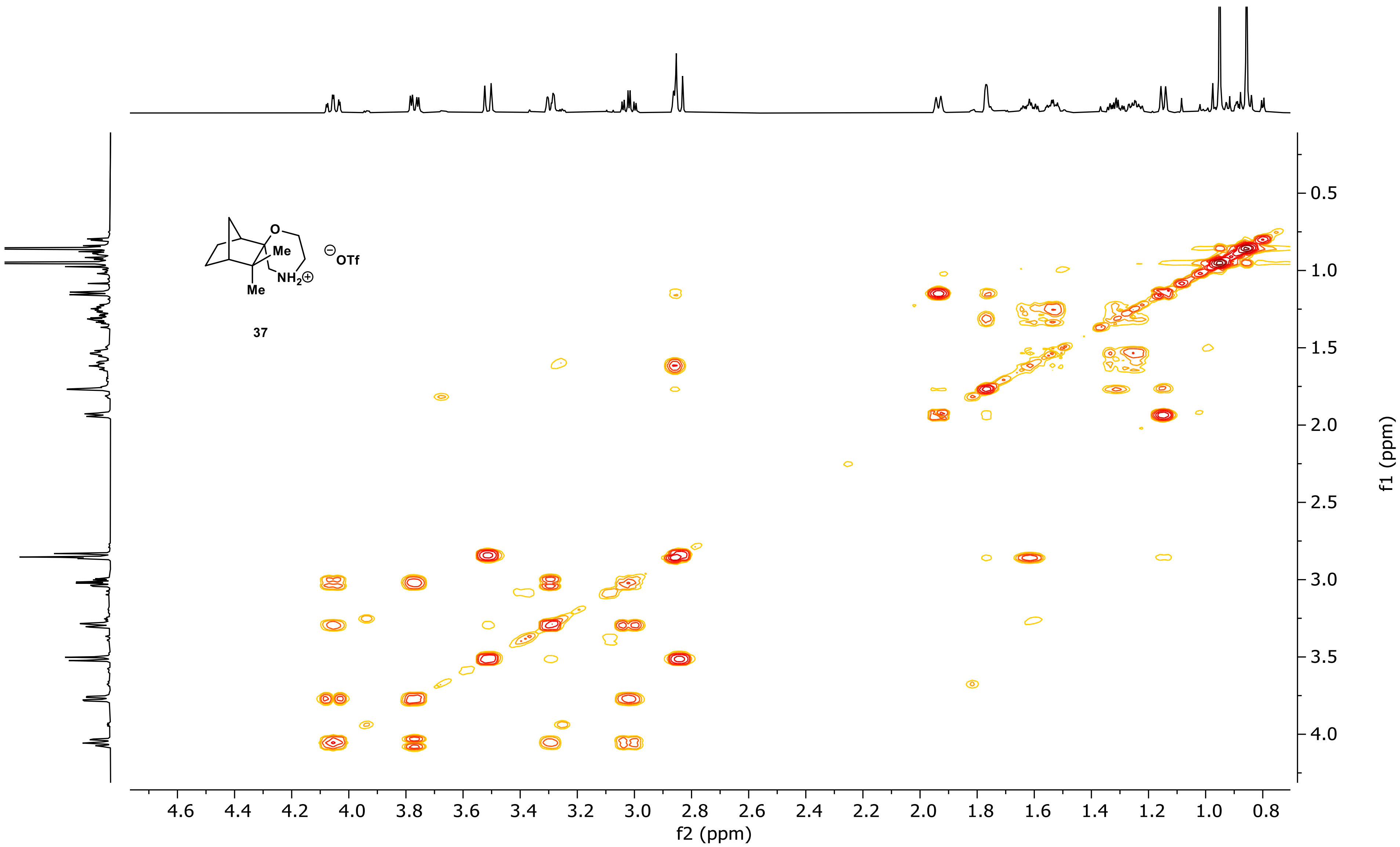

HSQC of spirobicyclo[2.2.1]heptane-2,2'-morpholine 37

$CDCl_3$, 23 ºC

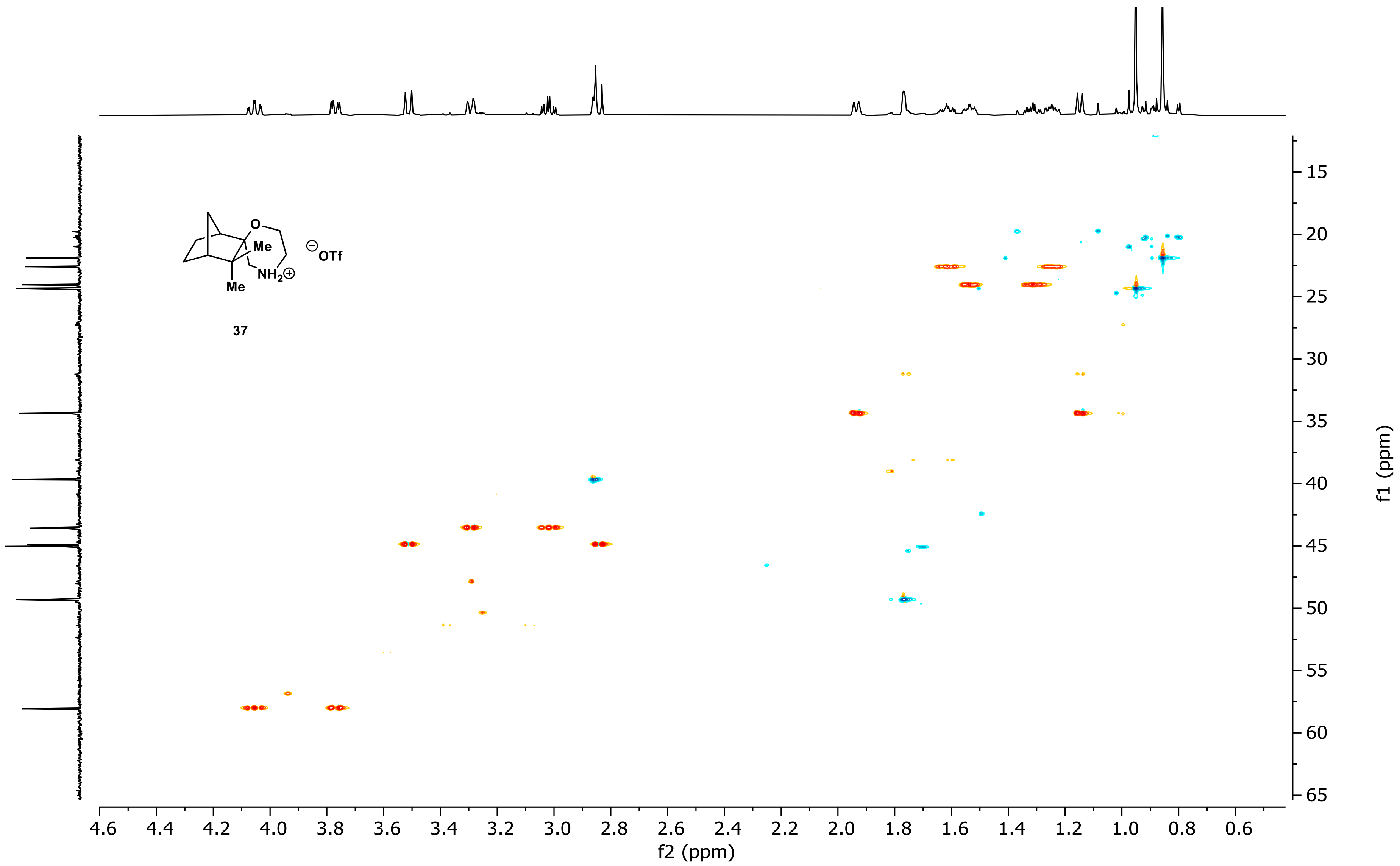

HMBC of spirobicyclo[2.2.1]heptane-2,2'-morpholine 37

$CDCl_3$, 23 ºC

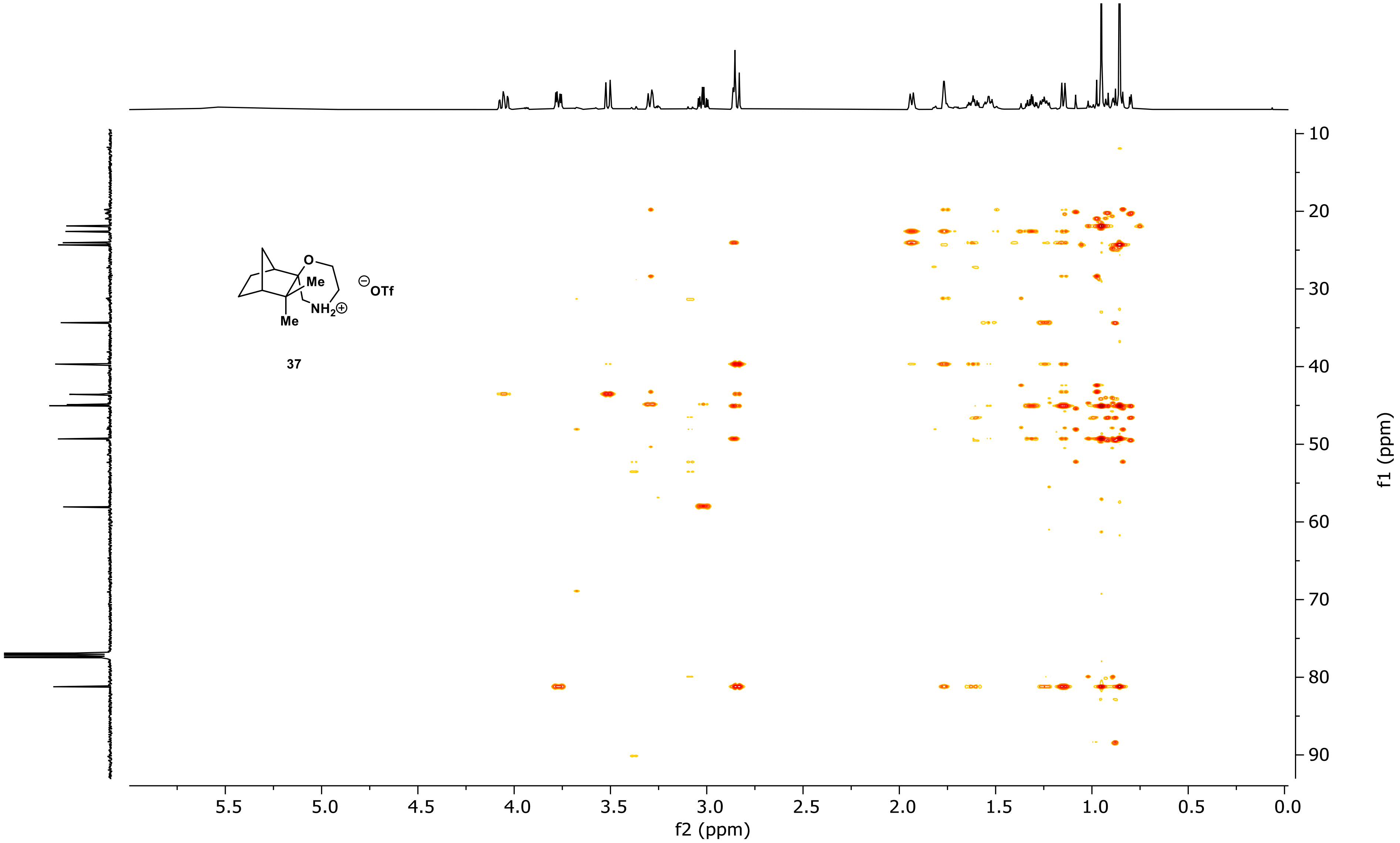

NOESY of spirobicyclo[2.2.1]heptane-2,2'-morpholine 37

CDCl₃, 23 ºC

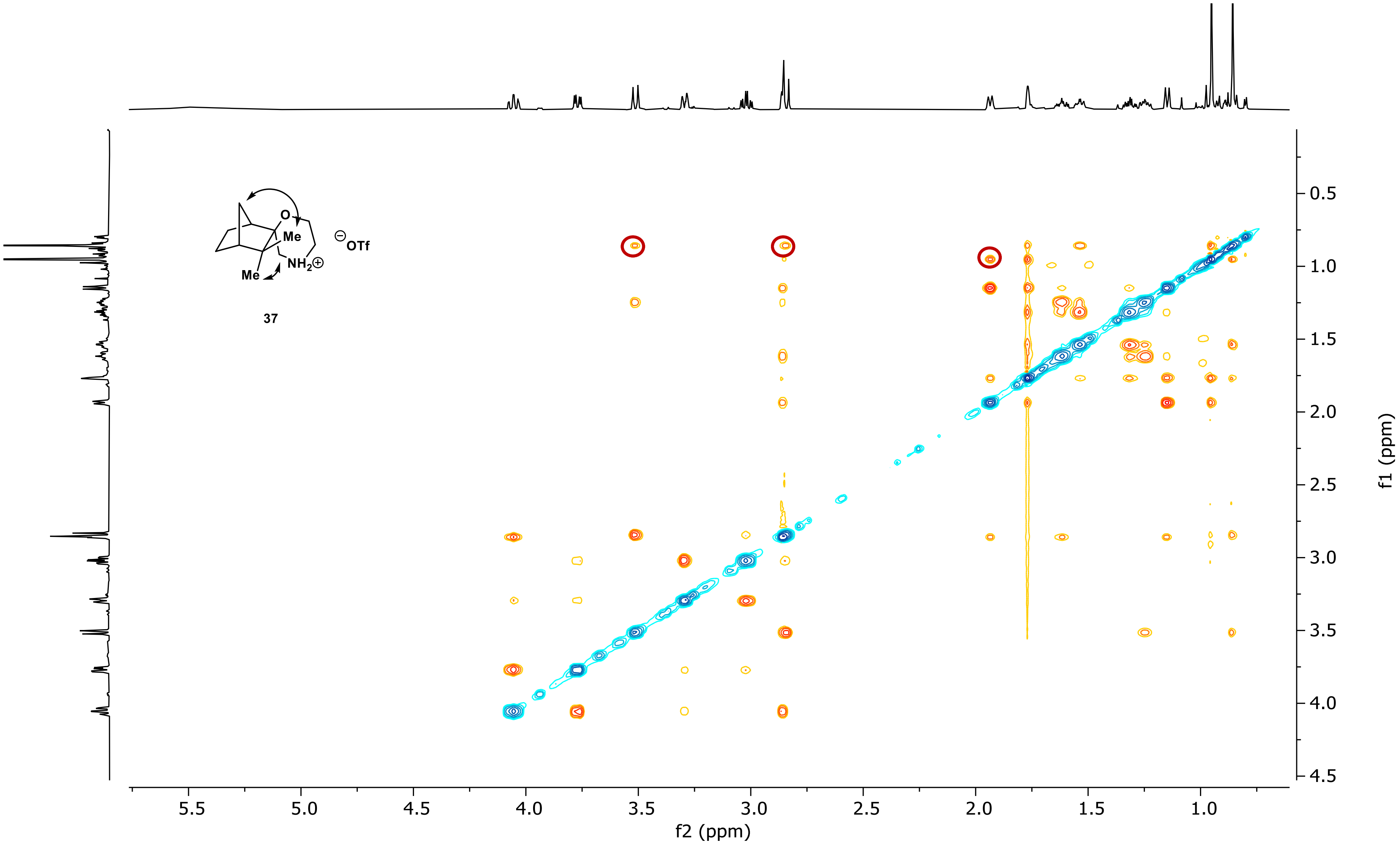

^{1}H NMR of pyriproxyphen derived alkene S1

$CDCl_3$, 23 ºC

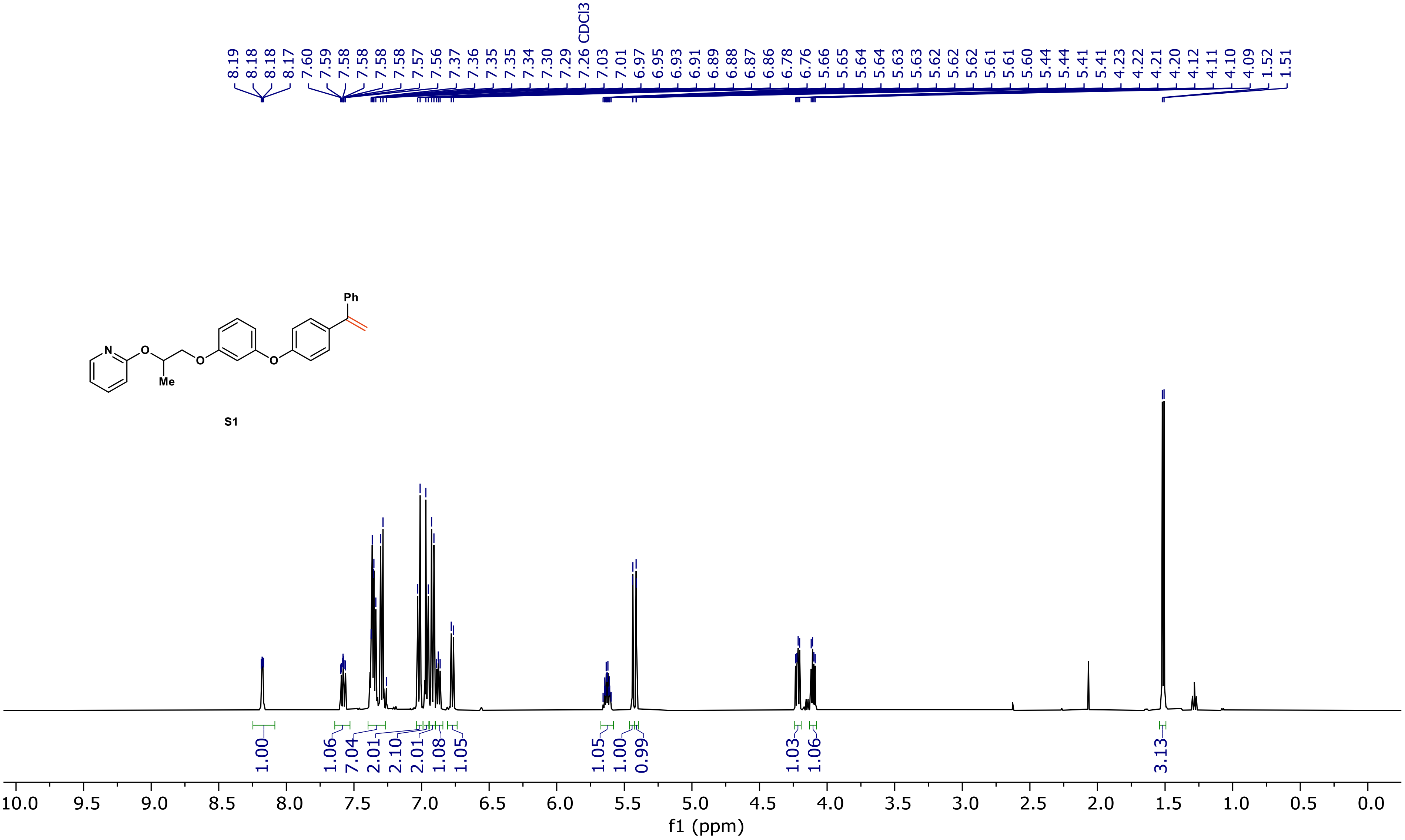

^{13}C NMR of pyriproxyphen derived alkene S1

CDCl$_3$, 23 ºC

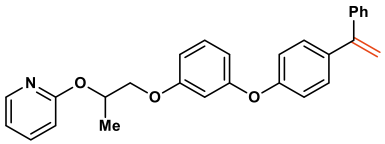

S1

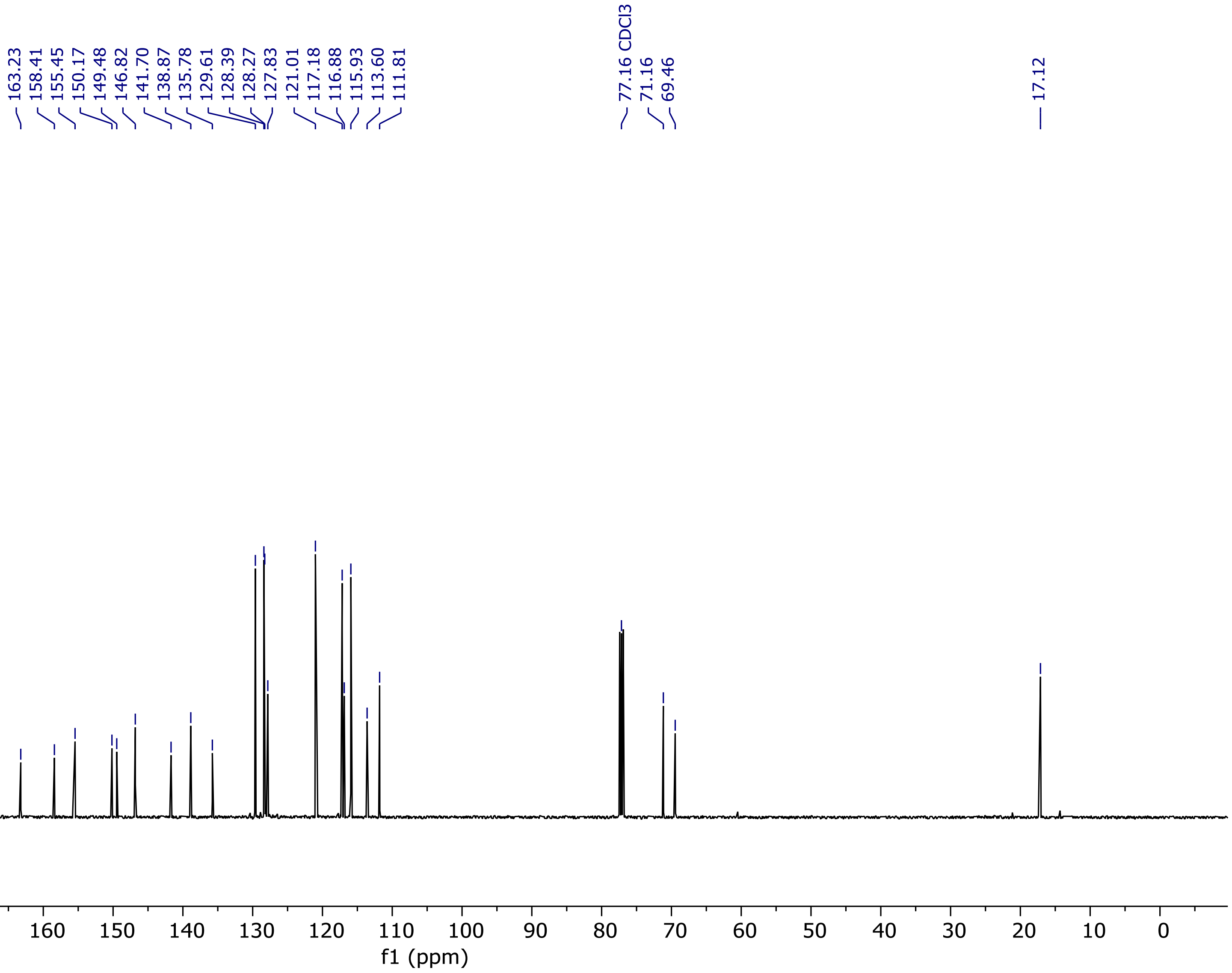

^{1}H NMR of pyriproxyphen derived morpholine 38

CD_3CN, 23 ºC

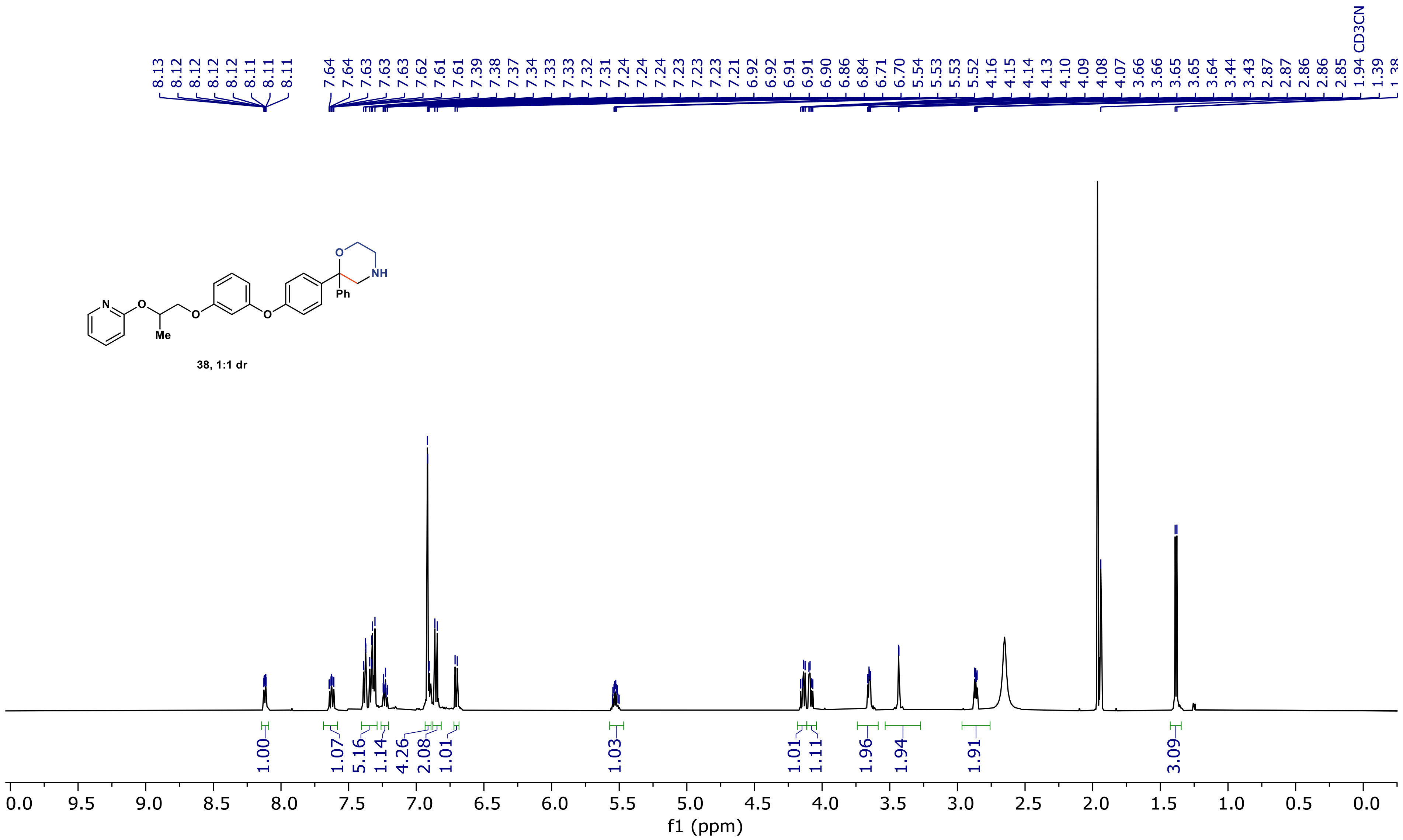

^{13}C NMR of pyriproxyphen derived morpholine 38

CD_3CN, 23 ºC

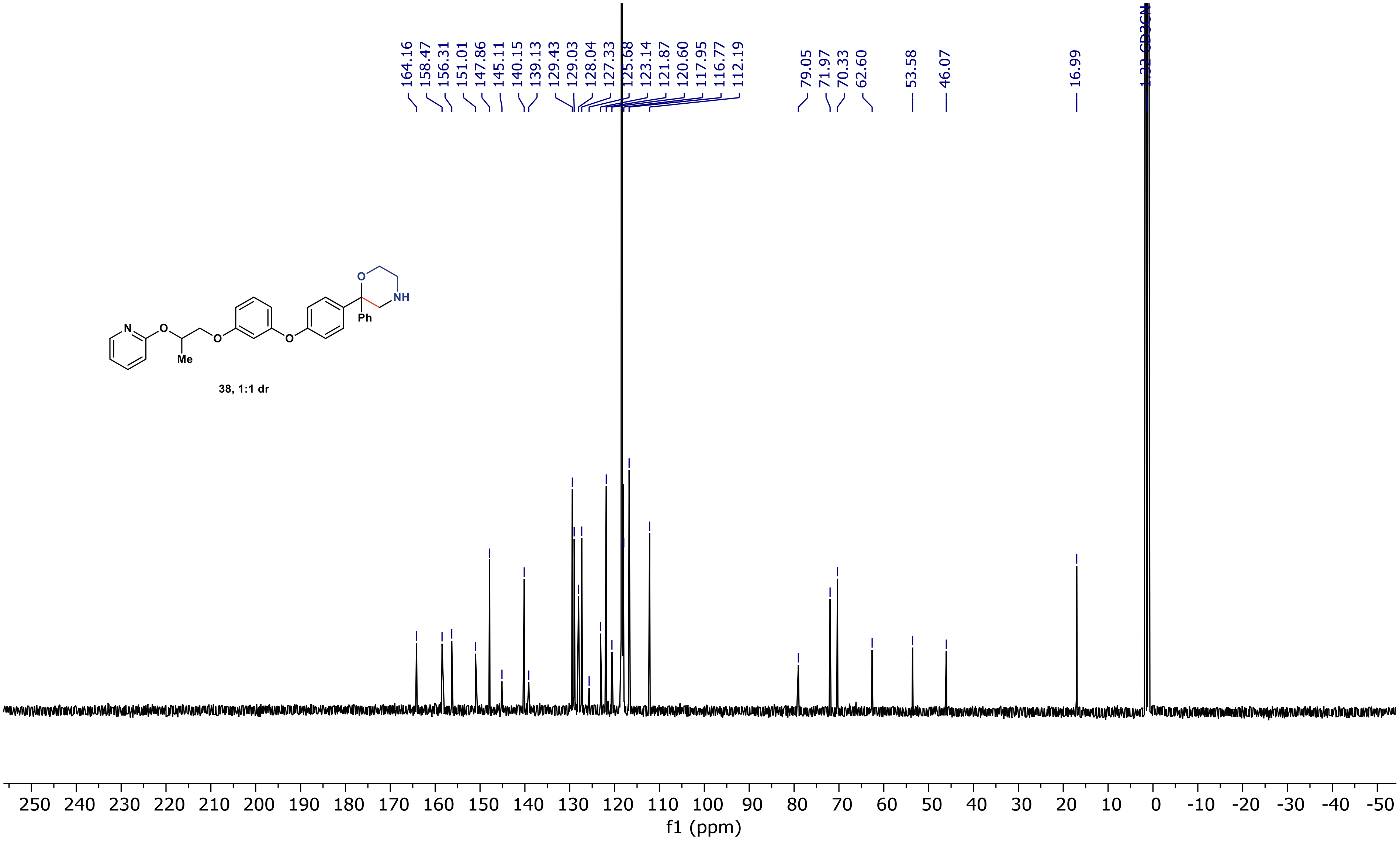

^{1}H NMR of bifonazole derived alkene S2

CDCl$_3$, 23 ºC

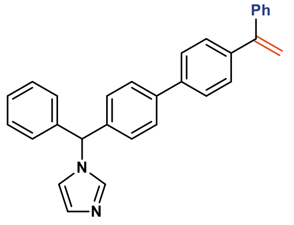

S2

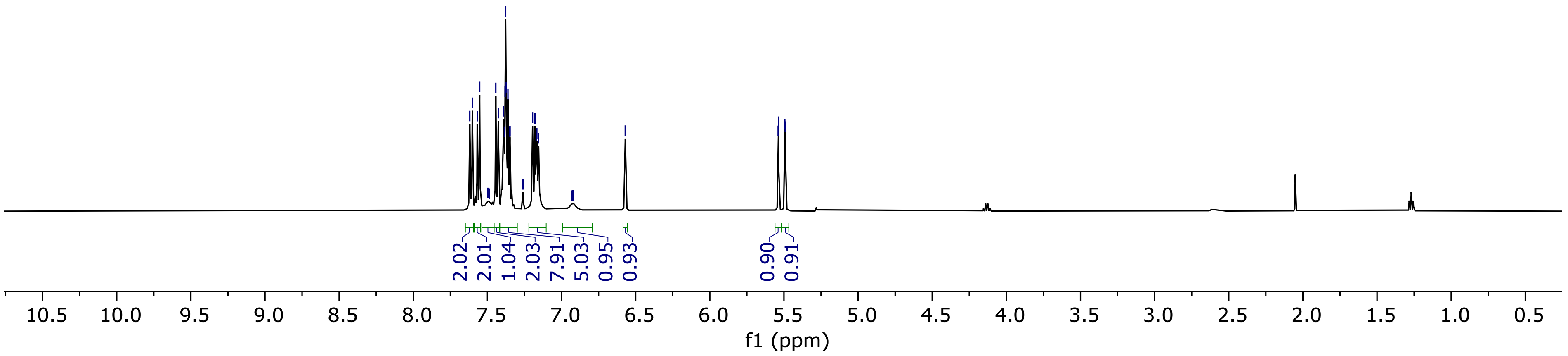

^{13}C NMR of bifonazole derived alkene S2

$CDCl_3$, 23 ºC

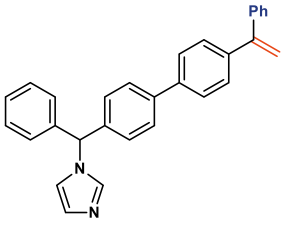

S2

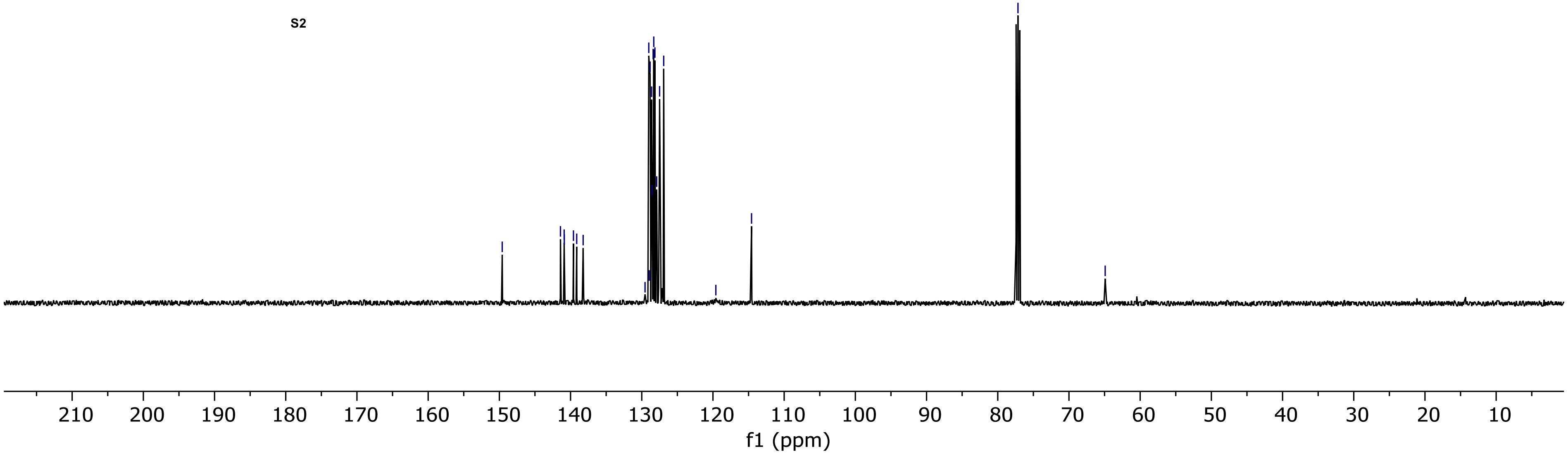

^{1}H NMR of bifonazole derived morpholine 39

$CDCl_3$, 23 ºC

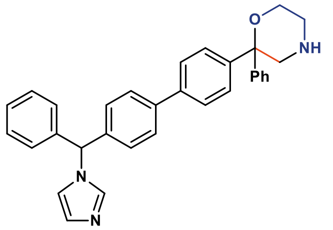
39, 1:1 dr

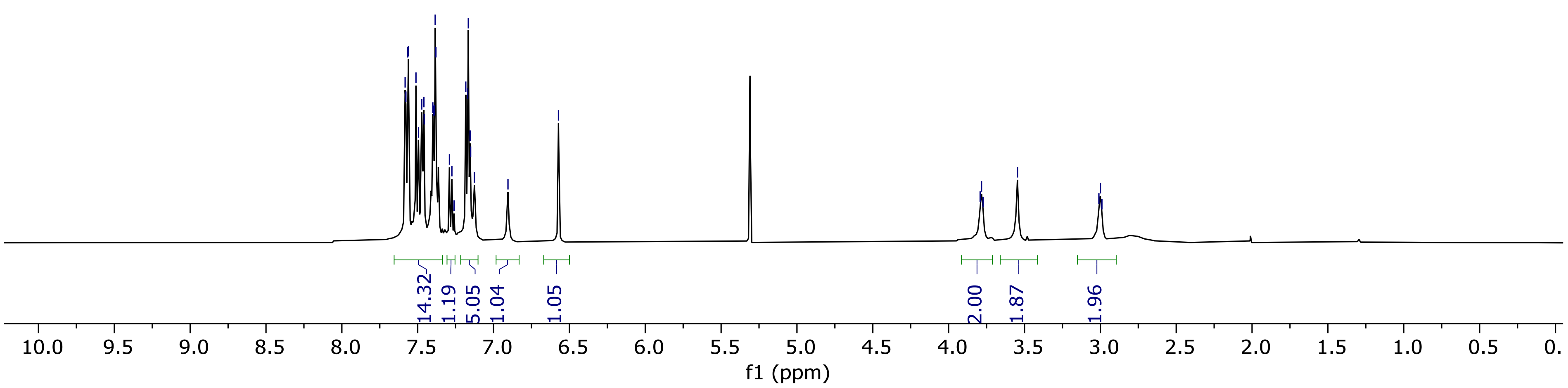

^{13}C NMR of bifonazole derived morpholine 39

CDCl$_3$, 23 ºC

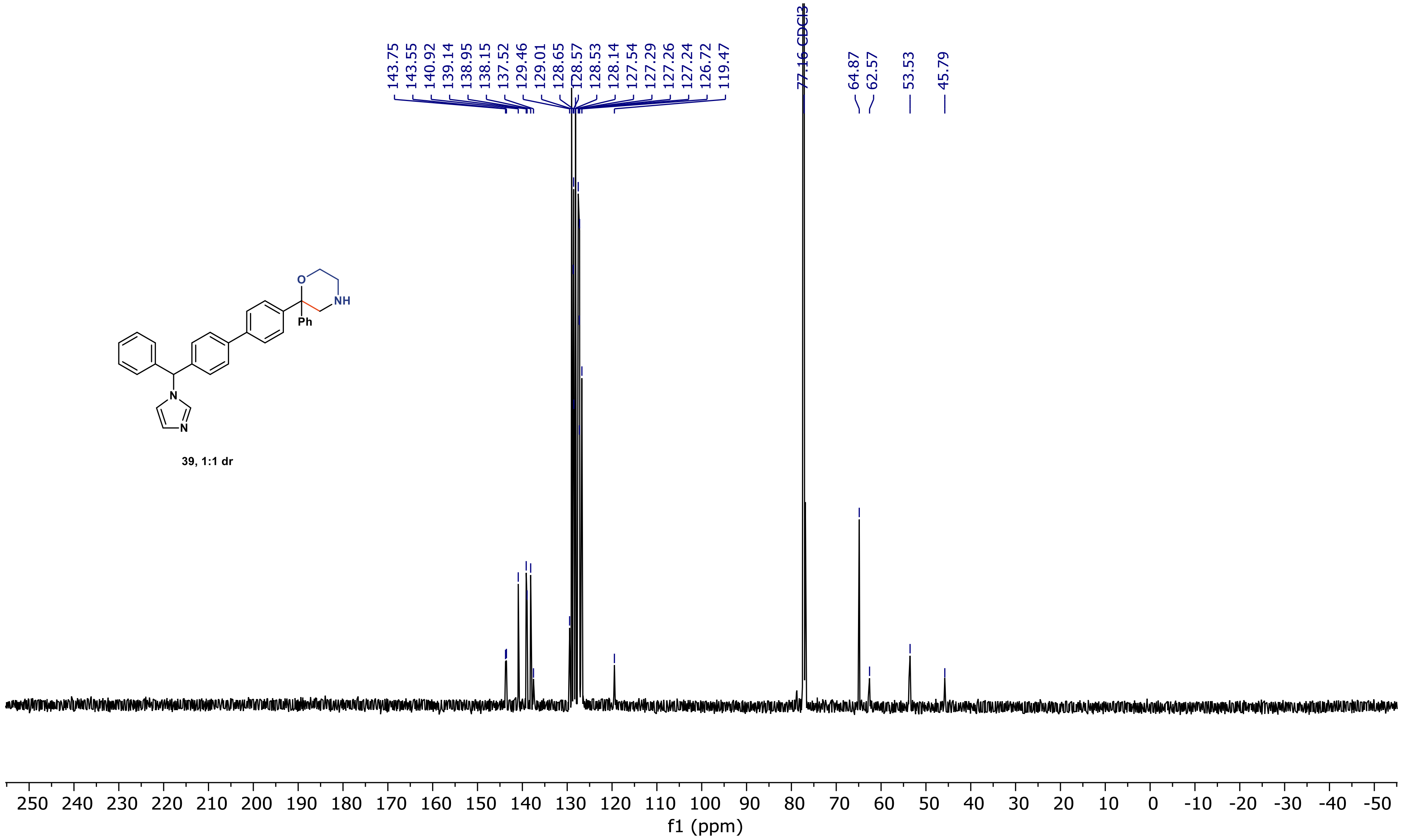

^{1}H NMR of dihydrodibenzothiepine 40

$CDCl_3$, 23 ºC

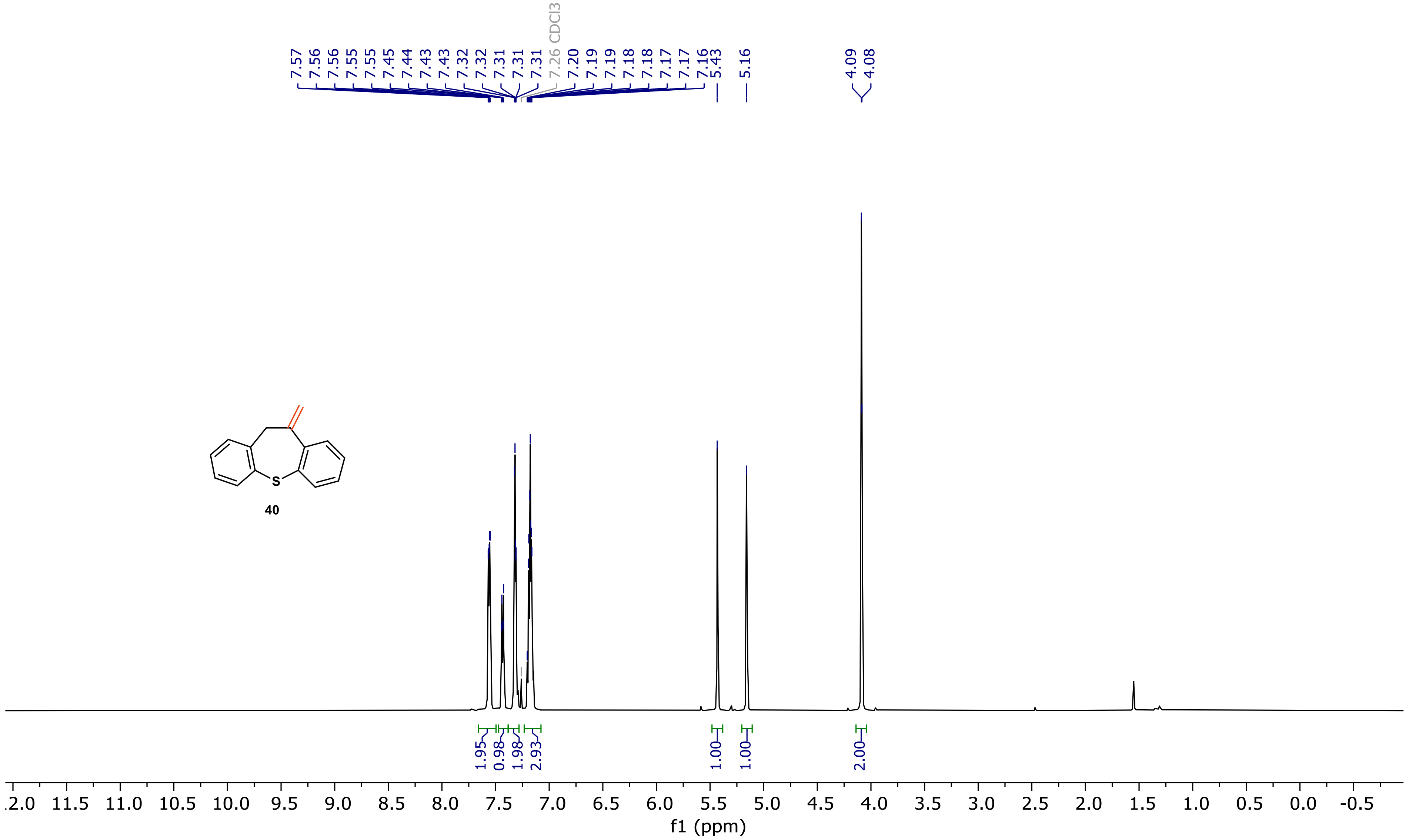

^{13}C NMR of dihydrodibenzothiepine 40

CD_3CN, 23 ºC

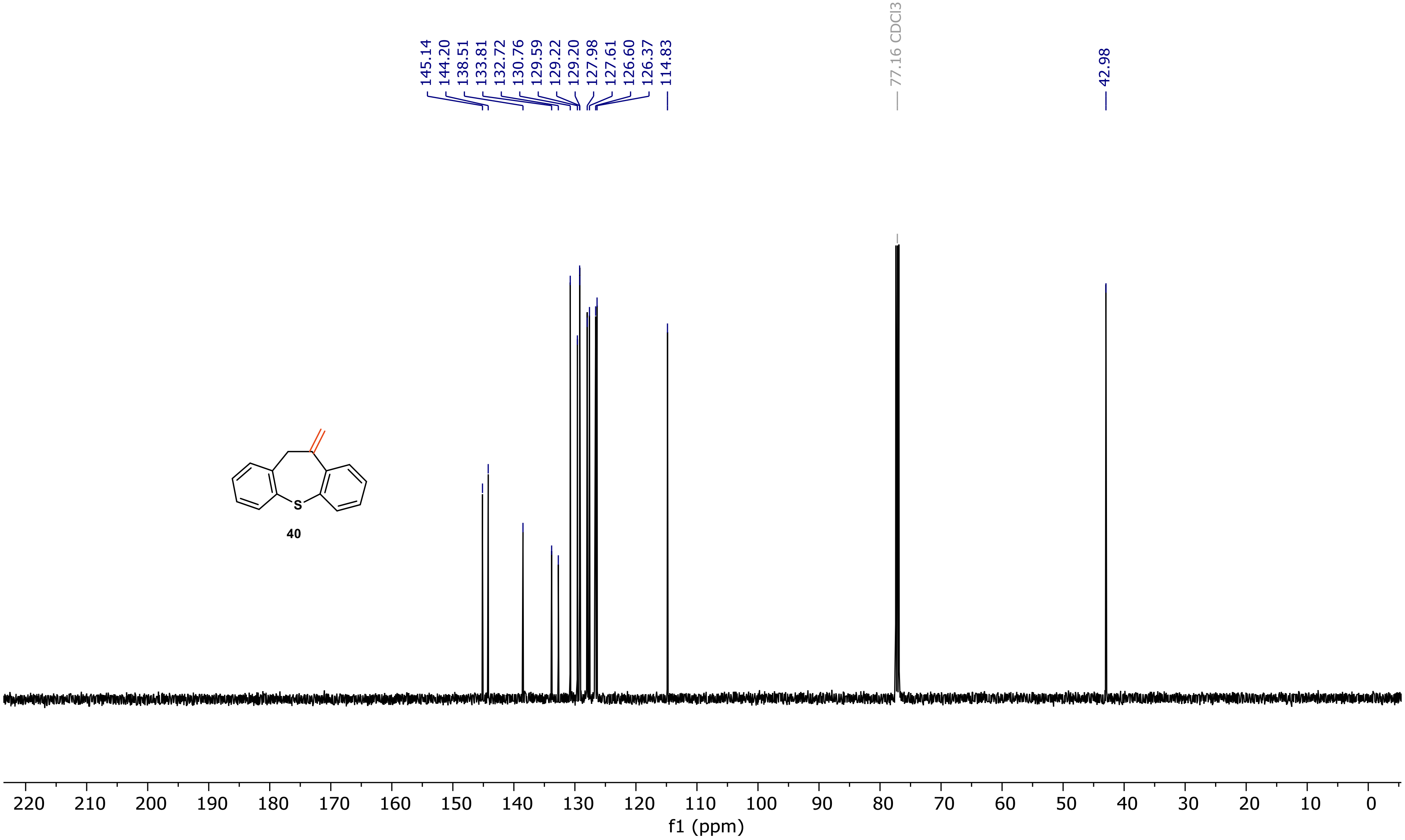

^{1}H NMR of spiro[dibenzothiepine-10,2'-morpholine] 41

CDCl3, 23 ºC

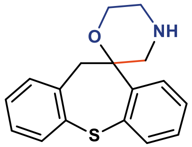

41

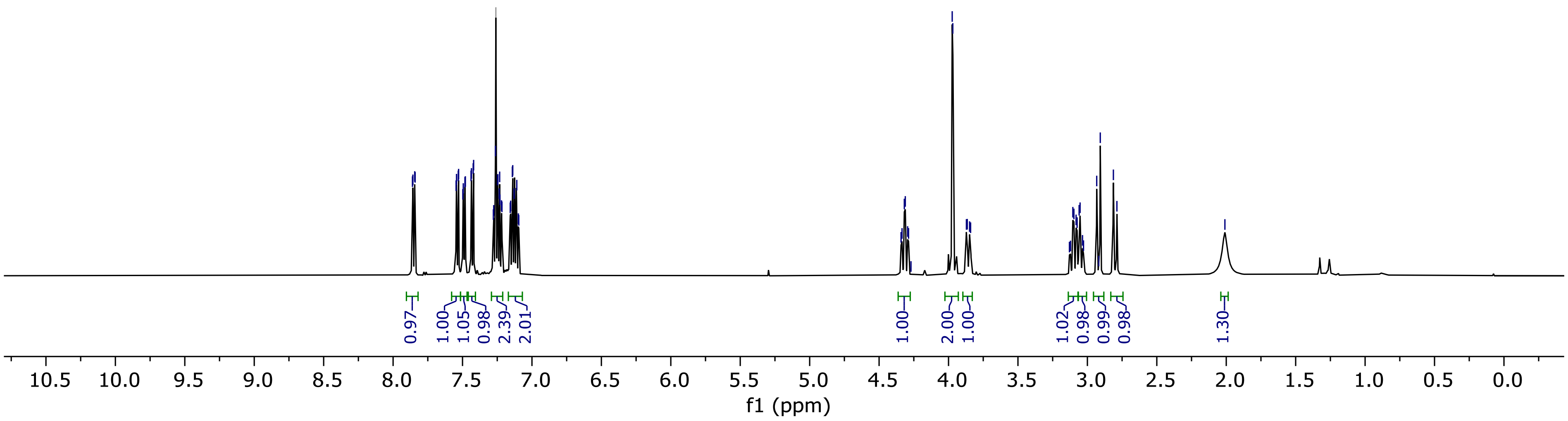

^{13}C NMR of spiro[dibenzothiepine-10,2'-morpholine] 41

CDCl$_3$, 23 ºC

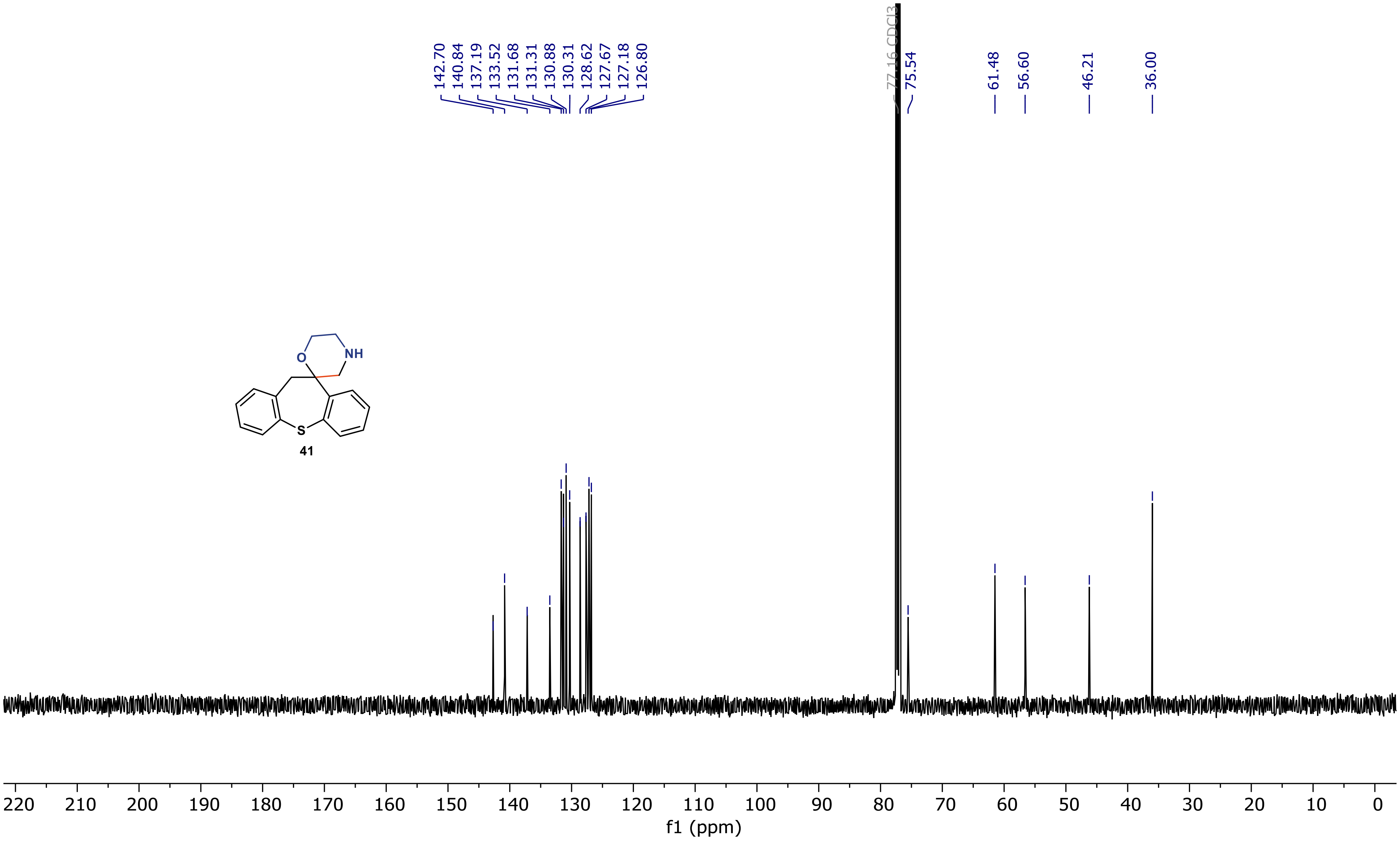

^{1}H NMR of 2-methyl-6-phenylmorpholine 42

$CDCl_3$, 23 ºC

a mixture of diastereoisomers with 2:1 dr

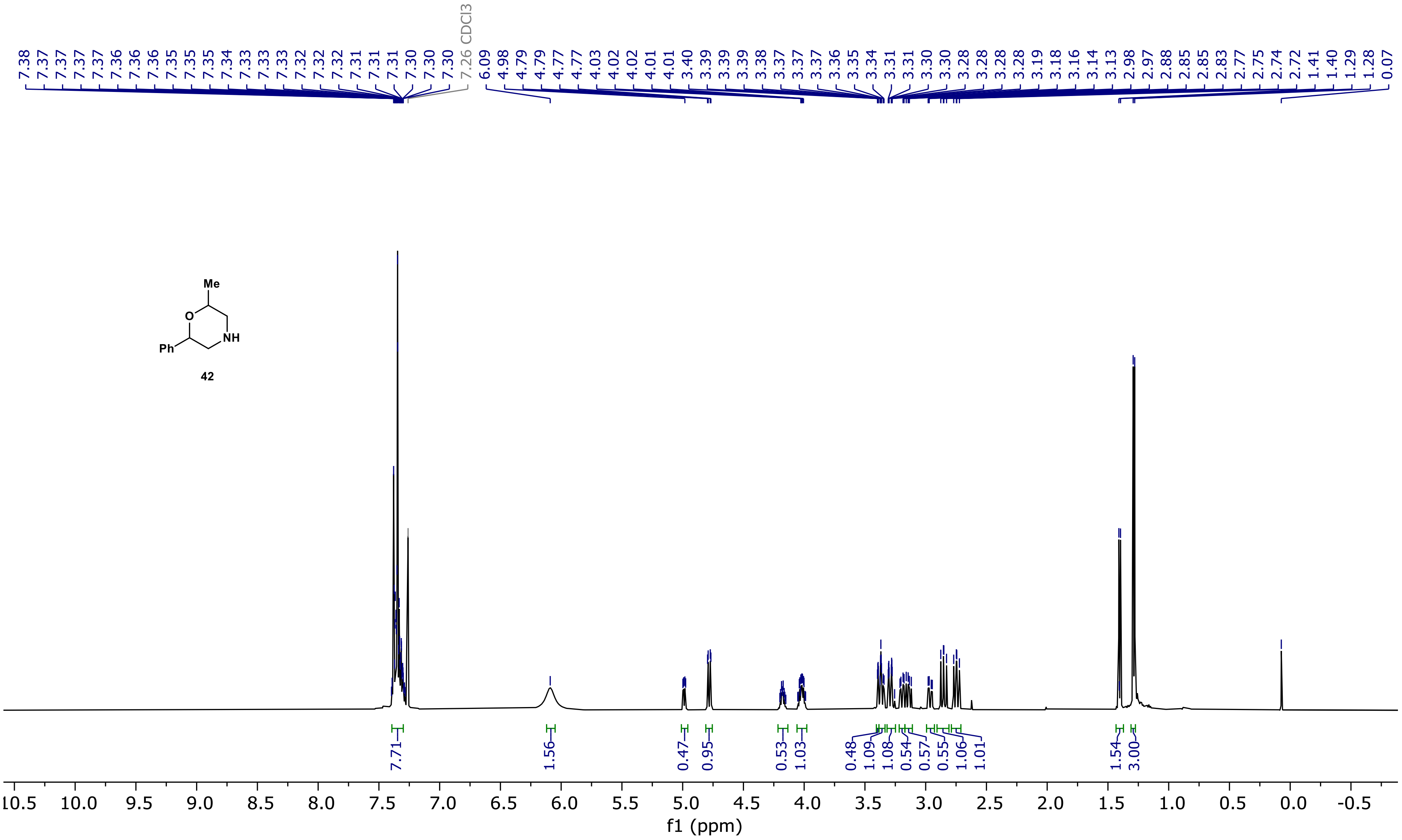

^{13}C NMR of 2-methyl-6-phenylmorpholine 42

$CDCl_3$, 23 ºC

a mixture of diastereoisomers with 2:1 dr

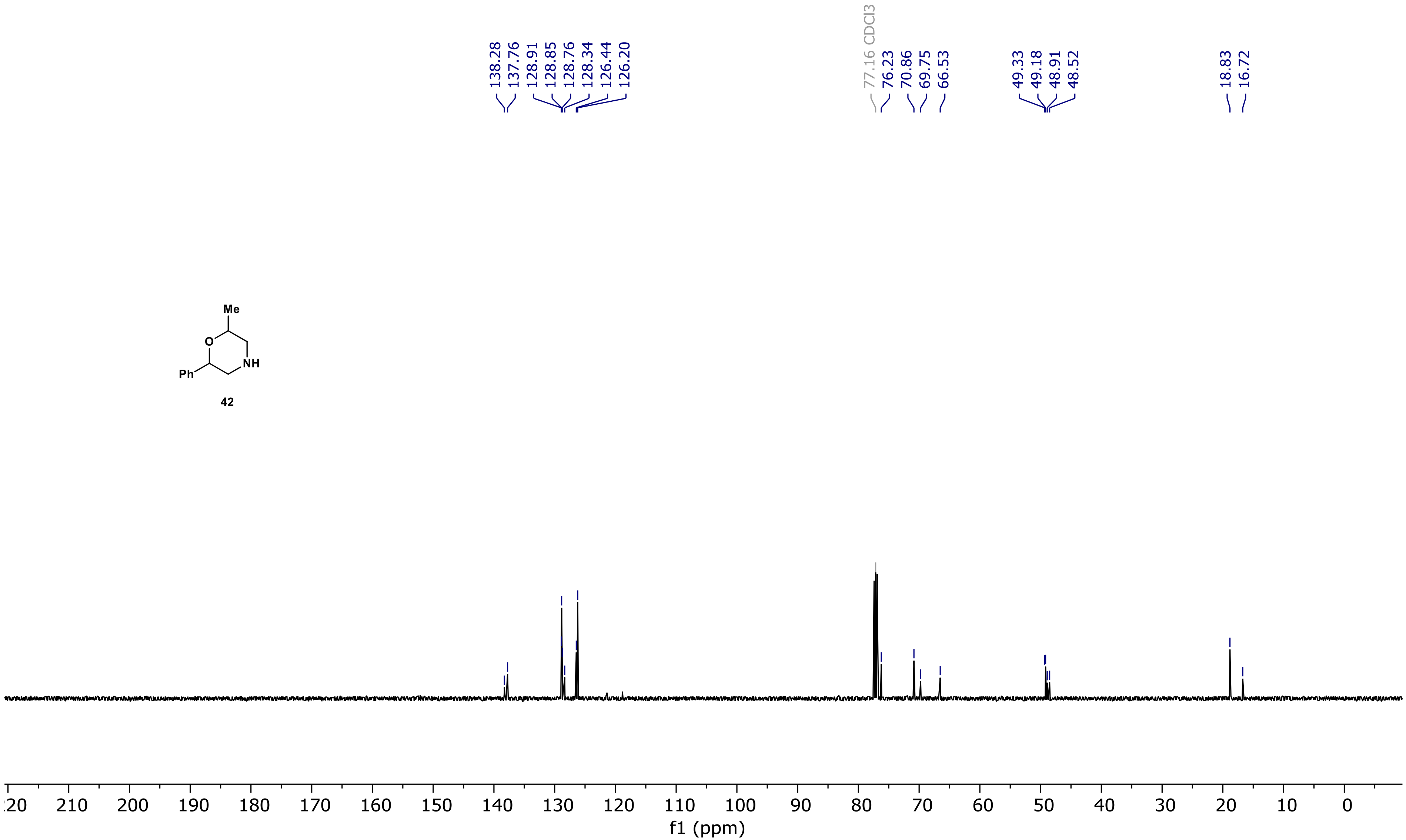

COSY of 2-methyl-6-phenylmorpholine 42

$CDCl_3$, 23 ºC

a mixture of diastereoisomers with 2:1 dr

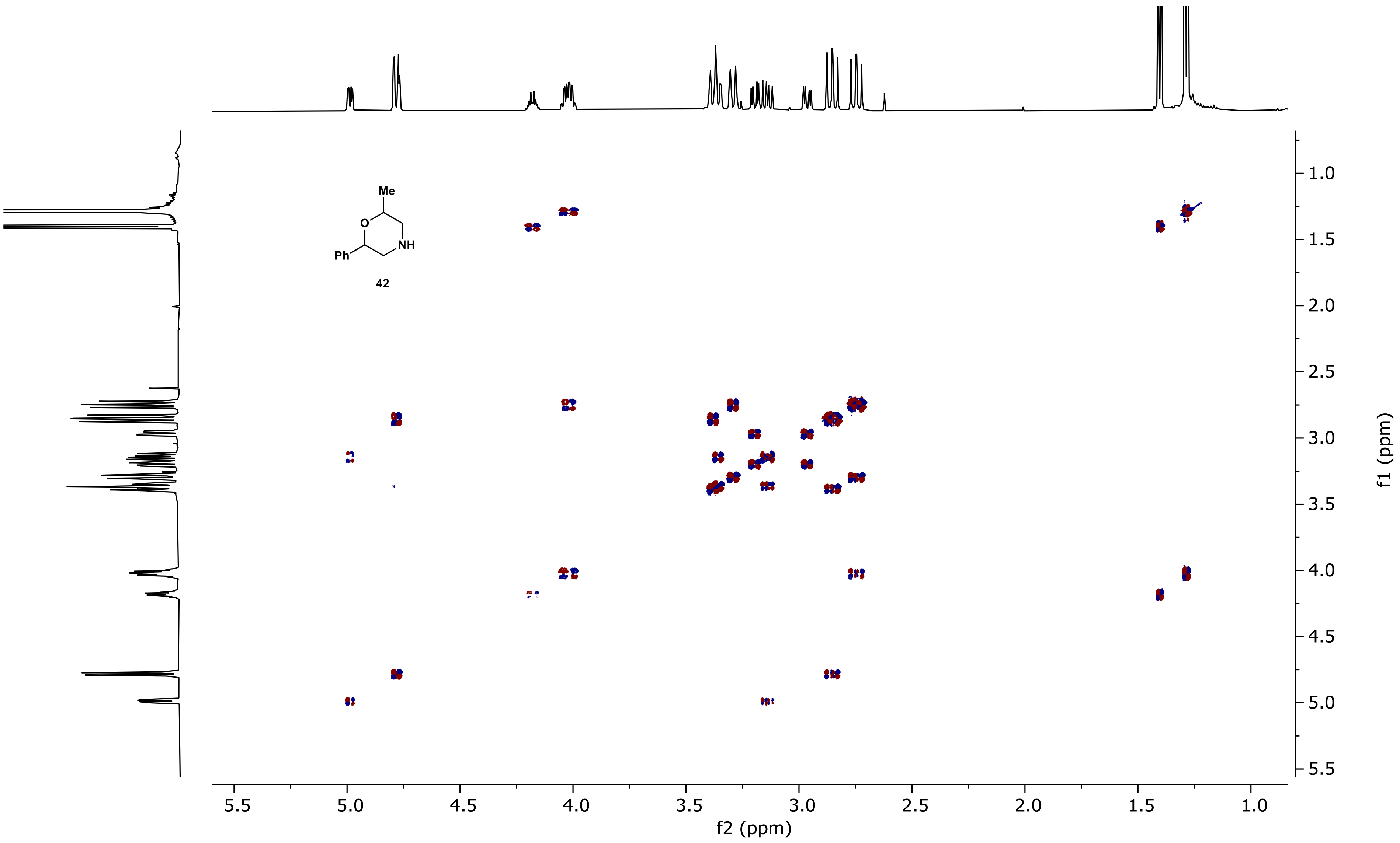

HSQC of 2-methyl-6-phenylmorpholine 42

$CDCl_3$, 23 ºC

a mixture of diastereoisomers with 2:1 dr

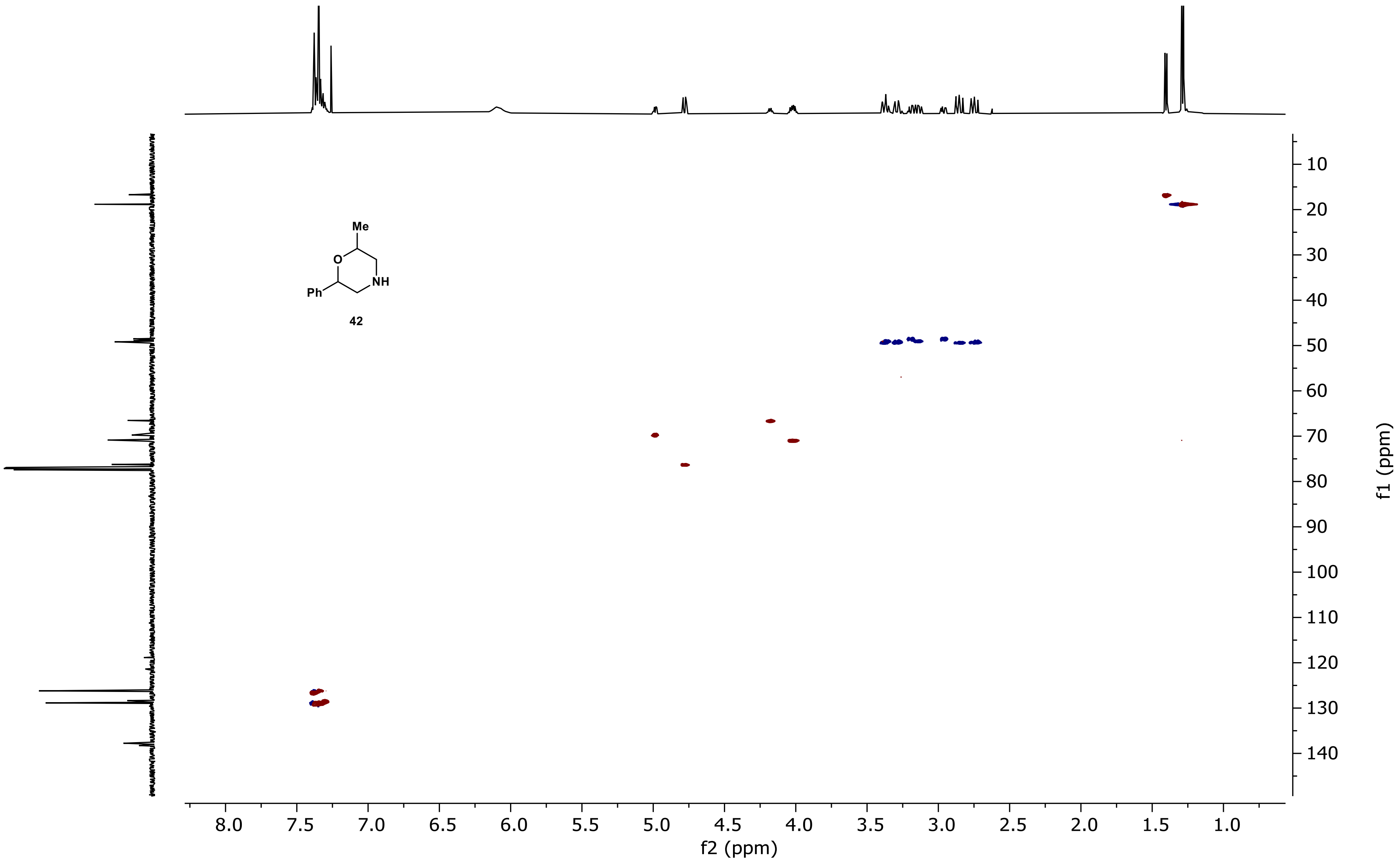

HMBC of 2-methyl-6-phenylmorpholine 42

$CDCl_3$, 23 ºC

a mixture of diastereoisomers with 2:1 dr

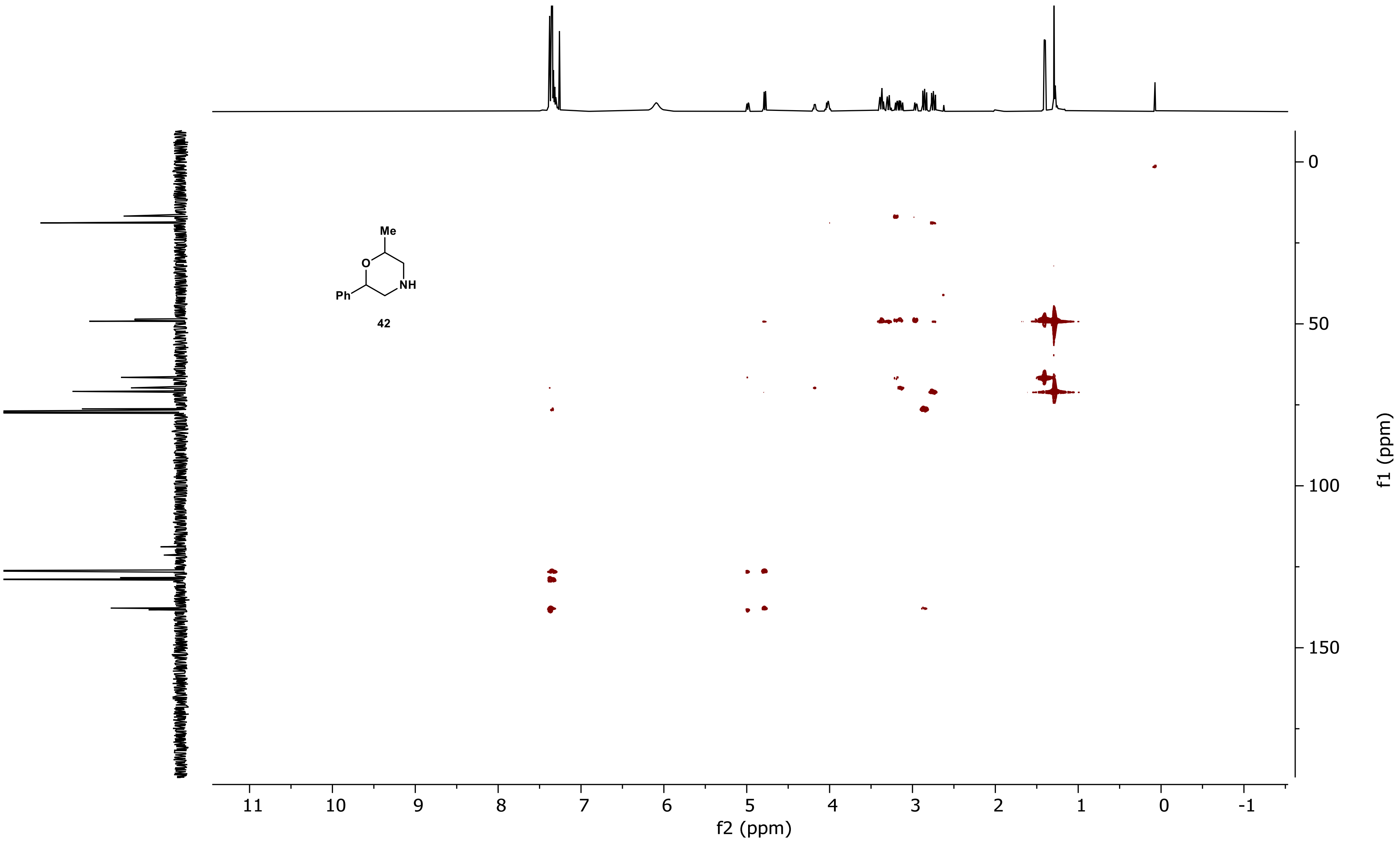

NOESY of 2-methyl-6-phenylmorpholine 42

$CDCl_3$, 23 ºC

a mixture of diastereoisomers with 2:1 dr

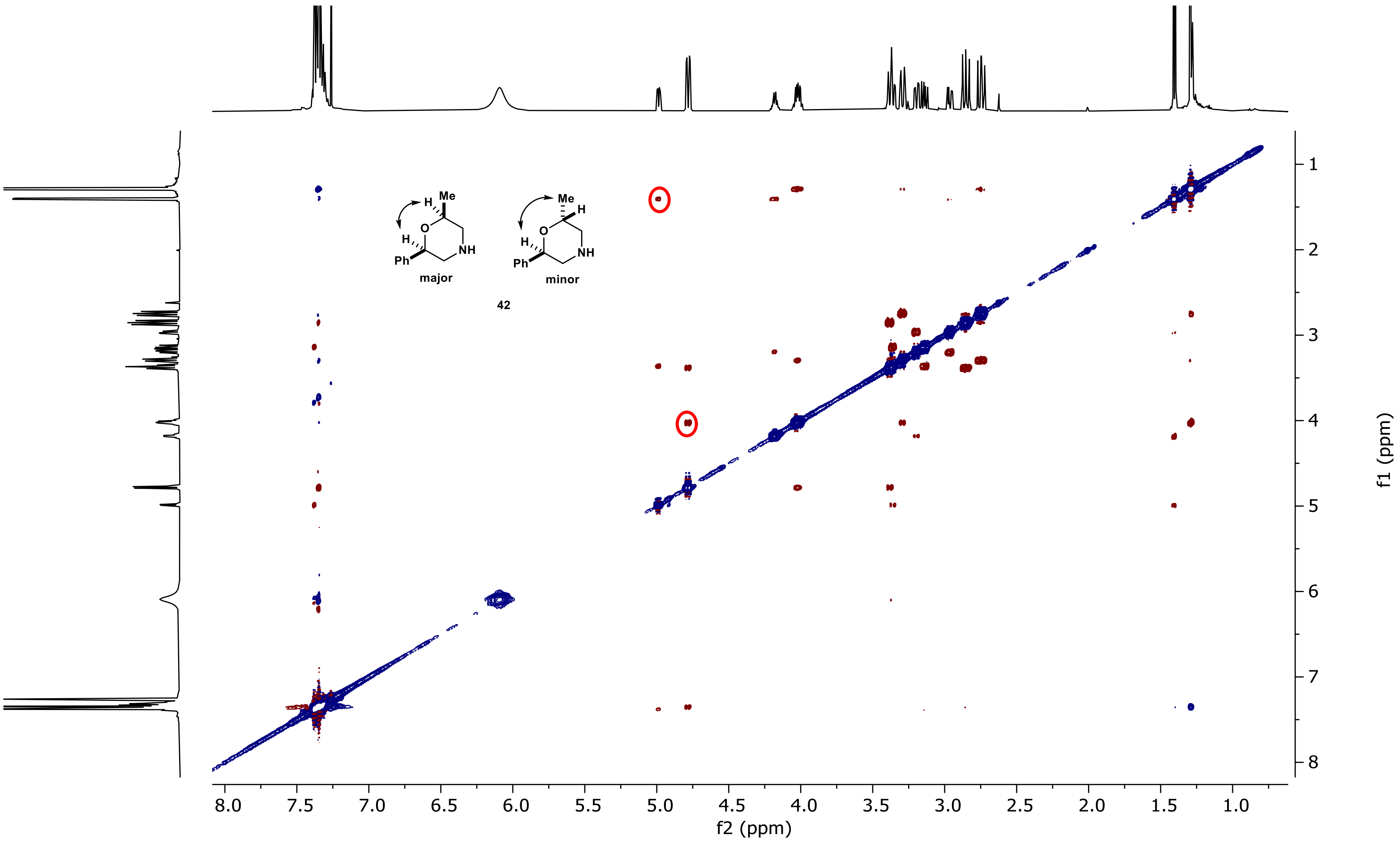

^{1}H NMR of 5-ethyl-2-phenylmorpholine 43

CD_3CN, 23 ºC

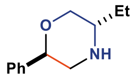

43

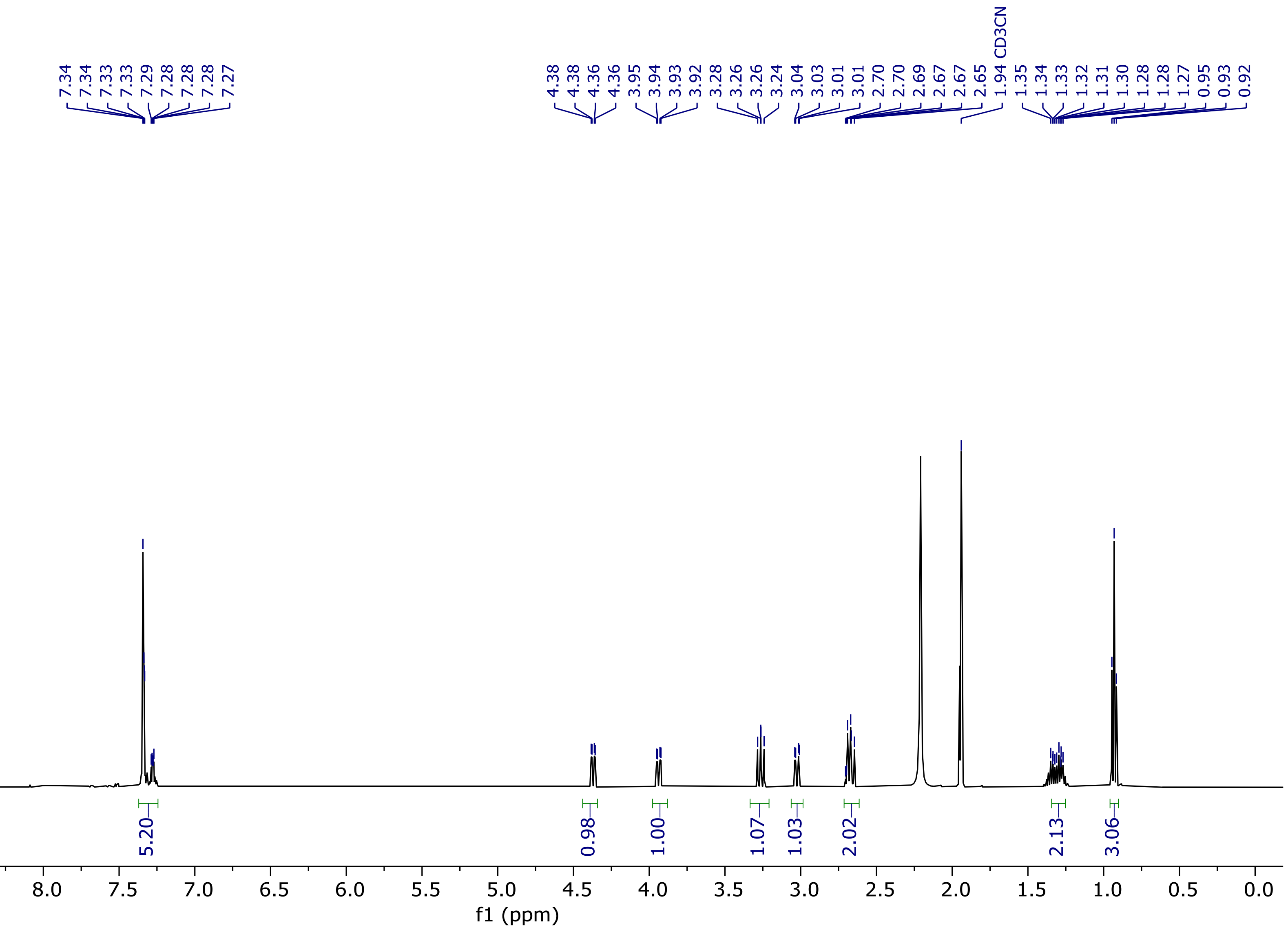

^{13}C NMR of 5-ethyl-2-phenylmorpholine 43

CD_3CN, 23 ºC

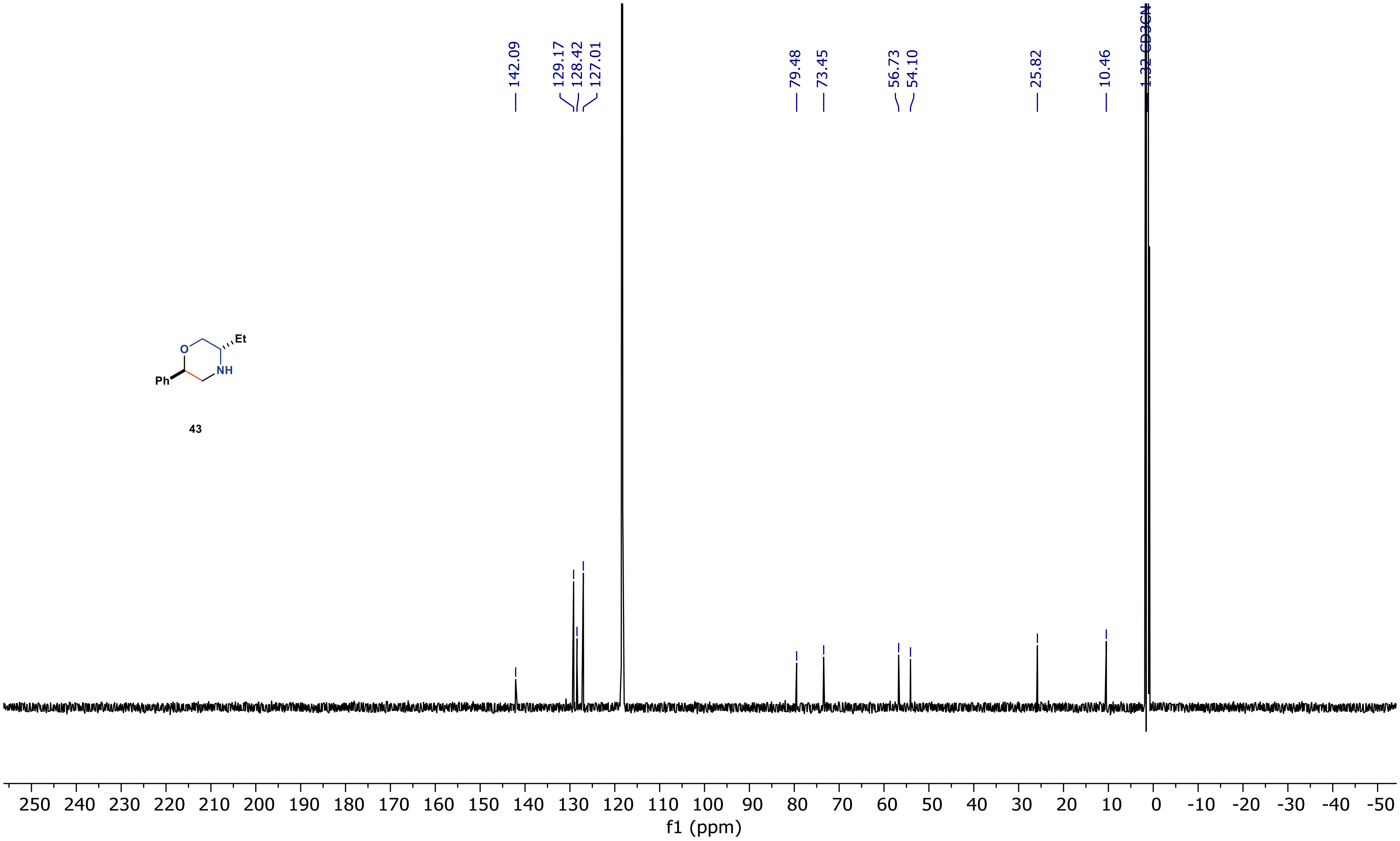

COSY of 5-ethyl-2-phenylmorpholine 43

CD_3CN, 23 ºC

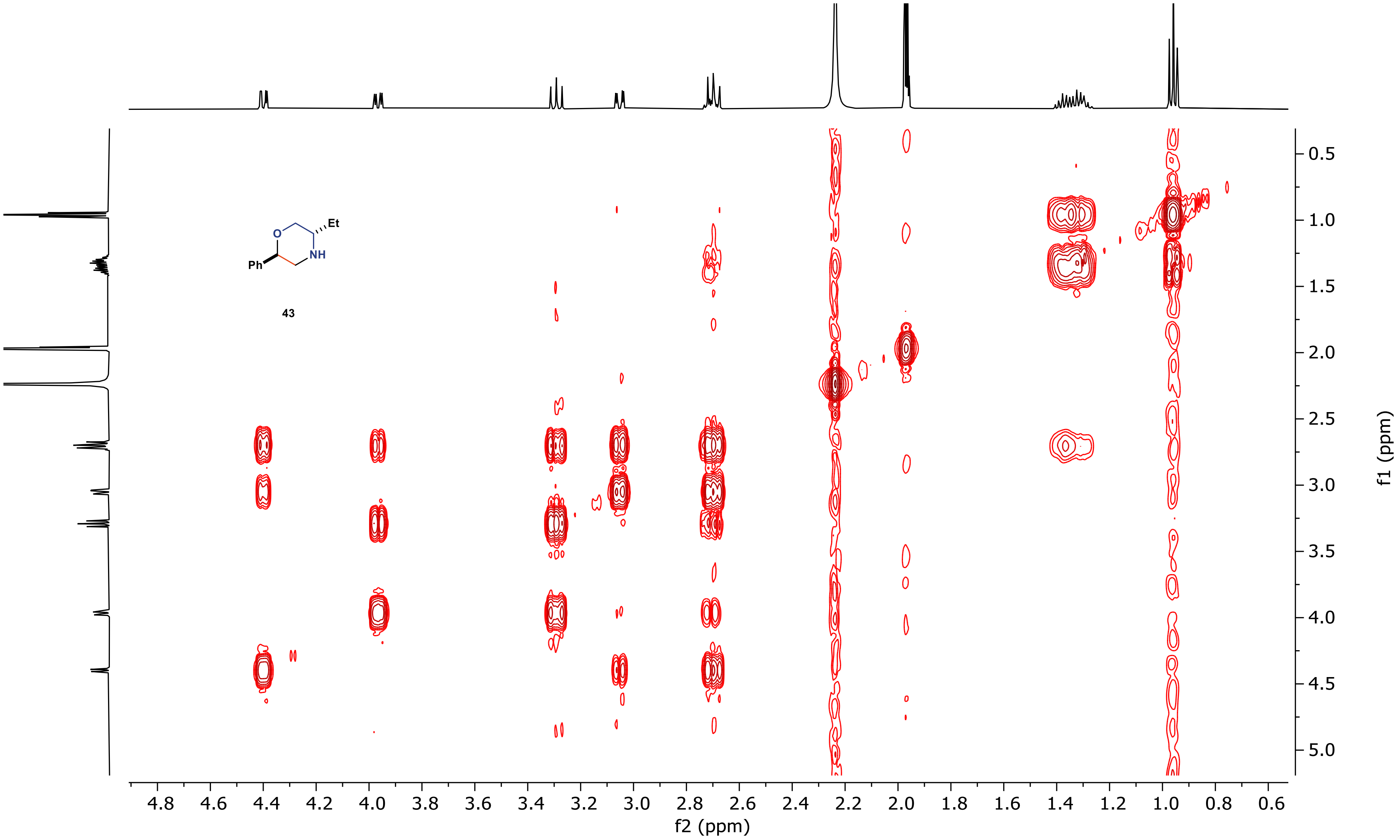

HSQC of 5-ethyl-2-phenylmorpholine 43

CD_3CN, 23 ºC

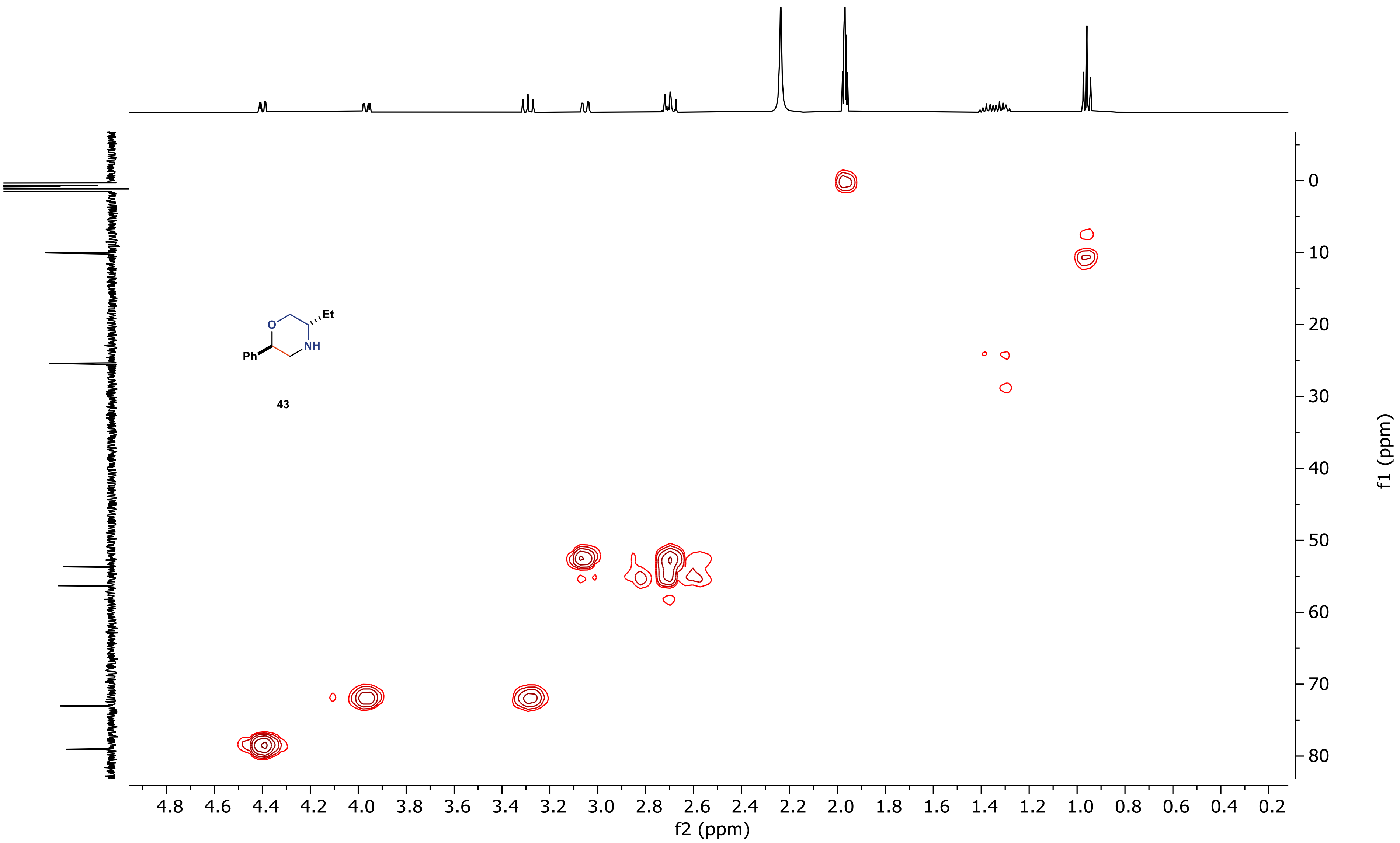

NOESY of 5-ethyl-2-phenylmorpholine 43

CD3CN, 23 ºC

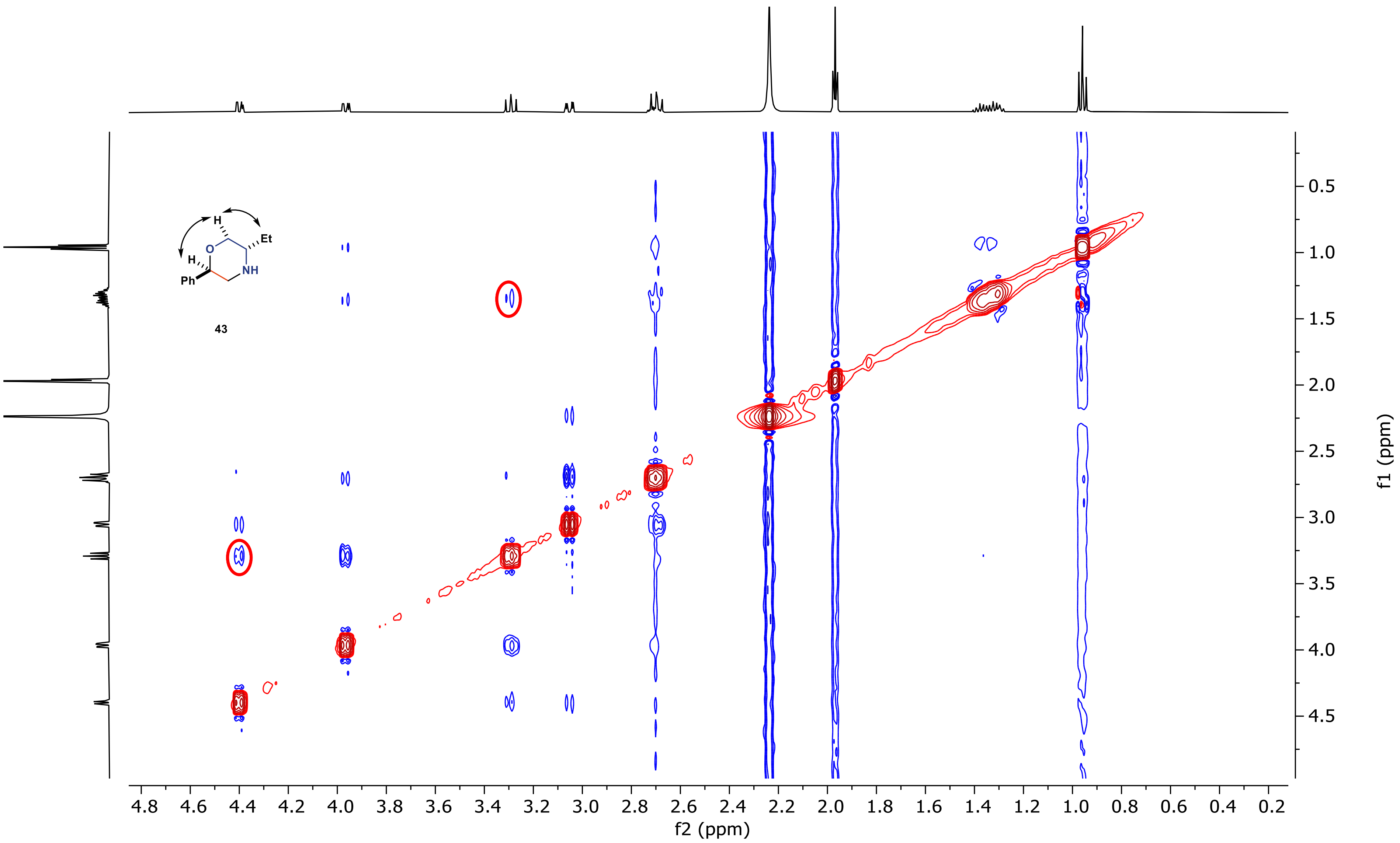

^{1}H NMR of 2,2-diphenyl-1,4-oxazepane 44

$CDCl_3$, 23 ºC

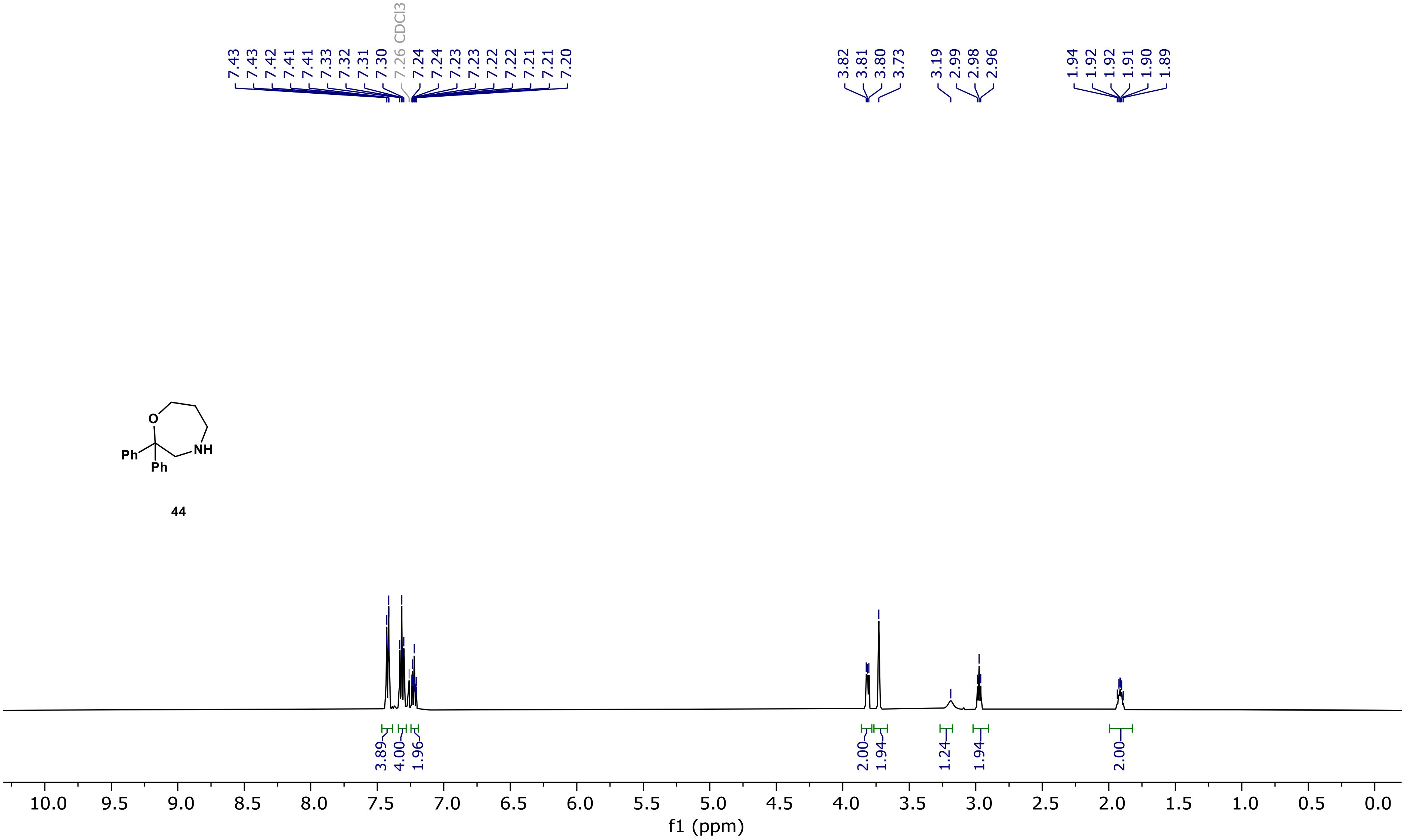

^{13}C NMR of 2,2-diphenyl-1,4-oxazepane 44

$CDCl_3$, 23 ºC

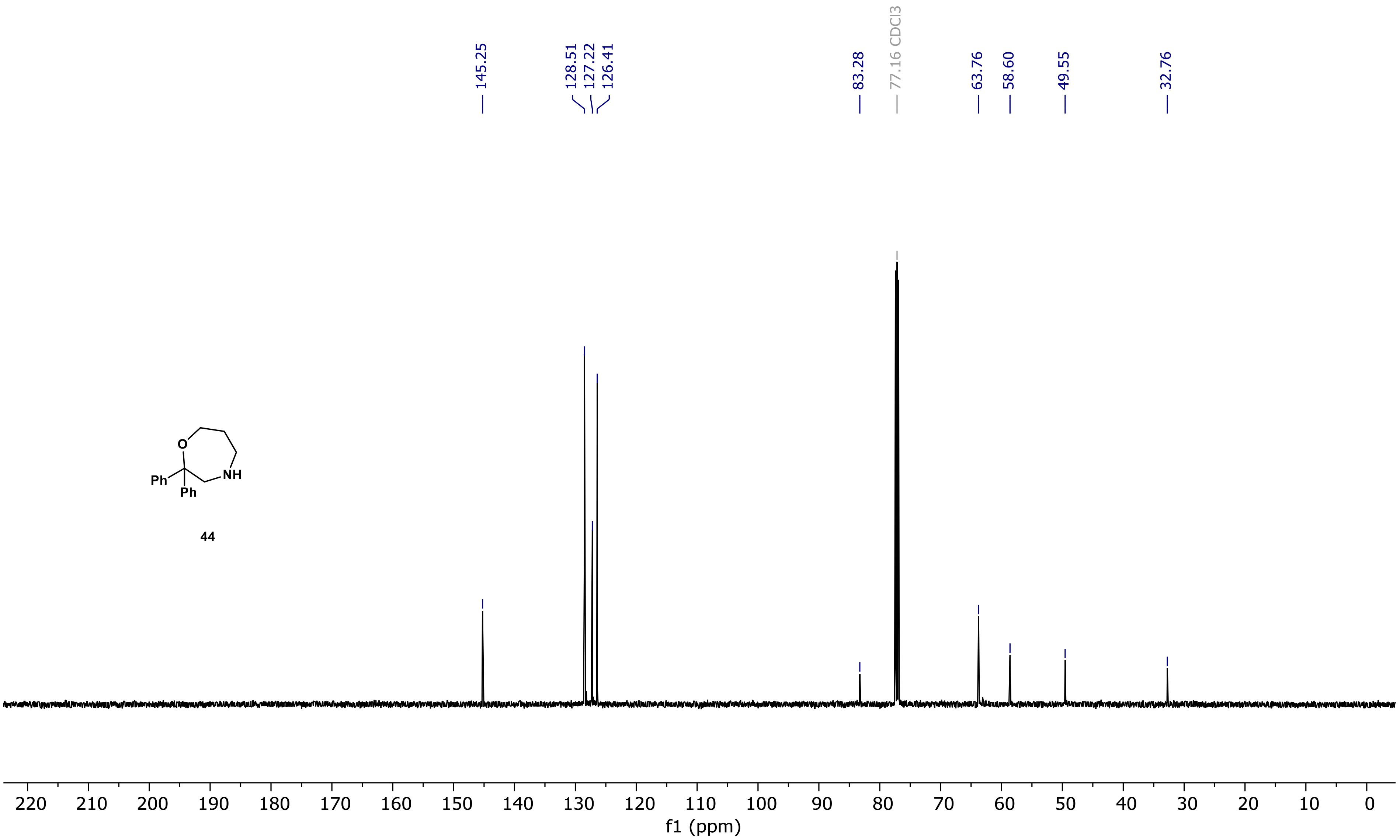

^{1}H NMR of 6,6-dimethyl-2-phenyl-1,4-oxazepane 45

CD_3CN, 23 ºC

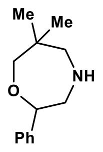

45

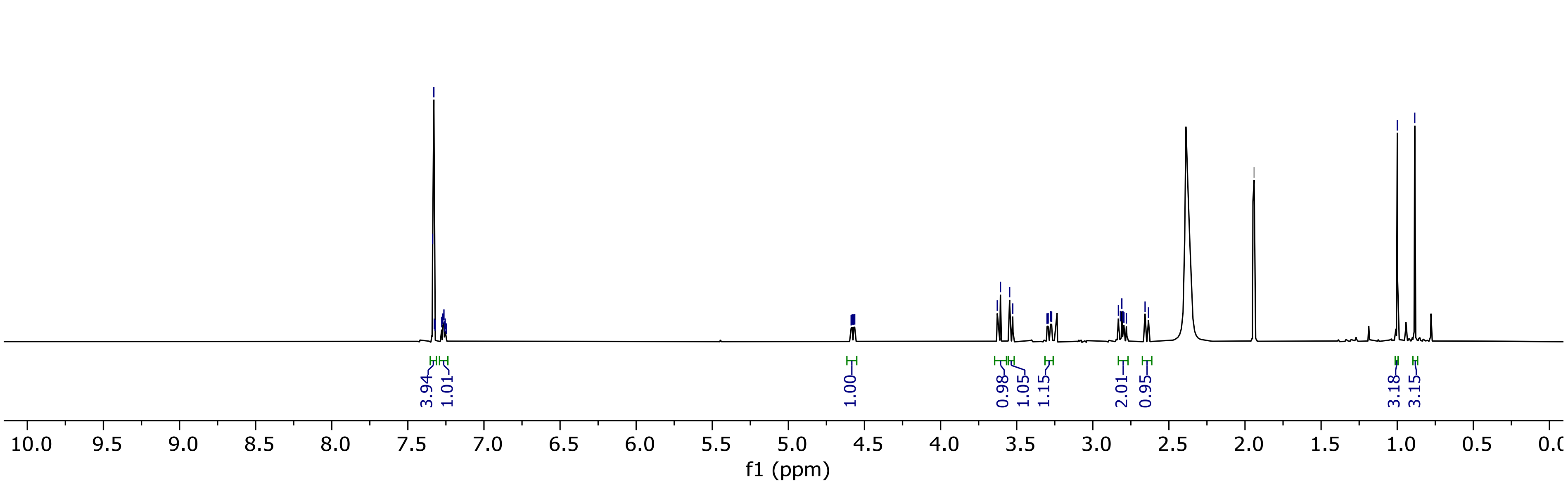

^{13}C NMR of 6,6-dimethyl-2-phenyl-1,4-oxazepane 45

CD_3CN, 23 ºC

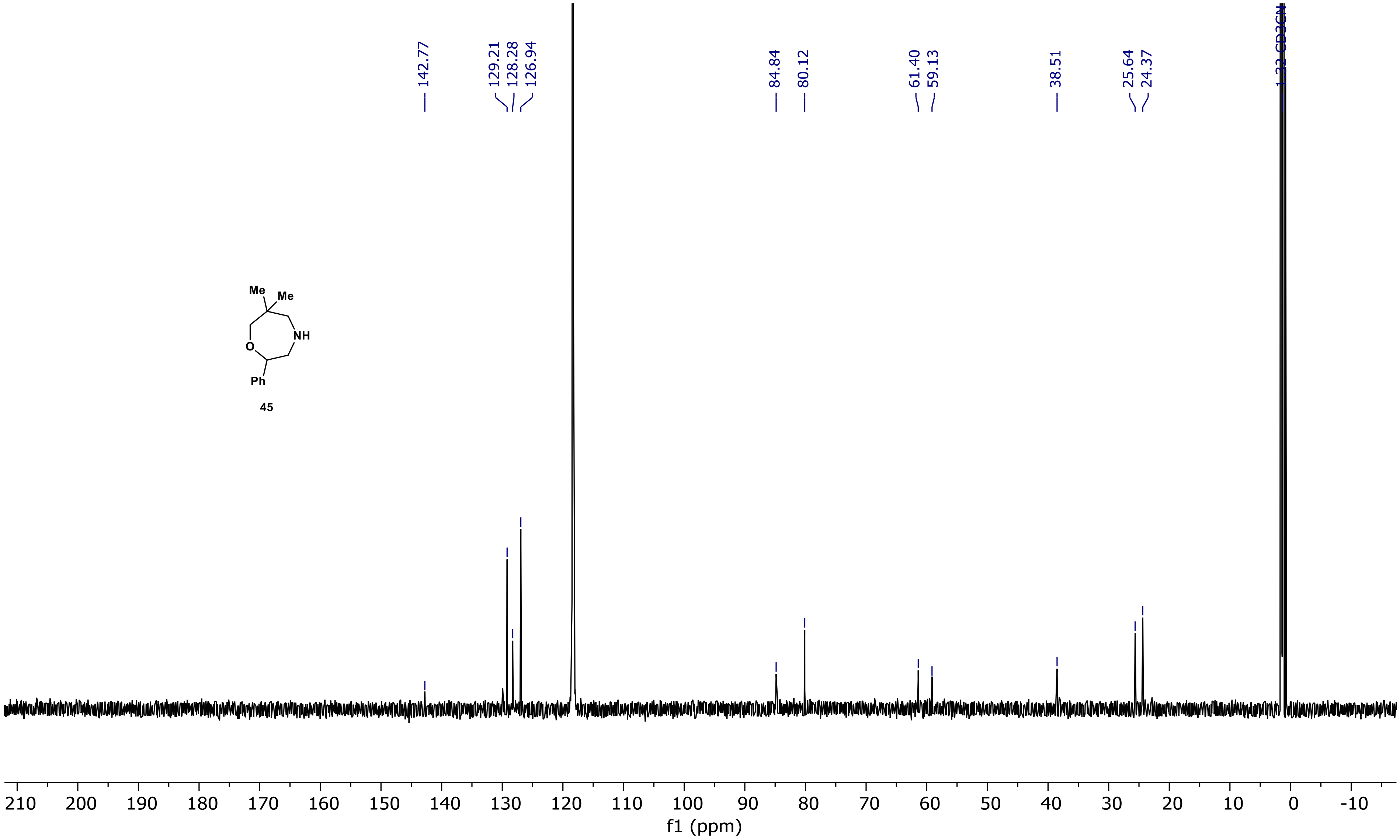

^{1}H NMR of N-phenylsulfonylpiperazine 46

CDCl$_3$, 23 ºC

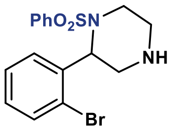

46

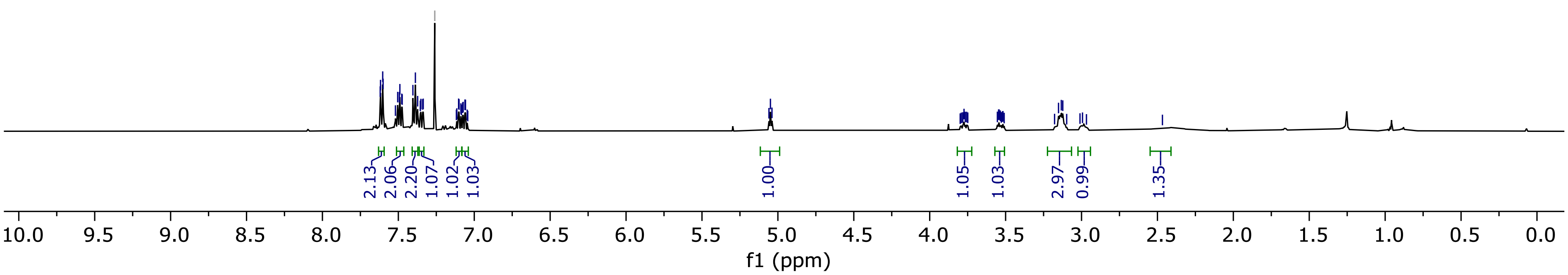

^{13}C NMR of N-phenylsulfonylpiperazine 46

$CDCl_3$, 23 ºC

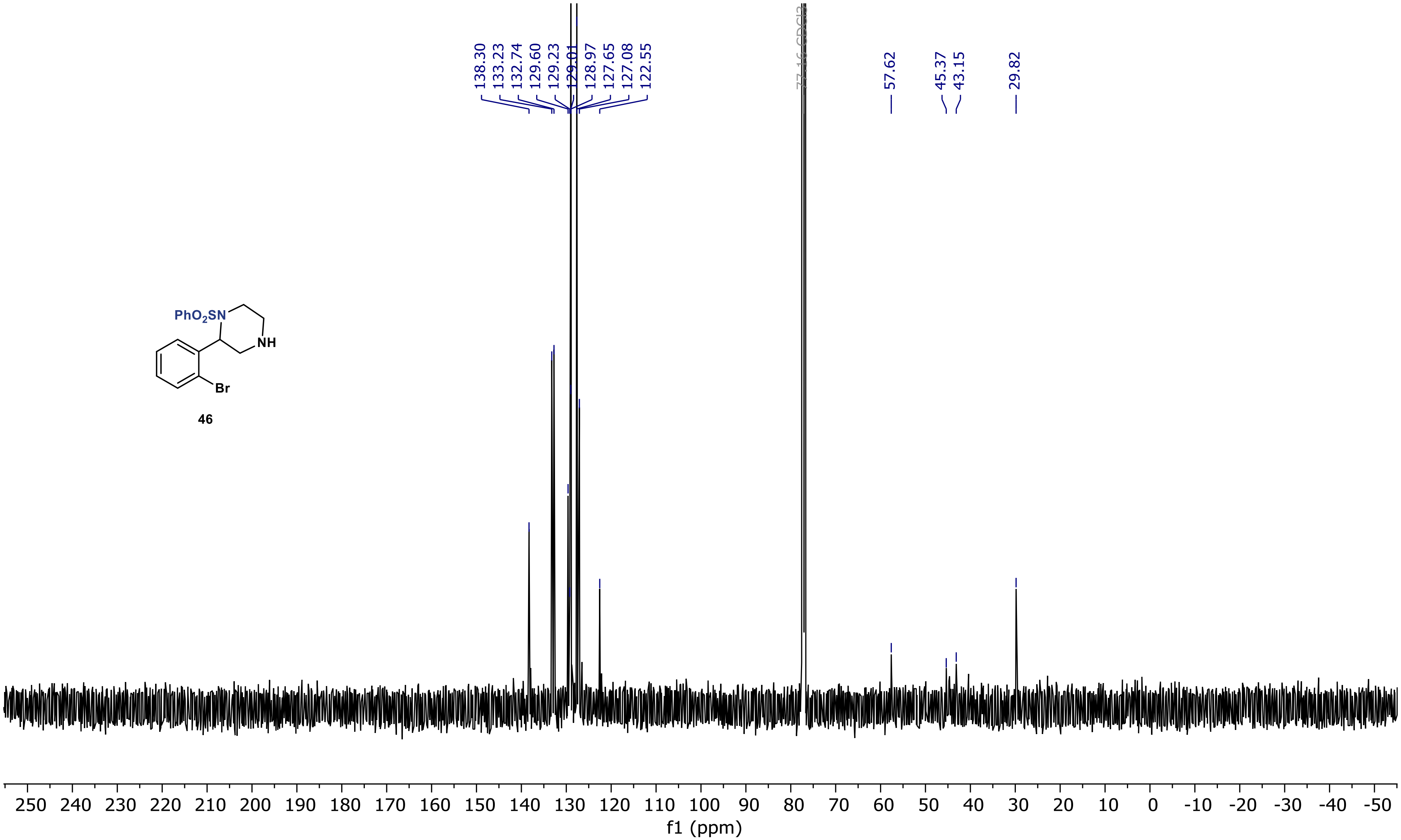

^{1}H NMR of N-Cbz-piperazine 47

CDCl$_3$, 23 ºC

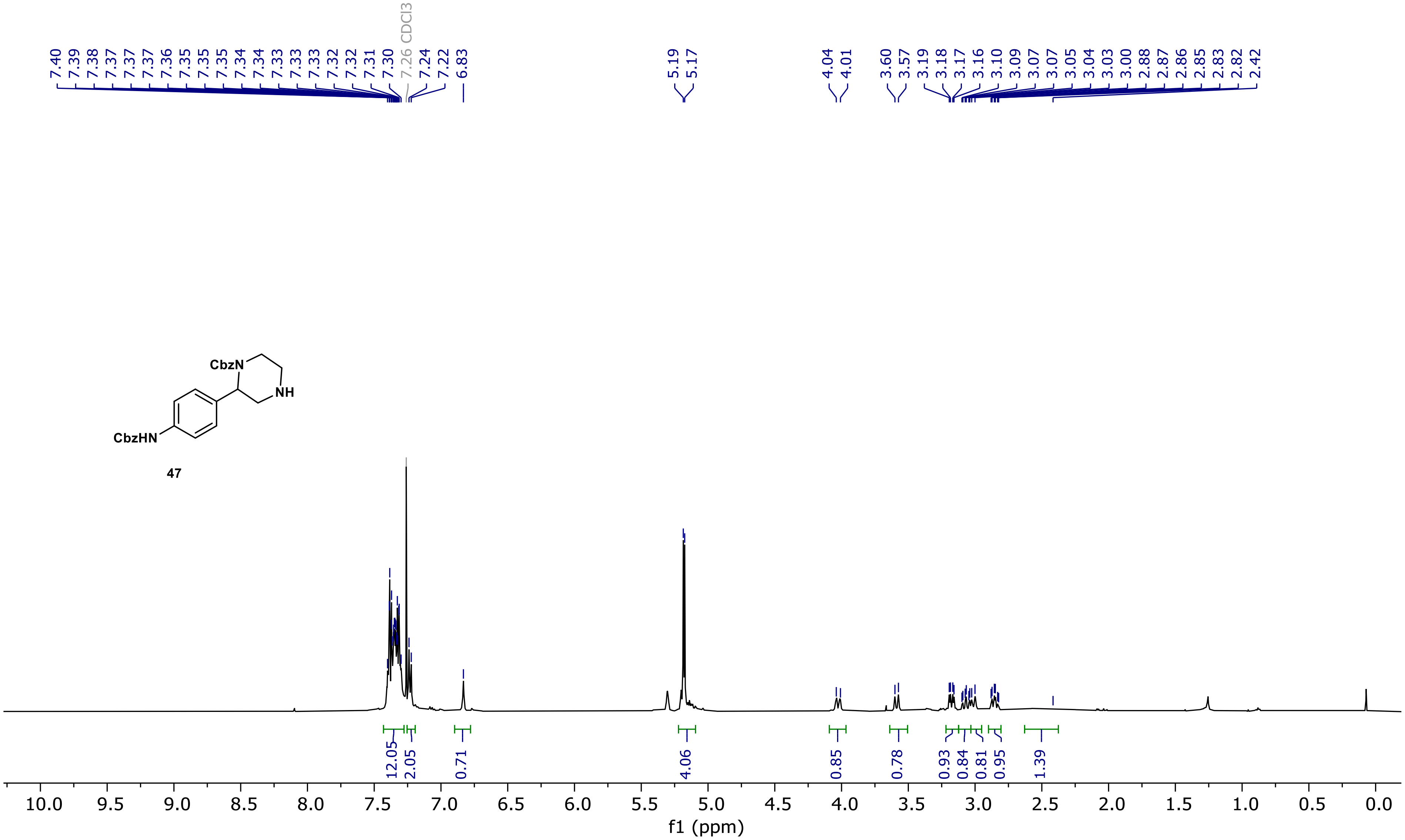

^{13}C NMR of N-Cbz-piperazine 47

$CDCl_3$, 23 ºC

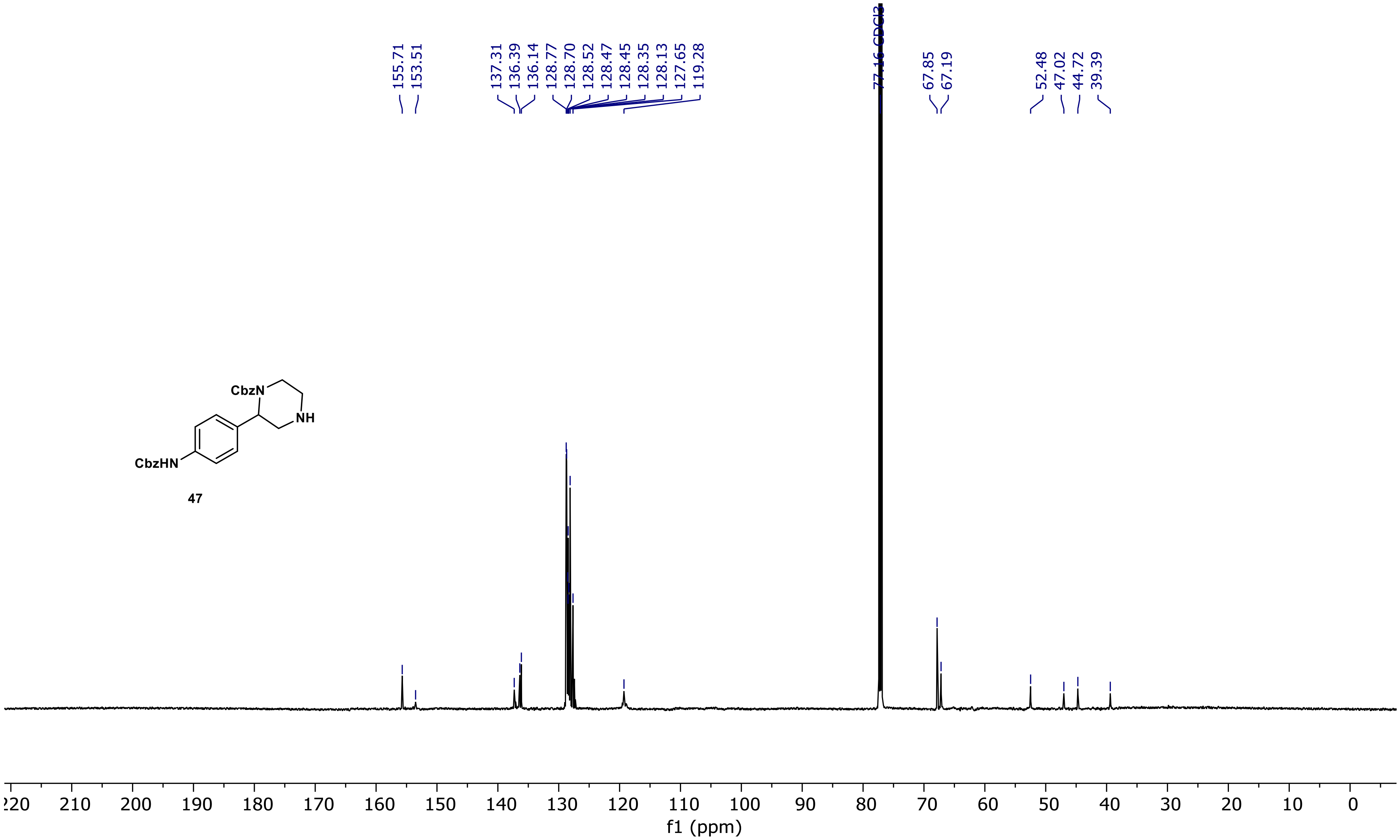

47

^{1}H NMR of dihydroimidazopyridinium 48

$CDCl_3$, 23 ºC

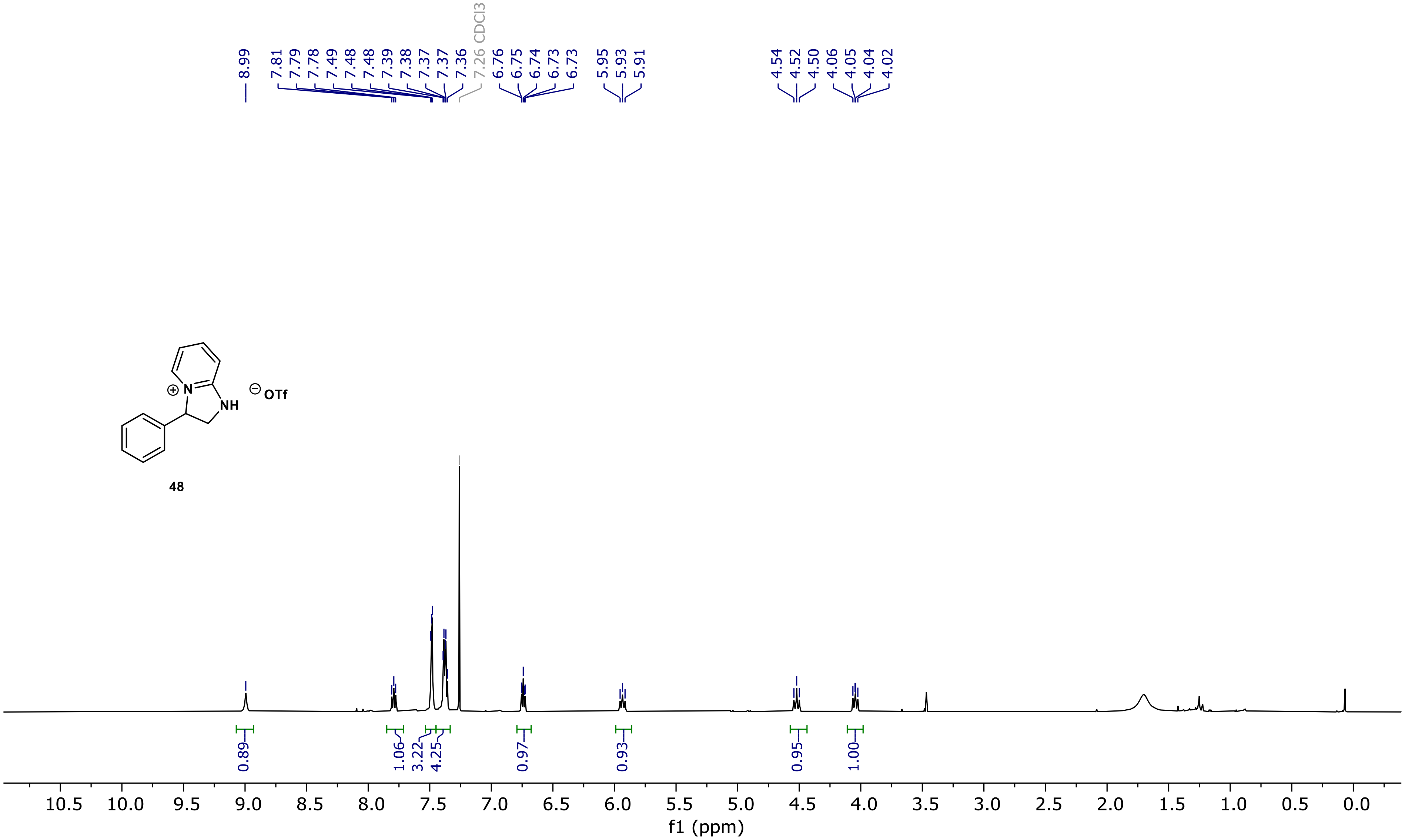

^{13}C NMR of dihydroimidazopyridinium 48

$CDCl_3$, 23 ºC

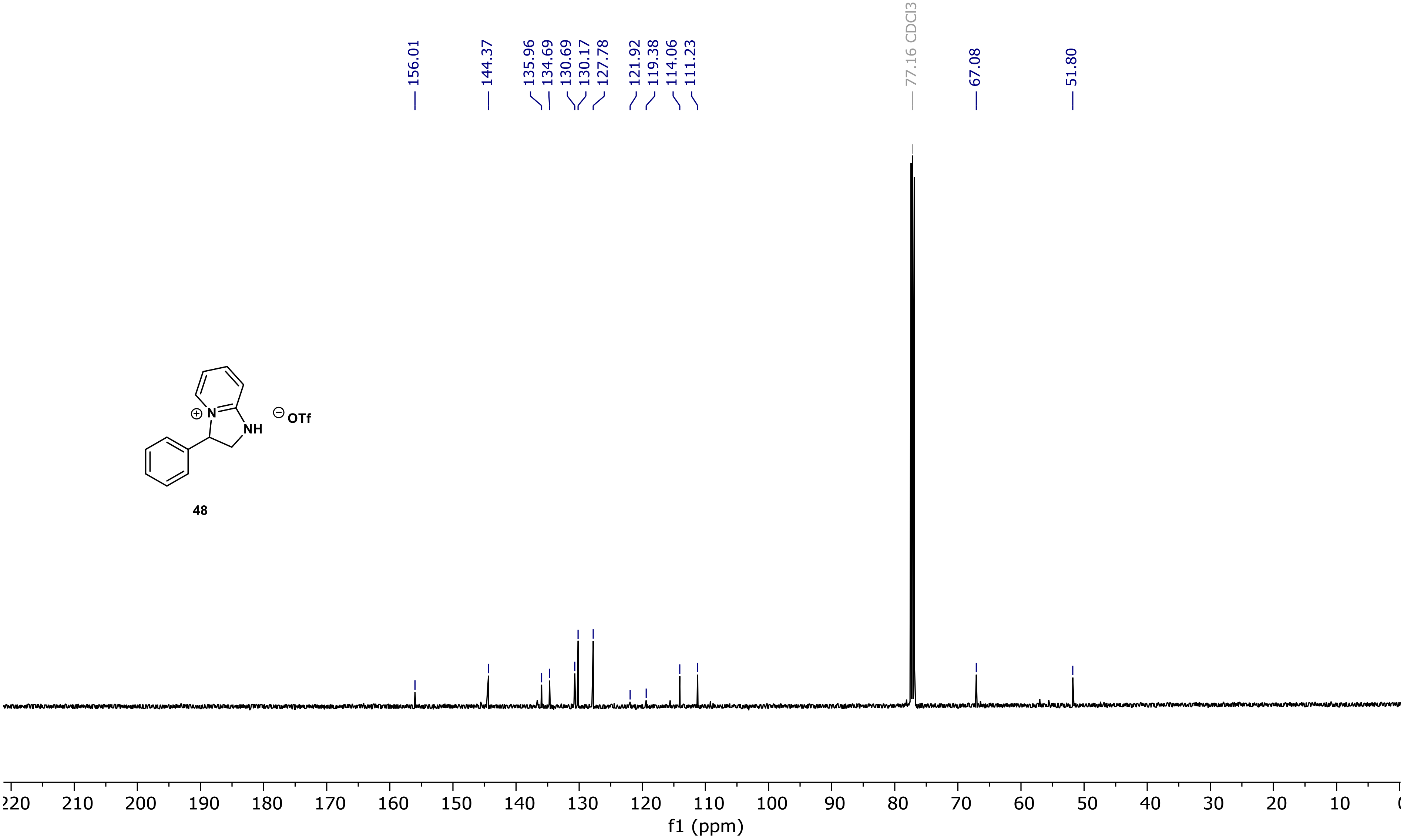

^{19}F NMR of dihydroimidazopyridinium 48

$CDCl_3$, 23 ºC

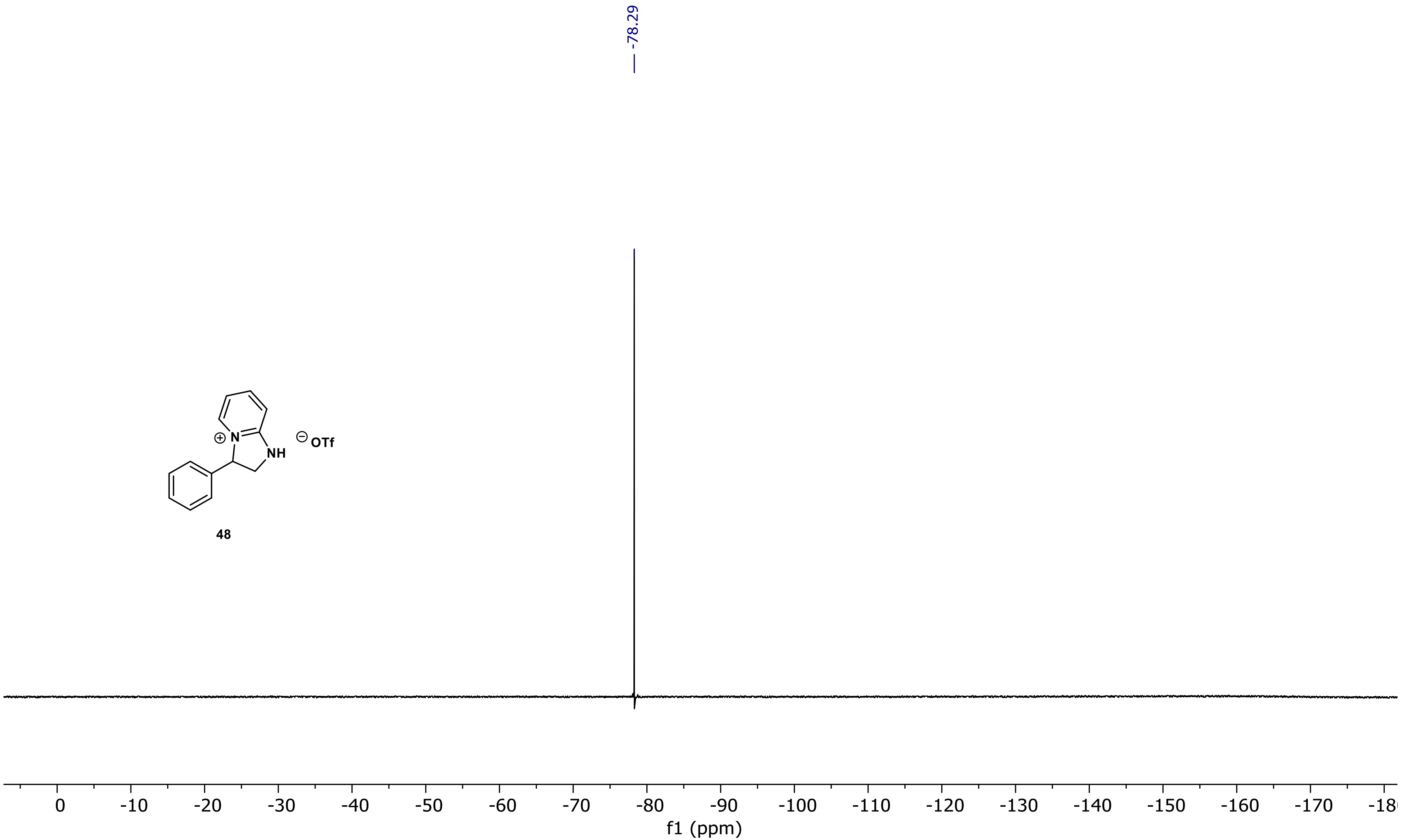

^{1}H NMR of dihydroimidazopyridinium 49

$CDCl_3$, 23 ºC

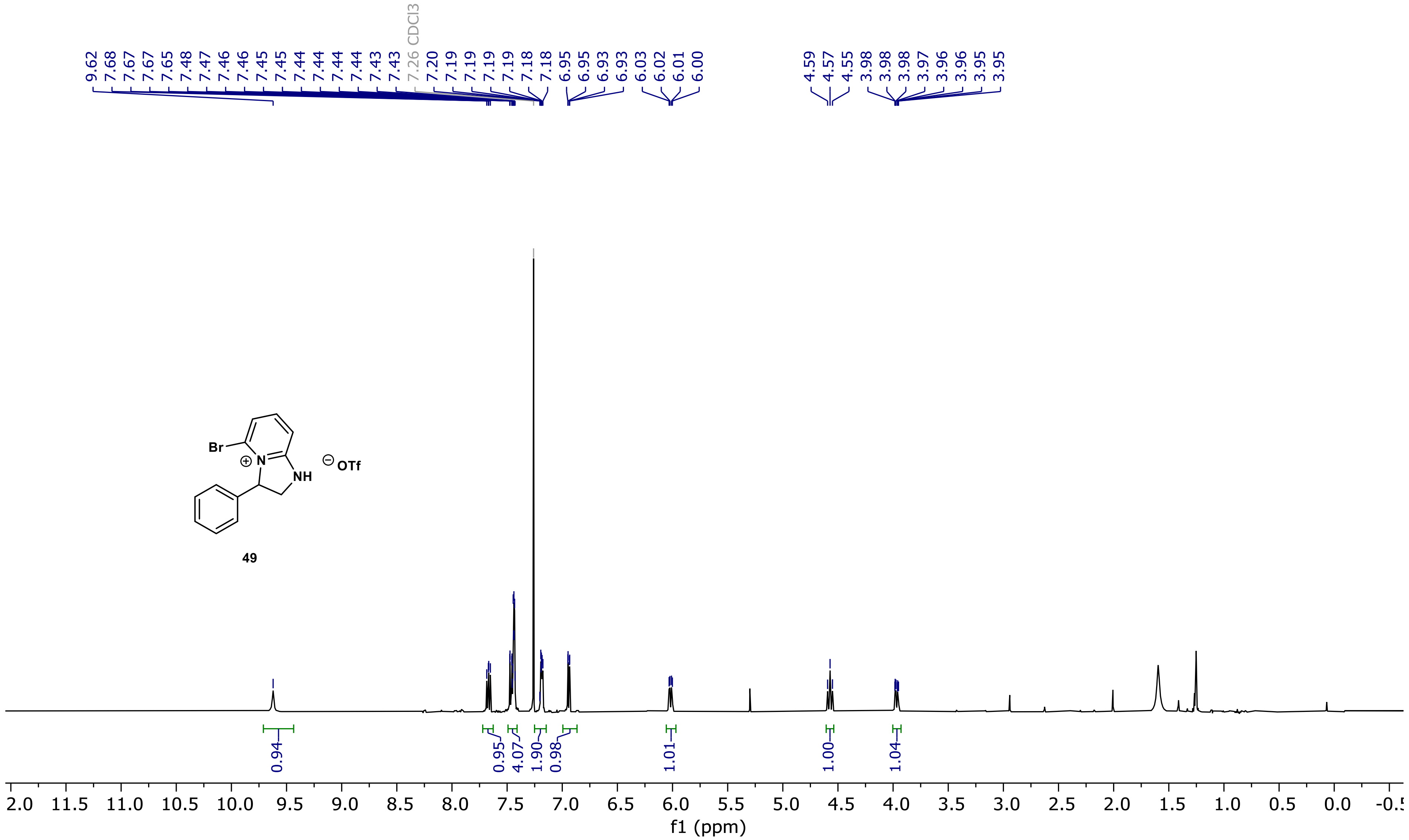

^{13}C NMR of dihydroimidazopyridinium 49

$CDCl_3$, 23 ºC

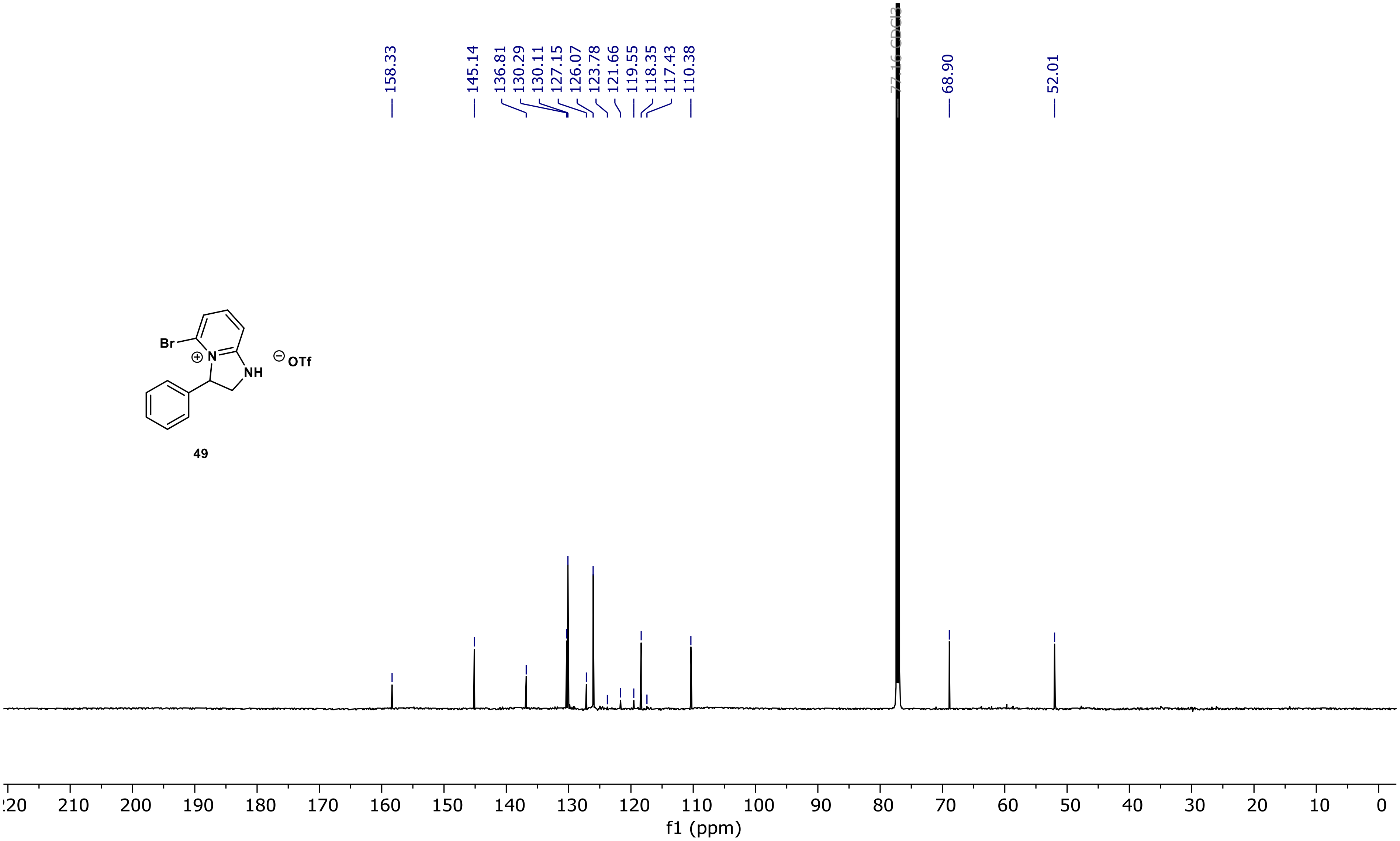

^{19}F NMR of dihydroimidazopyridinium 49

CDCl$_3$, 23 ºC

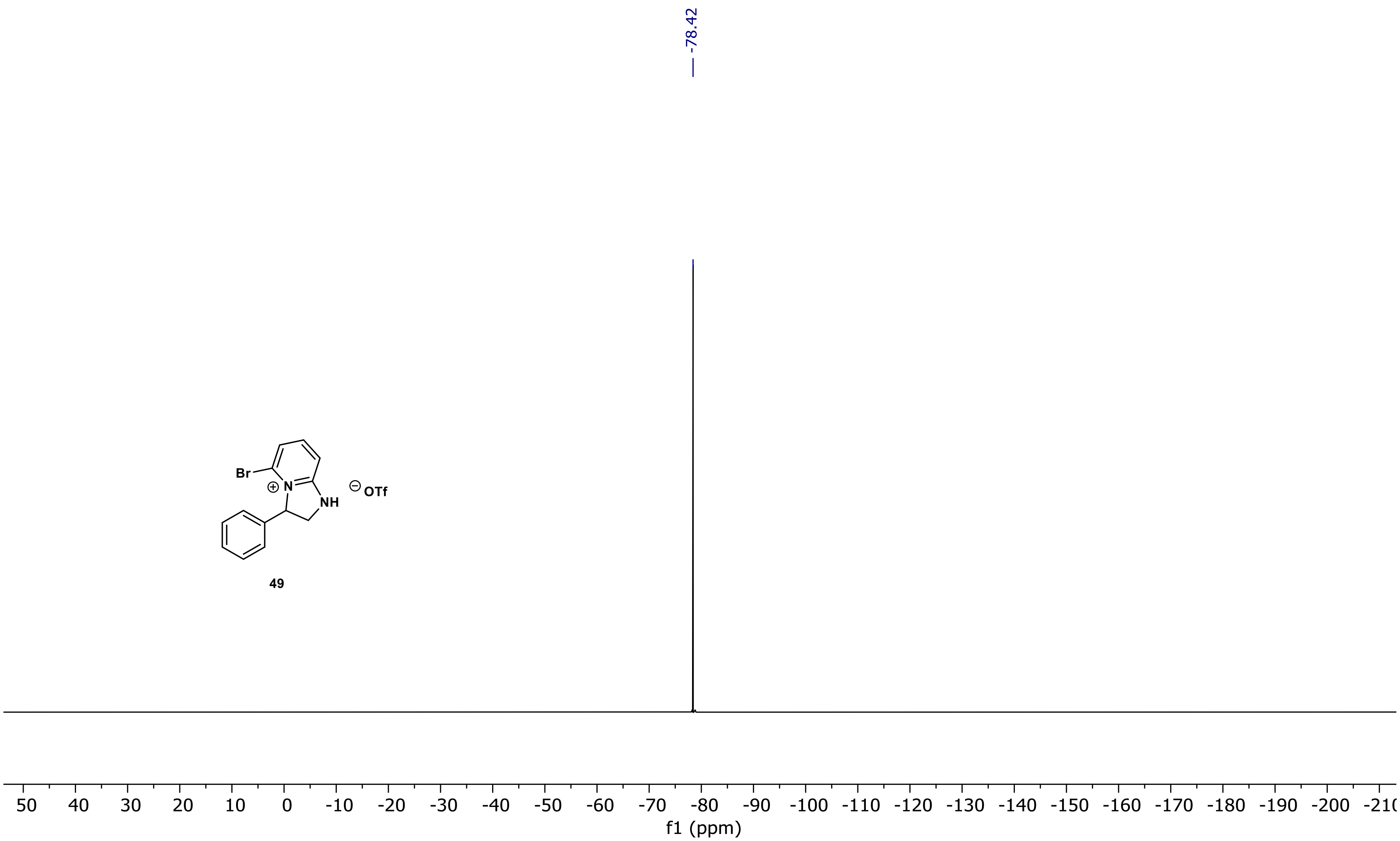

^{1}H NMR of dihydrooxazole 50

$CDCl_3$, 23 ºC

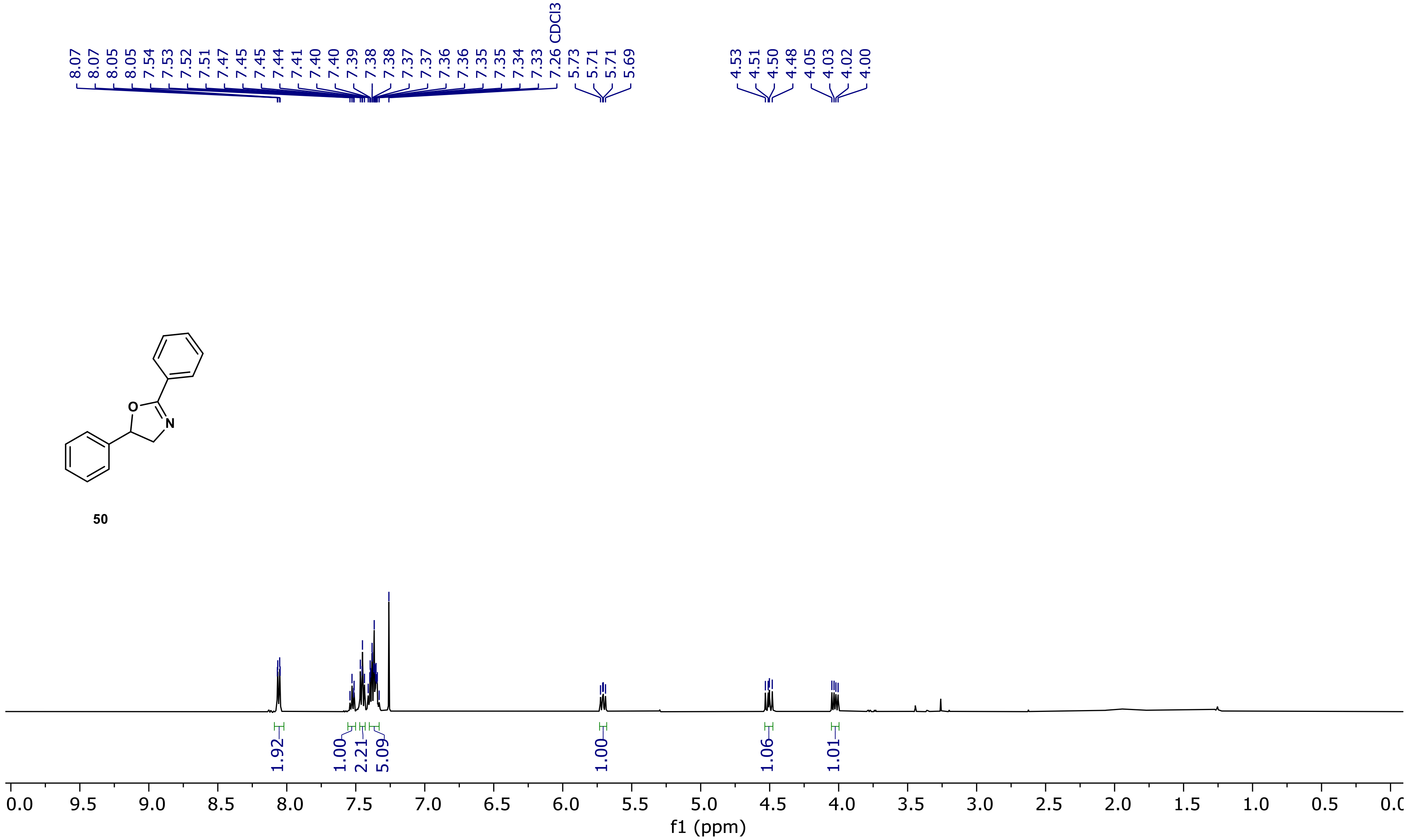

50

^{13}C NMR of dihydrooxazole 50

$CDCl_3$, 23 ºC

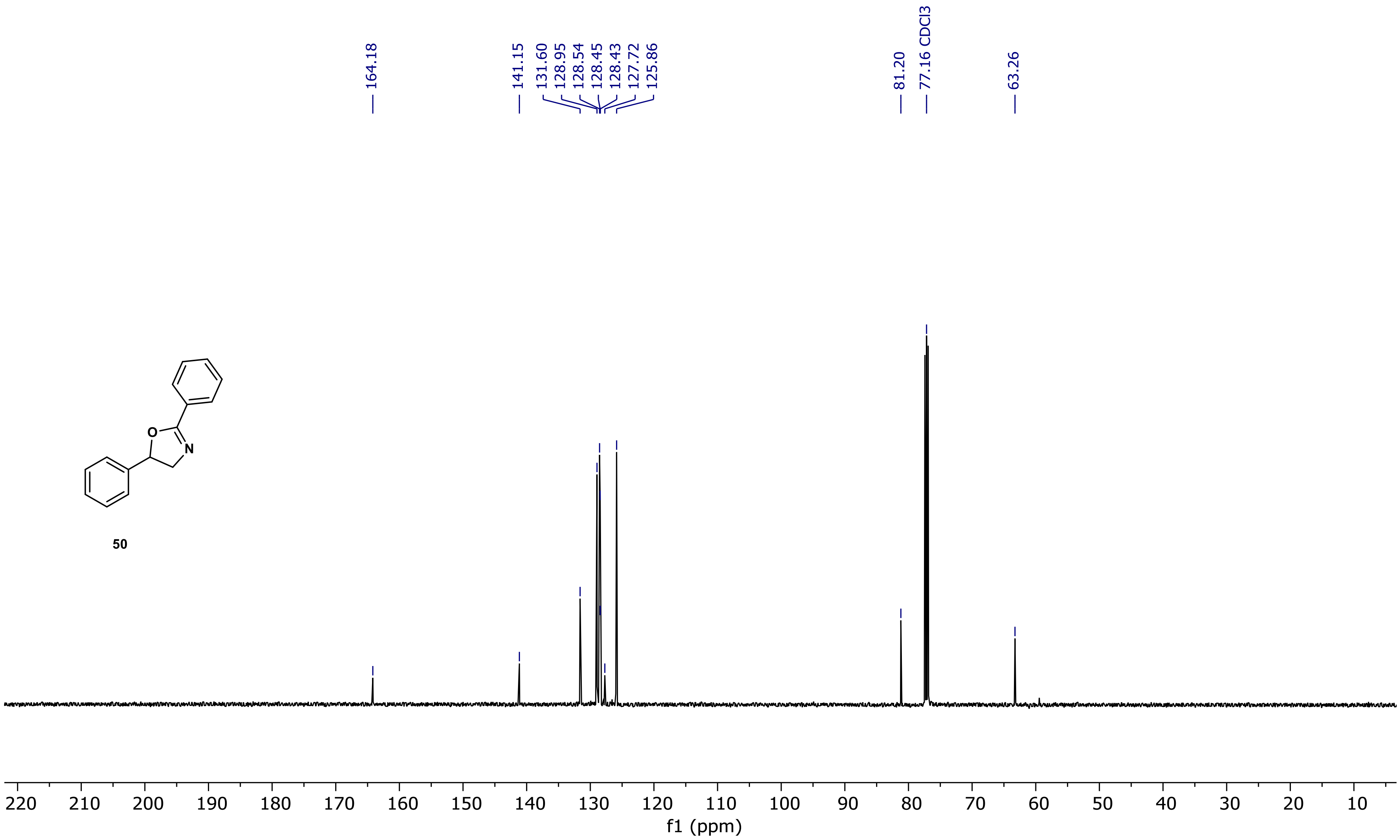

^{1}H NMR of dihydrooxazole 51

$CDCl_3$, 23 ºC

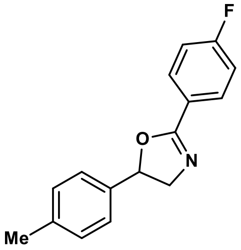

51

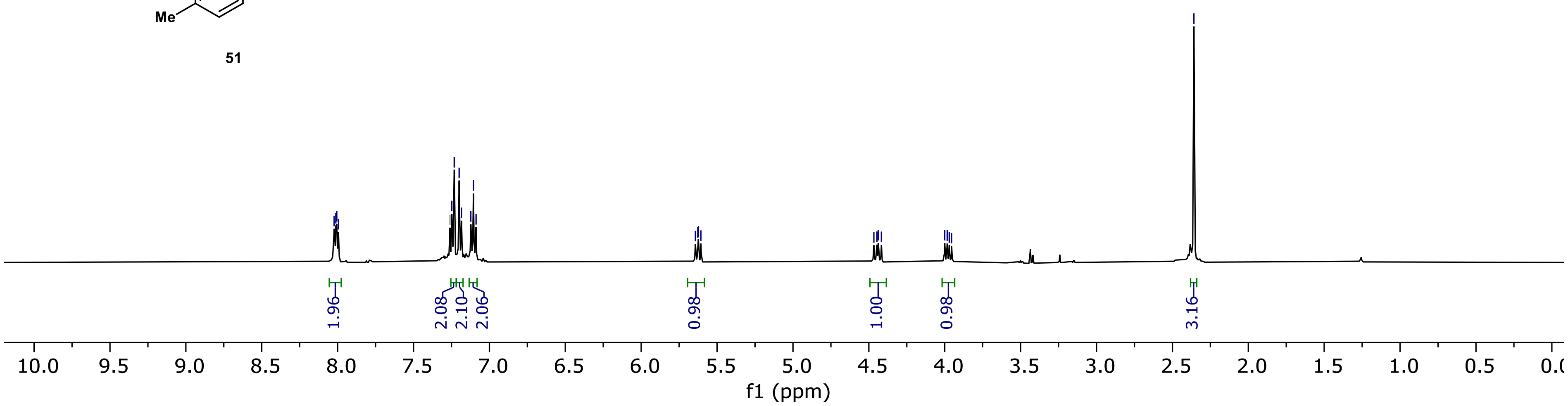

^{13}C NMR of dihydrooxazole 51

CDCl$_3$, 23 ºC

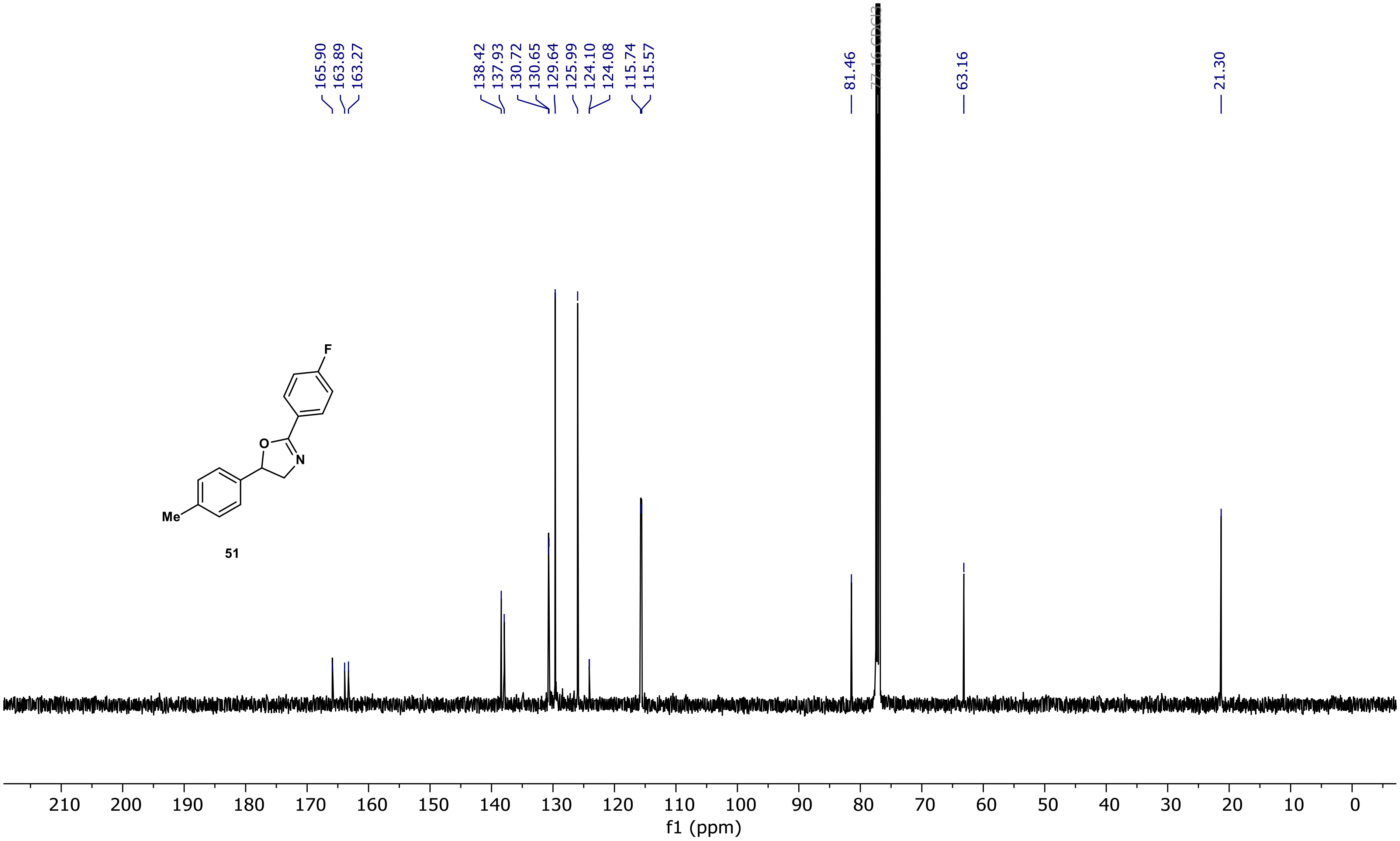

^{19}F NMR of dihydrooxazole 51

$CDCl_3$, 23 ºC

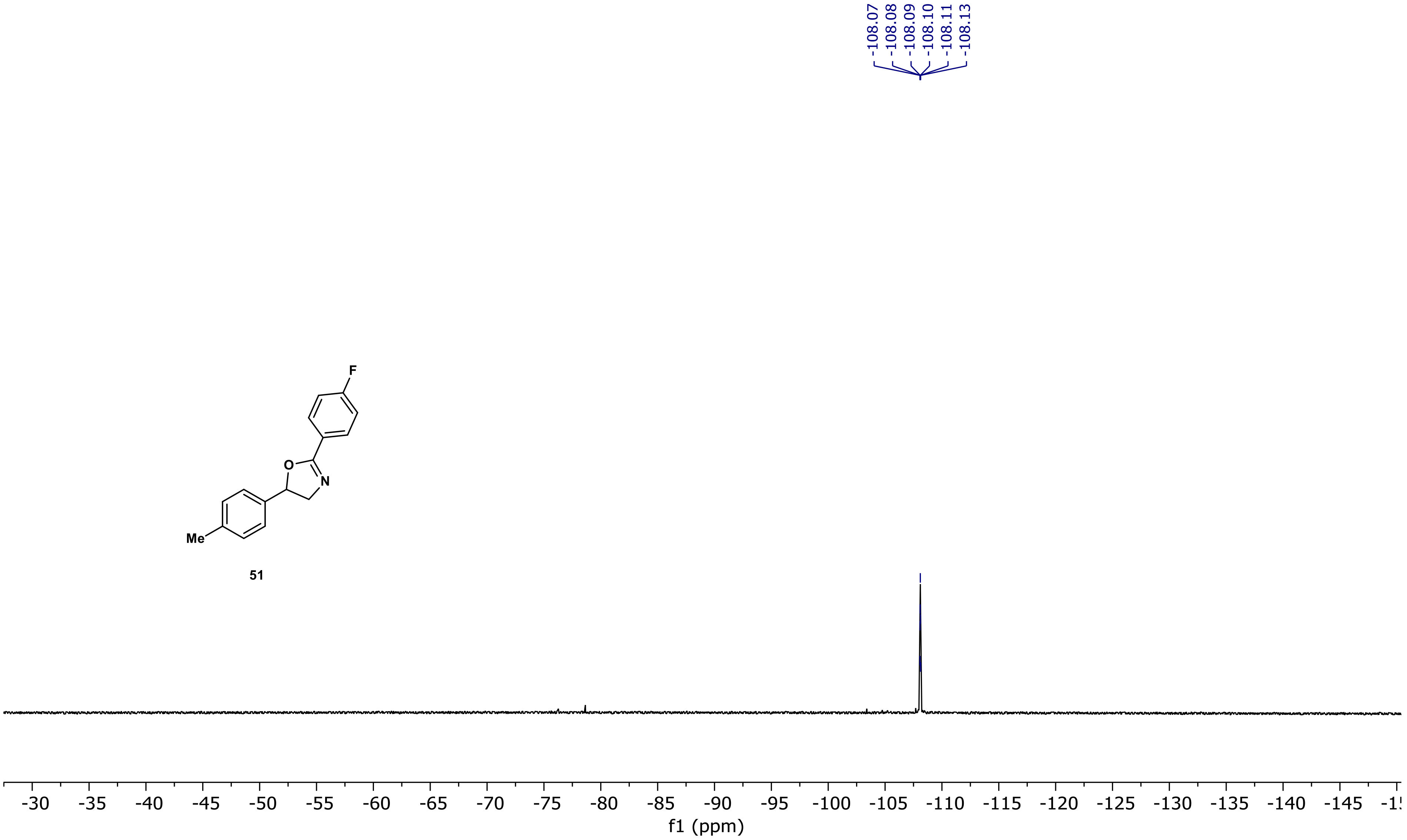

51

^{1}H NMR of dihydroindeno[2,1-d]oxazole 52

$CDCl_3$, 23 ºC

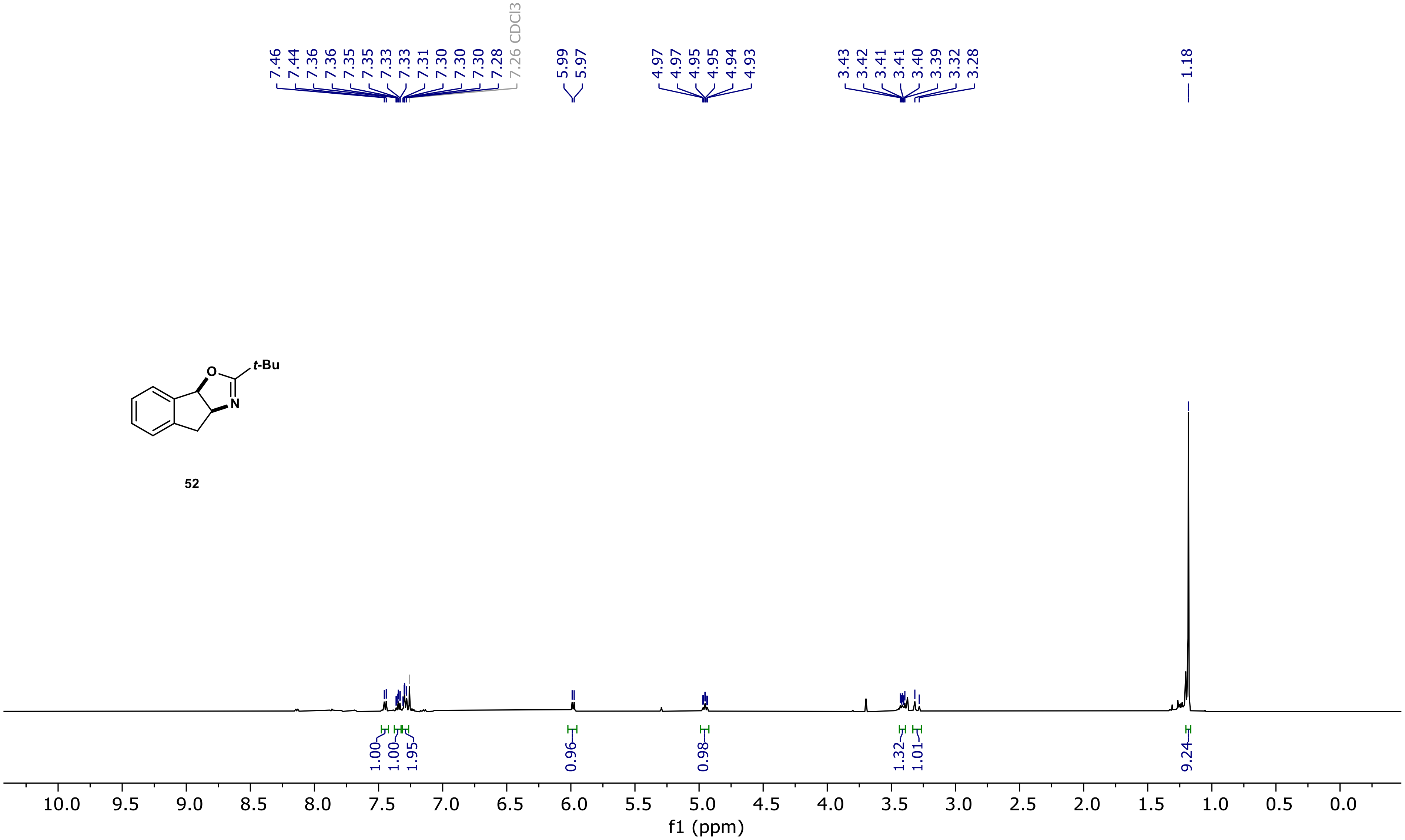

^{13}C NMR of dihydroindeno[2,1-d]oxazole 52

CD_2Cl_2, 23 ºC

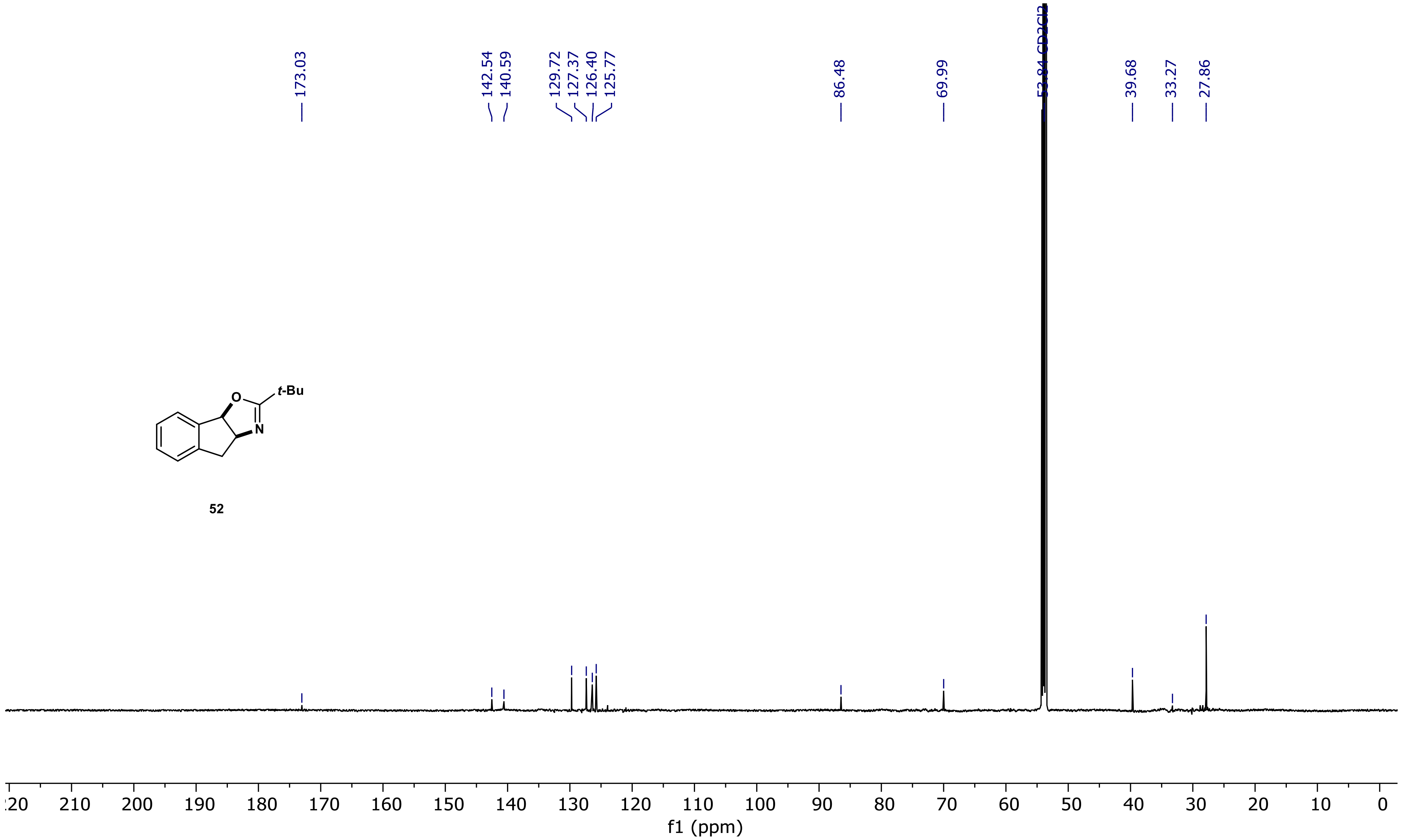

^{1}H NMR of 2,2-diphenyltetrahydrobenzofuran 53

CD_3CN, 23 ºC

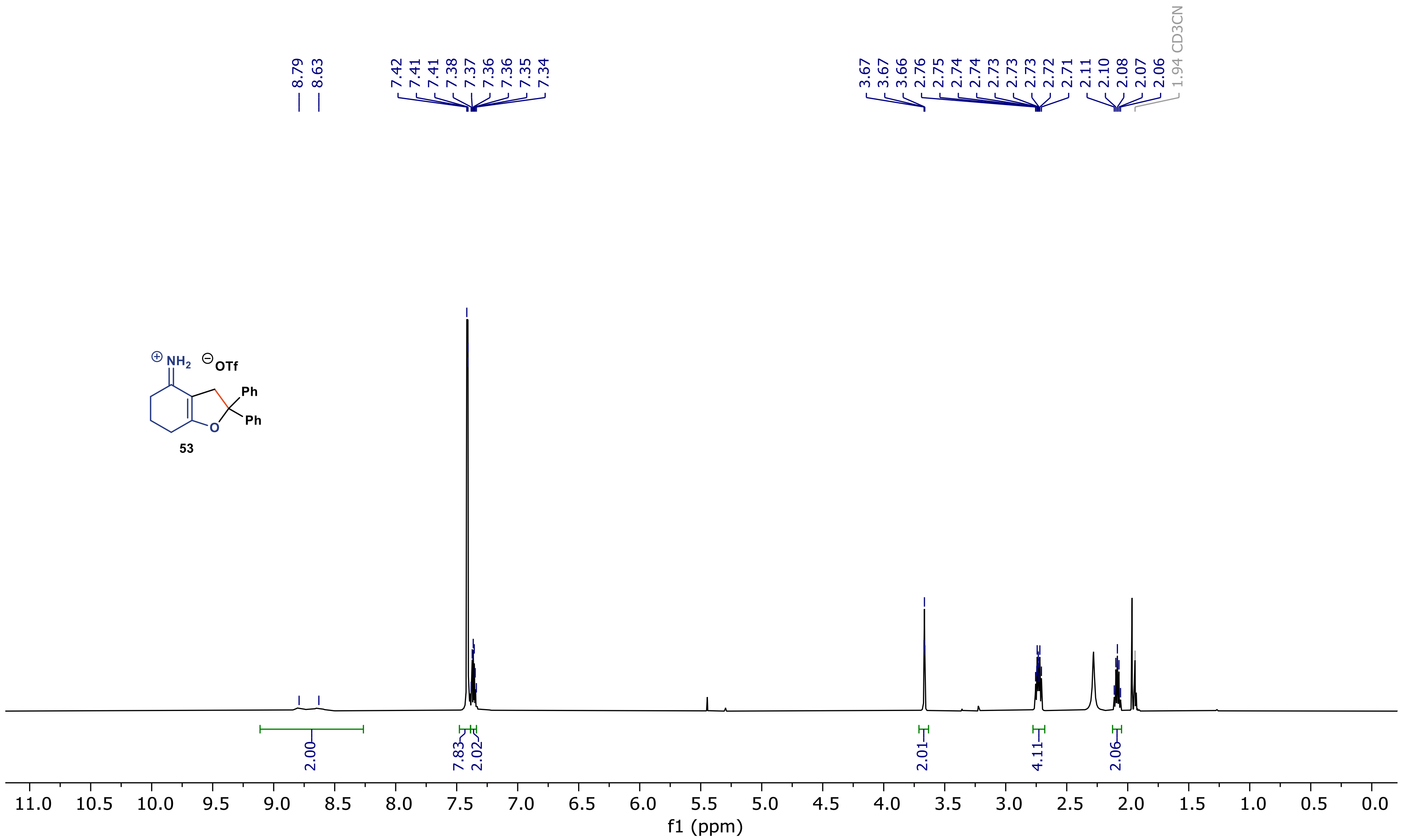

^{13}C NMR of 2,2-diphenyltetrahydrobenzofuran 53

CD_3CN, 23 ºC

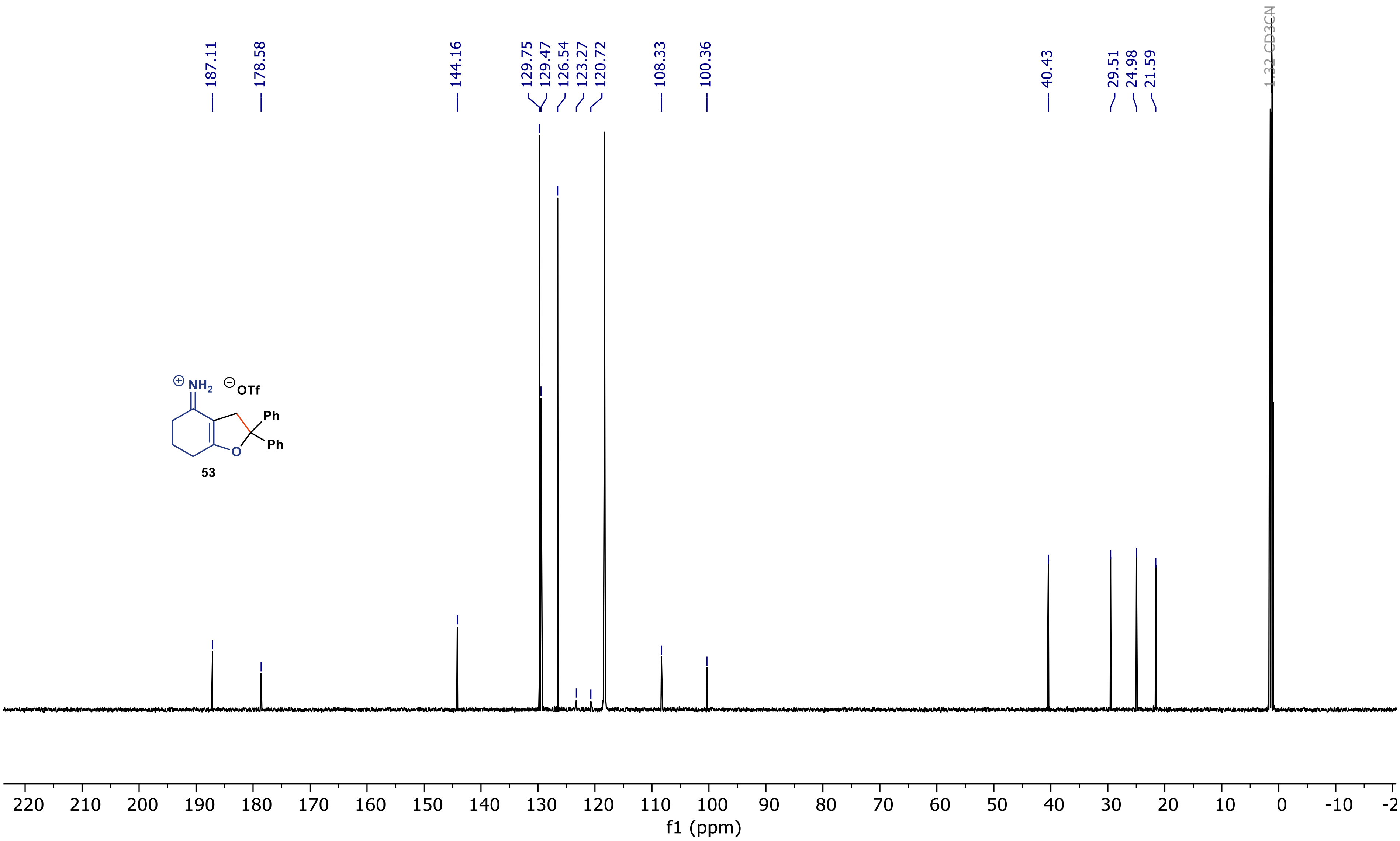

^{19}F NMR of 2,2-diphenyltetrahydrobenzofuran 53

CD_3CN, 23 ºC

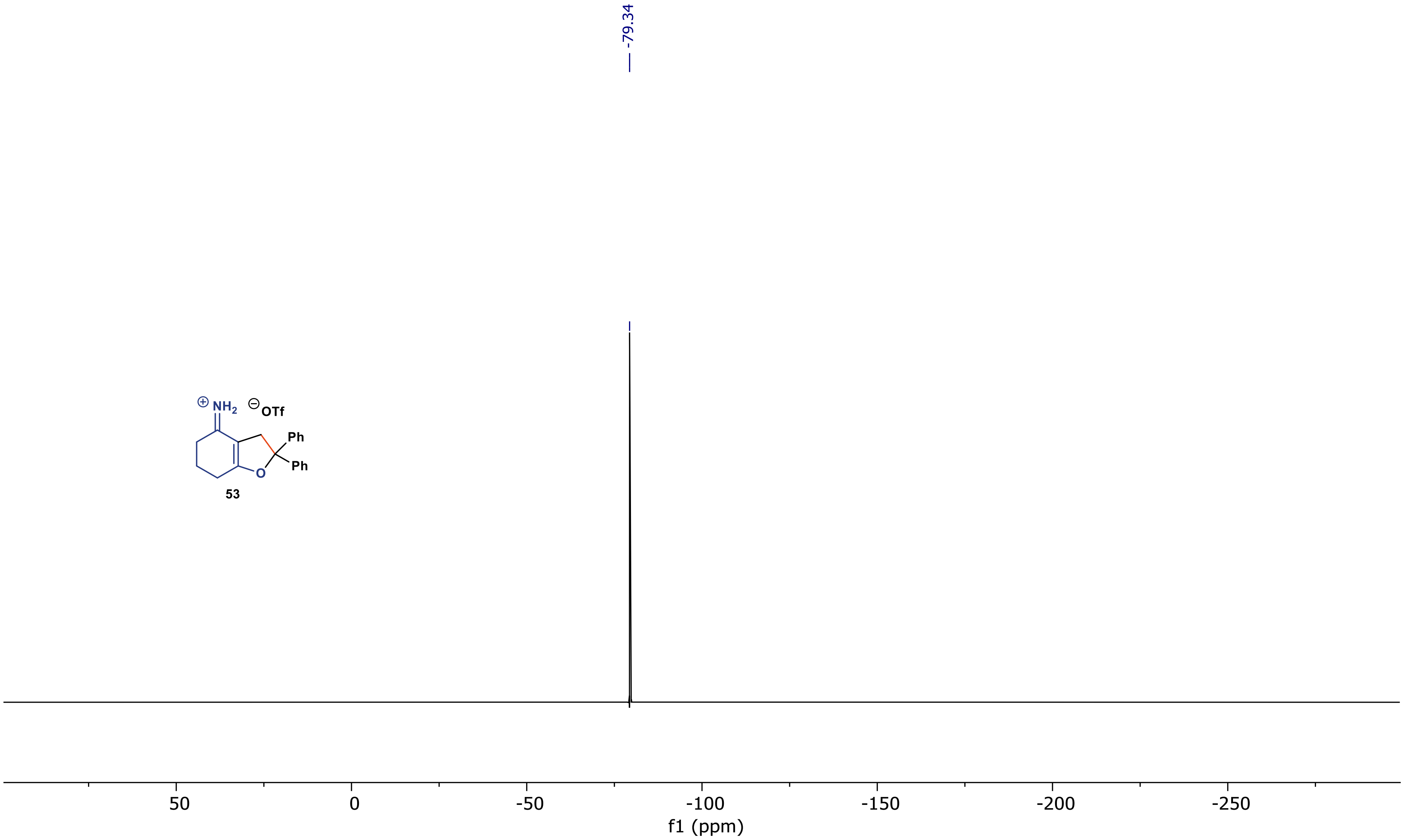

^{1}H NMR of dihydroimidazopyrimidinium 54

$CDCl_3$, 23 ºC

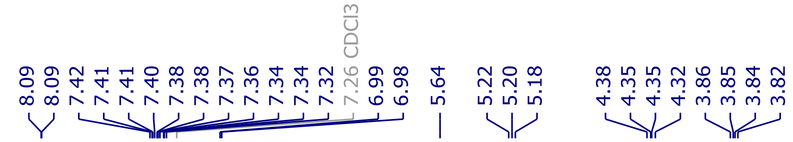

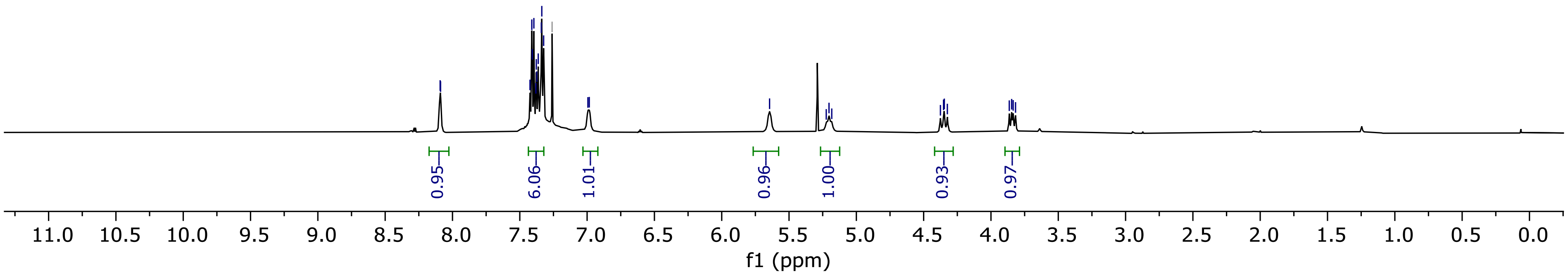

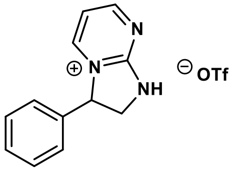

54

^{13}C NMR of dihydroimidazopyrimidinium 54

$CDCl_3$, 23 ºC

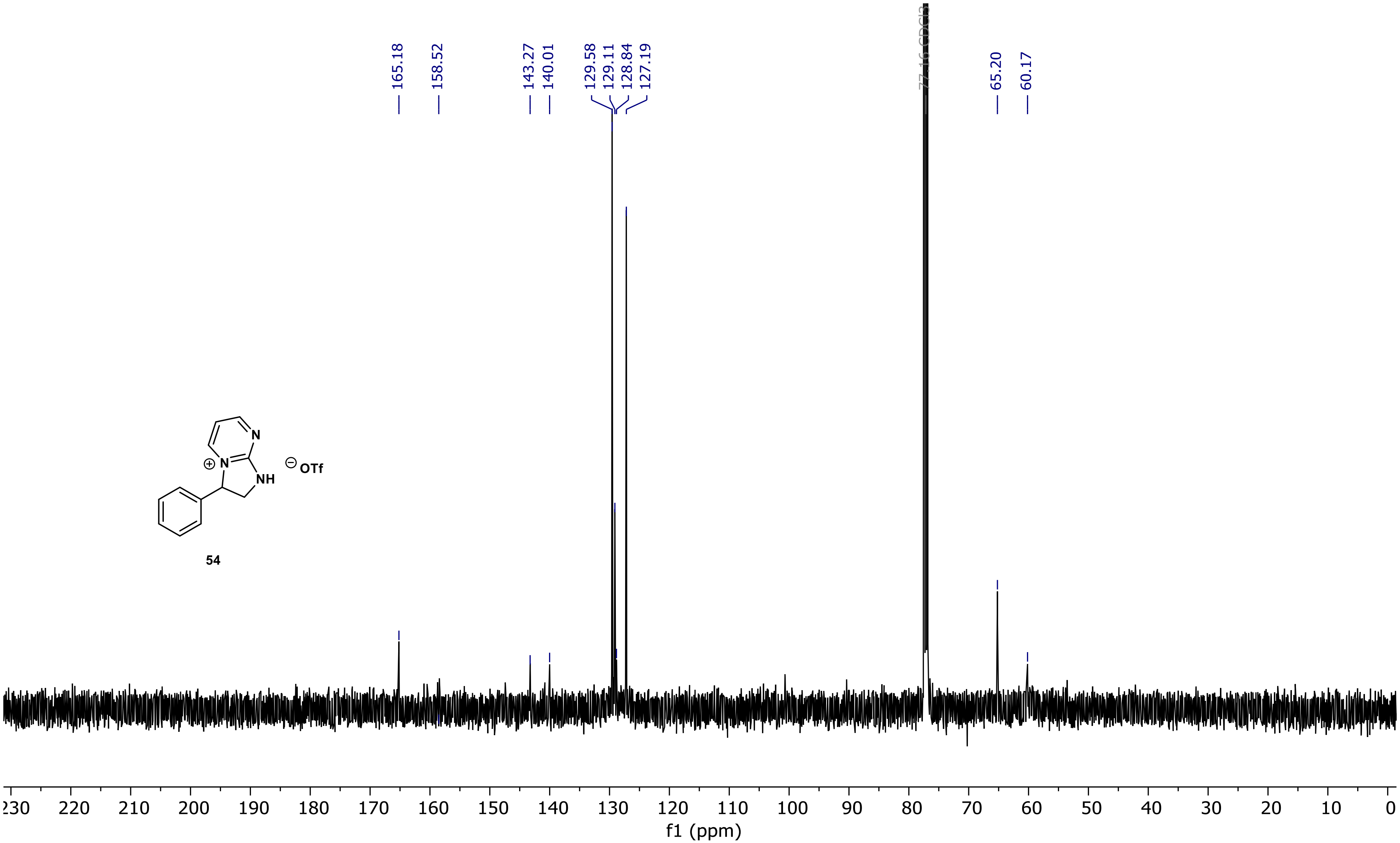

^{19}F NMR of dihydroimidazopyrimidinium 54

$CDCl_3$, 23 ºC

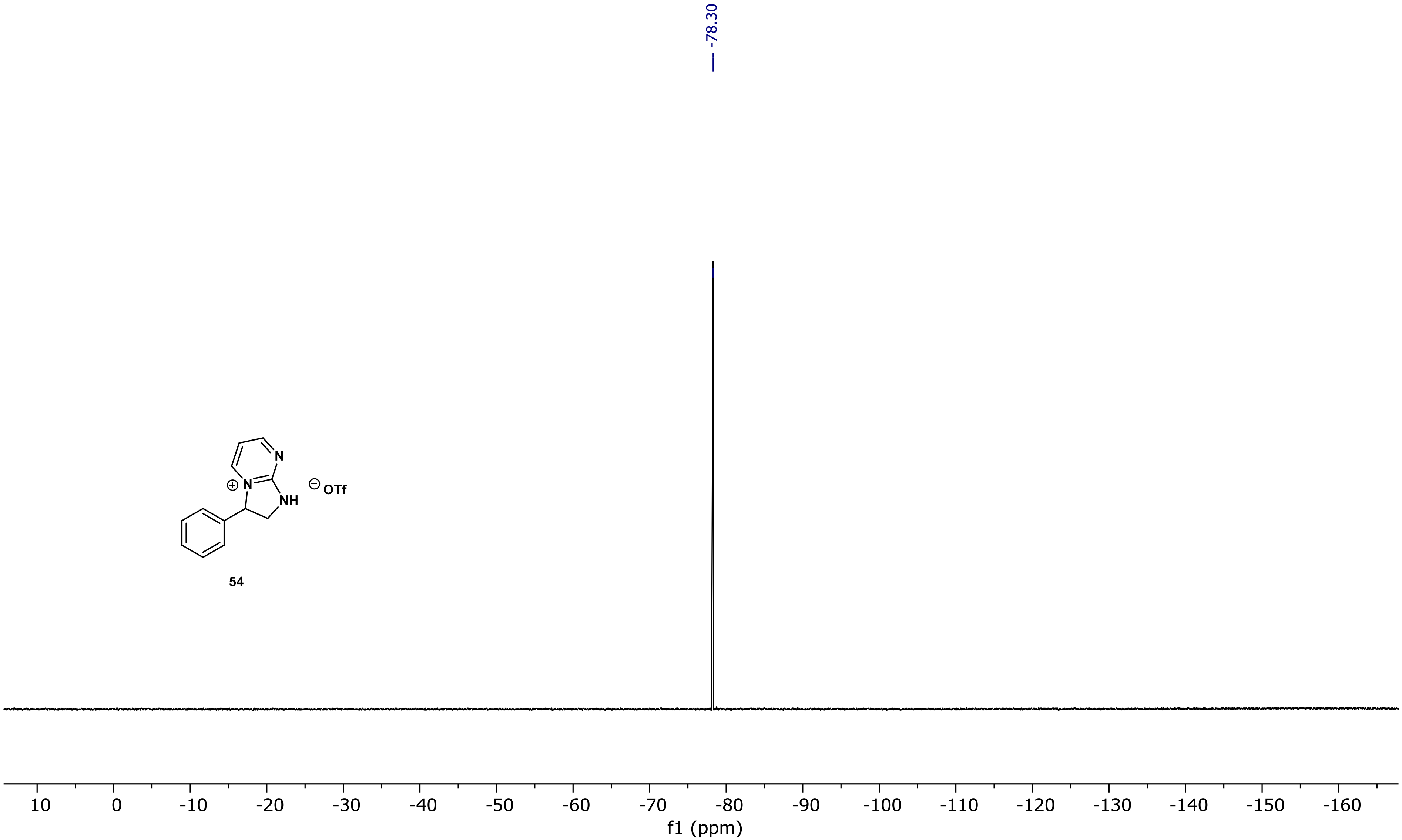

^{1}H NMR of dihydroimidazo[1,3,5]triazinone 55

CD_2Cl_2, 23 ºC

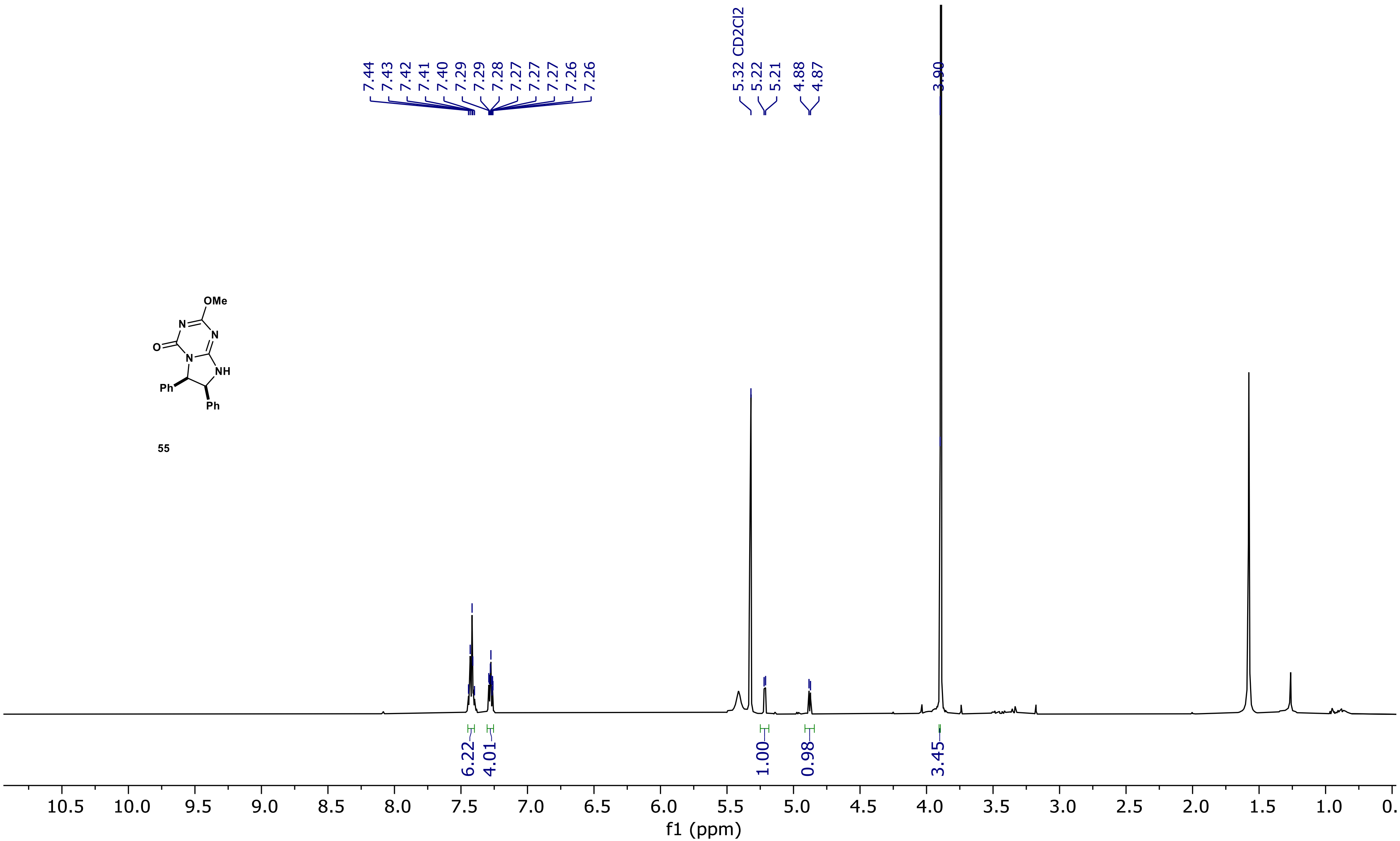

^{13}C NMR of dihydroimidazo[1,3,5]triazinone 55

d$_6$-DMSO, 23 ºC

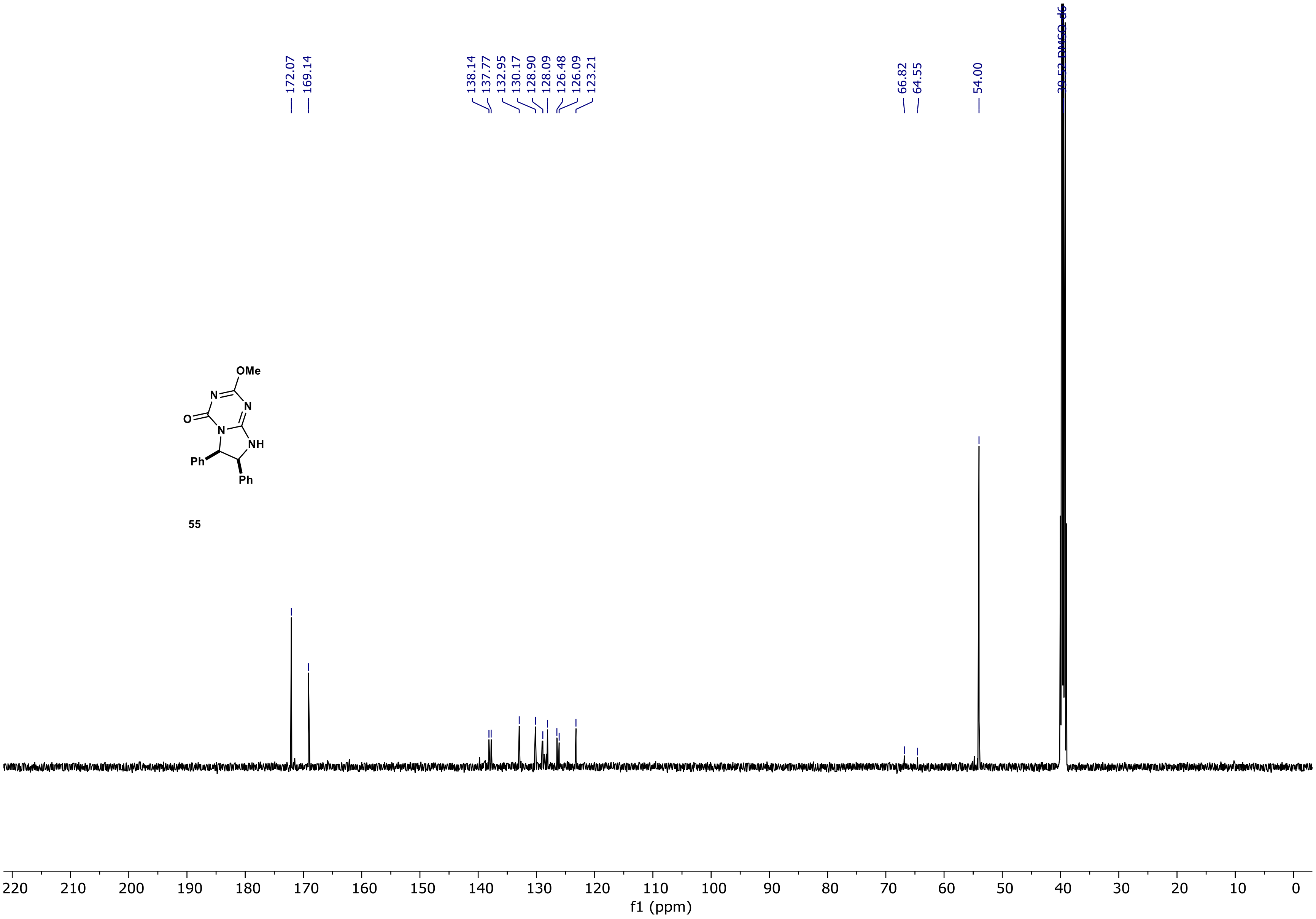

NOESY of dihydroimidazo[1,3,5]triazinone 55

CD3CN, 23 °C

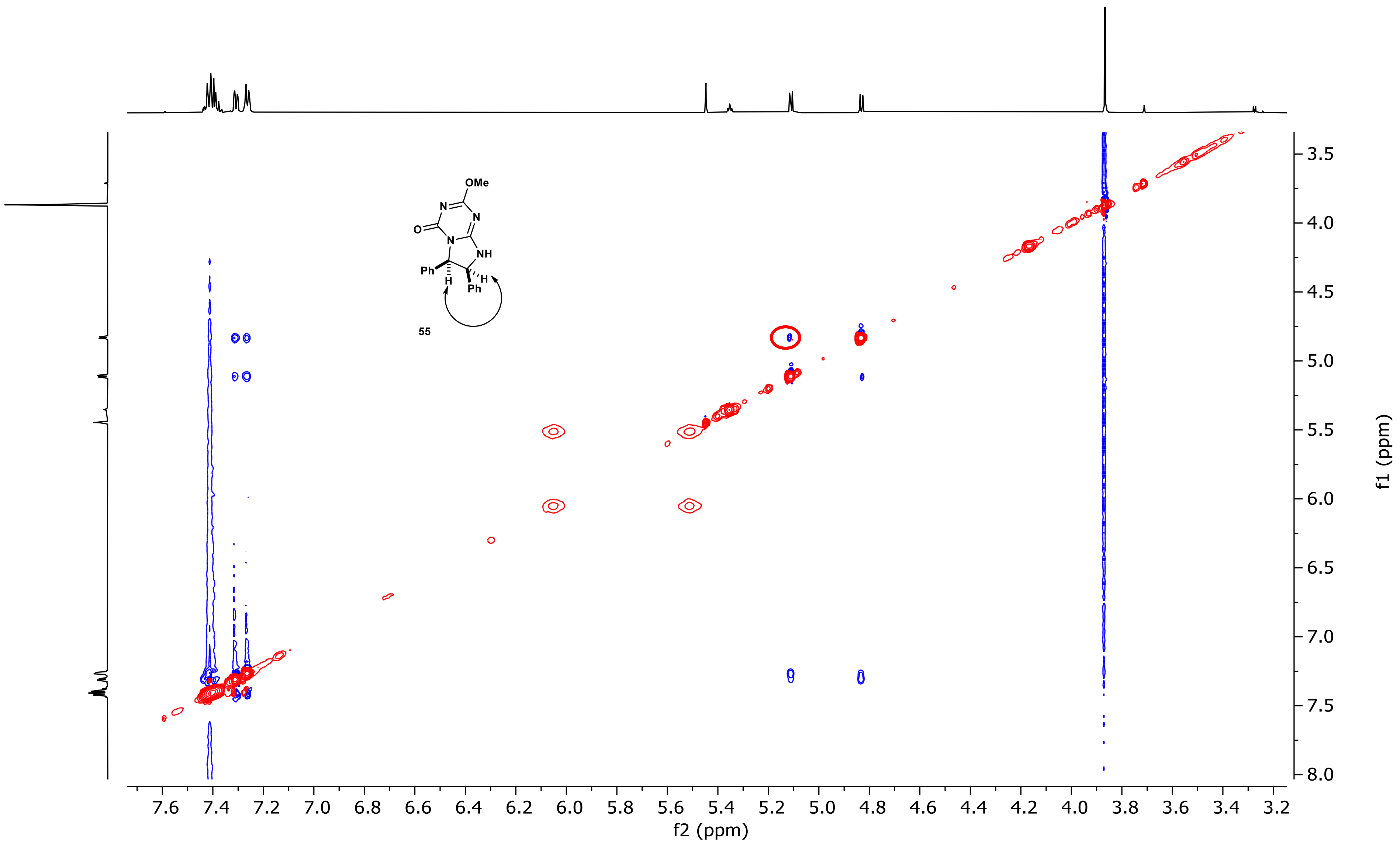

^{1}H NMR of cyclopropane ring opened product 56

CD_2Cl_2, 23 ºC

a mixture of stereoisomers with 1:1 E:Z

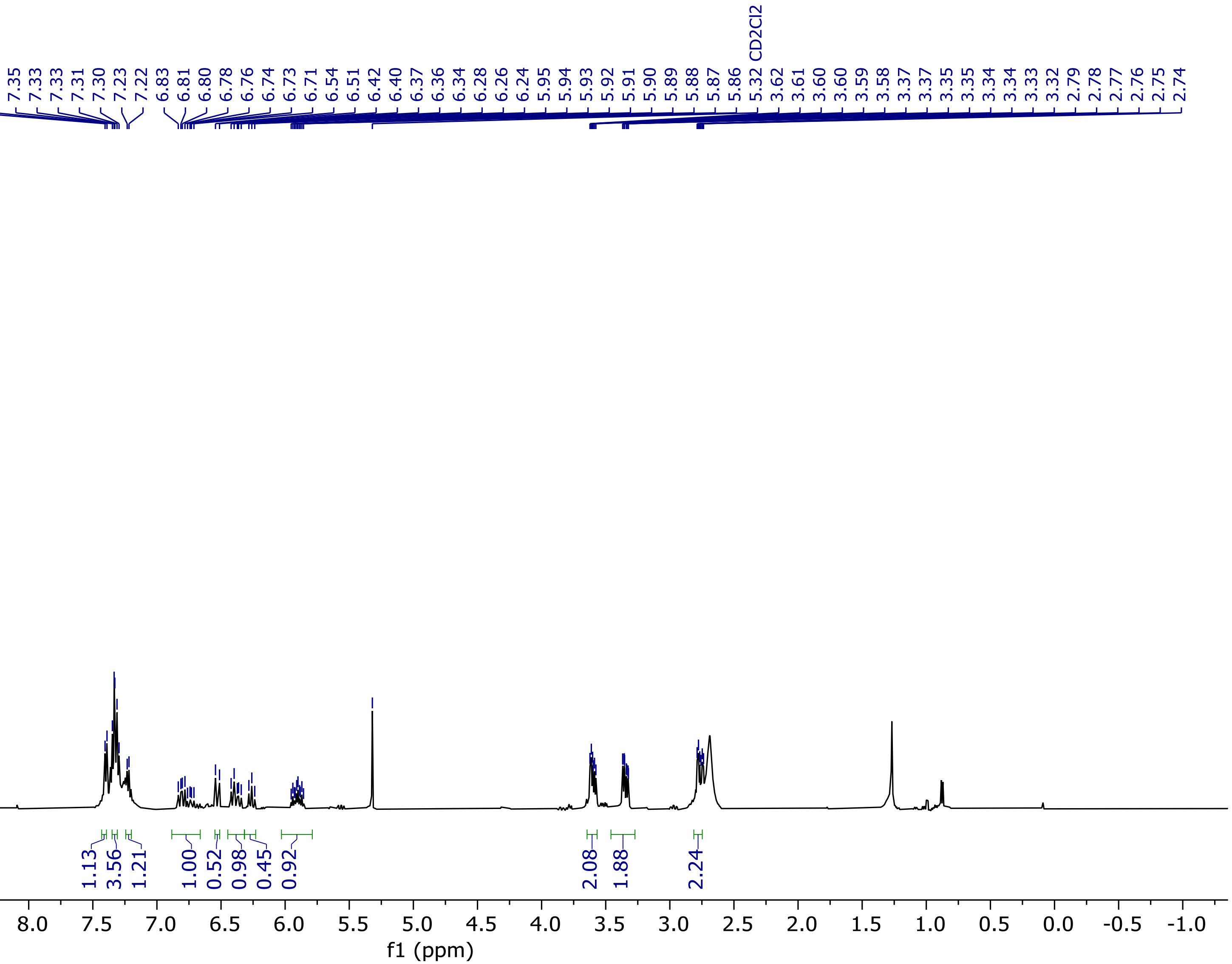

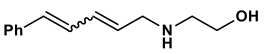

56

^{13}C NMR of cyclopropane ring opened product 56

CD_2Cl_2, 23 ºC

a mixture of stereoisomers with 1:1 E:Z

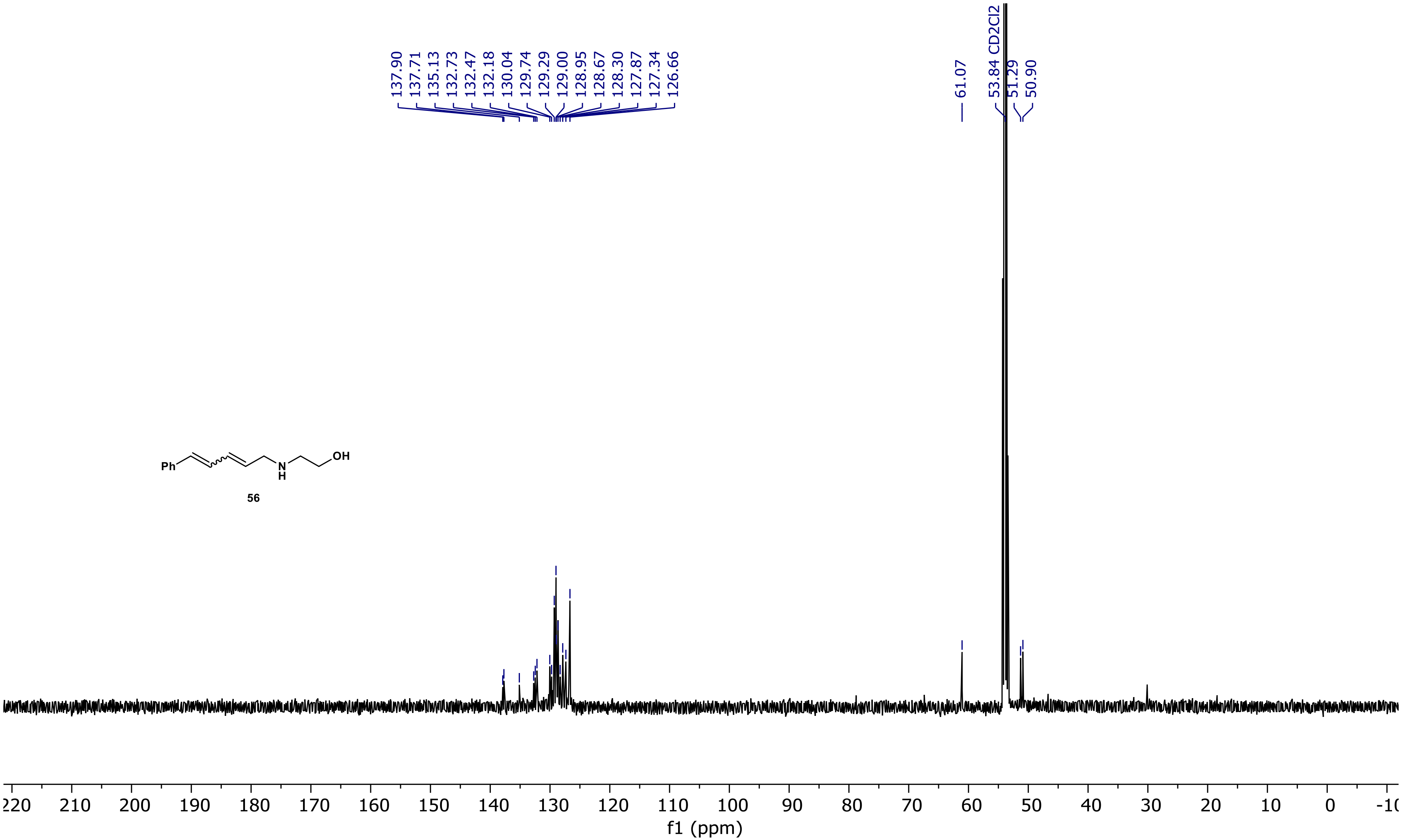

^{1}H NMR of 1,6-diene radical trap product 57

CDCl$_3$, 23 ºC

a mixture of diastereoisomers with 2:1 dr

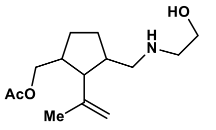

57

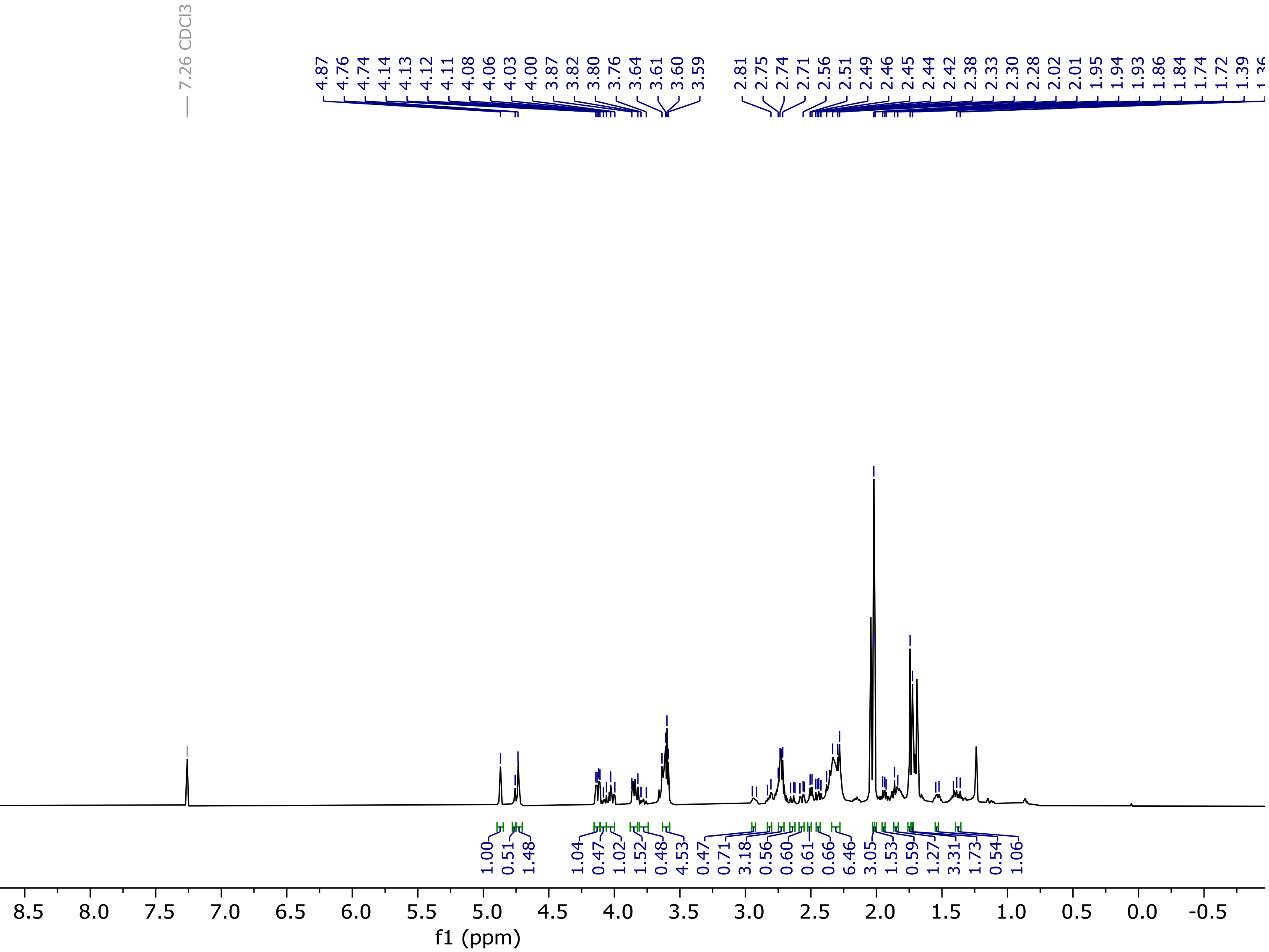

^{13}C NMR of 1,6-diene radical trap product 57

$CDCl_3$, 23 ºC

a mixture of diastereoisomers with 2:1 dr

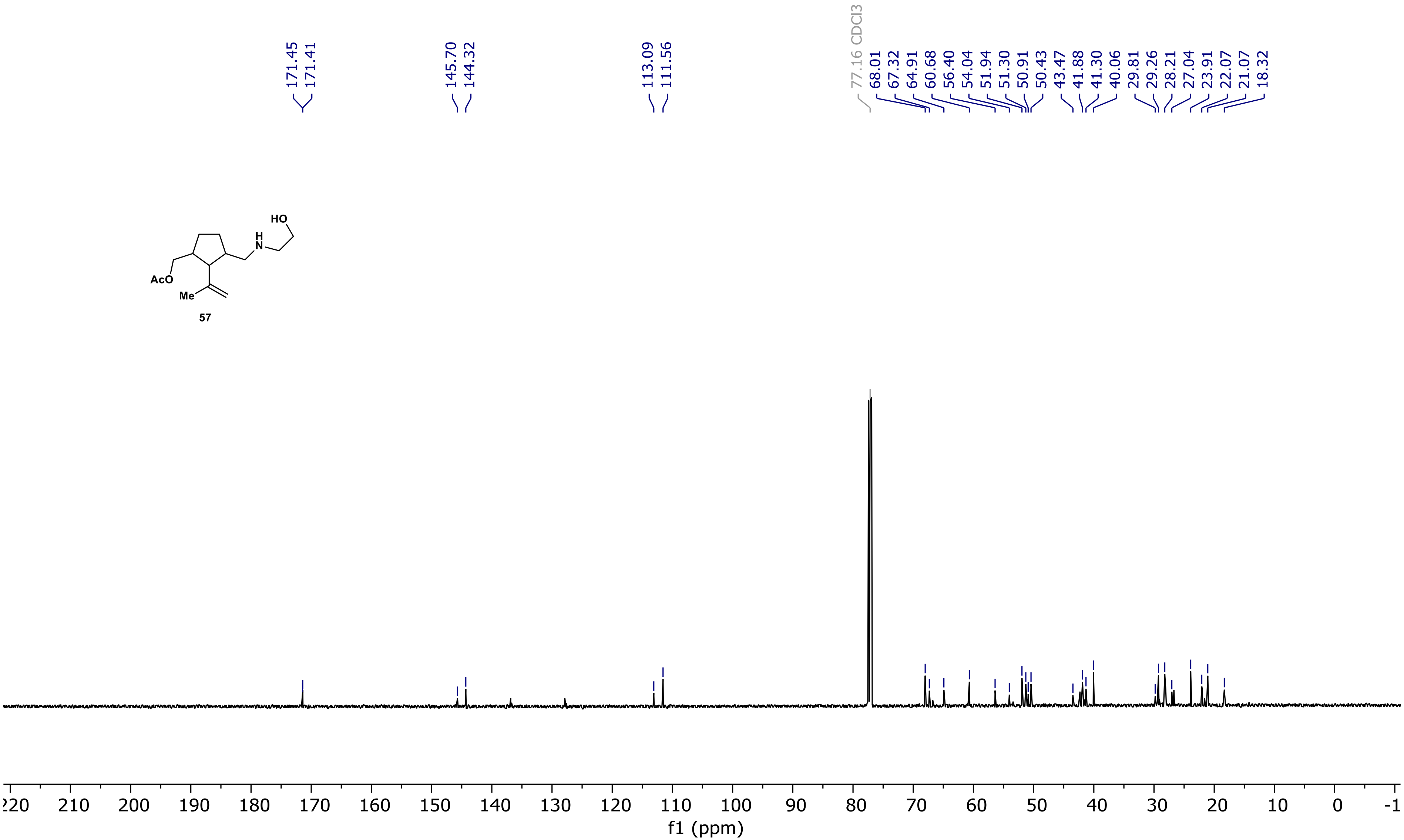

REFERENCES

1. Fulmer, G. R. et al. NMR Chemical Shifts of Trace Impurities: Common Laboratory Solvents, Organics, and Gases in Deuterated Solvents Relevant to the Organometallic Chemist. *Organometallics* **29**, 2176–2179 (2010).

2. He, Z., Pulis, A. P. & Procter, D. J. The Interrupted Pummerer Reaction in a Sulfoxide-Catalyzed Oxidative Coupling of 2-Naphthols. *Angew. Chem. Int. Ed.* **58**, 7813–7817 (2019).

3. Yu, H., Li, Z. & Bolm, C. Iron(II)-Catalyzed Direct Synthesis of NH Sulfoximines from Sulfoxides. *Angew. Chem. Int. Ed.* **57**, 324–327 (2018).

4. Cao, H. et al. Photo-induced Decarboxylative Heck-Type Coupling of Unactivated Aliphatic Acids and Terminal Alkenes in the Absence of Sacrificial Hydrogen Acceptors. *J. Am. Chem. Soc.* **140**, 16360–16367 (2018).

5. Zhao, Z., To, A. J. & Murphy, G. K. Difluorinative ring expansions of benzo-fused carbocycles and heterocycles are achieved with p-(difluoroiodo)toluene. *Chem. Commun.* **55**, 14821–14824 (2019).

6. Shang, L. et al. Redox-Neutral α-Arylation of Alkyl Nitriles with Aryl Sulfoxides: A Rapid Electrophilic Rearrangement. *J. Am. Chem. Soc.* **139**, 4211–4217 (2017).

7. Prakash, G. K. S., Weber, C., Chacko, S. & Olah, G. A. New Electrophilic Difluoromethylating Reagent. *Org. Lett.* **9**, 1863–1866 (2007).

8. Devery III, J. J. et al. Ligand functionalization as a deactivation pathway in a *fac*-Ir(ppy)$_3$-mediated radical addition. *Chem. Sci.* **6**, 537–541 (2015).

9. Ruffoni, A. et al. Practical and regioselective amination of arenes using alkyl amines. *Nat. Chem.* **11**, 426–433 (2019).

10. Peng, H.-L. & Callender, R. Mechanism for Fluorescence Quenching of Tryptophan by Oxamate and Pyruvate: Conjugation and Solvation-Induced Photoinduced Electron Transfer. *J. Phys. Chem. B* **122**, 6483–6490 (2018).

11. Zhang, Z.-Q., Meng, X.-Y., Sheng, J., Lan, Q. & Wang, X.-S. Enantioselective Copper-Catalyzed 1,5-Cyanotrifluoromethylation of Vinylcyclopropanes. *Org. Lett.* **21**, 8256–8260 (2019).
